# C─S Bond Formation of Aryl Germanes

**DOI:** 10.1002/anie.3776319

**Published:** 2026-08-26

**Authors:** Jaime Ponce‐de‐León, Markus D. Schoetz, Amit Dahiya, Susanna L. Kirsten, Kristina Deckers, Steffen Benkhoff, Franziska Schoenebeck

**Affiliations:** ^1^ Institute of Organic Chemistry RWTH Aachen University Aachen Germany

**Keywords:** modularity, organogermane, radical, site‐selectivity, thiolation

## Abstract

Aryl germanes have emerged as robust and orthogonal coupling partners, but the chemistry enabling their functionalization is still in its infancy. Herein, we report the first C─S bond formation of aryl germanes. In contrast to conventional C─S cross‐couplings of prefunctionalized arenes that largely rely on transition‐metal catalysis, this transformation proceeds in the absence of a metal catalyst at room temperature and tolerates air and moisture. The reaction is orthogonal to common coupling handles, including aryl halides and aryl silanes, enabling selective thiolations in multifunctional molecules. Mechanistic studies support a radical‐based aromatic substitution pathway.

## Graphical abstract

Site‐selective C─S bond formation of aryl germanes, even in the presences of other functional groups such as silane or boron derivatives, is described. The protocol operates under metal‐free conditions at room temperature without the need of inert atmosphere.

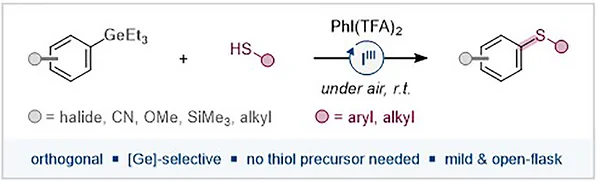

## 1 Introduction

Trialkylgermanes (GeR_3_) have emerged as versatile and orthogonal aryl coupling partners within contemporary synthetic chemistry [1, 2, 3, 4]. These substituents are non‐toxic [5], stable and straightforward to introduce [6, 7, 8, 9, 10]. They are unreactive under typical Pd^(0)^/Pd^(II)^ catalysis, allowing aryl germanes to persist through complex reaction sequences without interfering with standard cross‐coupling processes [2, 11]. At the same time, recent advances have demonstrated that aryl germanes can undergo selective activation even in the presence of traditionally more reactive functional groups such as aryl halides, boronic acids/esters, silanes, or stannanes [12, 13, 14, 15]. The combination of robustness and controlled reactivity has enabled new C─C and C─O bond‐forming reactions [2, 16], as well as halogenation [17] and nitration [18], highlighting the potential of aryl germanes as programmable handles in modular synthesis (Figure 1a). Despite this progress, there are still relatively few transformations available for these motifs and no example of C─S bond formation from aryl germanes has been described.

**FIGURE 1:**
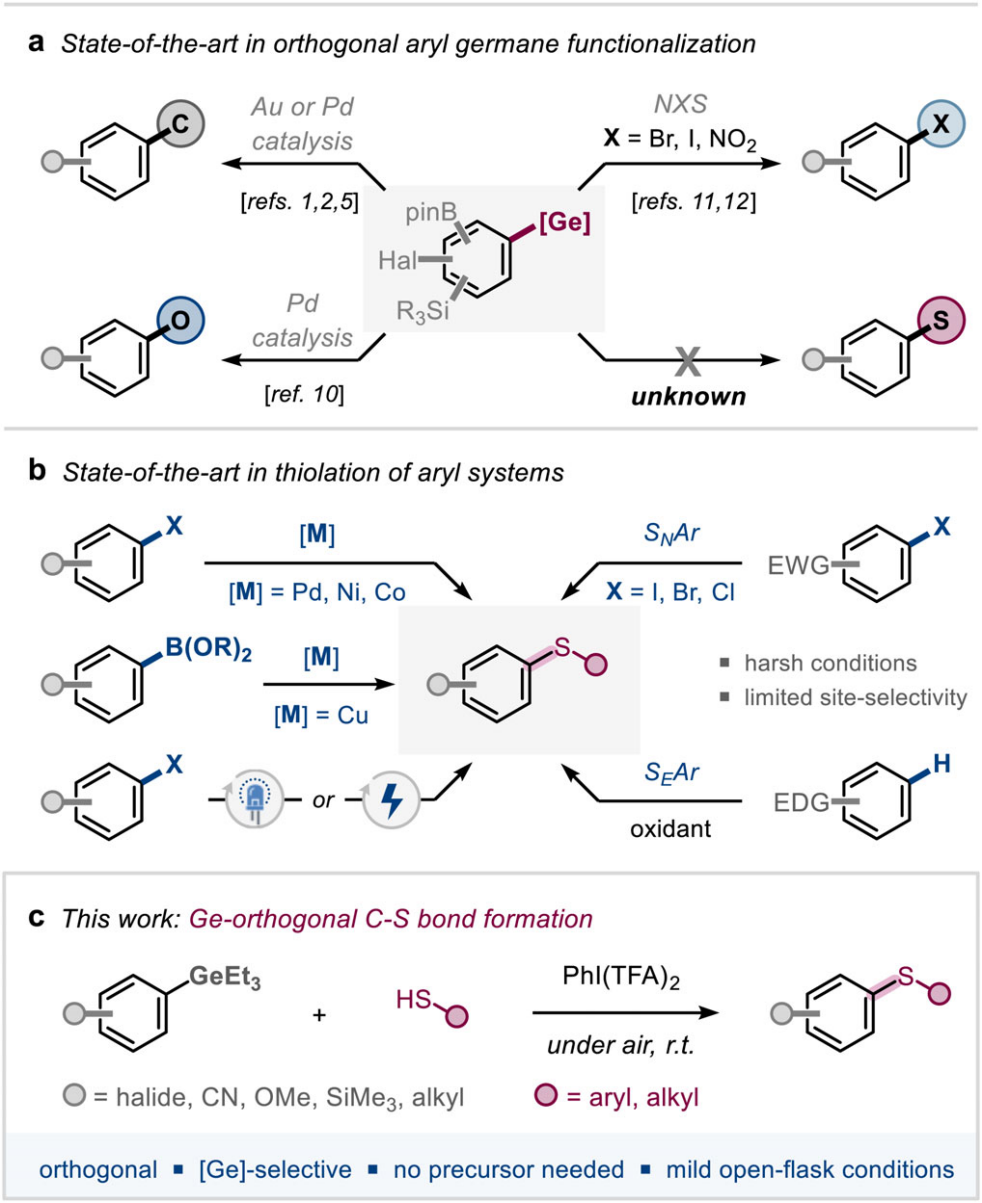
(a) state‐of‐the‐art in orthogonal aryl germane functionalization; (b) state‐of‐the‐art in thiolation of aryl systems, and (c) this work: Ge‐selective, C─S bond formation.

The formation of aryl─S bonds is of broad significance in medicinal chemistry, agrochemistry and materials science. Thioethers are common in bioactive molecules, including therapeutics for neurodegenerative diseases, viral infections and cancer [19, 20, 21, 22, 23], and they are important building blocks in functional materials and polymer chemistry [24, 25, 26, 27]. Aryl sulfides also serve as key precursors to sulfoxides, sulfones, sulfilimines, and sulfoximines [28, 29, 30]. Their synthesis relies primarily on metal‐catalyzed cross‐coupling reactions of aryl halides [31, 32, 33], most commonly under palladium catalysis [34, 35, 36, 37], although nickel [38, 39, 40, 41, 42, 43, 44], copper [45, 46, 47, 48, 49, 50, 51, 52], and cobalt [53, 54, 55, 56] systems are also effective (Figure 1b). Similar metal‐catalyzed approaches have been extended to activated pseudohalide analogues, such as diazonium salts [57, 58, 59]. Site‐selective Pd‐mediated transformations of poly(pseudo)halogenated arenes have also been demonstrated [60]. Photocatalytic [61, 62] and electrochemical methods [63, 64, 65, 66, 67] have been developed as well.

Metal‐free radical or ionic substitution pathways [68, 69, 70, 71, 72], including light‐promoted reactions [73, 74, 75], have emerged as complementary strategies [76, 77]. However, S_N_Ar methods are generally restricted to electron‐deficient arenes [78, 79], while S_E_Ar and radical approaches often target C─H sites directly [80, 81, 82, 83, 84], raising challenges of site‐selectivity or requiring previous steps for the preparation of specialized thiol precursors [85, 86, 87, 88].

In view of the central role of aryl‐S linkages and the distinct orthogonality of aryl germanes, a method for the metal‐free thiolation of aryl germanes would substantially expand the synthetic repertoire available for modular and predictable molecule construction. Such a transformation would provide a new and highly selective functional handle that is compatible with existing aryl electrophiles and could enable enhanced levels of control in multi‐step arene functionalization.

Building on our group's prior demonstration of the feasibility of radical substitution at an aryl germane [18], we questioned whether an analogous process could be extended to thiolation. Such a strategy would enable site‐selective functionalization at the *ipso*‐carbon of the aryl germane, in contrast to conventional C–H electrophilic thiolations, which typically yield product mixtures and require electron‐rich arenes as substrates. Moreover, we anticipated that this approach would be compatible with other common synthetic handles such as halides, silanes, or boron derivatives, which are not generally prone to radical *ipso*‐substitution [74].

We therefore sought reaction conditions that would allow direct generation of thiyl radicals [91] from commercially available thiols using PIFA as a formal oxidant [92, 93, 94], without relying on specialized precursors, which would promote selective reaction with the aryl germane.

## 2 Results and Discussion

### 2.1 Reaction Optimization and Mechanistic Studies

Through systematic optimization, we found that employing 2.0 equivalents each of the thiol and PIFA in MeCN under non‐inert conditions furnished the desired thiolation product in 96% yield (based on ^19^F‐NMR analysis) (Figure 2a; see Supporting Information for optimization details). Comparable yields were obtained in halogenated solvents such as DCE and HFIP; however, MeCN was selected due to its lower toxicity. In contrast, reactions conducted in toluene or THF afforded little to no product (<15%). We also observed a pronounced concentration effect: decreasing the reaction concentration to 0.25 M resulted in an approximately 33% reduction in yield.

**FIGURE 2:**
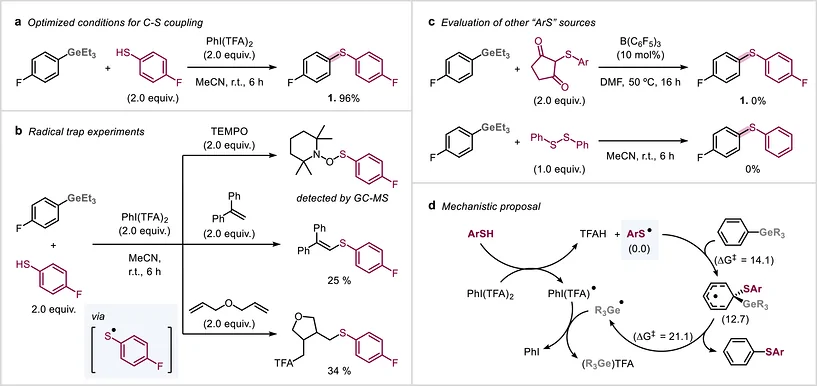
(a) Optimized reaction conditions. (b) Evaluation of other thiolation reagents. c) Reaction outcome in the presence of radical traps. d) Mechanistic proposal. Computed Gibbs free energies (in kcal/mol) [89, 90] for R = Me are shown in brackets. For details, see the Supporting Information. TFA = trifluoroacetate.

To gain insight into the mechanism of this transformation, we conducted a series of mechanistic experiments (Figure 2b). The addition of TEMPO completely inhibited product formation. However, because TEMPO is known to react directly with PIFA [95, 96, 97], we examined additional radical quenchers. Introduction of diphenylethylene into the reaction mixture resulted in formation of the corresponding thiyl radical‐trapping adduct, which was isolated in 25% yield, with no formation of the thioether product. Furthermore, when diallyl ether was used as a scavenger, the expected radical cyclization product bearing a trifluoroacetate group was also isolated (34% yield). These results support the generation of a thiyl radical under the reaction conditions.

In addition, substitution of the thiol/PIFA system with a nonradical but electrophilic sulfur source employed in C–H thiolation, 4‐(fluorophenyl)thiosuccinimide, failed to produce any product, even in the presence of B(C_6_F_5_)_3_ as an activator (Figure 2c) [81]. A similar outcome was observed when phenyl disulfide was employed as thiolation reagent.

We next performed DFT calculations [89, 90] employing PhGeMe_3_ and PhSH as model reagents (see Supporting Information for further details). Addition of the thiyl radical, generated by reaction between PIFA and the thiol, to the *ipso*‐carbon of the aryl germane takes place with an activation barrier of 14.1 kcal/mol, leading to the radical σ‐complex intermediate. The dissociation of the GeMe_3_ radical from this intermediate, with a higher associated activation barrier (Δ*G* ^‡^ = 21.1 kcal/mol), renders the thioether product (Figure 2d). Moreover, GC‐MS and ^19^F NMR analyses of the crude reaction mixture indicated the formation of (R_3_Ge)TFA.

### 2.2 Scope of the Reaction and Orthogonality

With a plausible mechanism and optimized conditions in hand, we next examined the reaction scope, with particular attention to its orthogonality to other commonly employed functional groups (Scheme 1). We first assessed intermolecular selectivity through competition experiments between the aryl germane and aryl silane or aryl pinacol boronic acid derivatives, each bearing a *para‐*electron‐withdrawing substituent as an activating group (Scheme 1a). Under these conditions, the protocol proved fully selective for aryl germane functionalization [98].

**SCHEME 1:**
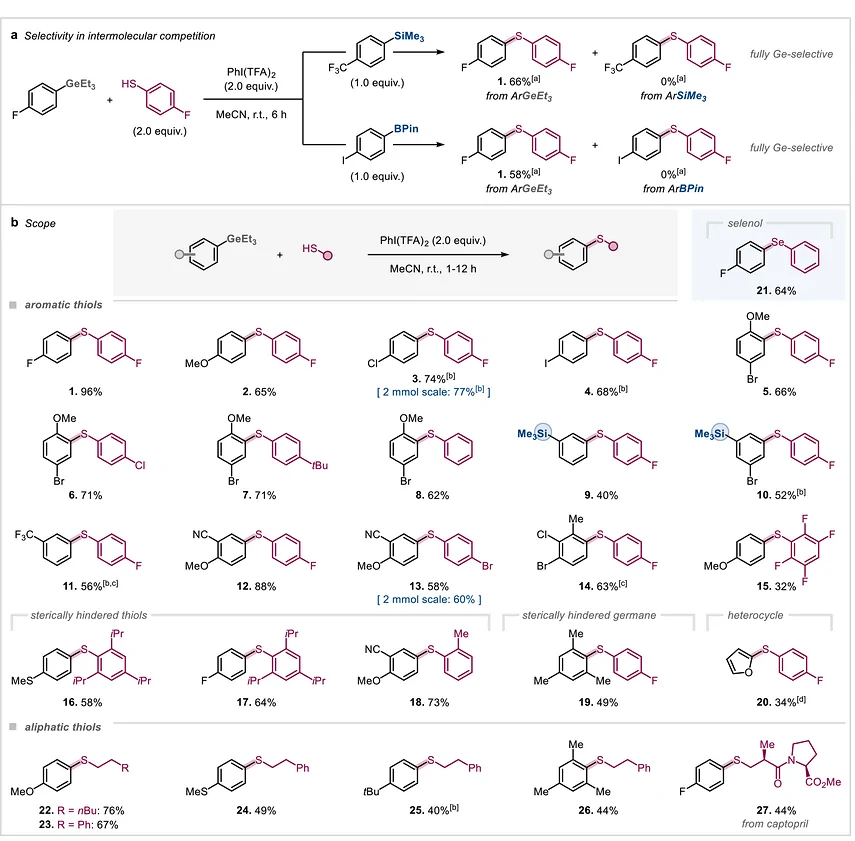
(a) Selective GeEt_3_‐thiolation of arenes that carry common coupling handles. (b) Scope of aryl germane thiolation. *Reaction conditions*: aryl germane (1.0 equiv.), thiol (2.0 equiv), PhI(TFA)_2_ (2.0 equiv.) in MeCN (1.0 M). ^[a]^ Quantitative ^19^F NMR yield. ^[b]^ Neat conditions. ^[c]^ With additional thiol (2.0 equiv.) and PhI(TFA)_2_ (2.0 equiv.) added after 2 h. ^[d]^ With DBU (2.0 equiv.) added. TFA = trifluoroacetate.

We then evaluated the intramolecular selectivity of aryl germanes over different synthetic handles, as it would be the entry to highly functionalized arenes (Scheme 1b). The reaction exhibited clear germanium selectivity over all halides, including chloride (**3**), bromide (**5**–**8**), and iodide (**4**), which are widely used in metal‐catalyzed thiolations. Halide tolerance was evident not only on the aryl germane but also on the thiol component, as both chloride‐ (**6**) and bromide‐containing (**13**) thiols were successfully engaged. This behavior contrasts with metal‐catalyzed cross‐coupling methods, which typically functionalize the halide and for which site‐selectivity can be challenging to achieve [60, 99]. Furthermore, aryl germane systems bearing trimethylsilyl substituents underwent selective *ipso‐*thiolation at the germane position (**9**, **10**), leaving the –SiMe_3_ group intact and thereby enabling subsequent post‐functionalization (vide infra).

Electron‐rich substrates, such as the *para*‐methoxy derivative (**2**) and the *ortho*‐methoxy series (**5**–**8**), were efficiently thiolated [100]. Likewise, aryl germanes bearing electron‐withdrawing groups, including cyano and trifluoromethyl substituents, as well as polyhalogenated substrates, underwent thiolation smoothly to afford the corresponding thioethers in good yields (**11**–**15**). These results indicate that the method is largely insensitive to the electronic properties of the aryl germane and does not require electronically activated substrates, in clear contrast to previously reported aromatic substitution methodologies [78, 79, 101, 102]. In some cases, we observed that full conversion of the aryl germane was not reached even after prolonged reaction times due to competitive disulfide formation. Hence, the overall outcome of the reaction is a balance between the rates of thiyl radical addition to the *ipso* carbon of the aryl germane substrate and its recombination toward disulfide generation. In these cases, conversion can be further improved simply by feeding the system with additional thiol and PIFA reagents. This approach was demonstrated for substrates **11** and **14**, for which the yields increased by 16% and 10%, respectively, following this strategy (see Supporting Information for further information). We also detected that electron‐rich substrates, such as *p*‐anisolegermane (**2**, **15**), suffered from competitive protodegermylation by reaction with the in situ generated trifluoroacetic acid. The degermylated byproduct was formed in substantial amounts (20%–50%), preventing higher yields for the thiolation reaction. However, importantly, there was only ever Ge‐thiolation in the site‐selectivity studies and no other competing C─S bond formation was seen.

We then evaluated steric effects, as thiolation involving bulky substrates is underexplored, and found them to be minimal with our protocol: *ortho*‐substituted aryl germanes, whether bearing methoxy groups (**5**–**8**), polyhalogenated *ortho*‐methyl substitution (**14**), or even a mesityl germane framework (**20**), all reacted effectively. Similarly, *ortho*‐substituted 2‐methylbenzenethiol participated well (**18**), and substrates containing two bulky isopropyl groups still afforded the desired products in good yields (**16**, **17**). Use of a heteroaryl germane provided the corresponding thiolated product (**19**), albeit in moderate yield, due to competitive protodegermylation.

The method extended beyond aromatic thiols: aliphatic thiols also performed efficiently (**22**–**27**), and it was also effective at larger scale (see entries **3** and **13** in Scheme 1). Moreover, the corresponding selenoether (**21**) was obtained from the analogous selenol in good yield without additional optimization, underscoring the versatility of this approach for selective C─S and C─Se bond formation.

However, functional groups that readily react with PIFA, such as phenols and amines, as well as oxidation‐prone and acid‐sensitive heteroaryl germanes, were not compatible with the reaction conditions and did not afford the corresponding aryl sulfides (see the Supporting Information for an overview of the reaction limitations).

We next investigated the utility of aryl germanes in orthogonal thiolation strategies and modular synthesis. With our protocol, the germane moiety can be selectively functionalized in the presence of an iodine substituent to furnish product **4**, or, alternatively, the C─I bond can be functionalized while leaving the germane group untouched (Scheme 2a). For example, using Pd^(I)^‐dimer–enabled thiolation [60], we selectively functionalized the aryl iodide to form the corresponding C─S bond in 72% yield, with the germane group remaining fully intact. Applying other C─S bond formation protocols, such as Ni‐catalysis [44], Ullman coupling [51], or metal‐free photoredox conditions via an EDA complex [74], also led to the selective C─I bond functionalization. In all cases, the remainder of the mass balance was unreacted starting material; the [Ge] handle remained fully intact to the reaction conditions. The exceptional degree of aryl germanes’ orthogonality in thiolation is superior to that of boronic acid derivatives, where only Chan–Lam coupling would allow for it [49], or that of aryl silanes, for which no direct thiolation method has been reported. To further demonstrate the versatility of this approach, we examined a C(sp^2^) building block containing three distinct functional groups (─Br, ─SiMe_3_, and ─GeEt_3_; **31**). We first achieved selective functionalization at the C–GeEt_3_ position without altering the other handles (**10**). Subsequent Pd^(I)^‐catalyzed alkylation at the bromide site proceeded smoothly to afford the desired product in 95% yield (**32**) [103]. Finally, treatment with NIS enabled conversion of the silane into the corresponding iodide (**33**), thereby completing the modular functionalization sequence (Scheme 2b).

**SCHEME 2:**
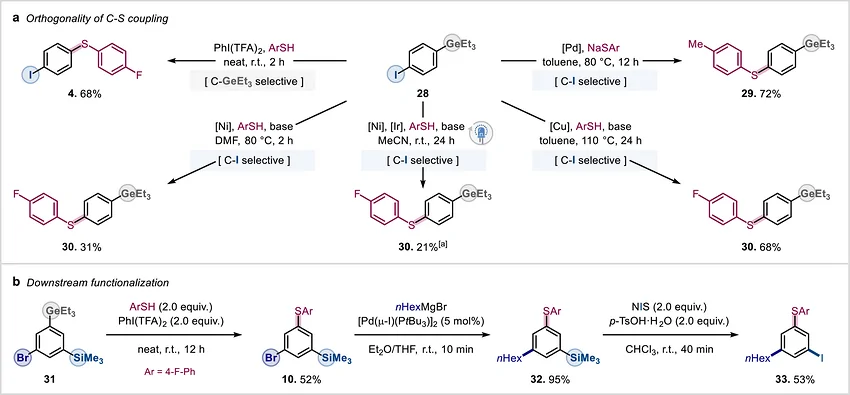
(a) Orthogonality of GeEt_3_‐selective thiolation of arenes. (b) Product postfunctionalization. ^[a]^ Based on quantitative ^19^F NMR yield.

## 3 Conclusion

In conclusion, a highly modular and selective approach for the thiolation of aryl germanes has been developed, enabling efficient C─S bond formation under mild, open‐flask and metal‐free conditions. The method is compatible with several synthetically useful functional groups, including C─Si, C─B, and C─halide functionalities, providing opportunities for orthogonal and sequential functionalization. With C─S bond formation now unlocked, this study further positions organogermanes as versatile and orthogonal building blocks, with broad potential to advance selective synthesis and expand the toolbox of synthetic organic chemistry.

## Author Contributions

**Markus D. Schoetz**: investigation. **Susanna L. Kirsten**: investigation. **Franziska Schoenebeck**: writing – original draft, supervision, resources. **Kristina Deckers**: investigation. **Steffen Benkhoff**: investigation. **Amit Dahiya**: investigation. **Jaime Ponce‐de‐León**: investigation.

## Conflicts of Interest

The authors declare no conflicts of interest.

## Supporting information




**Supporting File**: anie74456‐sup‐0001‐SuppMat.pdf

## Data Availability

The data that support the findings of this study are available in the Supporting Information of this article.
