# Supplementary material for: C─S Bond Formation of Aryl Germanes
Source: Angew Chem Int Ed Engl. 2026 Aug 26;65(41):e3776319. doi: 10.1002/anie.3776319 (PMC13638098; doi:10.1002/anie.3776319)
Supplement: Supplementary file 1 [file ANIE-65-e3776319-s001.pdf]

Contents

1. General Experimental Details S2

2. Optimization of Reaction Conditions S4

2.1 Screening of amount of thiol and PIFA reagent S4

2.2 Screening of concentration S5

2.3 Solvent screening S6

3. C-S Bond Formation with Aryl Germanes S7

3.1 General Procedure S7

3.2 Characterization Data of the Products S7

4. Orthogonal Functionalization S17

4.1 Pd(I)-I-dimer catalyzed C-S bond formation S17

4.2 Cu catalyzed C-S bond formation S17

4.3 Ni catalyzed C-S bond formation S18

4.4 Light promoted C-S bond formation S18

4.5 Pd(I)-I-dimer catalyzed Kumada Cross-coupling S19

4.6 Iodination of (3-((4-fluorophenyl)thio)-5-hexylphenyl)trimethylsilane S20

5. Mechanistic Studies S21

5.1 TEMPO experiment S21

5.2 1,1-diphenylethylene experiment S21

5.3 Diallylether experiment S22

5.4 Arylthiosuccinimide as sulfur source S22

5.5 Disulfide as sulfur source S23

5.6 Intermolecular competition experiments S23

5.7 Limitations of scope S24

6. Computational Analysis S25

6.1 General details S25

6.2 Method comparison S25

6.3 XYZ coordinates of computed structures and energies S25

7. NMR Spectra S29

8. References S73

1. General Experimental Details

Techniques

All reactions were performed utilizing standard Schlenk techniques under an Argon atmosphere or inside a glovebox unless otherwise stated. Glassware and magnetic stir bars were dried in an oven (130 °C) for at least 24 hours prior to use. Unless otherwise stated, experiments were carried out at room temperature (25 ± 2 °C). The removal of solvents in vacuo was achieved using a rotary evaporator (bath temperatures up to 40 °C) at a pressure of 20 mmHg (diaphragm pump), or at 0.1 mmHg (oil pump) on a vacuum line at room temperature.

Solvents, reagents and starting materials

Unless otherwise stated, all anhydrous solvents were purchased from Sigma Aldrich. DCM, THF, and toluene were dried using an MBraun 800 or an Innovative Technology PS-MD-5 solvent purification system. Technical grade solvents were distilled prior to use for chromatography and extraction.

Unless otherwise stated, all reagents and starting materials were commercially available and used as received.

The following compounds were synthesized according to literature procedures: triethyl(4-methoxyphenyl)germane [1], (5-bromo-2-methoxyphenyl)triethylgermane [2], 2-methoxy-5-(triethylgermyl)benzo-nitrile [3], (3-bromo-5-(triethylgermyl)phenyl)trimethylsilane [3], triethyl(4-iodophenyl)germane [4], trimethyl(3-(triethylgermyl)phenyl)silane [5], (4-bromo-3-chloro-2-methylphenyl)triethylgermane [6], (4-chlorophenyl)triethylgermane [6], triethyl(3-(trifluoromethyl)phenyl)germane [7], triethylmesitylgermane [1], methyl ((*S*)-3-mercapto-2-methylpropanoyl)-L-prolinate (*captopril* methyl ester) [8].

Purification

Thin layer chromatography (TLC) was performed on Macherey Nagel ALUGRAM Xtra SIL G UV254 aluminium plates with unmodified silica and visualized either under UV light or stained with iodine, $KMnO_4$ or PMA. Flash silica gel column chromatography was performed with silica gel (0.04 – 0.063 mm particle size) purchased from Macherey Nagel. Preparative HPLC was performed on a Knauer Azura HPLC (employing UV detector 2600, at 254 and 230 nm) using a LiChrosorb Si60 column (Merck, 250 x 25 mm, 7 µm silica poriosity).

Characterization

All ^{1}H, ^{13}C and ^{19}F NMR spectra were recorded on Bruker Avance Neo 600, Varian VNMRS 600 or Varian VNMRS 400 or Bruker Avance Neo 400 spectrometers at ambient temperature (unless otherwise specified). Chemical shifts (δ) are reported in parts per million (ppm) and were referenced either to residual solvent peak (for ^{1}H and ^{13}C spectra) or internally by the instrument after locking and shimming to the deuterated solvent (for ^{19}F). Coupling constants (*J*) are given in Hertz (Hz). Multiplicities of signals in ^{1}H, ^{19}F, and ^{13}C NMR were designated as s (singlet), d (doublet), dd (doublet of doublets), dt (doublet

of triplets), ddd (doublet of doublets of doublets), t (triplet), td (triplet of doublets), q (quartet), p (quintet), h (sextet), hept (septet), and m (multiplet).

Gas chromatography coupled with mass spectrometry (GC-MS) was performed on an Agilent Technologies 5975 series MSD mass spectrometer under electrospray ionization (EI) mode coupled with an Agilent Technologies 7820A gas chromatograph employing an Agilent 19091s-433 HP-5MS column (30 m x 0.250 µm x 0.250 µm). High-resolution mass spectrometry (HRMS) was performed using a Thermo Scientific LTQ Orbitrap XL spectrometer (ESI), Finnigan MAT 95 (EI) or Bruker Maxis II LC-MS-System (APCI). IR spectra were recorded on a Spectrum 100 spectrometer with a UATR Diamond/KRS-5 crystal with attenuated total reflectance (ATR).

Photochemistry setup

Photochemical reactions were carried out in an EvoluChem™ PhotoRedOxBox (see photo below) equipped with one EvoluChem 450PF LED lamp (λ_{max} = 450 nm, 30 W)

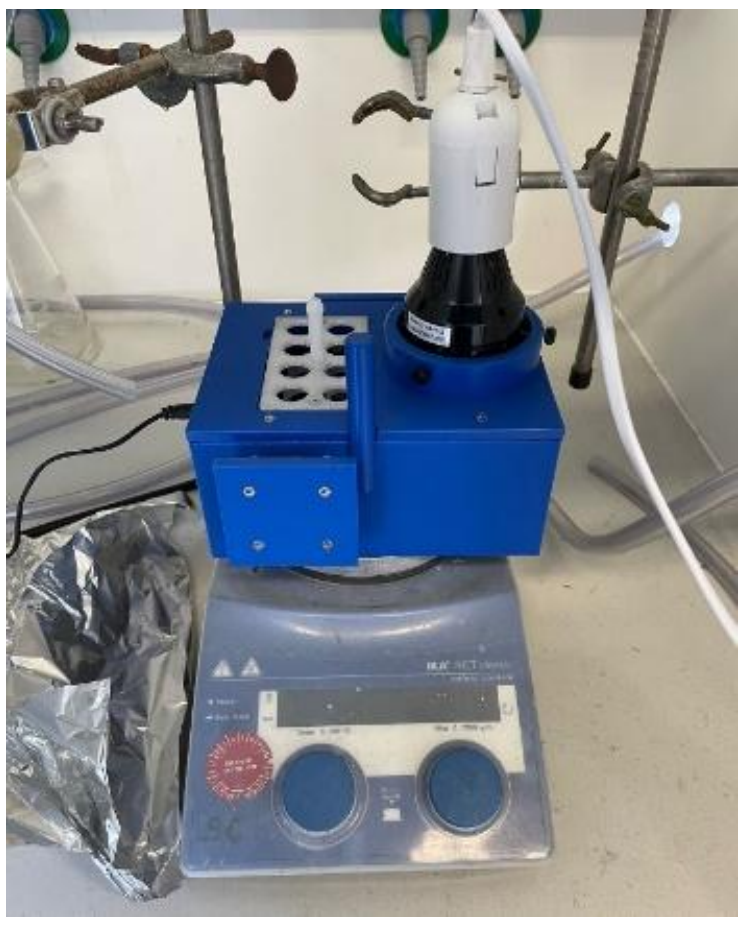

2. Optimization of Reaction Conditions

2.1 Screening of amount of thiol and PIFA reagent

Under air, a 4 mL screw-capped glass vial equipped with a magnetic stir bar was charged with with triethyl(4-fluorophenyl)germane (25.4 mg, 0.1 mmol, 1.0 equiv.), 4-fluorobenzenethiol and dissolved in MeCN (0.25 M). $PhI(TFA)_2$ was added to the solution. The reaction mixture was stirred for 12 h at room temperature. Then, the reaction mixture was analyzed by quantitative ^{19}F NMR using 1,4-difluorobenzene as internal standard.

Table S2. Screening of amount of thiol and PIFA reagent.

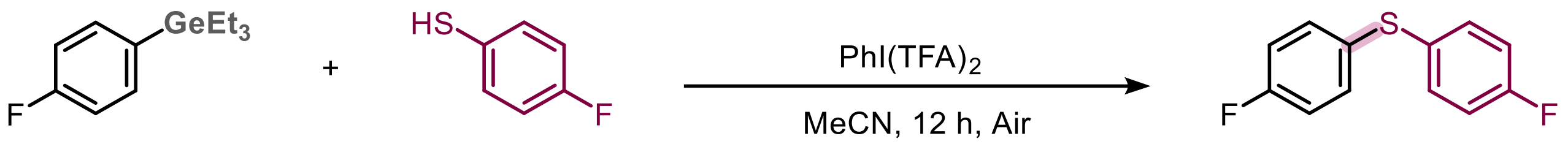

Entry	PIFA (equiv.)	Thiol (equiv.)	Yield (%) [a]
1	1.2	1.2	19
2	1.2	1.5	18
3	1.2	2.0	21
4	1.5	1.2	31
5	1.5	1.5	41
6	1.5	2.0	37
7	2.0	1.2	8
8	2.0	1.5	48
9	2.0	2.0	67
10	2.0	2.5	61
11	2.0	3.0	62

[a] Yield was determined by quantitative ^{19}F NMR using 1,4-difluorobenzene as internal standard.

2.2 Screening of concentration

Under air, a 4 mL screw-capped glass vial equipped with a magnetic stir bar was charged with triethyl(4-fluorophenyl)germane (25.4 mg, 0.1 mmol, 1.0 equiv.), 4-fluorobenzenethiol (21.3 μL,0.2 mmol, 2.0 equiv.) and dissolved in MeCN. PhI(TFA)$_2$ (86.0 mg, 0.2 mmol, 2.0 equiv.) was added to the solution. The reaction mixture was stirred for 12 h at room temperature. Then, the reaction mixture was analyzed by quantitative ^{19}F NMR using 1,4-difluorobenzene as internal standard.

Table S3. Screening of concentration.

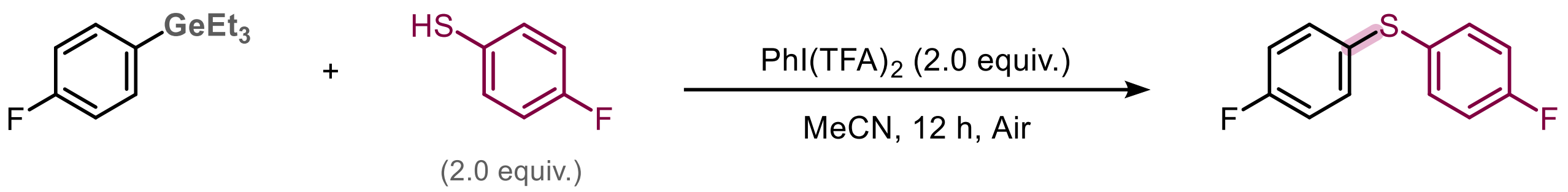

Entry	Concentration (mol/L)	Yield (%) [a]
1	1.0	96
2	0.5	76
3	0.25	67
4	0.20	38
5	0.17	46

[a] Yield was determined by quantitative ^{19}F NMR using 1,4-difluorobenzene as internal standard.

2.3 Solvent screening

Under air, a 4 mL screw-capped glass vial equipped with a magnetic stir bar was charged with triethyl(4-fluorophenyl)germane (25.4 mg, 0.1 mmol, 1.0 equiv.), 4-fluorobenzenethiol (21.3 μL,0.2 mmol, 2.0 equiv.) and dissolved in the corresponding solvent (1 M, 0.1 mL). PhI(TFA)$_2$ (86.0 mg, 0.2 mmol, 2.0 equiv.) was added to the solution. The reaction mixture was stirred for 12 h at room temperature. Then, the reaction mixture was analyzed by quantitative ^{19}F NMR using 1,4-difluorobenzene as internal standard.

Table S1. Solvent screening.

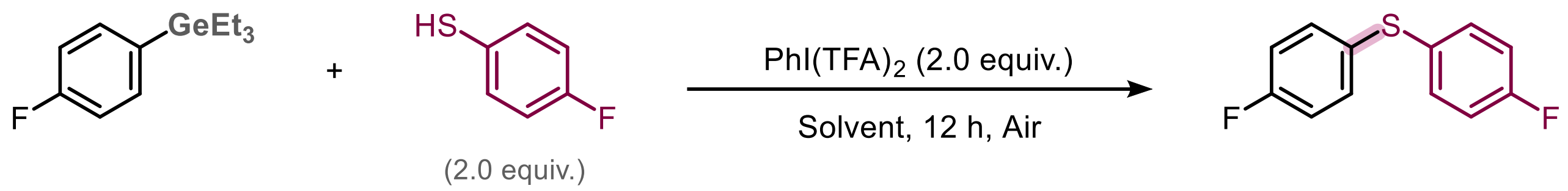

Entry	Solvent	Yield (%) [a]
1	MeCN	96
2	toluene	14
3	DCE	94
4	THF	0
5	HFIP	95

[a] Yield was determined by quantitative ^{19}F NMR using 1,4-difluorobenzene as internal standard.

3. C-S Bond Formation with Aryl Germanes

3.1 General Procedure

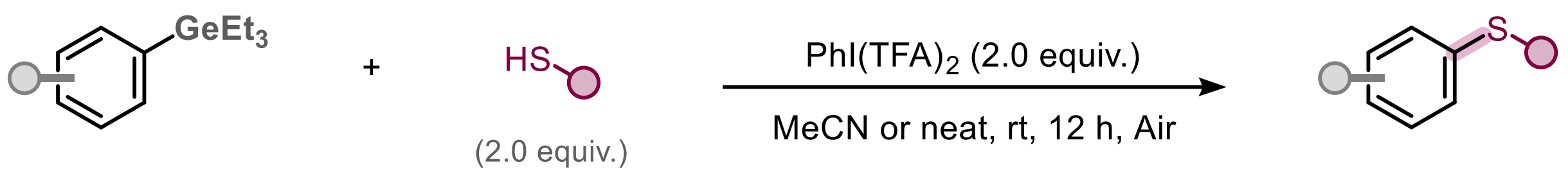

Under air, a 4 mL screw-capped glass vial equipped with a magnetic stir bar was charged with the aryl germane (1.0 equiv.), thiol (2.0 equiv.) and dissolved in MeCN (1.0 M). $PhI(TFA)_2$ (2.0 equiv.) was added to the solution. The reaction solution was stirred for 1 h – 12 h at room temperature. After the reaction was complete (as monitored by GC-MS or TLC) the mixture was filtered through a plug of silica rinsing with diethyl ether. The resulting solution was concentrated under reduced pressure and the obtained crude residue was purified by preparative TLC or flash chromatography on silica gel.

3.2 Characterization Data of the Products

Bis(4-fluorophenyl)sulfane (1)

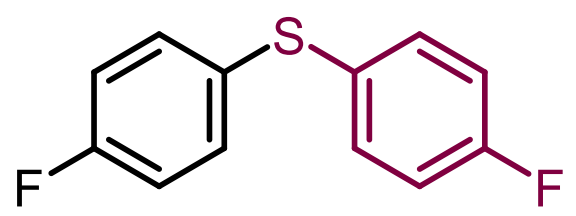

Prepared, following the general procedure from triethyl(4-fluorophenyl)germane (76.5 mg, 0.3 mmol, 1.0 equiv.) and 4-fluorobenzenethiol (64.0 μL, 0.6 mmol, 2.0 equiv.). The title product was obtained after purification by column chromatography (*n*-pentane) as a colorless liquid (64.0 mg, 0.288 mmol, 96%).

R_f = 0.47 (*n*-pentane). **^{1}H NMR** (600 MHz, CD_2Cl_2) δ 7.36 – 7.31 (m, 4H), 7.06 – 7.01 (m, 4H). **^{13}C{^{1}H} NMR** (151 MHz, CD_2Cl_2) δ 162.7 (d, *J* = 246.7 Hz), 133.5 (d, *J* = 8.0 Hz), 131.7 (d, *J* = 3.1 Hz), 116.7 (d, *J* = 22.1 Hz). **^{19}F NMR** (564 MHz, CD_2Cl_2) δ -115.19 (tt, *J* = 8.7, 5.3 Hz, 2F). **IR** (neat, cm^{-1}): 3068, 2925, 2854, 2328, 2111, 1886, 1758, 1639, 1587, 1485, 1396, 1291, 1224, 1154, 1085, 1012, 935, 821. **HRMS** (APCI) calculated for $C_{12}H_8F_2S$: 222.0309 [M]$^+$, found: 222.0304.

(4-Fluorophenyl)(4-methoxyphenyl)sulfane (2)

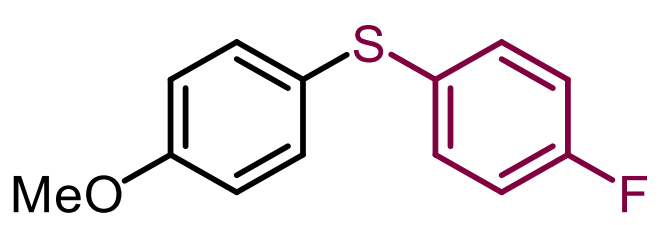

Prepared, following the general procedure from triethyl(4-methoxyphenyl)germane (80.1 mg, 0.3 mmol, 1.0 equiv.) and 4-fluorobenzenethiol (64.0 μL, 0.60 mmol, 2.0 equiv.). The title product was obtained after purification by preparative TLC (*n*-pentane/EtOAc 40:1) as a yellow liquid (45.3 mg, 0.19 mmol, 64%).

R_f = 0.45 (*n*-pentane/Et_2O 40:1). **^{1}H NMR** (600 MHz, $CDCl_3$) δ 7.37 – 7.34 (m, 2H), 7.21 – 7.19 (m, 2H), 6.99 – 6.94 (m, 2H), 6.89 – 6.87 (m, 2H), 3.81 (s, 3H). **^{13}C{^{1}H} NMR** (151 MHz, $CDCl_3$) δ 161.8 (d, *J* = 245.9 Hz), 159.8, 134.6, 133.3 (d, *J* = 3.2 Hz), 131.2 (d, *J* = 8.0 Hz), 125.4, 116.2 (d, *J* = 22.3 Hz), 115.1, 55.5. **^{19}F NMR** (564 MHz, $CDCl_3$) δ -116.19 (tt, *J* = 8.5, 5.0 Hz, 1F). **IR** (neat, cm^{-1}): 2937, 2323, 2107, 1883, 1588, 1486, 1288, 1242, 1029, 821. **HRMS** (ESI) calculated for $C_{13}H_{11}OFS$: 234.0509 [M]$^+$, found: 234.0506. These data are in agreement with those reported previously in the literature [9].

(4-Chlorophenyl)(4-fluorophenyl)sulfane (3)

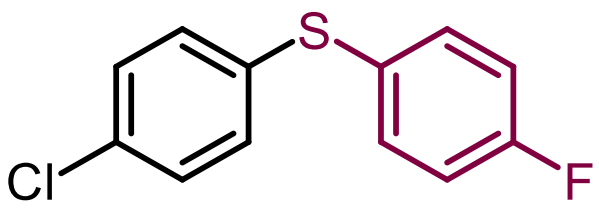

Prepared, following the general procedure from (4-chlorophenyl)triethylgermane (81.4 mg, 0.3 mmol, 1.0 equiv.) and 4-fluorobenzenethiol (64.0 µL, 0.60 mmol, 2.0 equiv.) under neat conditions. The title product was obtained after purification by preparative HPLC (*n*-hexane, flow rate 20 mL/min) as a colourless liquid (52.7 mg, 0.221 mmol, 74%).

Note: Performing the reaction on a scale of 2.0 mmol with a reaction time of 17 h, the title product was obtained after purification by column chromatography (pentane) as a colourless liquid (221.6 mg, 0.928 mmol, 46% product; and 321.0 mg of mixed fraction containing 47.5% product and 52.5% disulfide byproduct; combined yield of product: 369.0 mg, 1.546 mmol, 77%).

Caution: When performing the reaction under neat conditions the reaction mixture becomes very hot upon addition of PIFA, especially on larger scale. Cooling with an ice bath should be considered.

R_f = 0.69 (*n*-pentane). **^{1}H NMR** (600 MHz, CD_2Cl_2) δ 7.42 – 7.37 (m, 2H), 7.26 (d, *J* = 8.6 Hz, 2H), 7.19 (d, *J* = 8.6 Hz, 2H), 7.09 – 7.03 (m, 2H). **^{13}C{^{1}H} NMR** (151 MHz, CD_2Cl_2) δ 163.0 (d, *J* = 247.8 Hz), 136.0, 134.9 (d, *J* = 8.6 Hz), 133.0, 131.4, 130.0 (d, *J* = 3.1 Hz), 129.6, 116.9 (d, *J* = 22.1 Hz). **^{19}F NMR** (564 MHz, CD_2Cl_2) δ -114.17 (tt, *J* = 8.7, 5.3 Hz, 1F). **IR** (neat, cm^{-1}): 1587, 1482, 1391, 1225, 1089, 1010, 817. **HRMS** (ESI) calculated for $C_{12}H_8ClFS$: 238.0014 $[M]^+$, found: 238.0010. These data are in agreement with those reported previously in the literature [10].

(4-Fluorophenyl)(4-iodophenyl)sulfane (4)

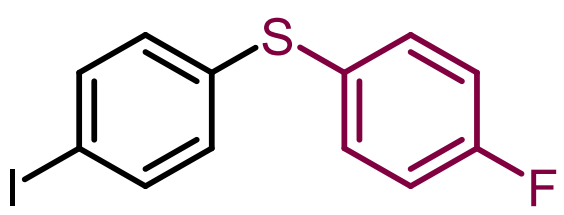

Prepared following the general procedure from triethyl(4-iodophenyl)germane (36.3 mg, 0.1 mmol, 1.0 equiv.) and 4-fluorobenzenethiol (21.3 µL, 0.20 mmol, 2.0 equiv.) under neat conditions. The title product was obtained after purification by column chromatography (*n*-pentane) as a yellow oil (22.3 mg, 0.068 mmol, 68%).

Caution: When performing the reaction under neat conditions the reaction mixture becomes very hot upon addition of PIFA, especially on larger scale. Cooling with an ice bath should be considered.

R_f = 0.67 (*n*-pentane). **^{1}H NMR** (600 MHz, $CDCl_3$) δ 7.57 (d, *J* = 8.5 Hz, 2H), 7.41 – 7.38 (m, 2H), 7.05 (dd, *J* = 8.6, 8.6 Hz, 2H), 6.96 – 6.94 (m, 2H). **^{13}C{^{1}H} NMR** (151 MHz, $CDCl_3$) δ 162.8 (d, *J* = 248.9 Hz), 138.2, 137.4, 134.9 (d, *J* = 8.4 Hz), 131.2, 129.2 (d, *J* = 3.5 Hz,), 116.8 (d, *J* = 21.8 Hz), 91.6. **^{19}F NMR** (564 MHz, $CDCl_3$) δ -112.96 (tt, *J* = 9.0, 5.1 Hz, 1F). **IR** (neat, cm^{-1}): 2924, 1586, 1484, 1223, 1080, 998, 800. **HRMS** (ESI) calculated for $C_{12}H_8FIS$: 329.9375 $[M]^+$, found: 329.9360.

(5-Bromo-2-methoxyphenyl)(4-fluorophenyl)sulfane (5)

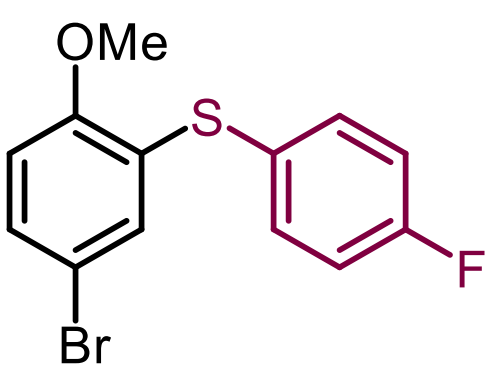

Prepared, following the general procedure from (5-bromo-2-methoxyphenyl)triethylgermane (44.5 mg, 0.13 mmol, 1.0 equiv.) and 4-fluorobenzenethiol (27.7 µL, 0.26 mmol, 2.0 equiv.). The title product was

obtained after purification by column chromatography (n-pentane / Et_2O 40:1) as a colorless solid (26.5 mg, 0.084 mmol, 66%).

$\boldsymbol{R_f}$ = 0.10 (n-pentane /Et_2O 40:1). **M.p.** = 126.4 °C. **^{1}H NMR** (600 MHz, $CDCl_3$) δ 7.45 – 7.42 (m, 2H), 7.26 (dd, J = 8.7, 2.4 Hz, 1H), 7.08 (dd, J = 8.6, 8.6 Hz, 2H), 6.92 (d, J = 2.4 Hz, 1H), 6.73 (d, J = 8.7 Hz, 1H), 3.86 (s, 3H). **^{13}C{^{1}H} NMR** (151 MHz, $CDCl_3$) δ 163.1 (d, J = 249.2 Hz), 155.4, 135.8 (d, J = 8.3 Hz), 131.3, 130.1, 128.6, 127.4 (d, J = 3.5 Hz), 116.9 (d, J = 22.0 Hz), 113.5, 112.2, 56.3. **^{19}F NMR** (564 MHz, $CDCl_3$) δ -112.47 (tt, J = 8.8, 5.1 Hz, 1F). **IR** (neat, cm^{-1}): 2933, 1585, 1470, 1231, 1064, 1020, 828. **HRMS** (ESI) calculated for $C_{13}H_{10}O^{79}BrFS$: 311.9620 $[M]^+$, found: 311.9614.

(5-Bromo-2-methoxyphenyl)(4-chlorophenyl)sulfane (6)

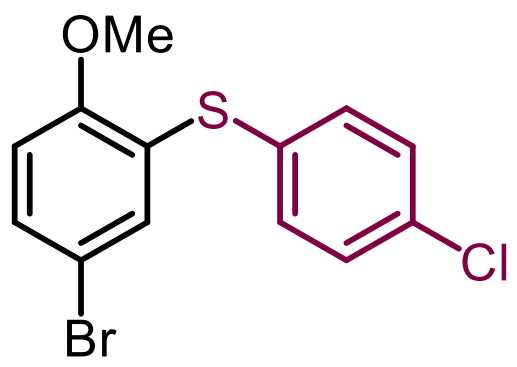

Prepared, following the general procedure from (5-bromo-2-methoxyphenyl)triethylgermane (103.8 mg, 0.3 mmol, 1.0 equiv.) and 4-chlorobenzenethiol (86.8 mg, 0.60 mmol, 2.0 equiv.). The title product was obtained after purification by preparative TLC (n-pentane/EtOAc 20:1) as a yellowish liquid (70.2 mg, 0.21 mmol, 71%).

$\boldsymbol{R_f}$ = 0.38 (n-pentane/Et_2O 40:1). **^{1}H NMR** (600 MHz, $CDCl_3$) δ 7.33 – 7.29 (m, 5H), 7.10 (d, J = 2.4 Hz, 1H), 6.76 (d, J = 8.7 Hz, 1H), 3.85 (s, 3H). **^{13}C{^{1}H} NMR** (151 MHz, $CDCl_3$) δ 156.3, 134.1, 133.6, 133.1, 131.9, 131.1, 129.8, 126.8, 113.4, 112.5, 56.3. **IR** (neat, cm^{-1}): 2932, 2326, 2091, 1908, 1572, 1471, 1379, 1242, 1089, 1015, 809. **HRMS** (ESI) calculated for $C_{13}H_{10}O^{81}BrClS$: 329.9298 $[M]^+$, found: 329.9294.

(5-Bromo-2-methoxyphenyl)(4-(tert-butyl)phenyl)sulfane (7)

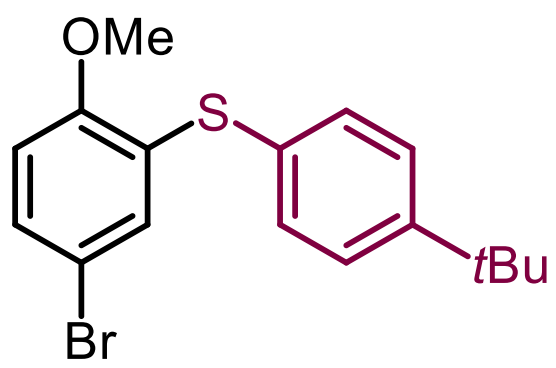

Prepared, following the general procedure from (5-bromo-2-methoxyphenyl)triethylgermane (103.8 mg, 0.3 mmol, 1.0 equiv.) and 4-(*tert*-butyl)benzenethiol (103.5 μL, 0.60 mmol, 2.0 equiv.). The title product was obtained after purification by preparative TLC (n-pentane/EtOAc 10:1) as a yellowish solid (75.2 mg, 0.21 mmol, 71%).

$\boldsymbol{R_f}$ = 0.44 (n-pentane/Et_2O 40:1). **M.p.** = 111.2 °C. **^{1}H NMR** (600 MHz, $CDCl_3$) δ 7.42 – 7.36 (m, 4H), 7.24 (dd, J = 8.6, 2.4 Hz, 1H), 6.96 (d, J = 2.4 Hz, 1H), 6.73 (d, J = 8.7 Hz, 1H), 3.88 (s, 3H), 1.34 (s, 9H). **^{13}C{^{1}H} NMR** (151 MHz, $CDCl_3$) δ 155.4, 151.9, 133.5, 131.2, 129.7, 129.0, 128.4, 126.8, 113.5, 112.1, 56.3, 34.8, 31.4. **IR** (neat, cm^{-1}): 2960, 2329, 1909, 1572, 1468, 1378, 1240, 1064, 1020, 827. **HRMS** (ESI) calculated for $C_{17}H_{19}O^{79}BrS$: 350.0335 $[M]^+$, found: 350.0328.

(5-Bromo-2-methoxyphenyl)(phenyl)sulfane (8)

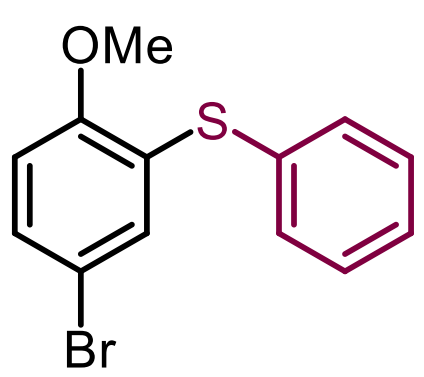

Prepared, following the general procedure from (5-bromo-2-methoxyphenyl)triethylgermane (103.8 mg, 0.3 mmol, 1.0 equiv.) and benzenethiol (61.2 μL mg, 0.60 mmol, 2.0 equiv.). The title product was obtained after purification by preparative TLC (Pentane/EtOAc 10:1) as a yellowish liquid (50.0 mg, 0.19 mmol, 62%).

***R*$_f$** = 0.38 (Pentane/Et_2O 40:1). **^{1}H NMR** (600 MHz, $CDCl_3$) δ 7.42 – 7.40 (m, 2H), 7.38 – 7.32 (m, 3H), 7.28 (dd, *J* = 8.7, 2.4 Hz, 1H), 7.03 (d, *J* = 2.4 Hz, 1H), 6.75 (d, *J* = 8.7 Hz, 1H), 3.86 (s, 3H). **^{13}C{^{1}H} NMR** (151 MHz, $CDCl_3$) δ 155.9, 132.9, 132.7, 132.3, 130.3, 129.6, 128.2, 127.9, 113.4, 112.3, 56.3. **IR** (neat, cm^{-1}): 2934, 1879, 1573, 1469, 1469, 1377, 1240, 1064, 1021, 745. **HRMS** (ESI) calculated for $C_{13}H_{11}O^{81}BrS$: 295.9688 $[M]^+$, found: 295.9687. These data are in agreement with those reported previously in the literature [11].

(3-((4-Fluorophenyl)thio)phenyl)trimethylsilane (9)

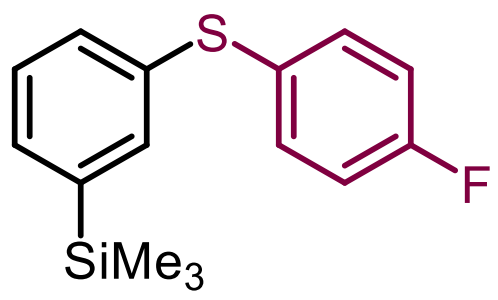

Prepared, following the general procedure from trimethyl(3-(triethylgermyl)phenyl)silane (30.9 mg, 0.1 mmol, 1.0 equiv.) and 4-fluorobenzenethiol (21.3 µL, 0.20 mmol, 2.0 equiv.). The title product was obtained after purification by column chromathography (*n*-pentane) as a colorless liquid (12.6 mg, 0.046 mmol, 46%).

***R*$_f$** = 0.66 (*n*-pentane). **^{1}H NMR** (600 MHz, CD_2Cl_2) δ 7.46 (dt, *J* = 1.9, 0.8 Hz, 1H), 7.40 – 7.35 (m, 3H), 7.27 (t, *J* = 7.5 Hz, 1H), 7.22 (ddd, *J* = 7.8, 2.1, 1.2 Hz, 1H), 7.08 – 7.00 (m, 2H), 0.23 (s, 9H). **^{13}C{^{1}H} NMR** (151 MHz, CD_2Cl_2) δ 162.7 (d, *J* = 246.8 Hz), 136.1, 135.4, 134.2 (d, *J* = 8.1 Hz), 132.2, 131.0 (d, *J* = 3.1 Hz), 130.9, 129.0, 116.7 (d, *J* = 21.8 Hz), -1.2. **^{19}F NMR** (564 MHz, CD_2Cl_2) δ -115.15 (tt, *J* = 8.6, 5.2 Hz, 1F). **IR** (neat, cm^{-1}): 2955, 1588, 1487, 1226, 1128, 832, 691. **HRMS** (ESI) calculated for $C_{15}H_{17}FSSi$: 276.0804 $[M]^+$, found: 276.0798.

(3-Bromo-5-((4-fluorophenyl)thio)phenyl)trimethylsilane (10)

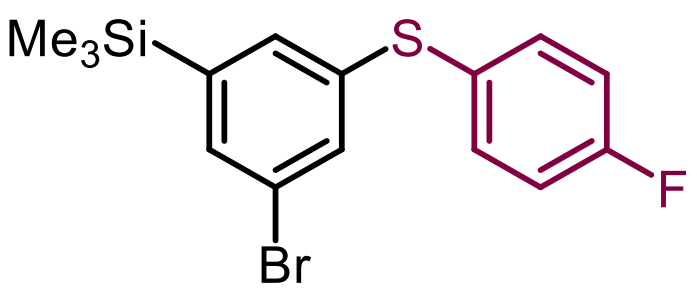

Prepared, following the general procedure from (3-bromo-5-(triethylgermyl)phenyl)trimethylsilane (116.4 mg, 0.3 mmol, 1.0 equiv.) and 4-fluorobenzenethiol (64.0 µL, 0.60 mmol, 2.0 equiv.) under neat conditions. The title product was obtained after purification by preparative TLC (*n*-pentane) as a colorless liquid (55.8 mg, 0.16 mmol, 52%).

Caution: When performing the reaction under neat conditions the reaction mixture becomes very hot upon addition of PIFA, especially on larger scale. Cooling with an ice bath should be considered.

***R*$_f$** = 0.72 (*n*-pentane). **^{1}H NMR** (600 MHz, CD_2Cl_2) δ 7.46 – 7.41 (m, 3H), 7.33 (dd, *J* = 1.9, 0.9 Hz, 1H), 7.26 (t, *J* = 1.9 Hz, 1H), 7.11 – 7.06 (m, 2H), 0.23 (s, 9H). **^{13}C{^{1}H} NMR** (151 MHz, CD_2Cl_2) δ 163.2 (d, *J* = 248.2 Hz), 145.2, 139.3, 135.3 (d, *J* = 8.5 Hz), 134.4, 132.8, 132.0, 129.3 (d, *J* = 3.5 Hz), 123.6, 117.0 (d, *J* = 22.2 Hz), -1.3. **^{19}F NMR** (564 MHz, CD_2Cl_2) δ -113.69 (tt, *J* = 8.6, 5.2 Hz, 1F). **IR** (neat, cm^{-1}): 2955, 1487, 1227, 1133, 831, 747, 680. **HRMS** (ESI) calculated for $C_{15}H_{16}{}^{79}BrFSSi$: 353.9909 $[M]^+$, found: 353.9904.

(4-Fluorophenyl)(3-(trifluoromethyl)phenyl)sulfane (11)

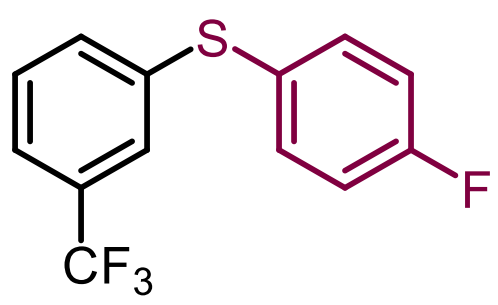

Prepared, upon modification of the general procedure from triethyl(3-(trifluoromethyl)phenyl)germane (30.4 mg, 0.1 mmol, 1.0 equiv.) and 4-fluorobenzenethiol (21.3 µL, 0.20 mmol, 2.0 equiv.) under neat conditions. After 2 hours, the mixture was filtered through a plug of silica rinsing with diethyl ether.

The resulting solution was concentrated under reduced pressure and 4-fluorobenzenethiol (21.3 µL, 0.20 mmol, 2.0 equiv.) and PIFA (86.0 mg, 0.2 mmol, 2.0 equiv.) were subsequently added. After stirring for 2 hours, the mixture was filtered through a plug of silica rinsing with diethyl ether. The resulting solution was concentrated under reduced pressure, and the title product was obtained after purification by column chromatography (*n*-pentane) as a yellowish liquid (19.4 mg, 0.072 mmol, 72%).

Caution: When performing the reaction under neat conditions the reaction mixture becomes very hot upon addition of PIFA, especially on larger scale. Cooling with an ice bath should be considered.

***R*$_f$** = 0.62 (*n*-pentane). **^{1}H NMR** (600 MHz, $CDCl_3$) δ 7.47 – 7.41 (m, 4H), 7.38 – 7.32 (m, 2H), 7.09 (dd, *J* = 8.6, 8.6 Hz 2H). **^{13}C{^{1}H} NMR** (151 MHz, $CDCl_3$) δ 163.2 (d, *J* = 249.5 Hz), 139.3, 135.6 (d, *J* = 8.3 Hz), 131.2, 131.7 (d, *J* = 32.6 Hz,), 129.6, 128.3 (d, *J* = 3.3 Hz), 125.4 (q, *J* = 4.1 Hz), 123.9 (q, *J* = 272.8 Hz), 123.2 (q, *J* = 3.8 Hz), 117.0 (d, *J* = 21.9). **^{19}F NMR** (564 MHz, $CDCl_3$) δ -62.9 (s, 3F), -112.2 (tt, *J* = 8.5, 4.1 Hz, 1F). **IR** (neat, cm^{-1}): 1588, 1487, 1319, 1228, 1126, 829, 698. **HRMS** (ESI) calculated for $C_{13}H_8F_4S$: 272.0283 [M]$^+$, found: 272.0275.

5-((4-Fluorophenyl)thio)-2-methoxybenzonitrile (12)

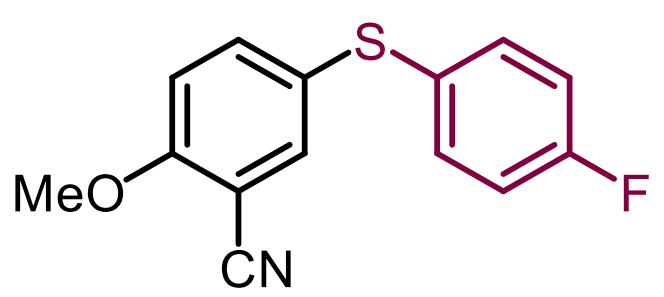

Prepared, following the general procedure from 2-methoxy-5-(triethylgermyl)benzonitrile (87.6 mg, 0.3 mmol, 1.0 equiv.) and 4-fluorobenzenethiol (64.0 µL, 0.60 mmol, 2.0 equiv.). The title product was obtained after purification by preparative TLC (*n*-pentane /EtOAc 40:1) as a yellowish liquid (68.5 mg, 0.264 mmol, 88%).

***R*$_f$** = 0.41 (*n*-pentane /Et_2O 40:1). **^{1}H NMR** (600 MHz, $CDCl_3$) δ 7.52 – 7.50 (m, 2H), 7.31 – 7.29 (m, 2H), 7.04 – 7.01 (m, 2H), 6.93 – 6.90 (m, 1H), 3.93 (s, 3H). **^{13}C{^{1}H} NMR** (151 MHz, $CDCl_3$) δ 162.6 (d, *J* = 248.4 Hz), 160.8, 137.6, 136.4, 133.5 (d, *J* = 8.0 Hz), 130.4 (d, *J* = 2.9 Hz,), 128.2, 116.8 (d, *J* = 21.9 Hz), 112.4, 103.2, 56.3. *Note*: The signal of the CN carbon was not observed in ^{13}C NMR. **^{19}F NMR** (564 MHz, $CDCl_3$) δ -113.53 (tt, *J* = 8.6, 5.2 Hz, 1F). **IR** (neat, cm^{-1}): 2944, 2229, 1688, 1589, 1485, 1390, 1282, 1222, 1141, 1016, 820. **HRMS** (ESI) calculated for $C_{14}H_{10}OFNS$: 259.0462 [M]$^+$, found: 259.0460.

5-((4-Bromophenyl)thio)-2-methoxybenzonitrile (13)

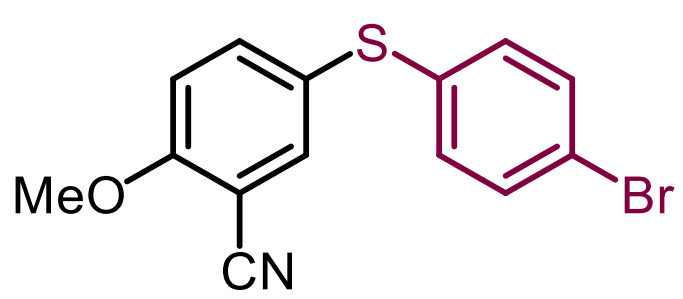

Prepared, following the general procedure from 2-methoxy-5-(triethylgermyl)benzonitrile (87.6 mg, 0.3 mmol, 1.0 equiv.) and 4-bromobenzenethiol (113.4 mg, 0.60 mmol, 2.0 equiv.). The title product was obtained after purification by column chromatography (*n*-pentane /EtOAc 9:1) as an off-white solid (55.3 mg, 0.173 mmol, 58%).

Note: Performing the reaction on a scale of 2.0 mmol with a reaction time of 17 h, the title product was obtained after purification by column chromatography (*n*-pentane /EtOAc 9:1) as an off-white solid (384.3 mg, 1.200 mmol, 60%).

***R*$_f$** = 0.30 (*n*-pentane /EtOAc 9:1). **M.p.** = 125–127 °C. **^{1}H NMR** (600 MHz, $CDCl_3$) δ 7.60 – 7.56 (m, 2H), 7.42 – 7.40 (m, 2H), 7.10 – 7.08 (m, 2H), 6.95 (d, *J* = 8.7 Hz, 1H), 3.94 (s, 3H). **^{13}C{^{1}H} NMR** (151 MHz,

$CDCl_3$) δ 161.2, 139.0, 137.8, 135.5, 132.6, 131.5, 126.4, 121.3, 115.6, 112.6, 103.4, 56.5. **IR** (neat, cm^{-1}) 2923, 2229, 1588, 1474, 1277, 1083, 1004, 816, 746. **HRMS** (ESI) calculated for $C_{14}H_{10}O^{79}BrNS$: 318.9661 $[M]^+$, found: 318.9666.

(4-Bromo-3-chloro-2-methylphenyl)(4-fluorophenyl)sulfane (14)

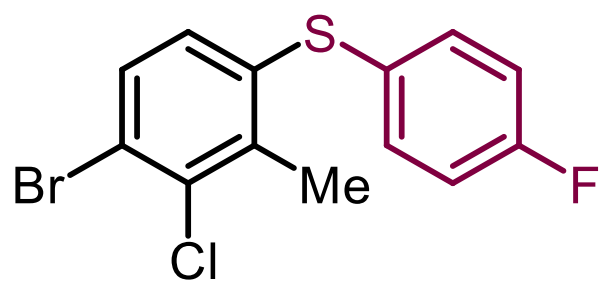

Prepared, following the general procedure from (4-bromo-3-chloro-2-methylphenyl)triethylgermane (31.3 mg, 0.1 mmol, 1.0 equiv.) and 4-fluorobenzenethiol (21.3 μL, 0.20 mmol, 2.0 equiv.). After 2 hours, the mixture was filtered through a plug of silica rinsing with diethyl ether. The resulting solution was concentrated under reduced pressure and 4-fluorobenzenethiol (21.3 μL, 0.20 mmol, 2.0 equiv.) and PIFA (86.0 mg, 0.2 mmol, 2.0 equiv.) were subsequently added. After stirring for 2 hours, the mixture was filtered through a plug of silica rinsing with diethyl ether. The resulting solution was concentrated under reduced pressure, and the title product was obtained after purification by preparative TLC (*n*-pentane) as a yellowish liquid (24.1 mg, 0.073 mmol, 73%).

R_f = 0.70 (*n*-pentane). **^{1}H NMR** (600 MHz, $CDCl_3$) δ 7.35 (d, *J* = 8.5, 1H), 7.31 – 7.28 (m, 2H), 7.07 – 7.03 (m, 2H), 6.84 (d, *J* = 8.5 Hz, 1H), 2.54 (s, 3H). **^{13}C{^{1}H} NMR** (151 MHz, $CDCl_3$) δ 162.7 (d, *J* = 248.3 Hz), 138.1, 137.0, 135.5, 134.1 (d, *J* = 8.3 Hz), 131.2, 129.6, 129.2 (d, *J* = 3.6 Hz), 121.8, 116.9 (d, *J* = 21.9 Hz), 19.4. **^{19}F NMR** (564 MHz, $CDCl_3$): δ -113.1– -113.2 (m, 1F). **IR** (neat, cm^{-1}): 2345, 1884, 1587, 1485, 1226, 1086, 823. **HRMS** (ESI) calculated for $C_{13}H_9{}^{79}BrClFS$: 329.9275 $[M]^+$, found: 329.9275.

(4-Methoxyphenyl)(2,3,5,6-tetrafluorophenyl)sulfane (15)

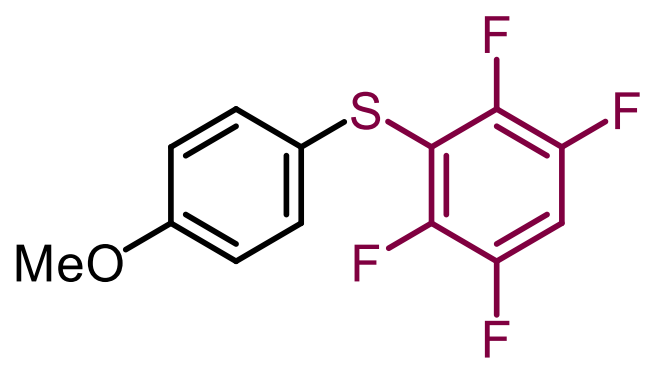

Prepared, following the general procedure from triethyl(4-methoxyphenyl)germane (80.1 mg, 0.3 mmol, 1.0 equiv.) and 2,3,5,6-tetrafluorobenzenethiol (109.5 mg, 0.6 mmol, 2.0 equiv.). The title product was obtained after purification by column chromatography (*n*-pentane) as a colorless liquid (27.6 mg, 0.096 mmol, 32%).

R_f = 0.14 (*n*-pentane). ^{1}**H NMR** (600 MHz, $CDCl_3$) δ 7.47 – 7.43 (m, 2H), 7.03 (tt, *J* = 9.6, 7.2 Hz, 1H), 6.86 – 6.82 (m, 2H), 3.79 (s, 3H). **^{13}C{^{1}H} NMR** (151 MHz, $CDCl_3$) δ 160.3, 147.7 – 145.1 (m), 147.1 – 145.1 (m), 134.7, 123.0, 116.8 (t, *J* = 19.9 Hz), 115.0, 106.5 (t, *J* = 22.8 Hz), 55.5. **^{19}F NMR** (564 MHz, $CDCl_3$) δ -133.76 (m), -137.99 (m). **IR** (neat, cm^{-1}): 3071, 2945, 2840, 2349, 2089, 1883, 1628, 1592, 1486, 1435, 1378, 1288, 1244, 1174, 1122, 1030, 915, 889, 827, 712, 671. **HRMS** (APCI) calculated for $C_{13}H_8OF_4S$: 288.0227 $[M]^+$, found: 288.0224.

Methyl(4-((2,4,6-triisopropylphenyl)thio)phenyl)sulfane (16)

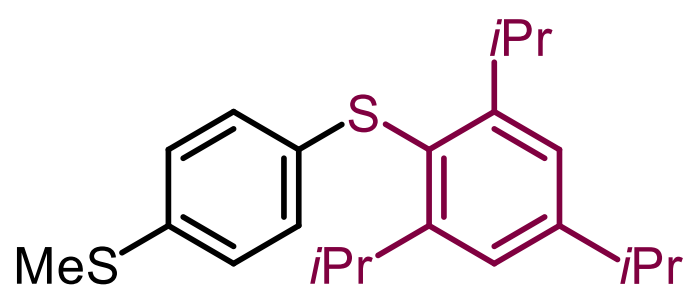

Prepared, following the general procedure from triethyl(4-(methylthio)phenyl)germane (28.3 mg, 0.1 mmol, 1.0 equiv.) and 2,4,6-triisopropylbenzenethiol (47.0 mg, 0.2 mmol, 2.0 equiv.). The title product was obtained after purification by column chromatography (*n*-pentane) as a colorless liquid (20.8 mg, 0.058 mmol, 58%).

R_f = 0.48 (*n*-pentane). **^{1}H NMR** (600 MHz, $CDCl_3$) δ 7.13 (s, 2H), 7.10 – 7.07 (m, 2H), 6.86 – 6.82 (m, 2H), 3.67 (hept, *J* = 6.9 Hz, 2H), 2.94 (hept, *J* = 7.0 Hz, 1H), 2.42 (s, 3H), 1.28 (d, *J* = 7.0 Hz, 6H), 1.14 (d, *J* = 6.9 Hz, 12H). **^{13}C{^{1}H} NMR** (151 MHz, $CDCl_3$) δ 154.0, 151.5, 137.4, 134.6, 127.8, 126.3, 125.2, 122.7, 34.8, 32.1, 24.3, 24.1, 16.5. **IR** (neat, cm^{-1}): 2960, 2926, 2868, 1596, 1565, 1469, 1384, 1361, 1312, 1164, 1103, 1063, 1011, 962, 938, 877, 807, 738, 706. **HRMS** (APCI) calculated for $C_{22}H_{30}S_2$: 358.1783 $[M]^+$, found: 358.1782.

(4-Fluorophenyl)(2,4,6-triisopropylphenyl)sulfane (17)

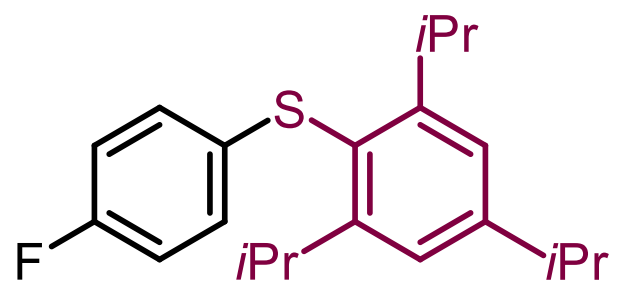

Prepared, following the general procedure from triethyl(4-fluorophenyl)germane (25.5 mg, 0.1 mmol, 1.0 equiv.) and 2,4,6-triisopropylbenzenethiol (47.0 mg, 0.2 mmol, 2.0 equiv.). The title product was obtained after purification by preparative TLC (*n*-pentane) as a colorless liquid (21.0 mg, 0.064 mmol, 64%).

R_f = 0.48 (*n*-pentane). **^{1}H NMR** (600 MHz, $CDCl_3$) δ 7.11 (s, 2H), 6.90 – 6.86 (m, 4H), 3.69 (hept, *J* = 6.9 Hz, 2H), 2.94 (hept, *J* = 6.9 Hz, 1H), 1.30 (d, *J* = 6.9 Hz, 6H), 1.15 (d, *J* = 6.9 Hz, 12H). **^{13}C{^{1}H} NMR** (151 MHz, $CDCl_3$) δ 160.7 (d, *J* = 243.3 Hz), 153.7, 151.0, 135.4 (d, *J* = 3.1 Hz), 127.1 (d, *J* = 7.4 Hz), 125.3, 122.5, 115.9 (d, *J* = 21.8 Hz), 34.5, 31.8, 24.4, 24.1. **^{19}F NMR** (564 MHz, $CDCl_3$) δ -119.06 (tt, *J* = 8.1, 5.5 Hz). **IR** (neat, cm^{-1}): 3049, 2961, 2870, 2352, 2069, 1999, 1873, 1764, 1593, 1564, 1487, 1462, 1384, 1362, 1311, 1225, 1156, 1088, 1063, 1012, 936, 878, 820, 760, 731, 686, 656. **HRMS** (APCI) calculated for $C_{21}H_{27}FS$: 330.1812 $[M]^+$, found: 330.1811.

2-Methoxy-5-(o-tolylthio)benzonitrile (18)

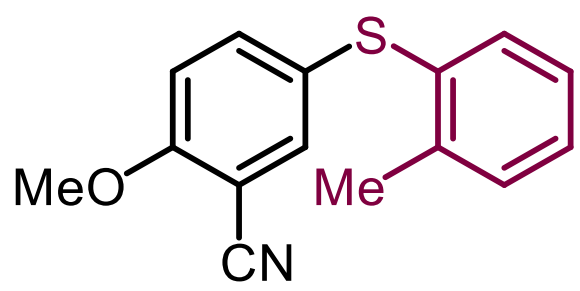

Prepared, following the general procedure from 2-methoxy-5-(triethylgermyl)benzonitrile (103.8 mg, 0.3 mmol, 1.0 equiv.) and 2-methylbenzenethiol (70.7 µL, 0.60 mmol, 2.0 equiv.). The title product was obtained after purification by preparative TLC (*n*-pentane /EtOAc 10:1) as a yellowish liquid (56.2 mg, 0.220 mmol, 73%).

R_f = 0.11 (*n*-pentane /Et_2O 40:1). **^{1}H NMR** (600 MHz, $CDCl_3$) δ 7.45 (dd, *J* = 8.8, 2.4, 1H), 7.42 (d, *J* = 2.3 Hz, 1H), 7.25 – 7.20 (m, 2H), 7.16 (dtd, *J* = 14.7, 7.7, 1.7 Hz, 2H), 6.92 (d, *J* = 8.8 Hz, 1H), 3.92 (s, 3H), 2.36 (s, 3H). **^{13}C{^{1}H} NMR** (151 MHz, $CDCl_3$) δ 160.5, 139.3, 137.1, 135.9, 134.0, 132.1, 131.0, 128.2, 127.8, 127.1, 112.4, 103.2, 56.4, 20.6. **IR** (neat, cm^{-1}): 2941, 2228, 1589, 1462, 1280, 1018, 749. **HRMS** (ESI) calculated for $C_{15}H_{13}ONS$: 255.0712 $[M]^+$, found: 255.0711.

(4-Fluorophenyl)(mesityl)sulfane (19)

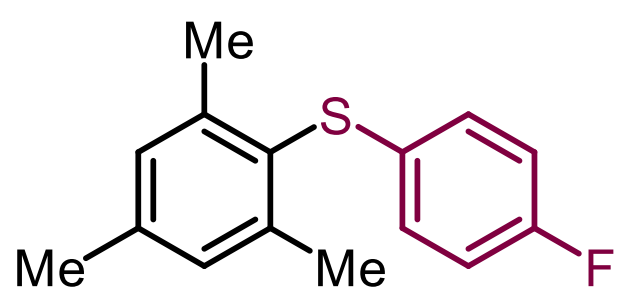

Prepared, following the general procedure from triethylmesitylgermane (83.7 mg, 0.3 mmol, 1.0 equiv.) and 4-fluorobenzenethiol (64.0 µL, 0.6 mmol, 2.0 equiv.). The title product was obtained after purification by preparative HPLC (*n*-hexane/EtOAc 99:1) as a colourless liquid (36.4 mg, 0.148 mmol, 49%).

R_f = 0.71 (*n*-hexane). **^{1}H NMR** (600 MHz, $CDCl_3$) δ 7.01 (s, 2H), 6.89 (d, *J* = 6.8 Hz, 4H), 2.38 (s, 6H), 2.32 (s, 3H). **^{13}C{^{1}H} NMR** (151 MHz, $CDCl_3$) δ 160.8 (d, *J* = 243.9 Hz), 143.7, 139.5, 133.5 (d, *J* = 3.2 Hz), 129.6, 127.5, 127.4 (d, *J* = 7.7 Hz), 116.1 (d, *J* = 22.1 Hz), 21.8, 21.3. **^{19}F NMR** (376 MHz, CD_2Cl_2) δ -118.63 (tt, *J* = 6.8, 6.7 Hz). **IR** (neat, cm^{-1}): 2922, 2853, 1601, 1589, 1463, 1435, 1396, 1376, 1293, 1226, 1155, 1086, 1058, 1032, 1012, 851, 821, 720. **HRMS** (EI) calculated for $C_{15}H_{15}FS$: 246.0878 $[M]^+$, found: 246.0877.

2-((4-Fluorophenyl)thio)furane (20)

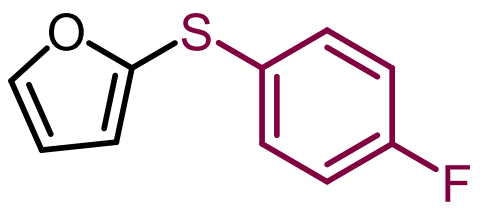

Prepared, following the general procedure from triethyl(furan-2-yl)germane (68.0 mg, 0.2 mmol, 1.0 equiv.), 4-fluorobenzenethiol (64.0 µL, 0.6 mmol, 2.0 equiv.) and DBU (89.7 µL, 0.6 mmol, 2.0 equiv.). The title product was obtained after purification by column chromatography (*n*-pentane) as a colorless liquid (20.0 mg, 0.102 mmol, 34%).

R_f = 0.34 (*n*-pentane). **^{1}H NMR** (400 MHz, CD_2Cl_2) δ 7.58 (d, *J* = 0.9 Hz, 1H), 7.24 – 7.18 (m, 2H), 7.04 – 6.95 (m, 2H), 6.75 (d, *J* = 2.3 Hz, 1H), 6.49 (dd, *J* = 3.2, 1.9 Hz, 1H). **^{13}C{^{1}H} NMR** (101 MHz, CD_2Cl_2) δ 162.3 (d, *J* = 245.8 Hz), 147.1, 143.9, 131.5 (d, *J* = 2.5 Hz), 130.7 (d, *J* = 8.1 Hz), 119.6, 116.5 (d, *J* = 22.1 Hz), 112.3. **^{19}F NMR** (376 MHz, CD_2Cl_2) δ -116.30 (tt, *J* = 9.1, 5.2 Hz). **IR** (neat, cm^{-1}): 3132, 2925, 2064, 1882, 1785, 1692, 1632, 1589, 1487, 1459, 1398, 1292, 1224, 1153, 1111, 1087, 1060, 1006, 907, 882, 821, 743. **HRMS** (APCI) calculated for $C_{10}H_7OFS$: 194.0196 $[M]^+$, found: 194.0196.

(4-Fluorophenyl)(phenyl)selane (21)

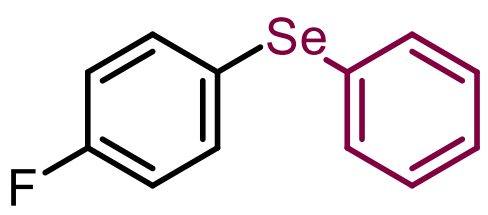

Prepared, following the general procedure from triethyl(4-fluorophenyl)germane (25.5 mg, 0.1 mmol, 1.0 equiv.) and benzeneselenol (21.2 µL, 0.2 mmol, 2.0 equiv.). The title product was obtained after purification by column chromatography (*n*-pentane) as a yellow liquid (16.1 mg, 0.064 mmol, 64%).

R_f = 0.36 (*n*-pentane). **^{1}H NMR** (600 MHz, CD_2Cl_2) δ 7.53 – 7.48 (m, 2H), 7.44 – 7.40 (m, 2H), 7.28 – 7.25 (m, 3H), 7.04 – 6.99 (m, 2H). **^{13}C{^{1}H} NMR** (151 MHz, CD_2Cl_2) δ 163.0 (d, *J* = 246.8 Hz), 136.2 (d, *J* = 7.8 Hz), 132.7, 132.1, 129.8, 127.6, 125.8 (d, *J* = 2.6 Hz), 116.9 (d, *J* = 21.5 Hz). **^{19}F NMR** (564 MHz, CD_2Cl_2) δ -114.87 (tt, *J* = 8.9, 5.5 Hz). **IR** (neat, cm^{-1}): 3062, 2924, 2852, 2327, 2111, 1883, 1695, 1581, 1482, 1438, 1393, 1292, 1224, 1155, 1087, 1063, 1015, 822, 735, 689. **HRMS** (APCI) calculated for $C_{12}H_9FSe$: 251.9848 $[M]^+$, found: 251.9846.

Hexyl(4-methoxyphenyl)sulfane (22)

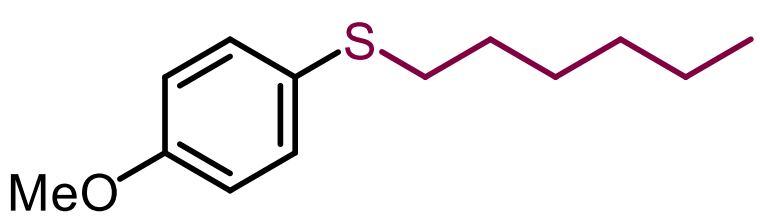

Prepared, following the general procedure from triethyl(4-methoxyphenyl)germane (25.5 mg, 0.1 mmol, 1.0 equiv.) and 1-hexanethiol (28.4 µL, 0.2 mmol, 2.0 equiv.). The title product was obtained after purification by column chromatography (*n*-pentane, *n*-pentane/Et_2O 80:1) as a yellow liquid (17.1 mg, 0.076 mmol, 76%).

R_f = 0.20 (*n*-pentane/Et_2O 80:1). **^{1}H NMR** (600 MHz, $CDCl_3$) δ 7.36 – 7.32 (m, 2H), 6.87 – 6.81 (m, 2H), 3.79 (s, 3H), 2.81 (t, *J* = 7.3 Hz, 2H), 1.61 – 1.54 (m, 2H), 1.42 – 1.35 (m, 2H), 1.31 – 1.20 (m, 4H), 0.88 (t,

J = 7.0 Hz, 2H). **$^{13}C\{^1H\}$ NMR** (151 MHz, $CDCl_3$) δ 158.8, 133.0, 127.1, 114.6, 55.5, 36.0, 31.5, 29.5, 28.5, 22.7, 14.2. **IR** (neat, cm^{-1}): 2925, 2855, 2330, 2105, 1993, 1870, 1741, 1672, 1592, 1491, 1460, 1283, 1241, 1174, 1100, 1032, 823, 724. **HRMS** (APCI) calculated for $C_{13}H_{20}OS$: 224.1229 $[M]^+$, found: 224.1228.

(4-methoxyphenyl)(phenethyl)sulfane (23)

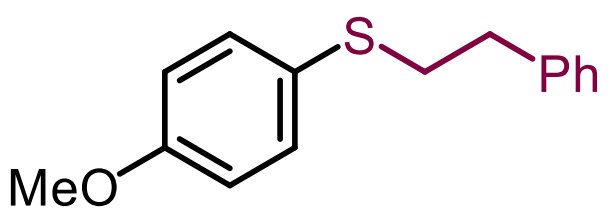

Prepared, following the general procedure from triethyl(4-methoxyphenyl)germane (25.5 mg, 0.1 mmol, 1.0 equiv.) and 2-phenylethanethiol (26.8 µL, 0.2 mmol, 2.0 equiv.). The title product was obtained after purification by column chromatography (*n*-pentane/Et_2O 80:1) as a yellow liquid (16.3 mg, 0.067 mmol, 67%).

R_f = 0.08 (*n*-pentane/Et_2O 80:1). **^1H NMR** (600 MHz, $CDCl_3$) δ 7.41 – 7.35 (m, 2H), 7.31 – 7.27 (m, 2H), 7.24 – 7.19 (m, 1H), 7.19 – 7.15 (m, 2H), 6.89 – 6.85 (m, 2H), 3.81 (s, 3H), 3.11 – 3.03 (m, 2H), 2.89 – 2.83 (m, 2H). **$^{13}C\{^1H\}$ NMR** (151 MHz, $CDCl_3$) δ 159.1, 140.5, 133.4, 128.7, 128.6, 126.5, 126.4, 114.7, 55.5, 37.4, 36.0. **IR** (neat, cm^{-1}): 3061, 3026, 2928, 2837, 2288, 2055, 1876, 1806, 1744, 1592, 1491, 1454, 1282, 1241, 1175, 1098, 1029, 823, 748, 699. **HRMS** (APCI) calculated for $C_{15}H_{16}OS$: 244.0916 $[M]^+$, found: 244.0915.

Methyl(4-(phenethylthio)phenyl)sulfane (24)

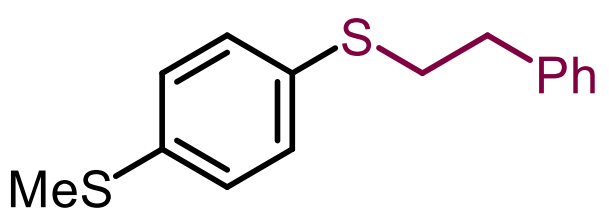

Prepared following the general procedure from triethyl(4-(methylthio)phenyl)germane (84.9 mg, 0.3 mmol, 1.0 equiv.) and 2-phenylethanethiol (80.3 µL, 0.6 mmol, 2.0 equiv.). The title product was obtained after purification by column chromatography (*n*-pentane) as a yellow liquid (38.4 mg, 0.147 mmol, 49%).

R_f = 0.60 (*n*-pentane). **^1H NMR** (600 MHz, CD_2Cl_2) δ 7.33 – 7.28 (m, 4H), 7.25 – 7.17 (m, 5H), 3.17 – 3.11 (m, 2H), 2.93 – 2.88 (m, 2H), 2.48 (s, 3H). **$^{13}C\{^1H\}$ NMR** (151 MHz, $CDCl_3$) δ 140.7, 137.1, 133.1, 130.6, 128.9, 128.8, 127.5, 126.8, 36.0, 35.9, 16.1. **IR** (neat, cm^{-1}): 3061, 3026, 2920, 2851, 2653, 2324, 2110, 1993, 1950, 1883, 1804, 1727, 1601, 1577, 1476, 1434, 1388, 1318, 1267, 1224, 1184, 1105, 1010, 963, 911, 808, 743, 698. **HRMS** (APCI) calculated for $C_{15}H_{16}S_2$: 260.0688 $[M]^+$, found: 260.0688.

(4-(*tert*-Butyl)phenyl)(phenethyl)sulfane (25)

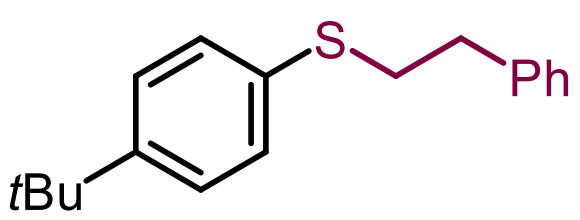

Prepared following the general procedure from (4-(tert-butyl)phenyl)triethylgermane (29.3 mg, 0.1 mmol, 1.0 equiv.) and 2-phenylethanethiol (26.8 µL, 0.2 mmol, 2.0 equiv.) under neat conditions. The title product was obtained after purification by column chromatography (*n*-pentane) as a yellow liquid (10.8 mg, 0.040 mmol, 40%).

Caution: When performing the reaction under neat conditions the reaction mixture becomes very hot upon addition of PIFA, especially on larger scale. Cooling with an ice bath should be considered.

***R**$_f$* = 0.09 (*n*-pentane). ^{1}**H NMR** (600 MHz, CD_2Cl_2) δ 7.35 – 7.33 (m, 2H), 7.32 – 7.28 (m, 4H), 7.21 (m, 3H), 3.15 (dd, *J* = 9.0, 6.6 Hz, 2H), 2.92 (dd, *J* = 9.0, 6.6 Hz, 2H), 1.31 (s, 9H). **^{13}C{^{1}H} NMR** (151 MHz, $CDCl_3$) δ 149.7, 140.9, 133.2, 129.6, 128.9, 128.8, 126.7, 126.4, 36.0, 35.8, 34.7, 31.4. **IR** (neat, cm^{-1}): 3064, 3027, 2958, 2864, 2655, 2324, 2112, 1992, 1944, 1892, 1801, 1725, 1656, 1600, 1492, 1454, 1395, 1363, 1315, 1268, 1224, 1198, 1156, 1119, 1014, 965, 910, 876, 821, 739, 698. **HRMS** (APCI) calculated for $C_{18}H_{22}S$: 270.1437 [M]$^+$, found: 270.1435.

Mesityl(phenethyl)sulfane (26)

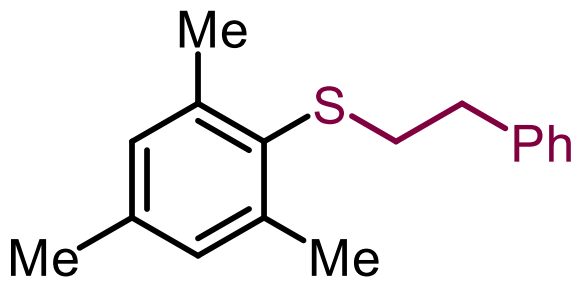

Prepared, following the general procedure from triethylmesitylgermane (28.0 mg, 0.1 mmol, 1.0 equiv.) and 2-phenylethanethiol (26.8 μL, 0.2 mmol, 2.0 equiv.). The title product was obtained after purification by column chromatography (*n*-pentane) as a colorless liquid (11.2 mg, 0.044 mmol, 44%).

***R**$_f$* = 0.28 (*n*-pentane). ^{1}**H NMR** (600 MHz, CD_2Cl_2) δ 7.28 – 7.24 (m, 2H), 7.21 – 7.16 (m, 1H), 7.16 – 7.14 (m, 2H), 6.94 (s, 2H), 2.89 – 2.85 (m, 2H), 2.82 – 2.78 (m, 2H), 2.48 (s, 6H), 2.26 (s, 3H). **^{13}C{^{1}H} NMR** (151 MHz, CD_2Cl_2) δ 143.2, 141.2, 138.4, 130.4, 129.3, 128.8, 128.7, 126.6, 37.0, 36.6, 22.1, 21.1. **IR** (neat, cm^{-1}): 3026, 2922, 2854, 1730, 1601, 1494, 1453, 1374, 1270, 1176, 1061, 1029, 850, 699, 521, 484. **HRMS** (ESI) calculated for $C_{17}H_{20}S$: 279.1178 [M+Na]$^+$, found: 279.1170.

Methyl ((*S*)-3-((4-fluorophenyl)thio)-2-methylpropanoyl)-L-prolinate (27)

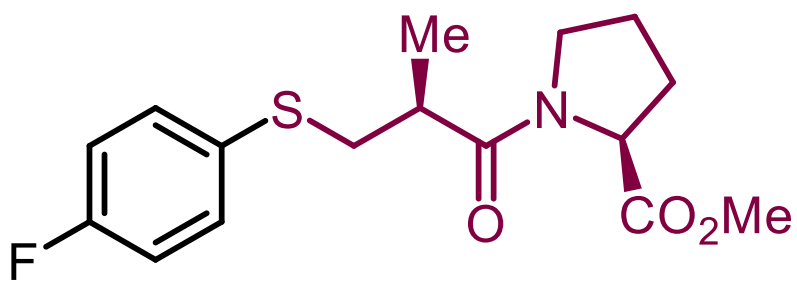

Prepared, following the general procedure from triethyl(4-fluorophenyl)germane (76.5 mg, 0.3 mmol, 1.0 equiv.) and methyl ((*S*)-3-mercapto-2-methylpropanoyl)-L-prolinate (*captopril* methyl ester) (138.8 mg, 0.6 mmol, 2.0 equiv.). The title product was obtained after purification by column chromatography (*n*-hexane/EtOAc 1:1; silica gel was neutralized using 0.1% NEt_3 in the eluent) as a colorless liquid (42.7 mg, 0.131 mmol, 44%).

***R**$_f$* = 0.38 (*n*-hexane/EtOAc 1:1). ^{1}**H NMR** (600 MHz, C_6D_6) δ 7.13 – 7.05 (m, 2H), 6.69 – 6.62 (m, 2H), 4.51 (dd, *J* = 7.1, 4.9 Hz, 1H), 3.35 (dd, *J* = 13.1, 8.5 Hz, 1H), 3.32 (s, 3H), 3.05 – 2.98 (m, 1H), 2.86 (dt, *J* = 9.8, 6.8 Hz, 1H), 2.72 (dd, *J* = 13.1, 5.6 Hz, 1H), 2.54 (dqd, *J* = 8.5, 6.8, 5.5 Hz, 1H), 1.54 – 1.46 (m, 3H), 1.22 – 1.12 (m, 4H). **^{13}C{^{1}H} NMR** (151 MHz, C_6D_6) δ 172.9, 172.8, 162.0 (d, *J* = 245.5 Hz), 132.3 (d, *J* = 7.8 Hz), 130.3 (d, *J* = 3.3 Hz), 116.17 (d, *J* = 22.1 Hz), 58.8, 51.6, 46.5, 38.7, 38.6, 28.9, 24.7, 17.5. **^{19}F NMR** (564 MHz, C_6D_6) δ -115.91 (tt, *J* = 8.5, 5.2 Hz). **IR** (neat, cm^{-1}): 2954, 2877, 1743, 1642, 1589, 1490, 1459, 1429, 1365, 1326, 1279, 1218, 1196, 1173, 1091, 1043, 1011, 829. **HRMS** (EI) calculated for $C_{16}H_{20}O_3NFS$: 325.1142 [M]$^+$, found: 325.1140.

4. Orthogonal Functionalization

4.1 Pd(I)-I-dimer catalyzed C-S bond formation

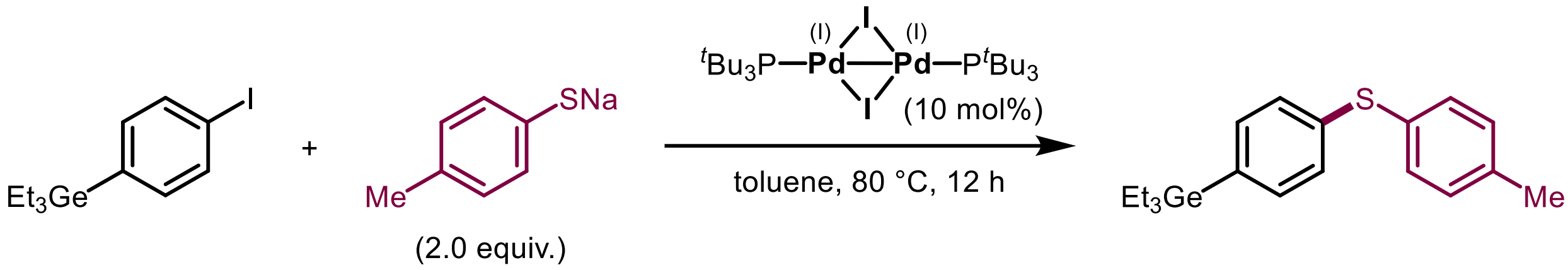

Triethyl(4-(*p*-tolylthio)phenyl)germane (29)

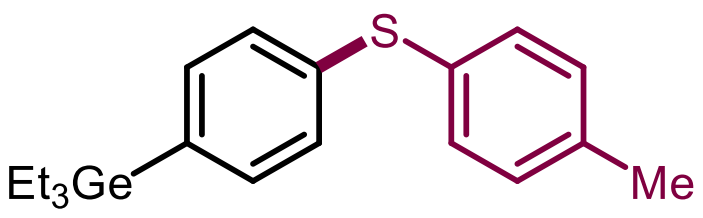

The coupling was carried out according to the literature [12]. In an Argon filled glovebox, in a 4 mL screw top vial triethyl(4-iodophenyl)germane (36.2 mg, 0.10 mmol, 1.0 equiv.) was dissolved in anhydrous toluene (0.5 mL) followed by addition of sodium 4-methylbenzenethiolate (32.2 mg, 0.22 mmol, 2.2 equiv.) and [Pd(μ-I)(P^{t}Bu$_3$)]$_2$ (8.7 mg, 10.0 μmol, 10.0 mol%). The reaction mixture was stirred for 12 h at 80 °C. Then the vial was taken out of the glovebox and was allowed to cool to room temperature. The solution was filtered through a pad of silica rinsing three times with Et_2O. Solvents were removed *in vacuo* and the crude mixture was purified by prepararive TLC (*n*-pentane). The title product was obtained as yellowish liquid (25.7 mg, 0.07 mmol, 72%).

***R*$_f$** = 0.62 (*n*-pentane). **^{1}H NMR** (600 MHz, $CDCl_3$): δ 7.32 (dd, *J* = 7.9, 5.3 Hz, 4H), 7.21 (d, *J* = 7.8 Hz, 2H), 7.15 (d, *J* = 7.8 Hz, 2H), 2.35 (s, 3H), 1.04 (t, *J* = 7.6 Hz, 9H), 0.96 (qd, *J* = 7.3, 1.7 Hz, 6H). **^{13}C{^{1}H} NMR** (151 MHz, $CDCl_3$): δ 137.9, 137.8, 137.4, 134.7, 132.8, 131.1, 130.2, 128.8, 21.3, 9.0, 4.3. **IR** (neat, cm^{-1}): 2950, 2870, 2322, 1900, 1574, 1486, 1379, 1067, 1013, 804, 700. **HRMS** (ESI) calculated for $C_{19}H_{26}{}^{74}GeS$: 360.0962 [M]$^+$, found: 360.0959.

4.2 Cu catalyzed C-S bond formation

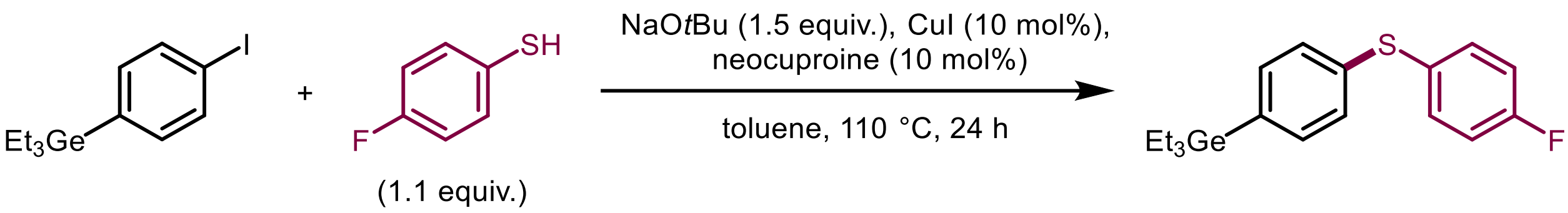

Triethyl(4-((4-fluorophenyl)thio)phenyl)germane (30)

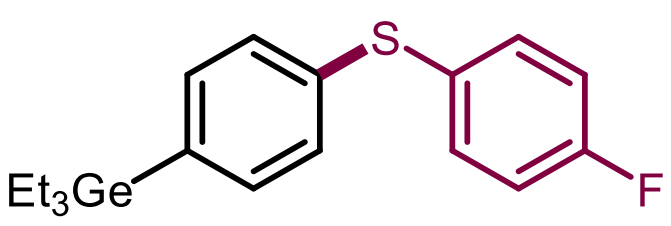

The coupling was carried out according to the literature [13]. In an Argon filled glovebox, in a 4 mL screw top vial triethyl(4-iodophenyl)germane (36.2 mg, 0.10 mmol, 1.0 equiv.) was dissolved in anhydrous toluene (0.3 mL) followed by addition of CuI (1.9 mg, 0.01 mmol, 0.10 equiv.), neocuproine (2.1 mg, 0.01 mmol, 0.10 equiv.) and NaOtBu (14.4 mg, 0.15 mmol, 1.5 equiv.). Then the vial was capped and taken out of the glovebox. 4-fluorobenzenethiol (11.0 μL, 0.11 mmol, 1.1 equiv.) was added and the reaction mixture was stirred for 12 h at 110 °C. It was

allowed to cool to room temperature. The solution was filtered through a pad of silica rinsing three times with Et_2O. Solvents were removed *in vacuo* and the crude mixture was purified by column chromatography (*n*-pentane). The title product was obtained as a colorless liquid (24.7 mg, 0.068 mmol, 68%).

R R_f = 0.36 (*n*-pentane). **^{1}H NMR** (600 MHz, $CDCl_3$): δ 7.43 – 7.39 (m, 2H), 7.34 (d, *J* = 8.1 Hz, 2H), 7.21 (d, *J* = 8.1 Hz, 2H), 7.07 – 7.01 (m, 2H), 1.07 – 1.03 (m, 9H), 0.99 – 0.94 (m, 6H). **^{13}C{^{1}H} NMR** (151 MHz, $CDCl_3$): δ 162.6 (d, *J* = 247.7 Hz), 138.4, 136.8, 134.8, 134.5 (d, *J* = 8.5 Hz), 130.0 (d, *J* = 3.4 Hz), 129.0, 116.5 (d, *J* = 22.1 Hz), 9.0, 4.3. **^{19}F NMR** (564 MHz, $CDCl_3$): δ -113.95 (tt, *J* = 8.5, 5.2 Hz, 1F). **IR** (neat, cm^{-1}): 3060, 2951, 2872, 1897, 1761, 1641, 1588, 1486, 1427, 1380, 1225, 1155, 1067, 1013, 967, 909, 809, 700. **HRMS** (ESI) calculated for $C_{18}H_{23}F^{74}GeS$: 364.0711 $[M]^+$, found: 364.0709.

4.3 Ni catalyzed C-S bond formation

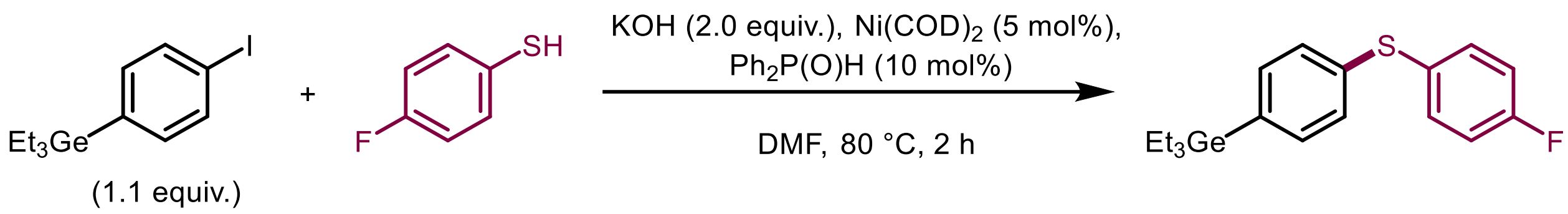

Triethyl(4-((4-fluorophenyl)thio)phenyl)germane (30)

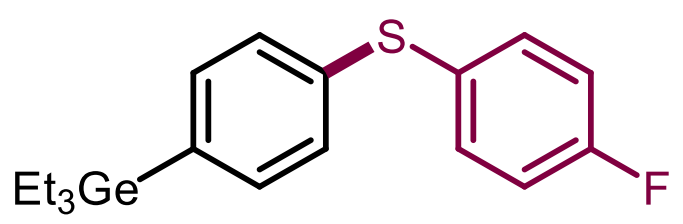

The coupling was carried out according to the literature [14]. In an Argon filled glovebox, in a 4 mL screw top vial triethyl(4-iodophenyl)germane (39.0 mg, 0.11 mmol, 1.1 equiv.) was dissolved in anhydrous DMF (0.6 mL) followed by addition of $Ni(COD)_2$ (1.4 mg, 0.005 mmol, 0.05 equiv.), diphenylphosphine oxide (2.0 mg, 0.01 mmol, 0.10 equiv.) and KOH (11.2 mg, 0.20 mmol, 2.0 equiv.). Then the vial was capped and taken out of the glovebox. 4-fluorobenzenethiol (10.2 µL, 0.10 mmol, 1.0 equiv.) was added and the reaction mixture was stirred for 2 h at 80 °C. It was allowed to cool to room temperature. The solution was filtered through a pad of silica rinsing three times with Et_2O. Solvents were removed *in vacuo* and the crude mixture was purified by column chromatography (*n*-pentane). The title product was obtained as a colorless liquid (11.0 mg, 0.033 mmol, 33%).

4.4 Light promoted C-S bond formation

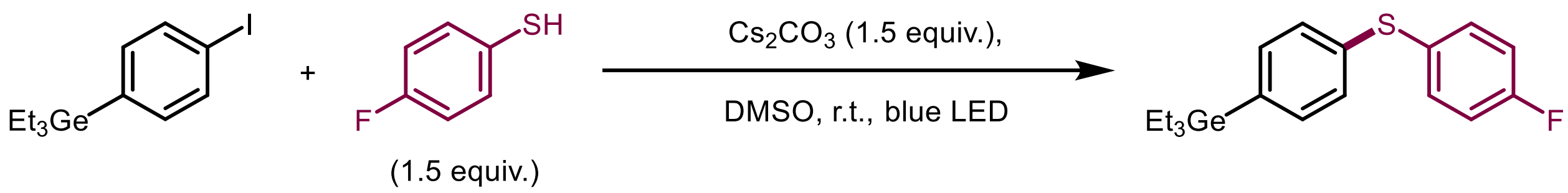

Triethyl(4-((4-fluorophenyl)thio)phenyl)germane (30)

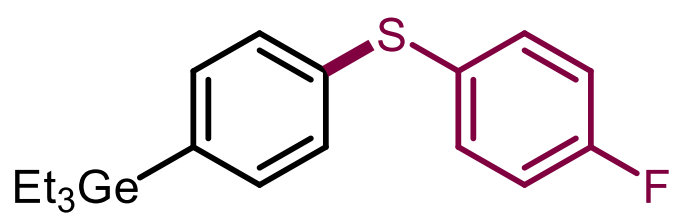

The coupling was carried out according to the literature [15]. Under air, in a 4 mL screw top vial triethyl(4-iodophenyl)germane (36.2 mg, 0.10 mmol, 1.0 equiv.) was dissolved in anhydrous DMSO (0.75 mL) followed by addition of 4-fluorobenzenethiol (16.0 µL, 0.15 mmol, 1.5 equiv.) and Cs_2CO_3 (49.0 mg, 0.15 mmol, 1.5 equiv.). The mixture was degassed by bubbling Ar through it for 10 min. Then, the vial was irradiated with blue LED (450 nm, 30W) for 6 h. 1,4-difluorobenzene (5.2 µL, 0.05 mmol) was added as internal standard, and the crude mixture was analyzed by NMR. The product was formed in 74% yield based on quantitative ^{19}F NMR.

4.5 Pd$^{(I)}$-I-dimer catalyzed Kumada Cross-coupling

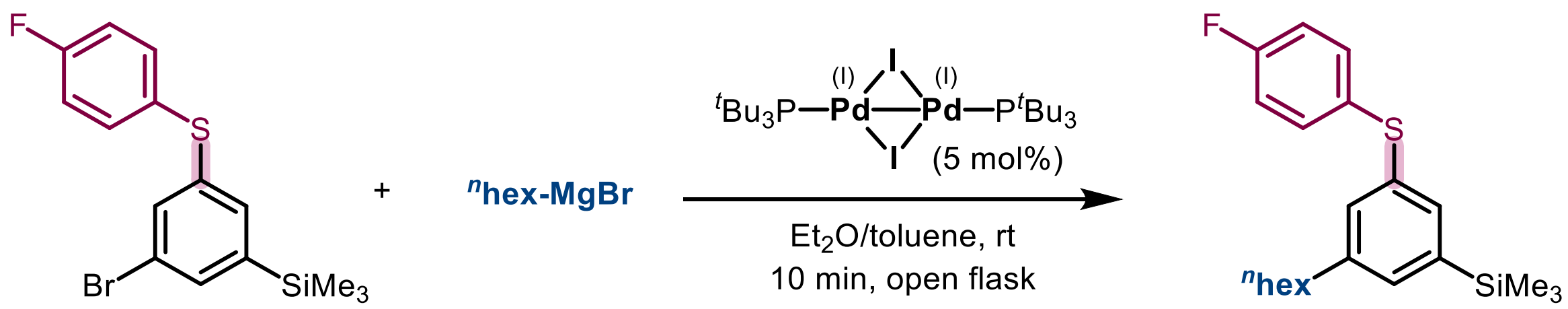

(3-((4-Fluorophenyl)thio)-5-hexylphenyl)trimethylsilane (32)

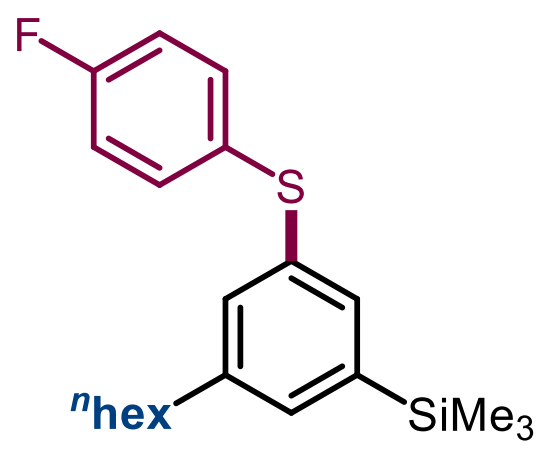

The coupling was carried out according to the literature [16]. Under air, an oven dried 4 mL screw top vial equipped with magnetic stirring bar was charged with (3-bromo-5-((4-fluorophenyl)thio)phenyl)trimethylsilane (95.9 mg, 0.27 mmol, 1.0 equiv.) and [Pd(µ-I)(P^{t}Bu$_3$)]$_2$ (11.8 mg, 0.0135 mmol, 5.0 mol%) in anhydrous toluene (1.0 mL). ^{n}HexMgBr (2.0 M in Et_2O, 270 µL, 0.54 mmol, 2.0 equiv.) was added dropwise. The reaction mixture was stirred in an open vial for 10 minutes and was then diluted with 2 mL of pentane. The mixture was filtered through a pad of silica and rinsed with diethyl ether. Solvents were removed under reduced pressure and the product was obtained after purification by preparative TLC (*n*-pentane) as a yellowish liquid (92.3 mg, 0.26 mmol, 95%).

***R*$_f$** = 0.42 (*n*-pentane). **^{1}H NMR** (600 MHz, $CDCl_3$): δ 7.37 – 7.34 (m, 2H), 7.29 – 7.28 (m, 1H), 7.18 (s, 1H), 7.08 (dd, *J* = 1.8, 1.8 Hz, 1H), 7.02 (dd, *J* = 8.7, 8.7 Hz, 2H), 2.55 (t, *J* = 7.8 Hz, 2H) 1.59 – 1.55 (m, 2H), 1.33 – 1.27 (m, 6H), 0.90 – 0.87 (m, 3H), 0.24 (s, 9H). **$^{13}C\{^1H\}$ NMR** (151 MHz, $CDCl_3$): δ 162.3 (d, *J* = 247.3 Hz), 143.5, 141.9, 135.4, 133.5 (d, *J* = 8.0 Hz), 132.7, 132.1, 131.1 (d, *J* = 3.3 Hz), 131.0, 116.4 (d, *J* = 21.9 Hz), 36.0, 31.8, 31.5, 29.1, 22.8, 14.2, -1.0. **^{19}F NMR** (564 MHz, $CDCl_3$): δ -114.73 (tt, *J* = 8.7, 4.4 Hz 1F). **IR** (neat, cm^{-1}): 2927, 2102, 1587, 1487, 1226 1136, 831, 753. **HRMS** (ESI) calculated for $C_{21}H_{29}FSSi$: 360.1743 $[M]^+$, found: 360.1740.

4.6 Iodination of (3-((4-fluorophenyl)thio)-5-hexylphenyl)trimethylsilane

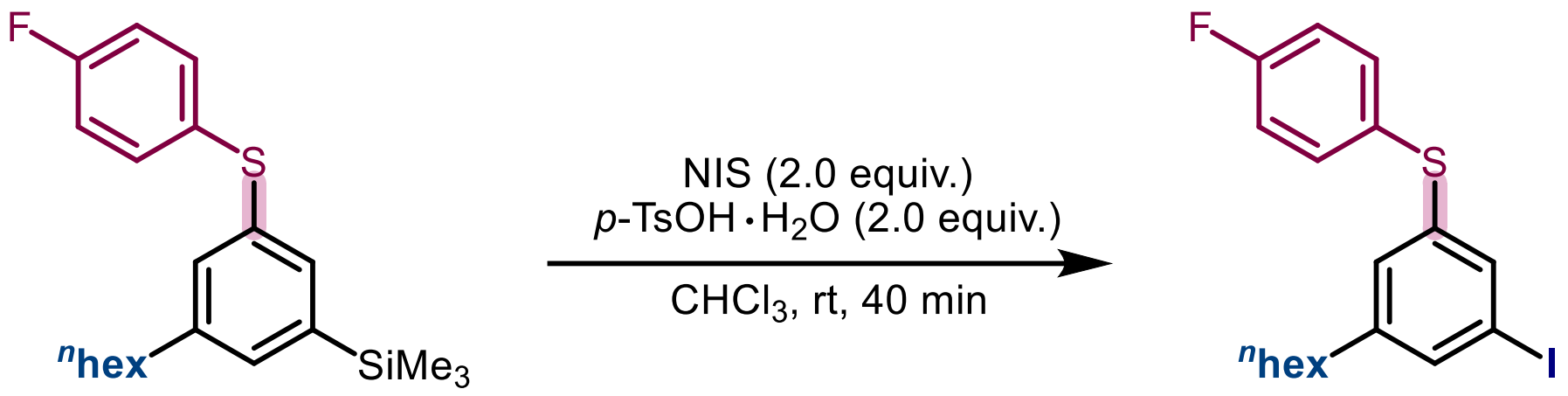

(4-Fluorophenyl)(3-hexyl-5-iodophenyl)sulfane (33)

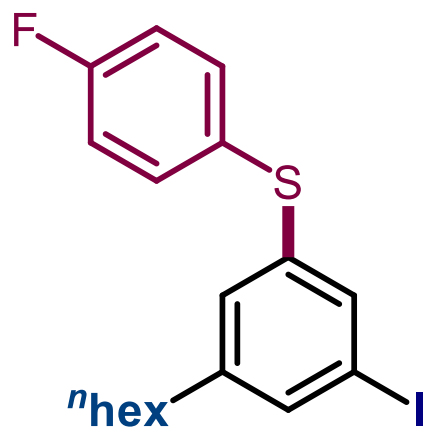

The reaction was carried out according to the literature [3]. A 4 mL screw top vial equipped with magnetic stir bar was charged with (3-((4-fluorophenyl)thio)-5-hexylphenyl)trimethylsilane (38.6 mg, 0.107 mmol, 1.0 equiv.), *N*-iodosuccinimide (NIS) (48.2 mg, 0.214 mmol, 2.0 equiv.), *p*-toluenesulfonic acid monohydrate (*p*-TsOH·H_2O) (40.7 mg, 0.214 mmol, 2.0 equiv.) and anhydrous $CHCl_3$ (1.0 mL). The solution was stirred for 40 min at room temperature. The reaction mixture was quenched by addition of aqueous solution of $Na_2S_2O_3$ (sat.). The organic layer was separated and the aqueous layer was extracted three times with DCM. The combined organic layers were dried over $MgSO_4$ and filtered. Solvents were removed under reduced pressure and the product was obtained after purification by preparative TLC (*n*-pentane) as a dark green liquid (23.7 mg, 0.057 mmol, 53%).

***R*$_f$** = 0.60 (*n*-pentane). **^{1}H NMR** (600 MHz, $CDCl_3$): δ 7.41 – 7.36 (m, 2H), 7.36 (s, 1H), 7.34 (dd, *J* = 1.6, 1.6 Hz, 1H), 7.07 – 7.04 (m, 2H), 7.00 (s, 1H), 2.48 – 2.45 (m, 2H), 1.55 – 1.50 (m, 2H), 1.30 – 1.23 (m, 6H), 0.89 – 0.85 (m, 3H). **^{13}C{^{1}H} NMR** (151 MHz, $CDCl_3$): δ 162.8 (d, *J* = 248.2 Hz), 146.2, 139.0, 135.9, 135.1, 134.8 (d, *J* = 8.4 Hz), 132.7, 129.3 (d, *J* = 3.2 Hz), 128.9, 116.8 (d, *J* = 21.8 Hz), 35.5, 31.7, 31.2, 28.9, 22.7, 14.2. **^{19}F NMR** (564 MHz, $CDCl_3$): δ -113.10 – -113.14 (m, 1F). **IR** (neat, cm^{-1}): 2924, 2855, 1730, 1586, 1487, 1397, 1227, 1003, 827. **HRMS** (ESI) calculated for $C_{18}H_{20}FIS$: 414.0309 [M]$^+$, found: 414.0309.

5. Mechanistic Studies

5.1 TEMPO experiment

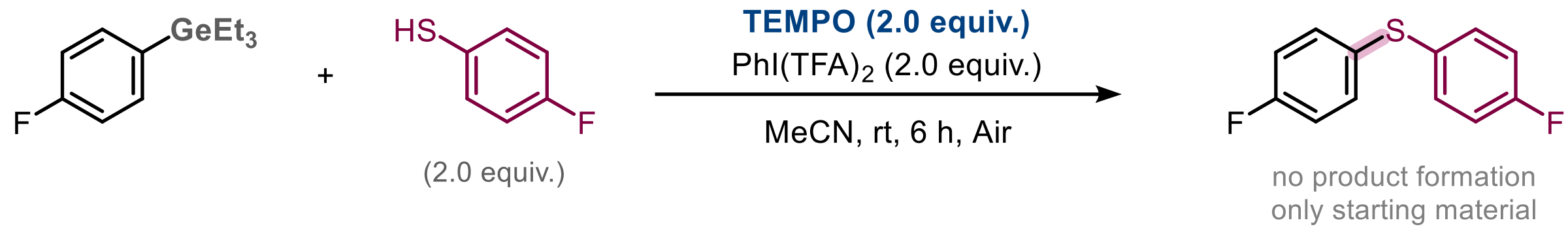

Under air, a 4 mL screw-capped glass vial equipped with a magnetic stir bar was charged with triethyl(4-fluorophenyl)germane (25.5 mg, 0.1 mmol, 1.0 equiv.), 4-fluorobenzenethiol (21.3 µL, 0.2 mmol, 2.0 equiv.), TEMPO (31.2 mg, 0.2 mmol, 2.0 equiv.) and dissolved in MeCN (1.0 M). $PhI(TFA)_2$ (86.0 mg, 0.2 mmol, 2.0 equiv.) was added to the solution. The reaction solution was stirred for 6 h at room temperature and analyzed via GC-MS.

5.2 1,1-diphenylethylene experiment

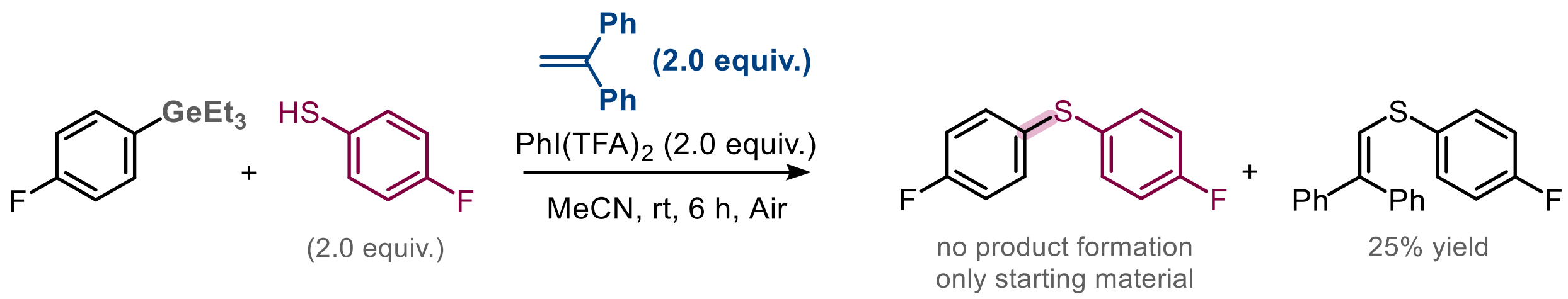

Under air, a 4 mL screw-capped glass vial equipped with a magnetic stir bar was charged with triethyl(4-fluorophenyl)germane (25.5 mg, 0.1 mmol, 1.0 equiv.), 4-fluorobenzenethiol (21.3 µL, 0.2 mmol, 2.0 equiv.), 1,1-diphenylethylene (33.3 µL, 0.2 mmol, 2.0 equiv.) and dissolved in MeCN (1.0 M). $PhI(TFA)_2$ (86.0 mg, 0.2 mmol, 2.0 equiv.) was added to the solution. The reaction solution was stirred for 6 h at room temperature and analyzed via GC-MS. Radical trap adduct product was obtained after purification by column chromatography (*n*-pentane) as a colorless liquid (15.4 mg, 0.050 mmol, 25%).

(2,2-diphenylvinyl)(4-fluorophenyl)sulfane

R$_f$ = 0.15 (*n*-pentane). **^{1}H NMR** (600 MHz, $CDCl_3$) δ 7.46 – 7.40 (m, 4H), 7.38 – 7.33 (m, 3H), 7.31 – 7.22 (m, 5H), 7.08 – 7.01 (m, 2H), 6.76 (s, 1H). **^{13}C{^{1}H} NMR** (151 MHz, $CDCl_3$) δ 162.3 (d, *J* = 247.4 Hz), 141.5, 141.0, 139.2, 132.2 (d, *J* = 8.5 Hz), 131.6 (d, *J* = 3.0 Hz), 129.9, 128.6, 128.5, 128.0, 127.5, 127.3, 124.8, 116.4 (d, *J* = 21.9 Hz). **^{19}F NMR** (564 MHz, $CDCl_3$) δ -114.67 (tt, *J* = 8.5, 5.1 Hz, 1F). **IR** (neat, cm^{-1}): 3057, 3027, 2924, 2853, 2323, 2098, 1955, 1882, 1809, 1750, 1589, 1487, 1441, 1396, 1336, 1225, 1155, 1088, 1014, 939, 908, 822, 754, 730, 694. **HRMS** (ESI) calculated for $C_{20}H_{15}FS$: 306.0873 $[M]^+$, found: 306.0866.

5.3 Diallylether experiment

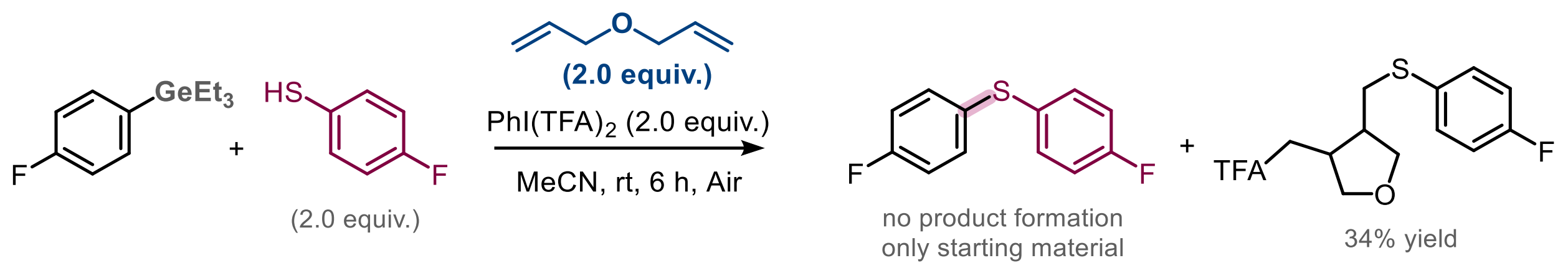

Under air, a 4 mL screw-capped glass vial equipped with a magnetic stir bar was charged with triethyl(4-fluorophenyl)germane (25.5 mg, 0.1 mmol, 1.0 equiv.), 4-fluorobenzenethiol (21.3 µL, 0.2 mmol, 2.0 equiv.), diallylether (24.4 µL, 0.2 mmol, 2.0 equiv.) and dissolved in MeCN (1.0 M). $PhI(TFA)_2$ (86.0 mg, 0.2 mmol, 2.0 equiv.) was added to the solution. The reaction solution was stirred for 6 h at room temperature and analyzed via GC-MS. Radical trap adduct product was obtained after purification by column chromatography (*n*-pentane/EtOAc 98:2) as a colorless liquid (23.2 mg, 0.068 mmol, 34%).

(4-(((4-fluorophenyl)thio)methyl)tetrahydrofuran-3-yl)methyl 2,2,2-trifluoroacetate

R$_f$ = 0.38 (*n*-pentane/EtOAc 95:5). **^{1}H NMR** (400 MHz, $CDCl_3$) δ 7.54 – 7.43 (m, 2H), 7.07 – 6.95 (m, 2H), 5.86 (ddt, *J* = 17.2, 10.4, 5.7 Hz, 1H), 5.34 – 5.14 (m, 2H), 4.53 (ddd, *J* = 44.5, 11.2, 6.2 Hz, 2H), 3.99 (dt, *J* = 5.7, 1.4 Hz, 2H), 3.73 – 3.53 (m, 2H), 3.42 (ddt, *J* = 6.7, 5.7, 3.4 Hz, 1H). **^{13}C{^{1}H} NMR** (151 MHz, $CDCl_3$) δ 163.1 (d, *J* = 249.1 Hz), 157.3 (q, *J* = 42.7 Hz), 135.8 (d, *J* = 8.5 Hz), 134.1, 127.8 (d, *J* = 3.5 Hz), 117.8, 116.5 (d, *J* = 22.2 Hz), 114.6 (q, *J* = 285.7 Hz), 72.4, 68.9, 66.9, 47.7. **^{19}F NMR** (376 MHz, $CDCl_3$) δ -74.94, -112.68 (tt, *J* = 8.9, 5.1 Hz). **IR** (neat, cm^{-1}): 3084, 2861, 2322, 2099, 1995, 1786, 1646, 1589, 1489, 1395, 1347, 1221, 1148, 995, 929, 831, 773, 731. **HRMS** (ESI) calculated for $C_{14}H_{14}F_4O_3S$: 338.0600 $[M]^+$, not found. Fragmentations belonging to the parent molecule were found instead. $C_9H_{10}FOS$: 185.0431 $[M]^+$, found: 185.0429. C_8H_6FS: 153.0169 $[M]^+$, found: 153.0167. C_7H_6FS: 141.0168 $[M]^+$, found: 141.0168.

5.4 Arylthiosuccinimide as sulfur source

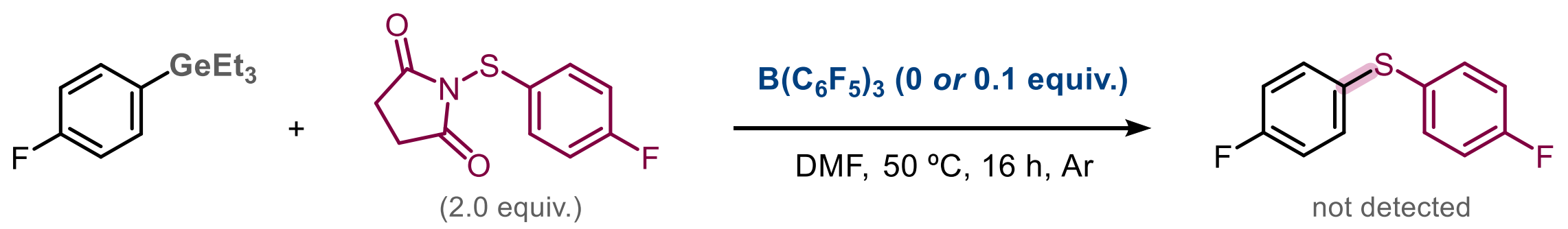

Inside an Ar-filled glovebox, a 4 mL screw-capped glass vial equipped with a magnetic stir bar was charged with triethyl(4-fluorophenyl)germane (15.3 mg, 0.06 mmol, 1.0 equiv.), 4-(fluorophenyl)thiosuccinimide (30.6 mg, 0.12 mmol, 2.0 equiv) and $B(C_6F_5)_3$ (3.1 mg, 0.006 mmol, 0.1 equiv) and dissolved in DMF (0.2 M). The vial was capped and the reaction mixture was stirred for 16 h at 50 °C. It was allowed to cool down to room temperature. Then, the reaction mixture was analyzed by quantitative ^{19}F NMR. No conversion of starting materials was detected.

5.5 Disulfide as sulfur source

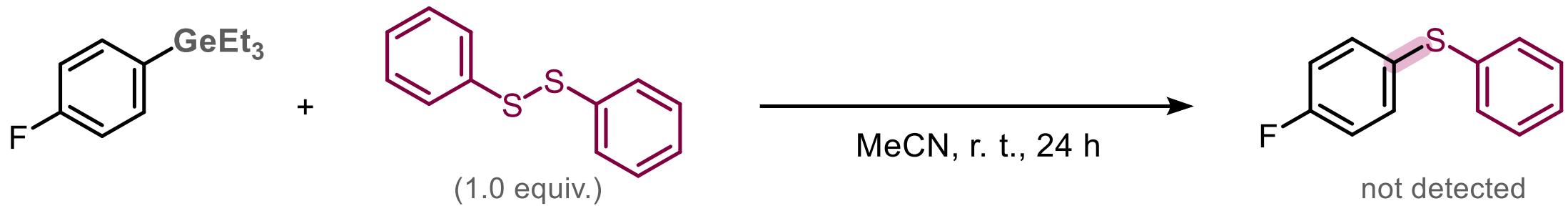

Under air, a 4 mL screw-capped glass vial equipped with a magnetic stir bar was charged with triethyl(4-fluorophenyl)germane (25.5 mg, 0.1 mmol, 1.0 equiv.) and phenyl disulfide (21.8 mg, 0.1 mmol, 1.0 equiv.), and dissolved in MeCN (1.0 M). The reaction solution was stirred for 24 h at room temperature and analyzed via GC-MS. No conversion of starting materials was detected.

5.6 Intermolecular competition experiments

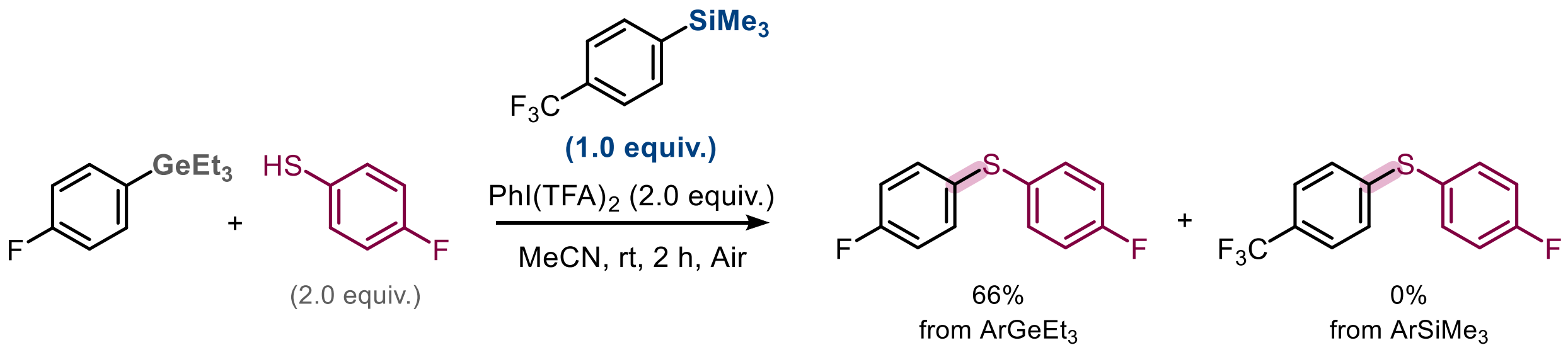

Under air, a 4 mL screw-capped glass vial equipped with a magnetic stir bar was charged with triethyl(4-fluorophenyl)germane (25.5 mg, 0.1 mmol, 1.0 equiv.), 4-fluorobenzenethiol (21.3 μL, 0.2 mmol, 2.0 equiv.), (4-trifluoromethyl)trimethylsilane (21.8 mg, 0.1 mmol, 1.0 equiv.) and dissolved in MeCN (1.0 M). $PhI(TFA)_2$ (86.0 mg, 0.2 mmol, 2.0 equiv.) was added to the solution. The reaction solution was stirred for 2 h at room temperature. The crude mixture was diluted with $CDCl_3$ (0.60 mL) and analyzed by quantitative ^{19}F NMR.

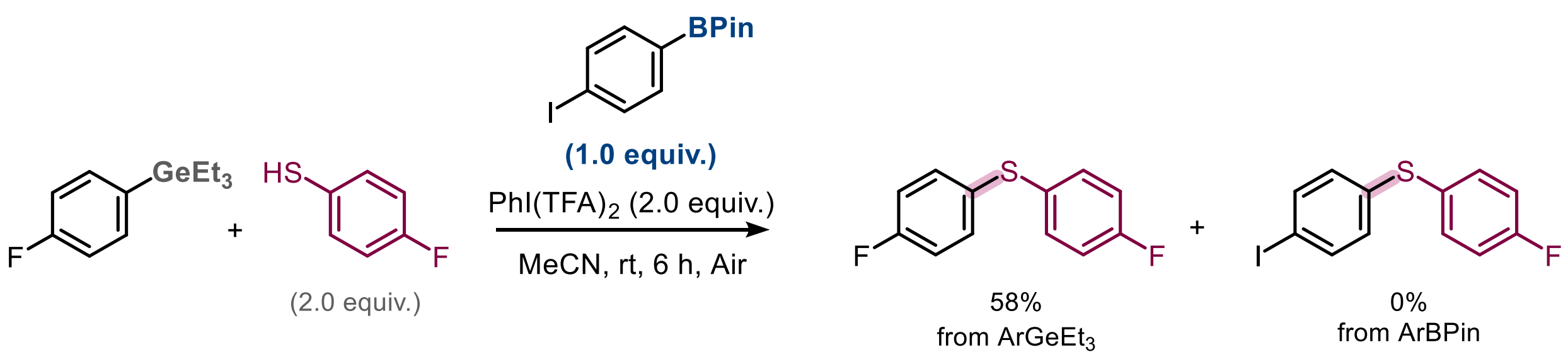

Under air, a 4 mL screw-capped glass vial equipped with a magnetic stir bar was charged with triethyl(4-fluorophenyl)germane (25.5 mg, 0.1 mmol, 1.0 equiv.), 4-fluorobenzenethiol (21.3 μL, 0.2 mmol, 2.0 equiv.), 2-(4-iodophenyl)-4,4,5,5-tetramethyl-1,3,2-dioxaborolane (33.0 mg, 0.1 mmol, 1.0 equiv.) and dissolved in MeCN (1.0 M). $PhI(TFA)_2$ (86.0 mg, 0.2 mmol, 2.0 equiv.) was added to the solution. The reaction solution was stirred for 6 h at room temperature. The crude mixture was diluted with $CDCl_3$ (0.60 mL) and analyzed by quantitative ^{19}F NMR.

5.7 Limitations of scope

The following substrates have been tested under the standard reaction conditions and failed to provide synthetically useful amounts of product.

failed (hetero)aryl germanes

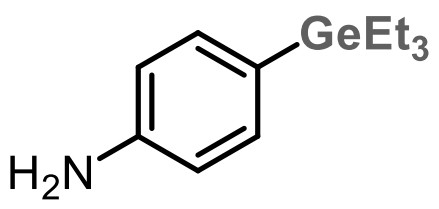

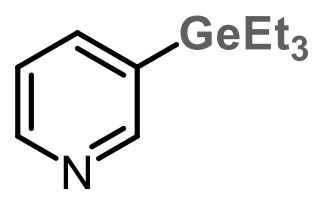

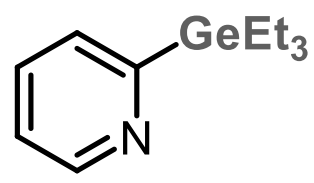

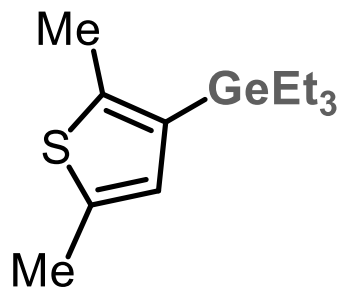

(also with added DBU)

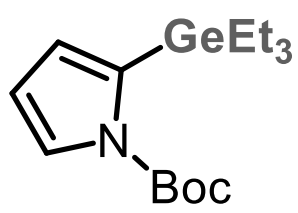

(also with added DBU)

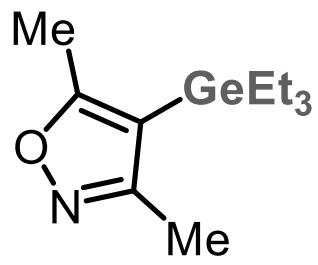

failed thiols

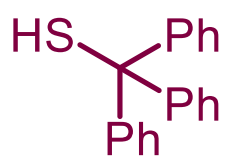

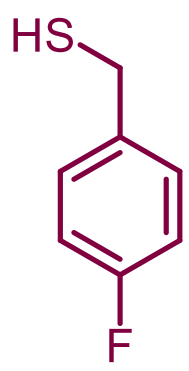

benzylic

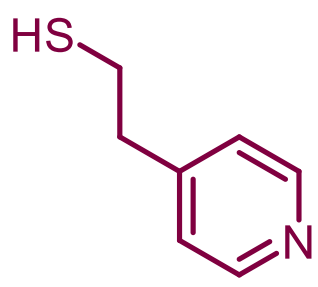

pyridine

6. Computational Analysis

6.1 General details

DFT calculations were carried out using the Gaussian 16 (A.03) program package [17]. Geometry optimizations and frequency analyses were calculated with B3LYP-D3BJ/6-31++G(d,p) (LANL2DZ for I) including solvation through the CPCM implicit solvation model (solvent = acetonitrile). Frequencies were used to determine the nature of the stationary points as minima (no imaginary frequency) or transition states (one imaginary frequency). Intrinsic reaction coordinate (IRC) calculations were performed to confirm that the obtained transition states connect the corresponding reactants and products. Electronic energies were calculated with different functionals (M06-2X, PBE0-D3BJ, MN15L, wB97XD) and def2-TZVP basis set including solvation using CPCM (MeCN). Energies are calculated at the sum of the high-basis set electronic energies plus the thermochemistry corrections at standard conditions (298.15 K and 1 atm). All energies were corrected to 1 M standard state (addition of 1.89 kcal/mol to every species).

6.2 Method comparison

Table S1. Gibbs free energies (in kcal/mol) at the CPCM(MeCN) ***method***/def2-TZVP//CPCM(MeCN) B3LYP-D3(BJ)/6-31++G(d,p) (LANL2DZ for I) level of theory.

method	TS1	II	TS2	III	IV + V
M06-2X	14.1	12.7	21.1	9.3	5.5
MN15L	13.4	12.2	20.2	8.8	6.5
PBE0-D3BJ	11.0	9.6	19.1	12.0	7.1
wB97XD	15.2	13.6	25.2	16.3	11.6

6.3 XYZ coordinates of computed structures and energies

$PhGeMe_3$

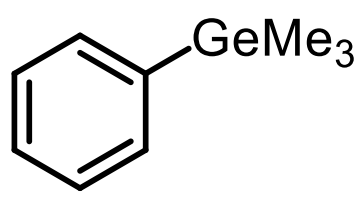

C	1.44907	1.21245	0.00013
C	2.84806	1.20729	0.00014
C	3.54143	-0.00599	0.00008
C	2.82505	-1.20722	0.00002
C	1.42803	-1.18837	-0.00005
C	0.71083	0.01889	0.00002
H	0.92730	2.16603	0.00018
H	3.39301	2.14710	0.00019
H	4.62740	-0.01660	0.00012
H	3.35516	-2.15552	-0.00002
H	0.89122	-2.13458	-0.00011
Ge	-1.22283	-0.00209	0.00006
C	-1.87823	1.83473	0.00090
H	-2.97185	1.84341	0.00296
H	-1.52607	2.36665	0.88957
H	-1.52939	2.36612	-0.88939
C	-1.82738	-0.92788	1.61296
H	-2.86809	-1.24805	1.50654
H	-1.20670	-1.80621	1.81248
H	-1.75533	-0.26122	2.47743
C	-1.82668	-0.92589	-1.61429
H	-2.86765	-1.24569	-1.50934
H	-1.75340	-0.25823	-2.47789
H	-1.20622	-1.80428	-1.81424

Zero-point correction = 0.201192 (Hartree/Particle)
Thermal correction to Energy = 0.213780
Thermal correction to Enthalpy = 0.214724
Thermal correction to Gibbs Free Energy = 0.162331
Sum of electronic and zero-point Energies = -2426.335577
Sum of electronic and thermal Energies = -2426.322989
Sum of electronic and thermal Enthalpies = -2426.322045
Sum of electronic and thermal Free Energies = -2426.374438
E(M06-2X) = -2428.38381156
E(MN15L) = -2428.23498630
E(PBE0-D3BJ) = -2427.87111031
E(wB97XD) = -2428.49789751

PhS radical (I)

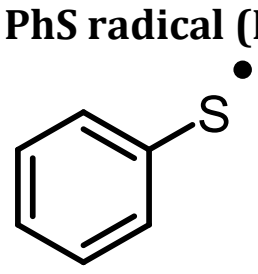

S	-0.75752	-1.97206	-0.00118
C	-2.29860	-1.19725	-0.00160
C	-2.94779	-0.86964	1.22191
C	-2.94709	-0.86954	-1.22544
C	-4.18800	-0.24507	1.21572

H	-2.45715	-1.11635	2.15731
C	-4.18730	-0.24498	-1.21991
H	-2.45591	-1.11618	-2.16059
C	-4.81178	0.06868	-0.00227
H	-4.67567	0.00047	2.15364
H	-4.67443	0.00064	-2.15809
H	-5.78140	0.55643	-0.00253

Zero-point correction = 0.090554 (Hartree/Particle)
Thermal correction to Energy = 0.096139
Thermal correction to Enthalpy = 0.097083
Thermal correction to Gibbs Free Energy = 0.060064
Sum of electronic and zero-point Energies = -629.757641
Sum of electronic and thermal Energies = -629.752057
Sum of electronic and thermal Enthalpies = -629.751113
Sum of electronic and thermal Free Energies = -629.788132
E(M06-2X) = -629.784436499
E(MN15L) = -629.654092156
E(PBE0-D3BJ) = -629.498219127
E(wB97XD) = -629.816368173

TS1

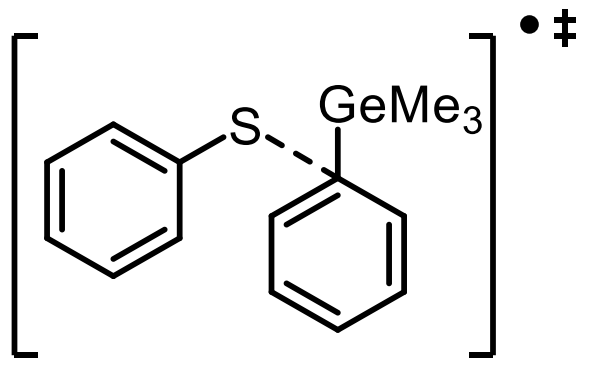

C	0.11732	0.98205	-1.23388
C	-0.65414	2.12240	-1.21976
C	-1.06434	2.70626	-0.00007
C	-0.65410	2.12250	1.21965
C	0.11736	0.98215	1.23384
C	0.50889	0.29297	0.00000
H	0.41451	0.54218	-2.18118
H	-0.94700	2.58205	-2.15993
H	-1.68200	3.59824	-0.00009
H	-0.94692	2.58223	2.15980
H	0.41458	0.54235	2.18117
Ge	2.46209	-0.22517	-0.00001
C	2.84065	-1.26551	-1.62126
H	3.90510	-1.51796	-1.64382
H	2.25721	-2.18936	-1.63122
H	2.60081	-0.68470	-2.51598
C	2.84079	-1.26493	1.62158
H	3.90510	-1.51800	1.64378
H	2.60171	-0.68353	2.51612
H	2.25681	-2.18843	1.63228
C	3.45012	1.47135	-0.00036
H	4.52675	1.27741	-0.00033
H	3.19396	2.05335	-0.88998
H	3.19399	2.05369	0.88906
S	-0.39038	-1.52469	0.00012
C	-2.09484	-1.01721	0.00006
C	-2.77088	-0.80465	1.21277
C	-2.77085	-0.80485	-1.21270
C	-4.10428	-0.38929	1.21077
H	-2.24701	-0.96281	2.14945
C	-4.10425	-0.38949	-1.21081
H	-2.24696	-0.96316	-2.14935
C	-4.77299	-0.17899	-0.00004
H	-4.61932	-0.22778	2.15319
H	-4.61927	-0.22814	-2.15326
H	-5.80957	0.14443	-0.00008

Zero-point correction = 0.290187 (Hartree/Particle)
Thermal correction to Energy = 0.309793
Thermal correction to Enthalpy = 0.310738
Thermal correction to Gibbs Free Energy = 0.239988
Sum of electronic and zero-point Energies = -3055.785715
Sum of electronic and thermal Energies = -3055.766108
Sum of electronic and thermal Enthalpies = -3055.765164
Sum of electronic and thermal Free Energies = -3055.835914
E(M06-2X) = -3058.16040609
E(MN15L) = -3057.88228562
E(PBE0-D3BJ) = -3057.36632744
E(wB97XD) = -3058.30467252

Wheland_radical (II)

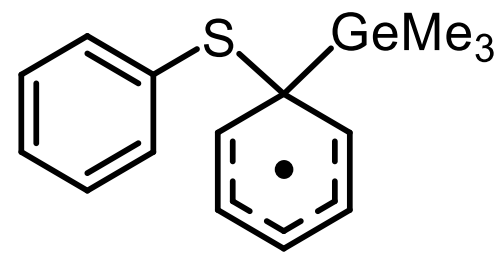

C	0.04238	0.98058	-1.23745
C	-0.62686	2.18168	-1.22051
C	-0.98067	2.80395	-0.00015
C	-0.62658	2.18214	1.22036
C	0.04269	0.98105	1.23759
C	0.40330	0.26555	0.00016
H	0.29335	0.51286	-2.18473
H	-0.88648	2.66081	-2.16092
H	-1.51734	3.74671	-0.00026
H	-0.88599	2.66161	2.16065
H	0.29388	0.51368	2.18499
Ge	2.37912	-0.20595	-0.00004
C	2.77890	-1.23796	-1.62187
H	3.84847	-1.46781	-1.64494
H	2.21528	-2.17408	-1.63141
H	2.52583	-0.66179	-2.51586
C	2.77940	-1.23797	1.62166
H	3.84923	-1.46656	1.64510
H	2.52529	-0.66236	2.51572
H	2.21687	-2.17475	1.63071
C	3.32154	1.51531	-0.00016
H	4.40273	1.34898	-0.00027
H	3.04997	2.09027	-0.88965
H	3.05015	2.09030	0.88936
S	-0.45917	-1.49941	0.00065
C	-2.17411	-1.01092	0.00033
C	-2.85117	-0.80684	1.21290
C	-2.85108	-0.80795	-1.21247
C	-4.18946	-0.40624	1.21074
H	-2.32595	-0.95990	2.14967
C	-4.18937	-0.40732	-1.21075
H	-2.32581	-0.96186	-2.14907
C	-4.86038	-0.20436	-0.00012
H	-4.70644	-0.25075	2.15306
H	-4.70628	-0.25266	-2.15325
H	-5.90067	0.10687	-0.00030

Zero-point correction = 0.290471 (Hartree/Particle)
Thermal correction to Energy = 0.310755
Thermal correction to Enthalpy = 0.311699
Thermal correction to Gibbs Free Energy = 0.238744
Sum of electronic and zero-point Energies = -3055.785468
Sum of electronic and thermal Energies = -3055.765184
Sum of electronic and thermal Enthalpies = -3055.764239
Sum of electronic and thermal Free Energies = -3055.837195
E(M06-2X) = -3058.16133406
E(MN15L) = -3057.88293510
E(PBE0-D3BJ) = -3057.36736439
E(wB97XD) = -3058.30593182

TS2

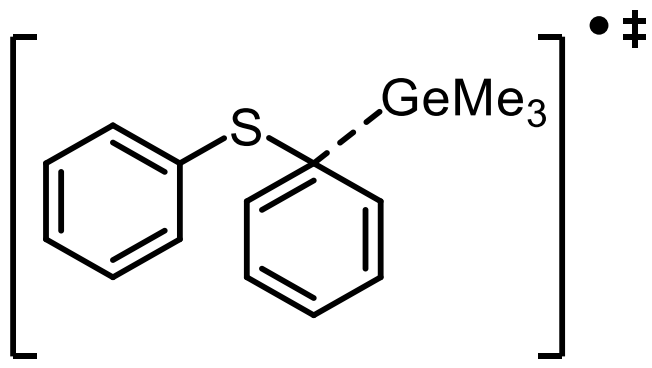

C	-0.17721	1.26385	1.25525
C	-0.52411	2.60613	1.21427
C	-0.71703	3.26223	-0.01484
C	-0.52398	2.55250	-1.21291
C	-0.17316	1.20968	-1.19416
C	-0.08202	0.50430	0.04693
H	-0.01315	0.76328	2.20405
H	-0.63065	3.15748	2.14419
H	-0.98470	4.31344	-0.03831
H	-0.62849	3.06194	-2.16670
H	-0.00122	0.67034	-2.11967
Ge	-2.41613	-0.72625	-0.00316
C	-2.69970	-2.21211	1.27228

H	-3.75371	-2.51333	1.24503
H	-2.07499	-3.06733	1.00298
H	-2.44951	-1.89059	2.28670
C	-2.67100	-1.30105	-1.87468
H	-3.71411	-1.60263	-2.02339
H	-2.44189	-0.47581	-2.55405
H	-2.01851	-2.14699	-2.10524
C	-3.57436	0.80888	0.42474
H	-4.62104	0.52321	0.27083
H	-3.43509	1.11014	1.46568
H	-3.33219	1.65361	-0.22404
S	0.76760	-1.08313	0.08237
C	2.50991	-0.63567	0.03326
C	2.98082	0.68439	0.02798
C	3.42782	-1.69867	0.00335
C	4.35742	0.93145	-0.00663
H	2.28709	1.51569	0.05118
C	4.79911	-1.44143	-0.02932
H	3.07010	-2.72492	0.00561
C	5.27238	-0.12417	-0.03513
H	4.71061	1.95867	-0.01080
H	5.49770	-2.27279	-0.05177
H	6.33915	0.07492	-0.06158

Zero-point correction = 0.289939 (Hartree/Particle)
Thermal correction to Energy = 0.309936
Thermal correction to Enthalpy = 0.310880
Thermal correction to Gibbs Free Energy = 0.237673
Sum of electronic and zero-point Energies = -3055.769058
Sum of electronic and thermal Energies = -3055.749061
Sum of electronic and thermal Enthalpies = -3055.748117
Sum of electronic and thermal Free Energies = -3055.821324
E(M06-2X) = -3058.14686081
E(MN15L) = -3057.86915909
E(PBE0-D3BJ) = -3057.35120670
E(wB97XD) = -3058.28633560

IRC_TS2 (III)

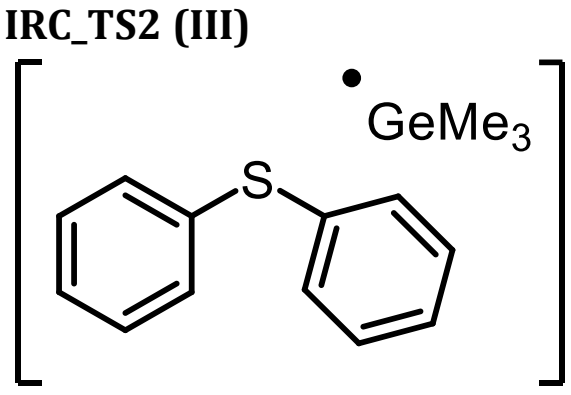

C	-0.00342	1.58947	1.33886
C	-0.54875	2.87242	1.24166
C	-0.73015	3.46433	-0.01158
C	-0.37217	2.76834	-1.17133
C	0.16791	1.48393	-1.07951
C	0.35764	0.89524	0.17772
H	0.14425	1.12616	2.30862
H	-0.83009	3.40683	2.14372
H	-1.15451	4.46075	-0.08575
H	-0.51728	3.22211	-2.14686
H	0.44702	0.94017	-1.97599
Ge	-2.69191	-1.00625	0.29211
C	-3.96834	-2.36015	0.98291
H	-4.86208	-2.35518	0.34655
H	-3.52296	-3.35830	0.95842
H	-4.26379	-2.12482	2.00884
C	-2.21950	-1.46447	-1.57726
H	-3.14063	-1.49743	-2.17212
H	-1.55048	-0.71050	-1.99730
H	-1.73151	-2.44140	-1.62378
C	-3.54806	0.77888	0.32162
H	-4.44343	0.74577	-0.31138
H	-3.84261	1.04857	1.33922
H	-2.86298	1.53646	-0.06378
S	1.03413	-0.75813	0.30434
C	2.78581	-0.47860	0.03141
C	3.38805	0.78480	0.09646
C	3.57382	-1.61171	-0.22585
C	4.76685	0.90821	-0.09991
H	2.79068	1.66677	0.29669
C	4.95112	-1.47889	-0.41103
H	3.11102	-2.59280	-0.28502
C	5.55519	-0.21813	-0.35217
H	5.22290	1.89277	-0.05094
H	5.54999	-2.36270	-0.61013
H	6.62528	-0.11572	-0.50263

Zero-point correction = 0.291233 (Hartree/Particle)
Thermal correction to Energy = 0.312107
Thermal correction to Enthalpy = 0.313051
Thermal correction to Gibbs Free Energy = 0.234901
Sum of electronic and zero-point Energies = -3055.776863
Sum of electronic and thermal Energies = -3055.755989
Sum of electronic and thermal Enthalpies = -3055.755045
Sum of electronic and thermal Free Energies = -3055.833196
E(M06-2X) = -3058.16299431
E(MN15L) = -3057.88460174
E(PBE0-D3BJ) = -3057.35972990
E(wB97XD) = -3058.29774629

$GeMe_3$ radical (IV)

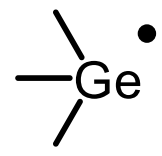

Ge	0.00009	-0.00002	-0.30307
C	1.85622	-0.07617	0.31760
H	1.87804	-0.07771	1.41448
H	2.34277	-0.98610	-0.04381
H	2.41551	0.79140	-0.04278
C	-0.99423	-1.56920	0.31756
H	-1.00666	-1.58772	1.41441
H	-2.02545	-1.53515	-0.04411
H	-0.52280	-2.48732	-0.04320
C	-0.86221	1.64544	0.31753
H	-0.87404	1.66444	1.41439
H	-0.31659	2.52185	-0.04241
H	-1.89245	1.69653	-0.04490

Zero-point correction = 0.107631 (Hartree/Particle)
Thermal correction to Energy = 0.115632
Thermal correction to Enthalpy = 0.116576
Thermal correction to Gibbs Free Energy = 0.074856
Sum of electronic and zero-point Energies = -2194.663542
Sum of electronic and thermal Energies = -2194.655541
Sum of electronic and thermal Enthalpies = -2194.654597
Sum of electronic and thermal Free Energies = -2194.696317
E(M06-2X) = -2196.69952353
E(MN15L) = -2196.64966170
E(PBE0-D3BJ) = -2196.35532985
E(wB97XD) = -2196.79037497

PhSPh (V)

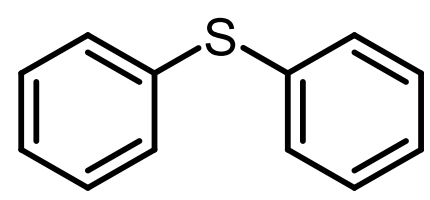

S	-0.69269	-2.98102	-0.32169
C	-2.04342	-1.83442	-0.06115
C	-2.32980	-1.37724	1.23150
C	-2.82996	-1.41939	-1.14439
C	-3.39900	-0.50067	1.43792
H	-1.71610	-1.70239	2.06517
C	-3.89412	-0.53953	-0.93326
H	-2.60430	-1.78026	-2.14257
C	-4.17979	-0.08023	0.35702
H	-3.61884	-0.14703	2.44054
H	-4.50050	-0.21686	-1.77400
H	-5.00922	0.60126	0.51907
C	0.65975	-1.90377	-0.79750
C	1.84869	-2.52828	-1.20774
C	0.58613	-0.50554	-0.76291
C	2.95182	-1.75704	-1.57671
C	1.69627	0.25745	-1.13884
C	2.88204	-0.35965	-1.54587
H	1.91027	-3.61240	-1.24066
H	-0.32531	-0.01149	-0.44719
H	3.86562	-2.25153	-1.89267
H	1.62788	1.34106	-1.10976
H	3.74001	0.23846	-1.83617

Zero-point correction = 0.182227 (Hartree/Particle)
Thermal correction to Energy = 0.192989
Thermal correction to Enthalpy = 0.193934

Thermal correction to Gibbs Free Energy = 0.141928
Sum of electronic and zero-point Energies = -861.381874
Sum of electronic and thermal Energies = -861.371112
Sum of electronic and thermal Enthalpies = -861.370168
Sum of electronic and thermal Free Energies = -861.422174

E(M06-2X) = -861.454295647
E(MN15L) = -861.223392509
E(PBE0-D3BJ) = -860.997116097
E(wB97XD) = -861.499865345

7. NMR Spectra

Bis(4-fluorophenyl)sulfane (1)

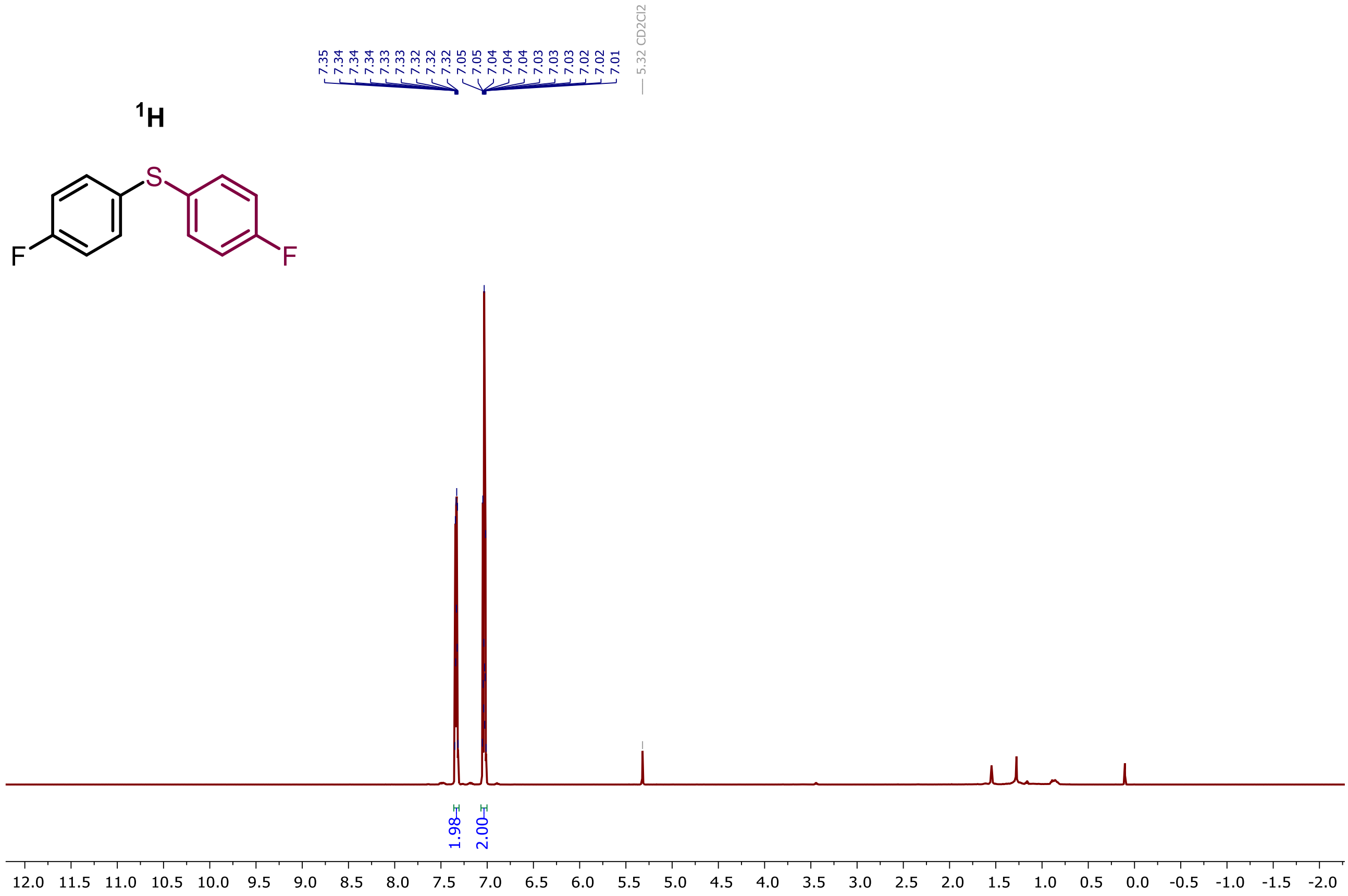

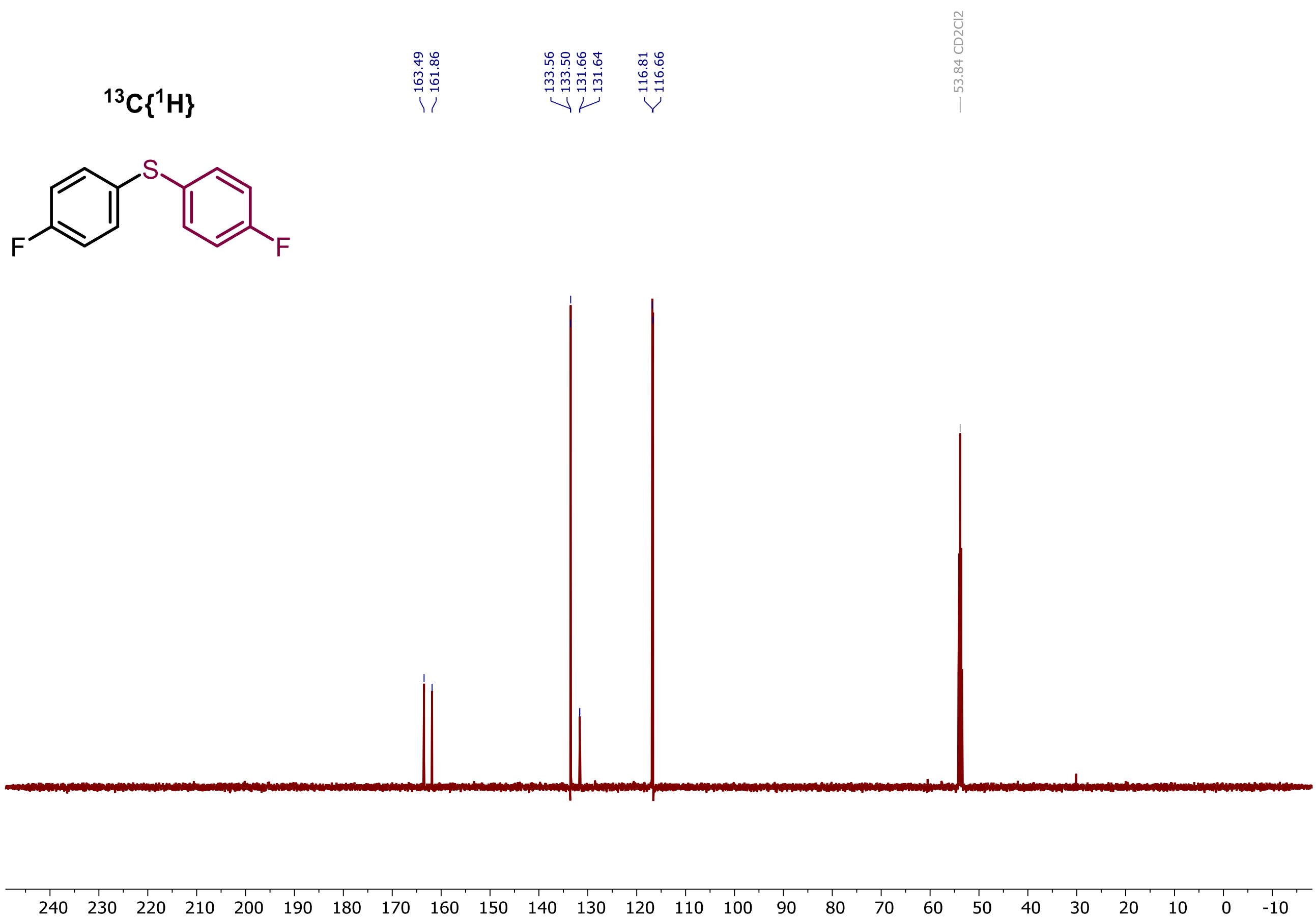

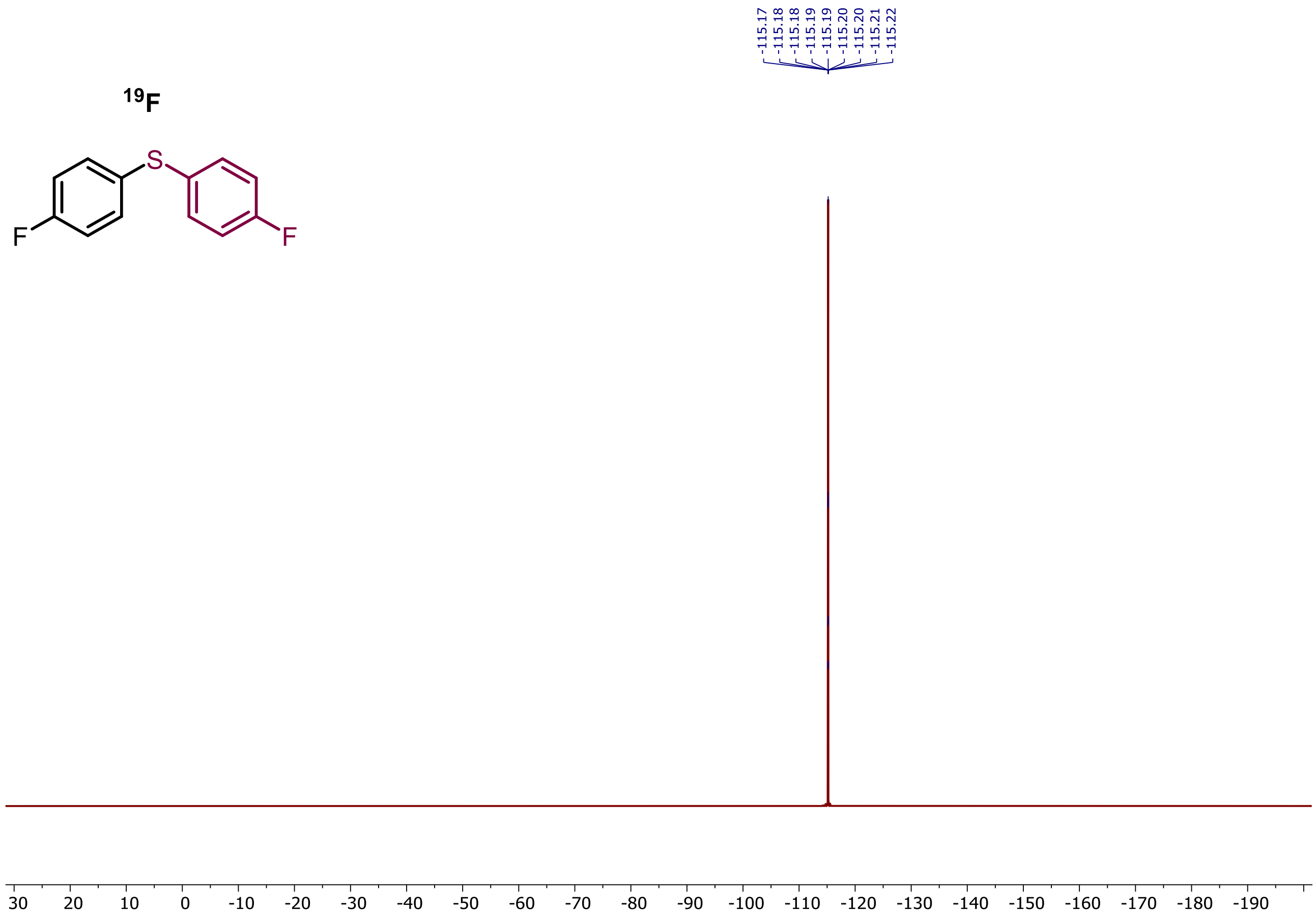

4-Fluorophenyl)(4-methoxyphenyl)sulfane (2)

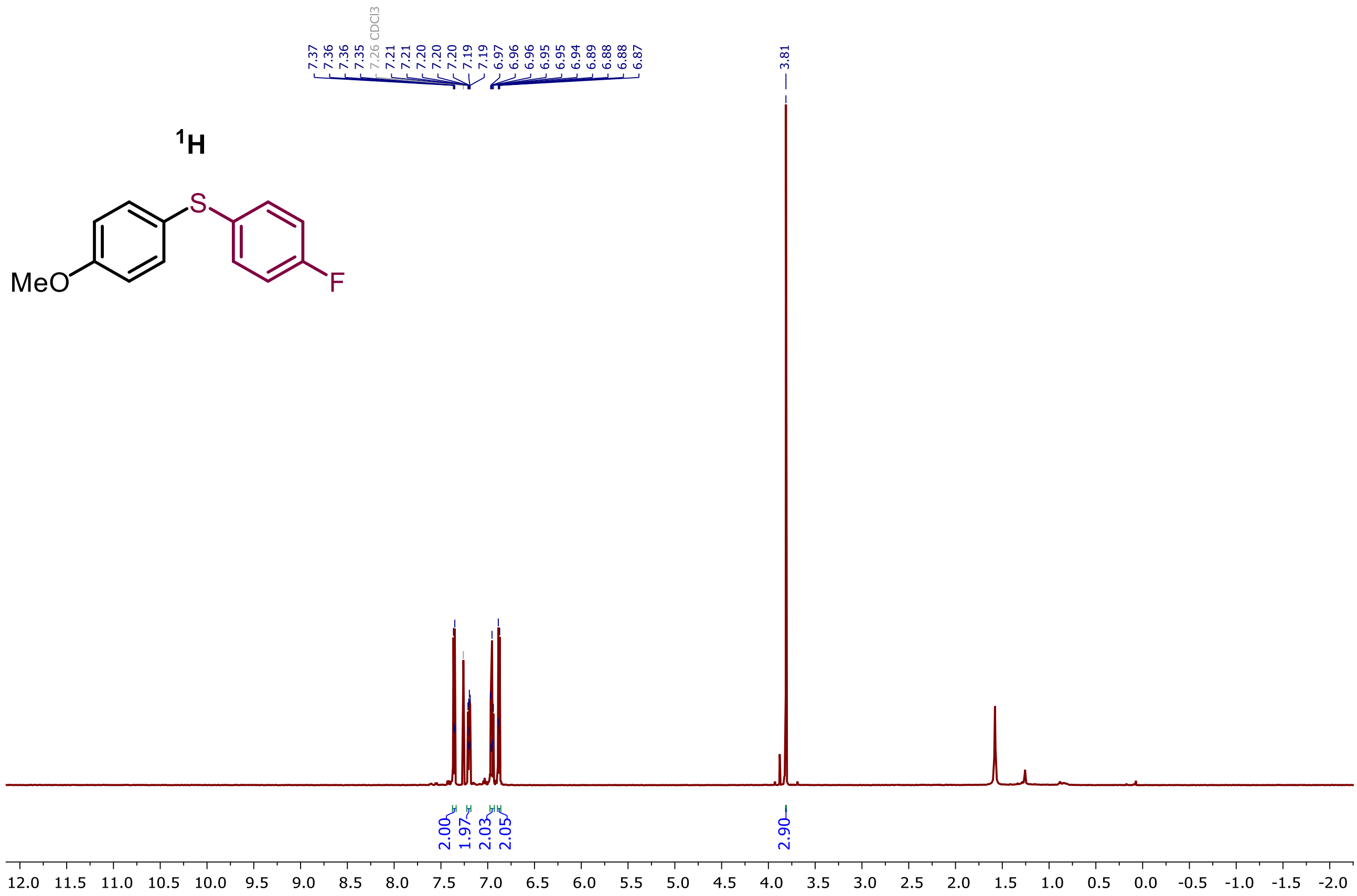

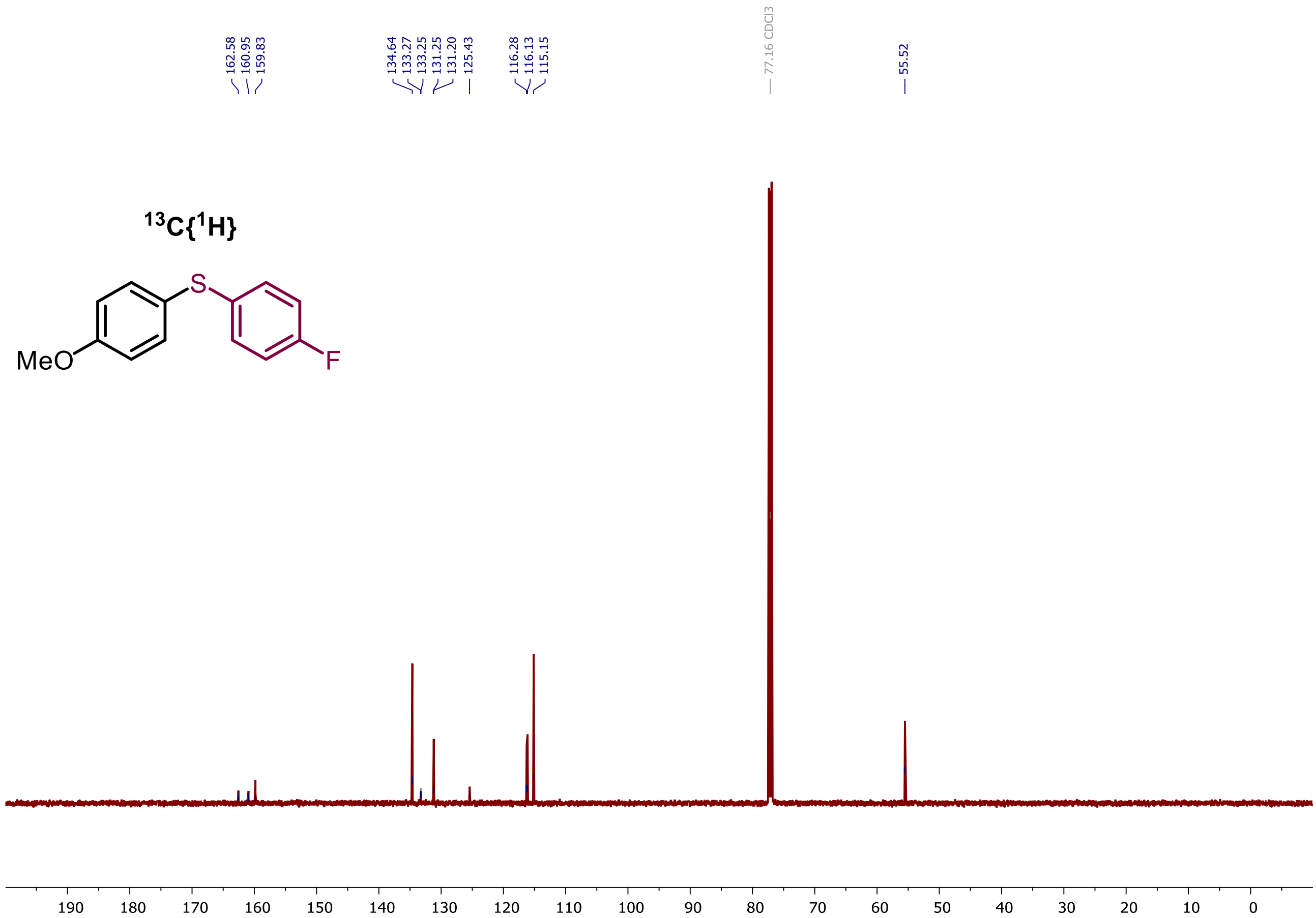
13C{1H}
MeO
S
F
162.58
160.95
159.83
134.64
133.27
133.25
131.25
131.20
125.43
116.28
116.13
115.15
77.16 CDCl3
55.52
190 180 170 160 150 140 130 120 110 100 90 80 70 60 50 40 30 20 10 0

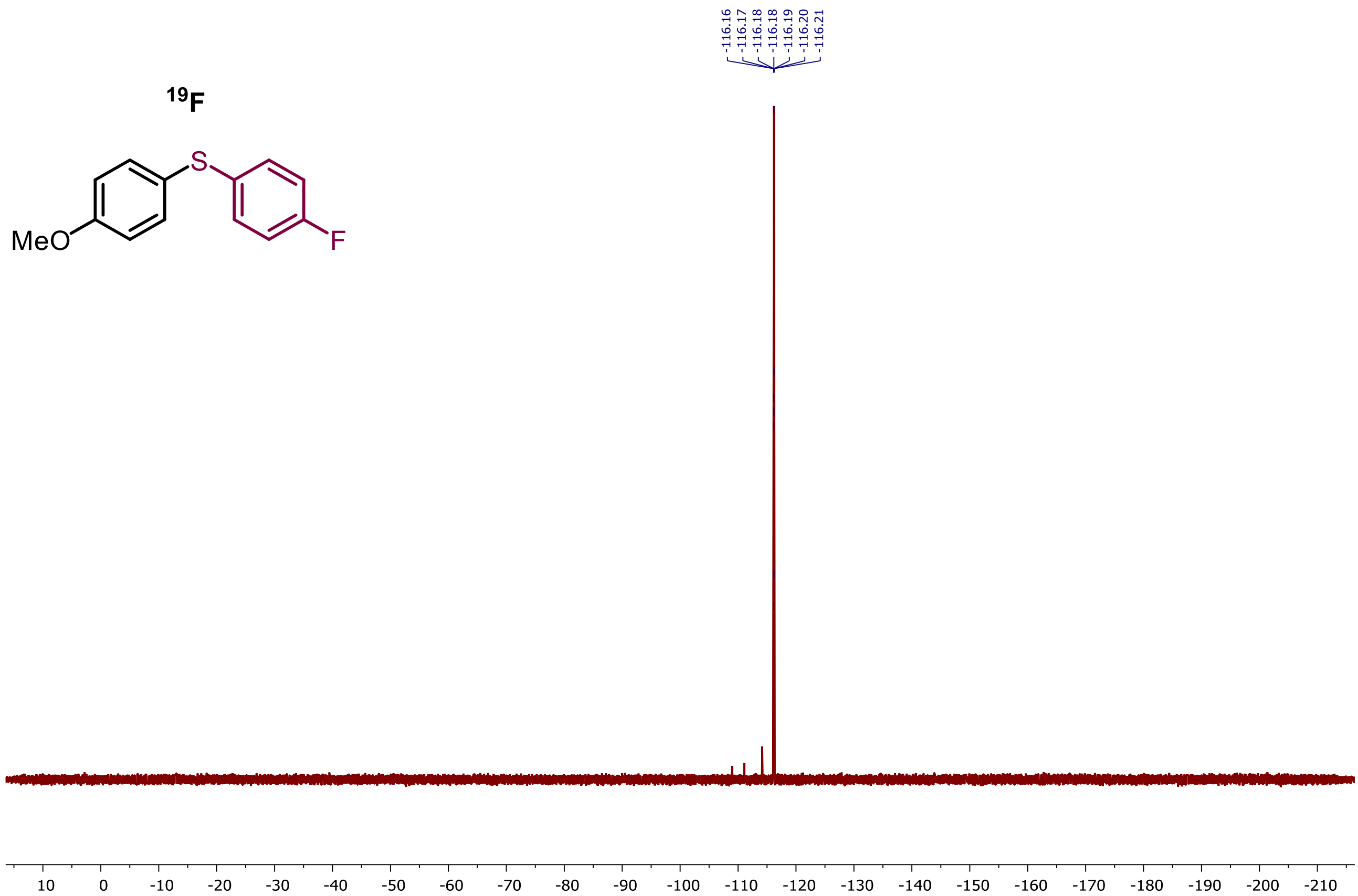
19F
MeO
S
F
-116.16
-116.17
-116.18
-116.19
-116.20
-116.21
10 0 -10 -20 -30 -40 -50 -60 -70 -80 -90 -100 -110 -120 -130 -140 -150 -160 -170 -180 -190 -200 -210

(4-Chlorophenyl)(4-fluorophenyl)sulfane (3)

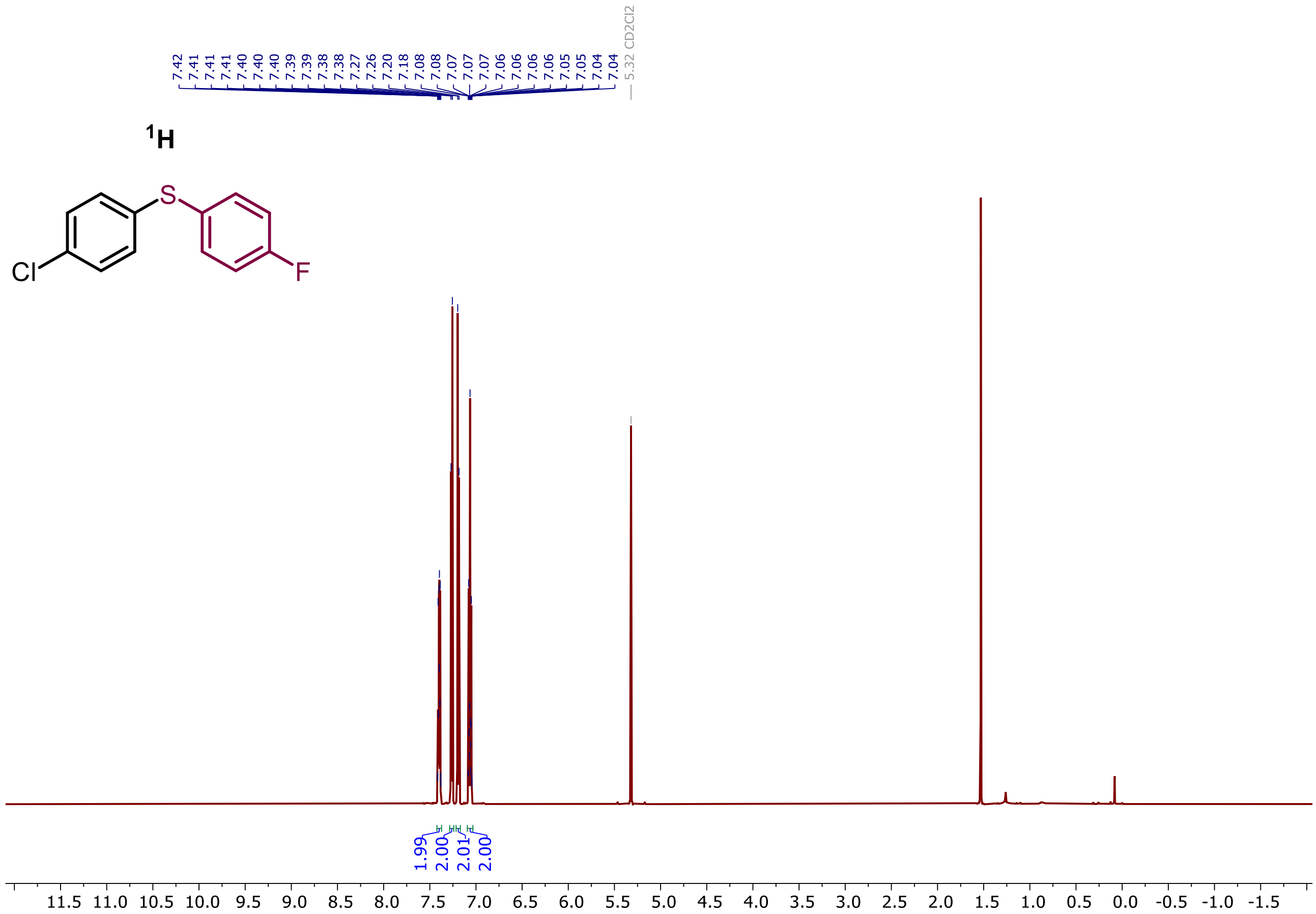

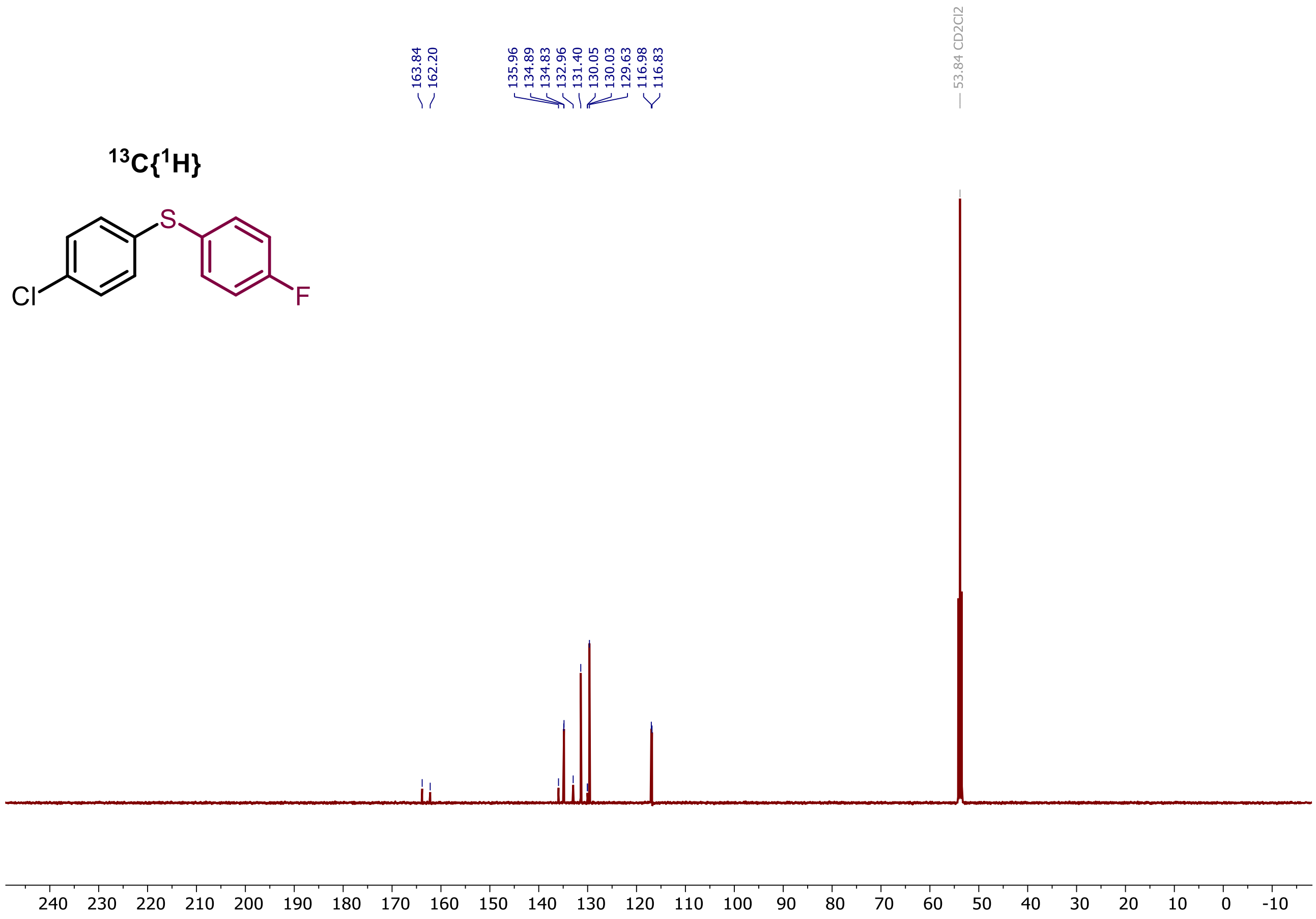

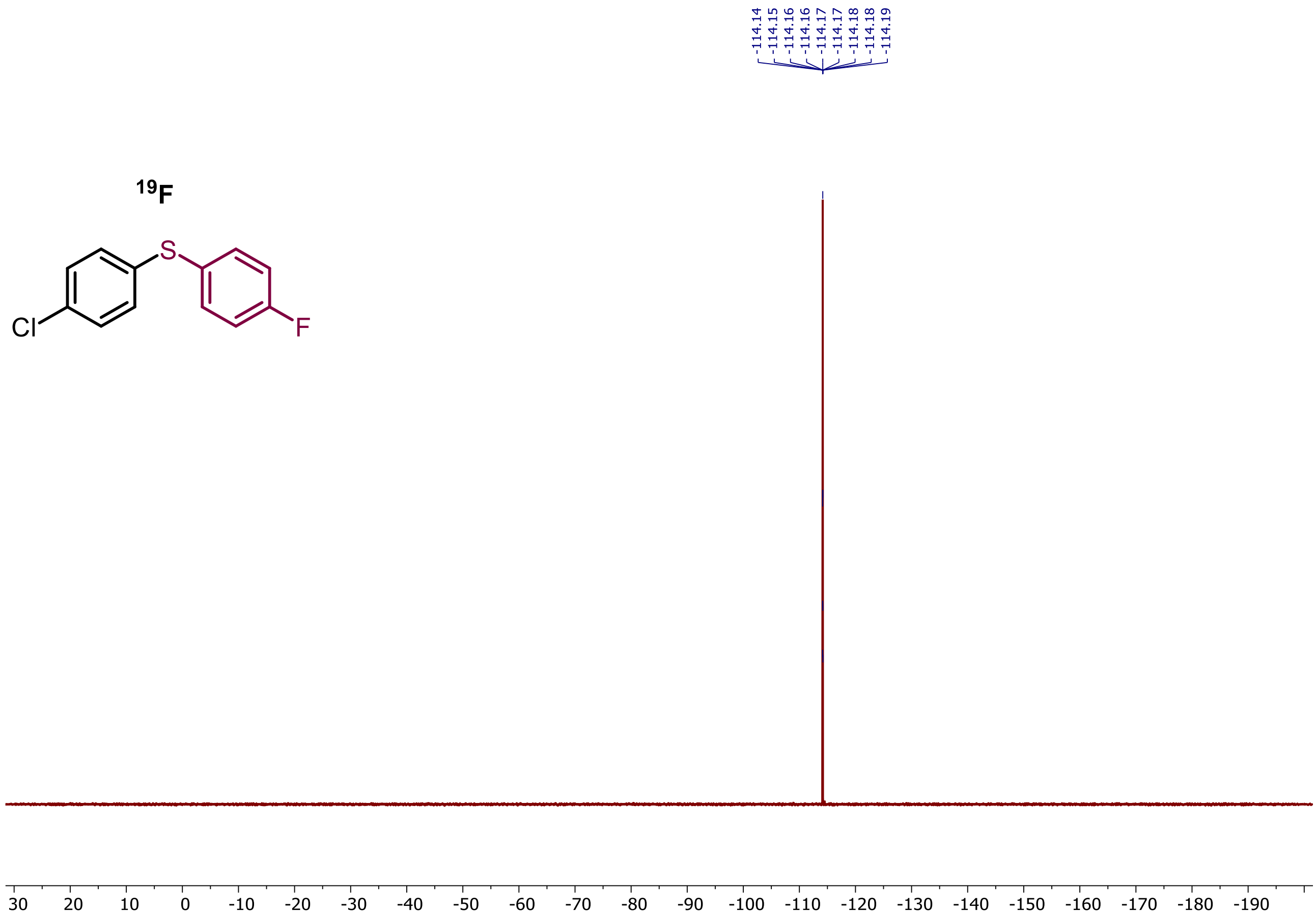

(4-Fluorophenyl)(4-iodophenyl)sulfane (4)

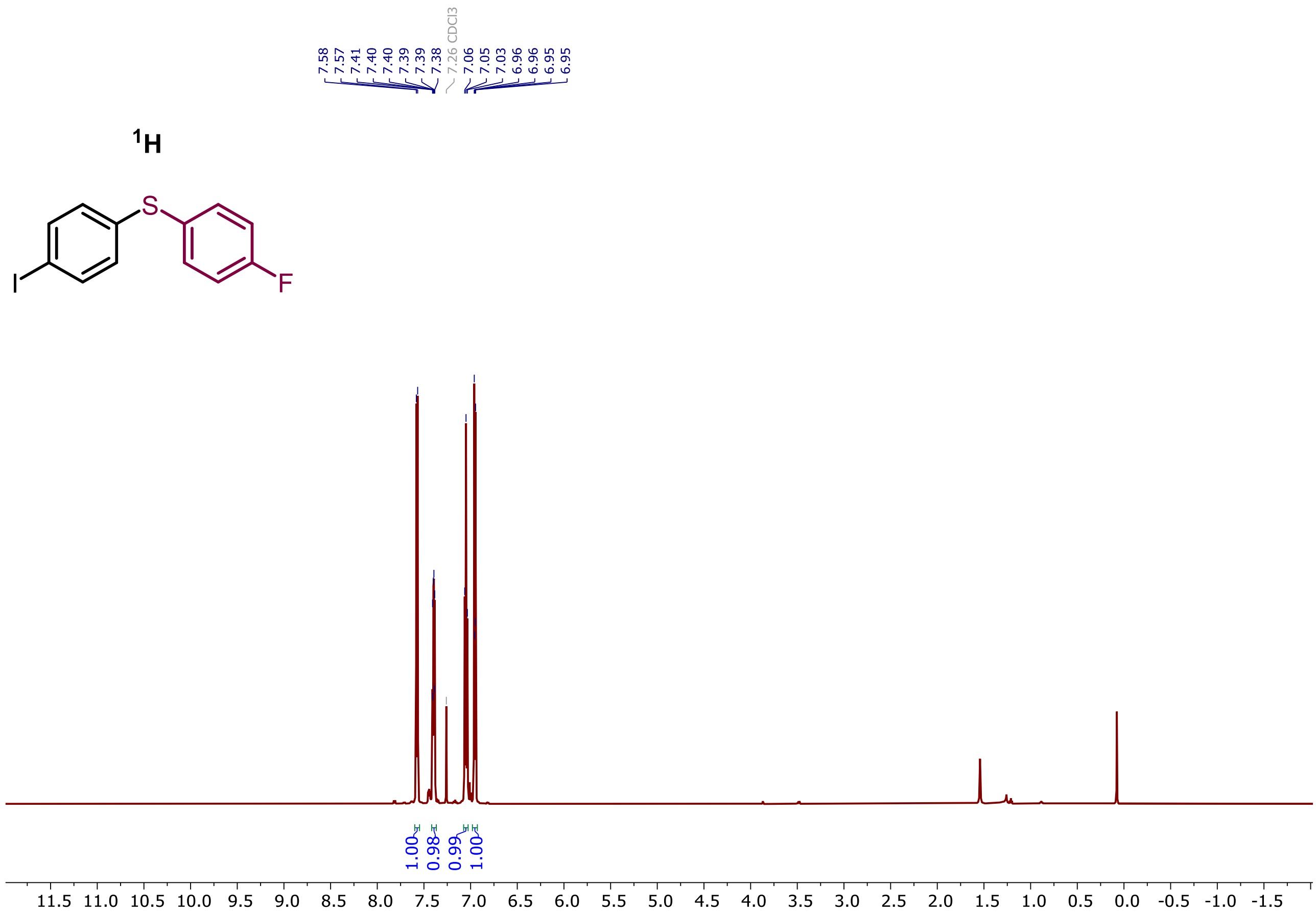

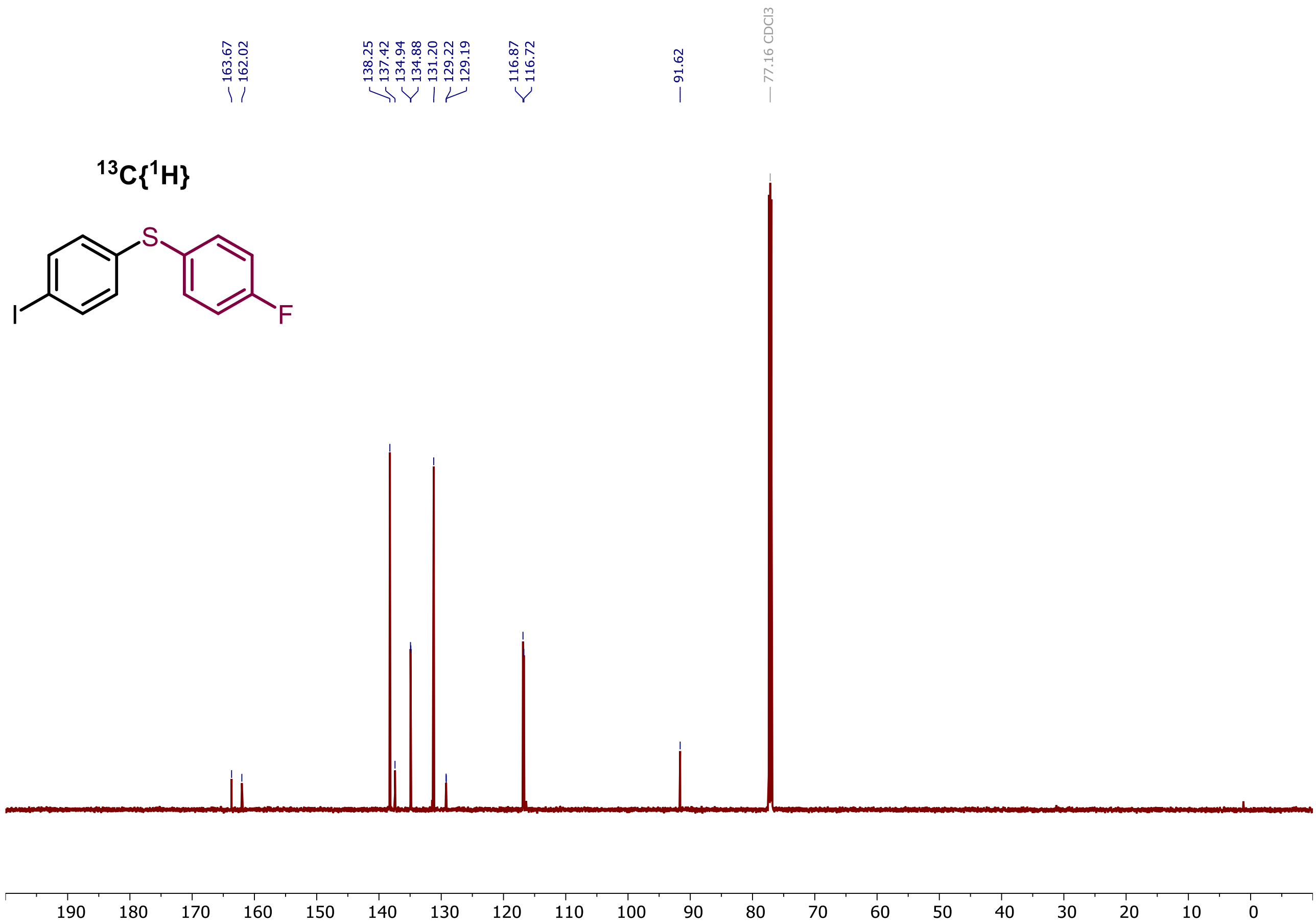
163.67
162.02
138.25
137.42
134.94
134.88
131.20
129.22
129.19
116.87
116.72
91.62
77.16 CDCl3
13C{1H}
S
I
F
190 180 170 160 150 140 130 120 110 100 90 80 70 60 50 40 30 20 10 0

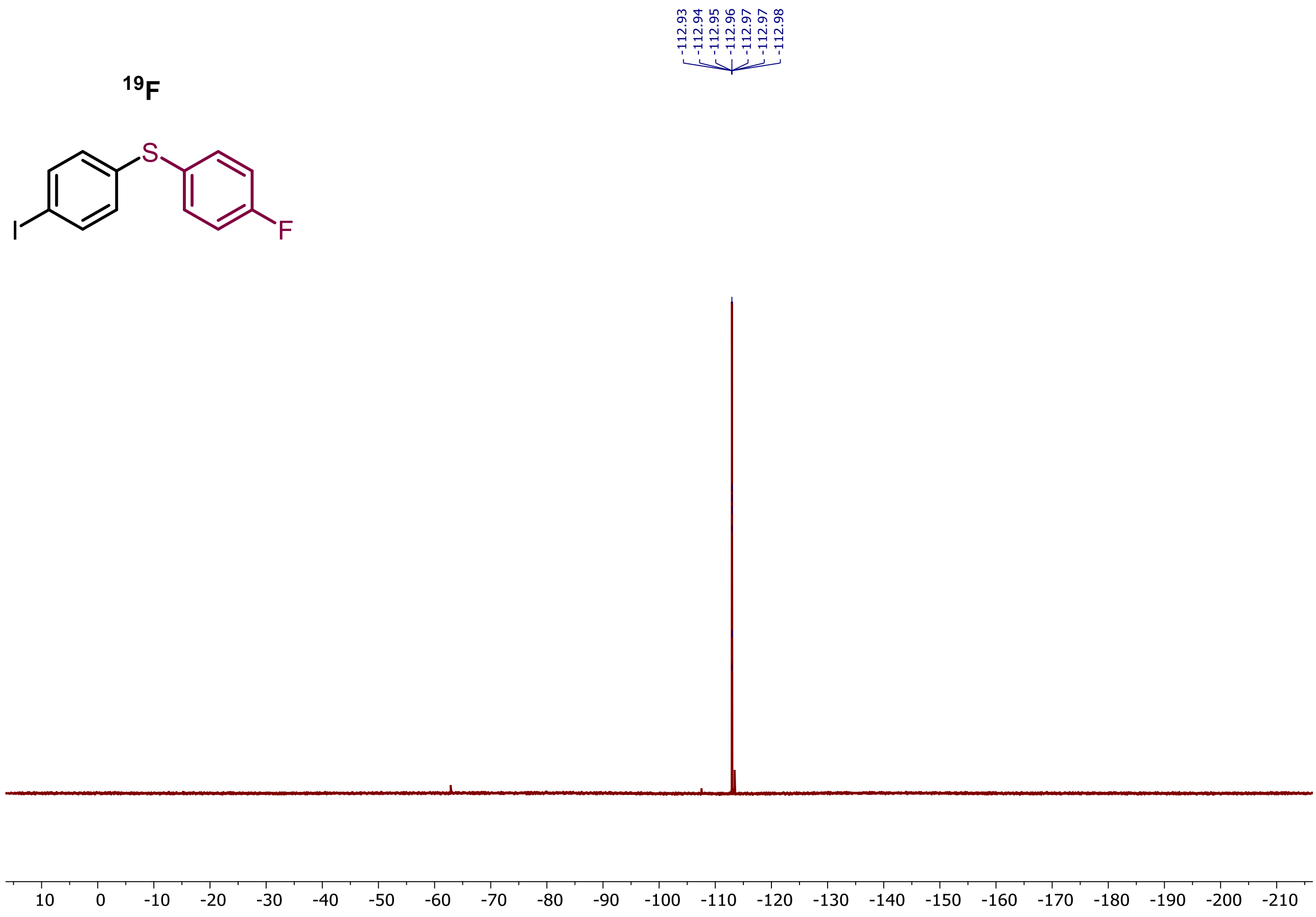
-112.93
-112.94
-112.95
-112.96
-112.97
-112.97
-112.98
19F
S
I
F
10 0 -10 -20 -30 -40 -50 -60 -70 -80 -90 -100 -110 -120 -130 -140 -150 -160 -170 -180 -190 -200 -210

(5-Bromo-2-methoxyphenyl)(4-fluorophenyl)sulfane (5)

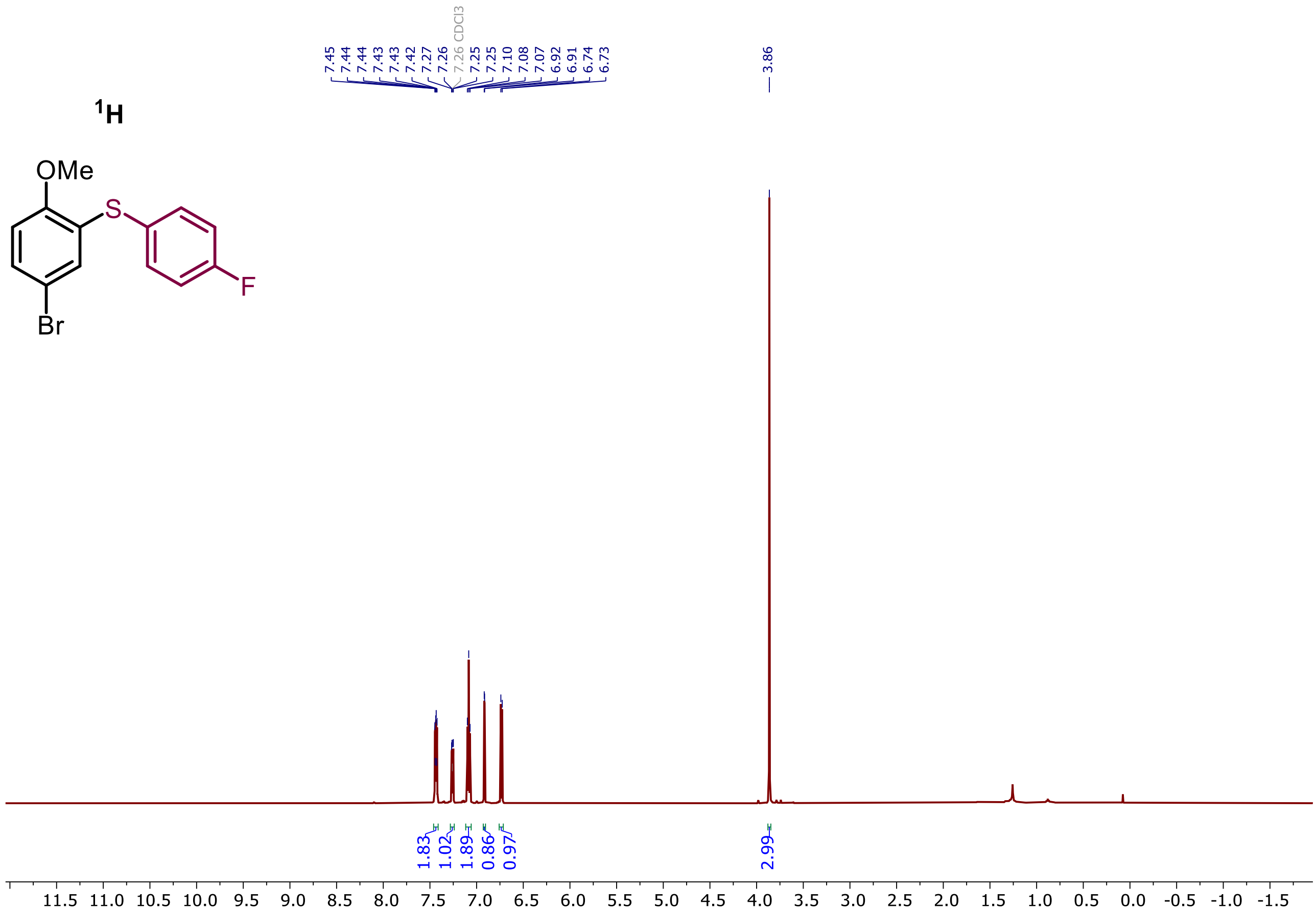

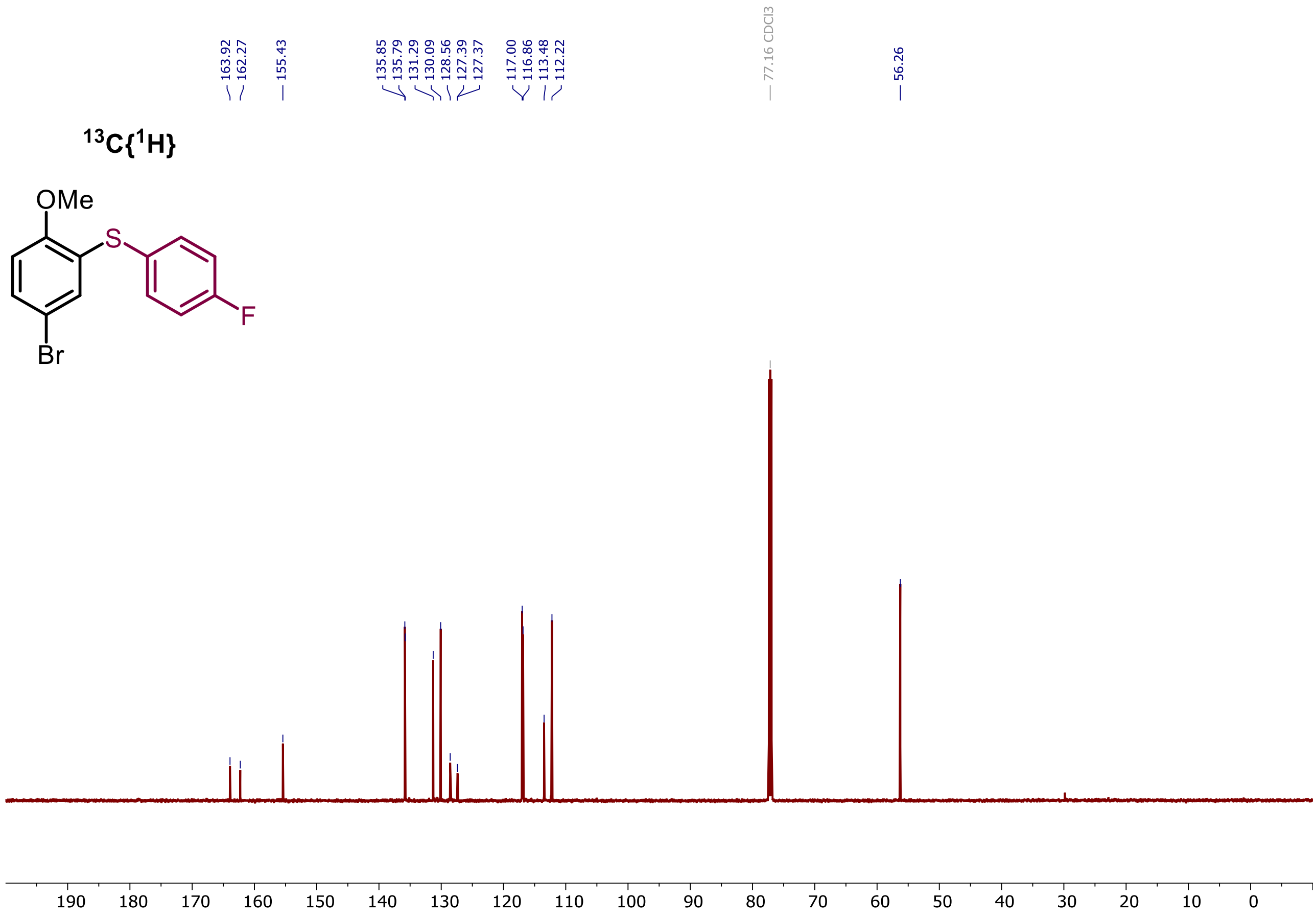

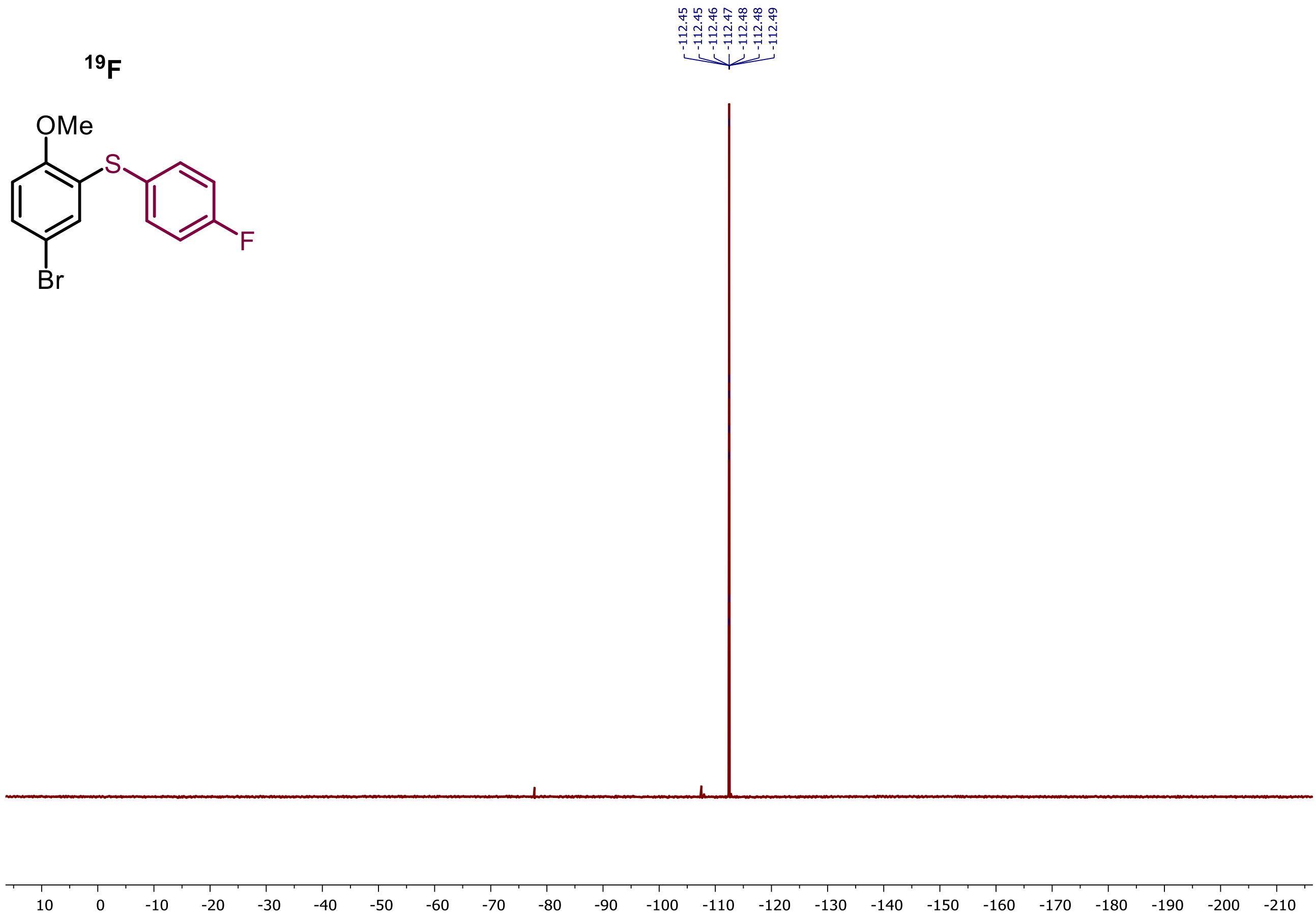

(5-bromo-2-methoxyphenyl)(4-chlorophenyl)sulfane (6)

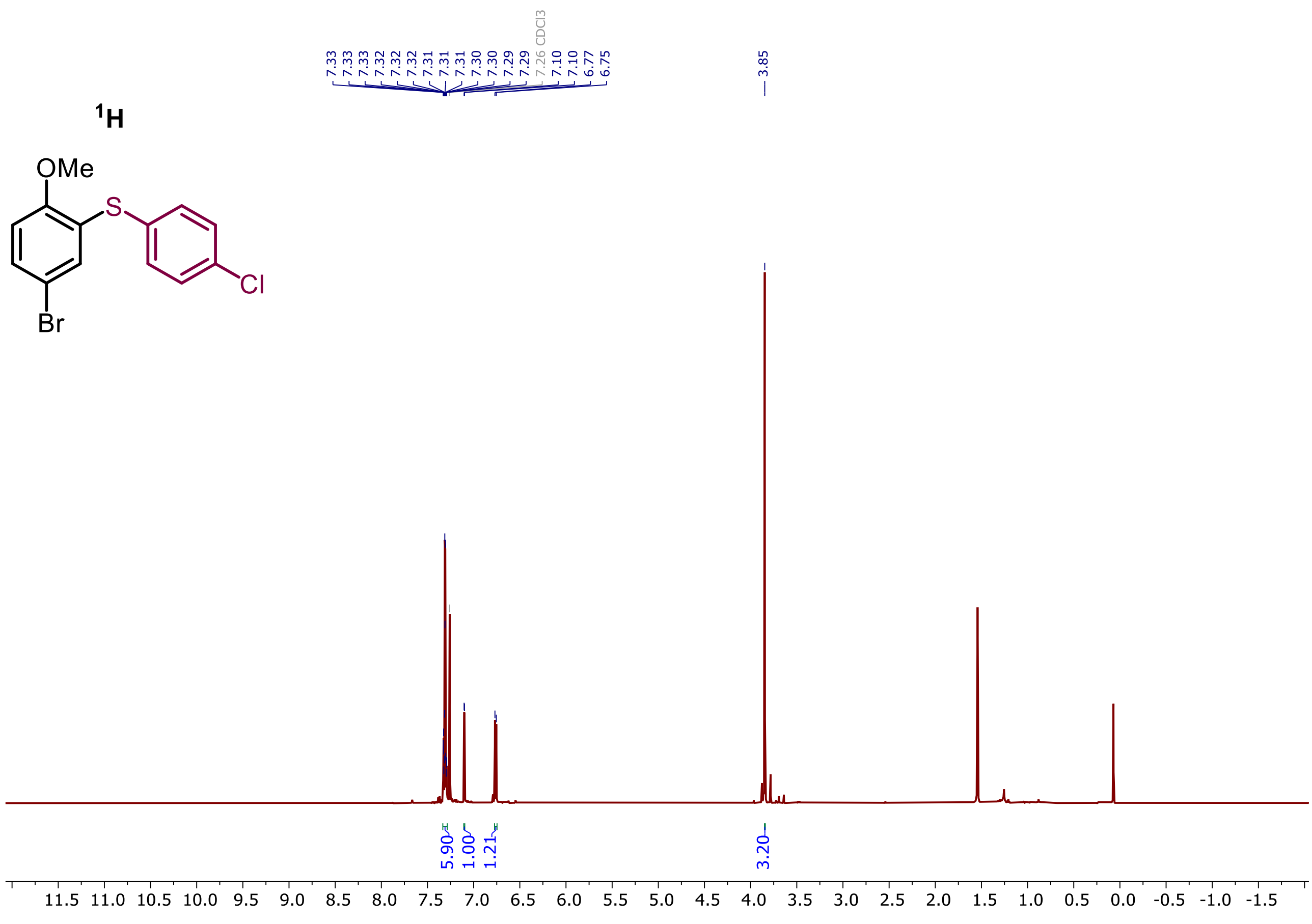

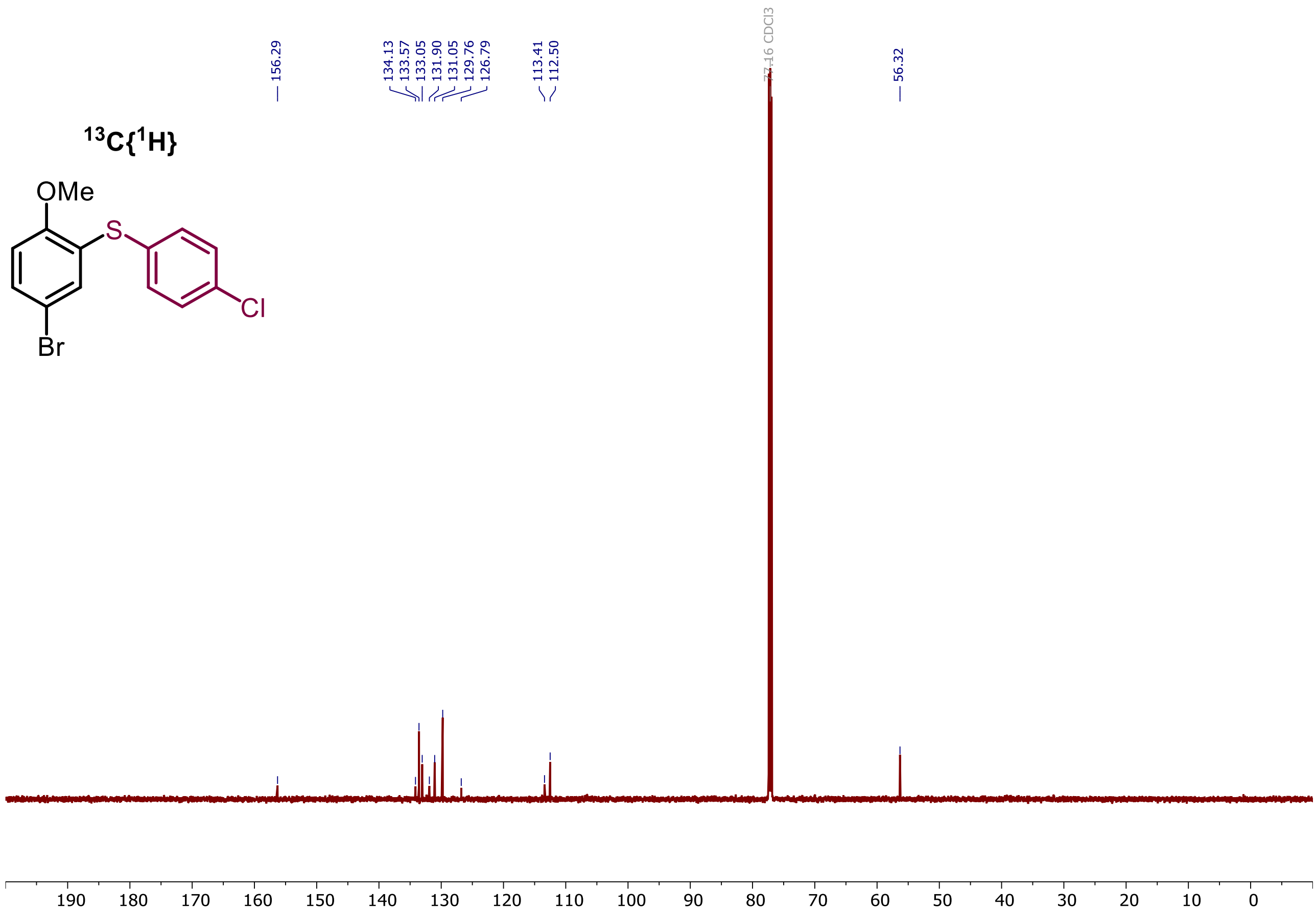

(5-Bromo-2-methoxyphenyl)(4-(*tert*-butyl)phenyl)sulfane (7)

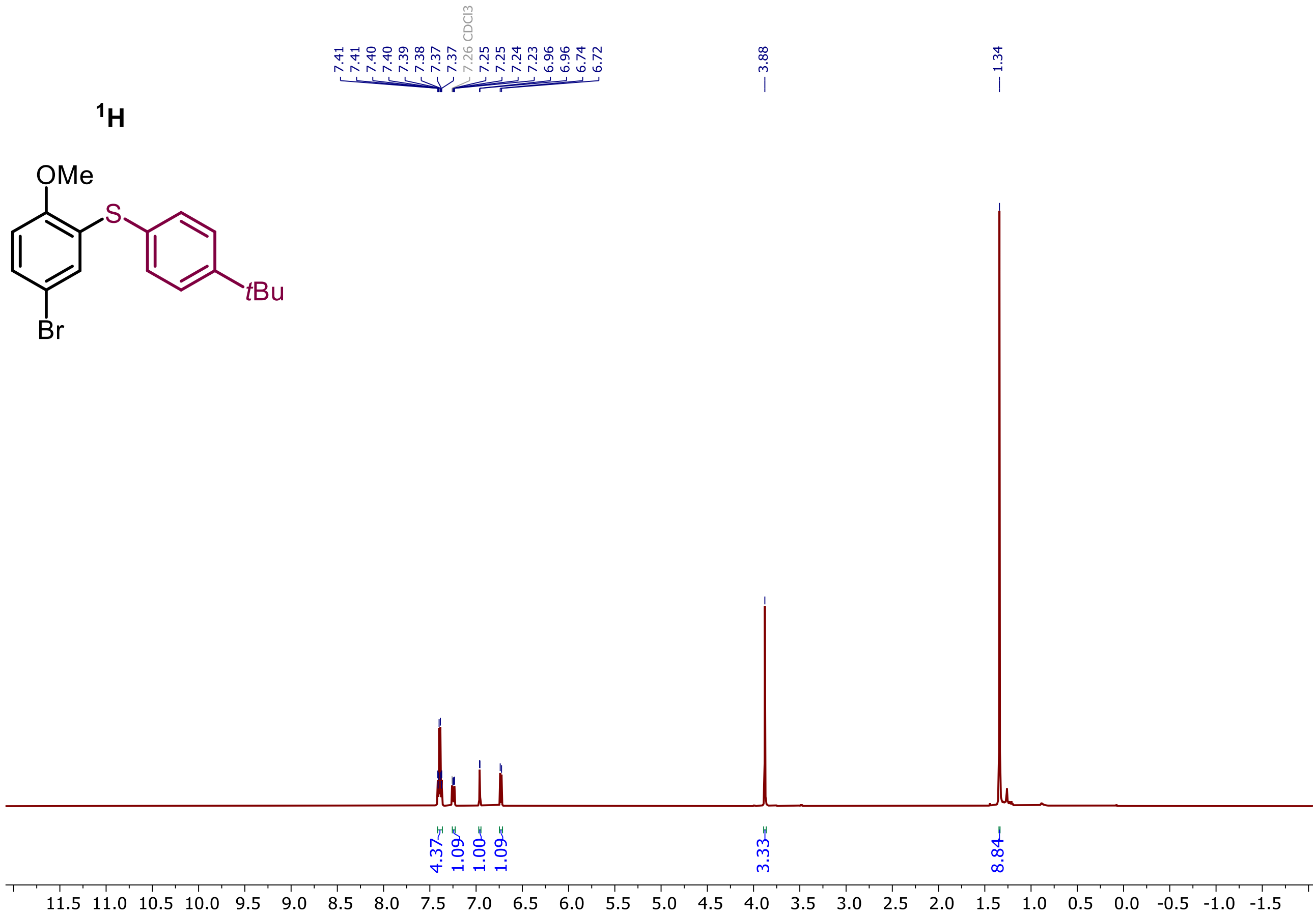

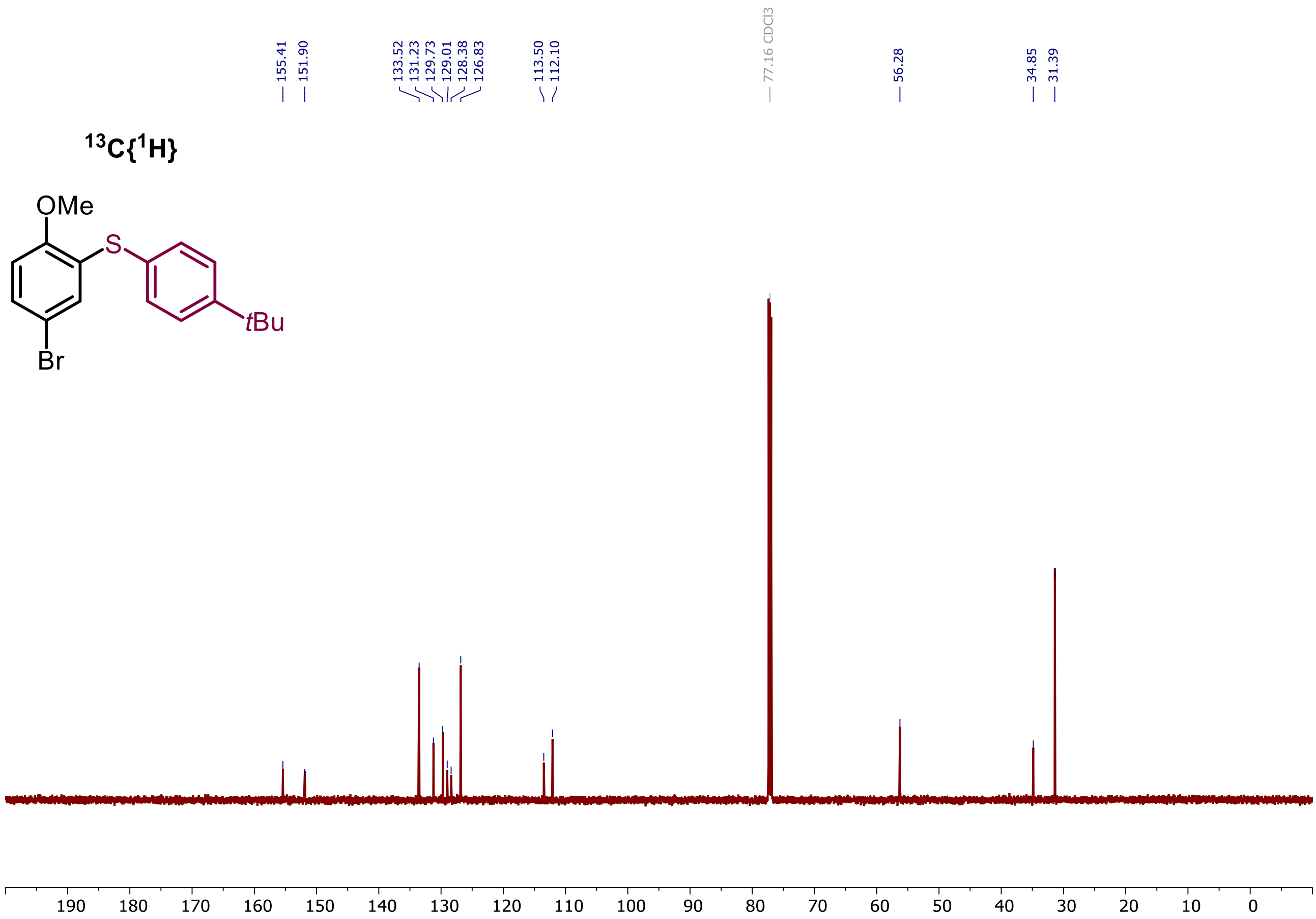

(5-Bromo-2-methoxyphenyl)(phenyl)sulfane (8)

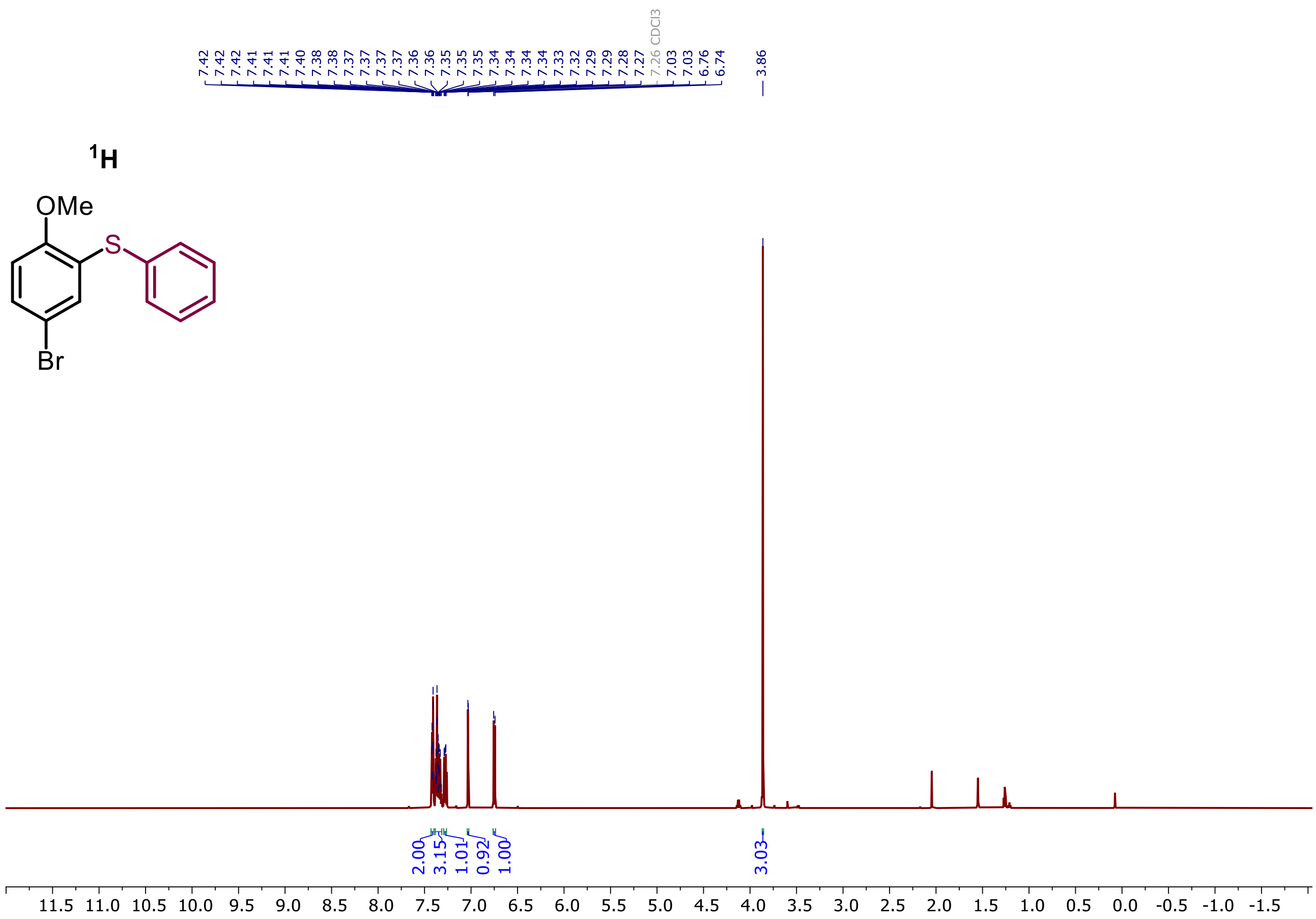

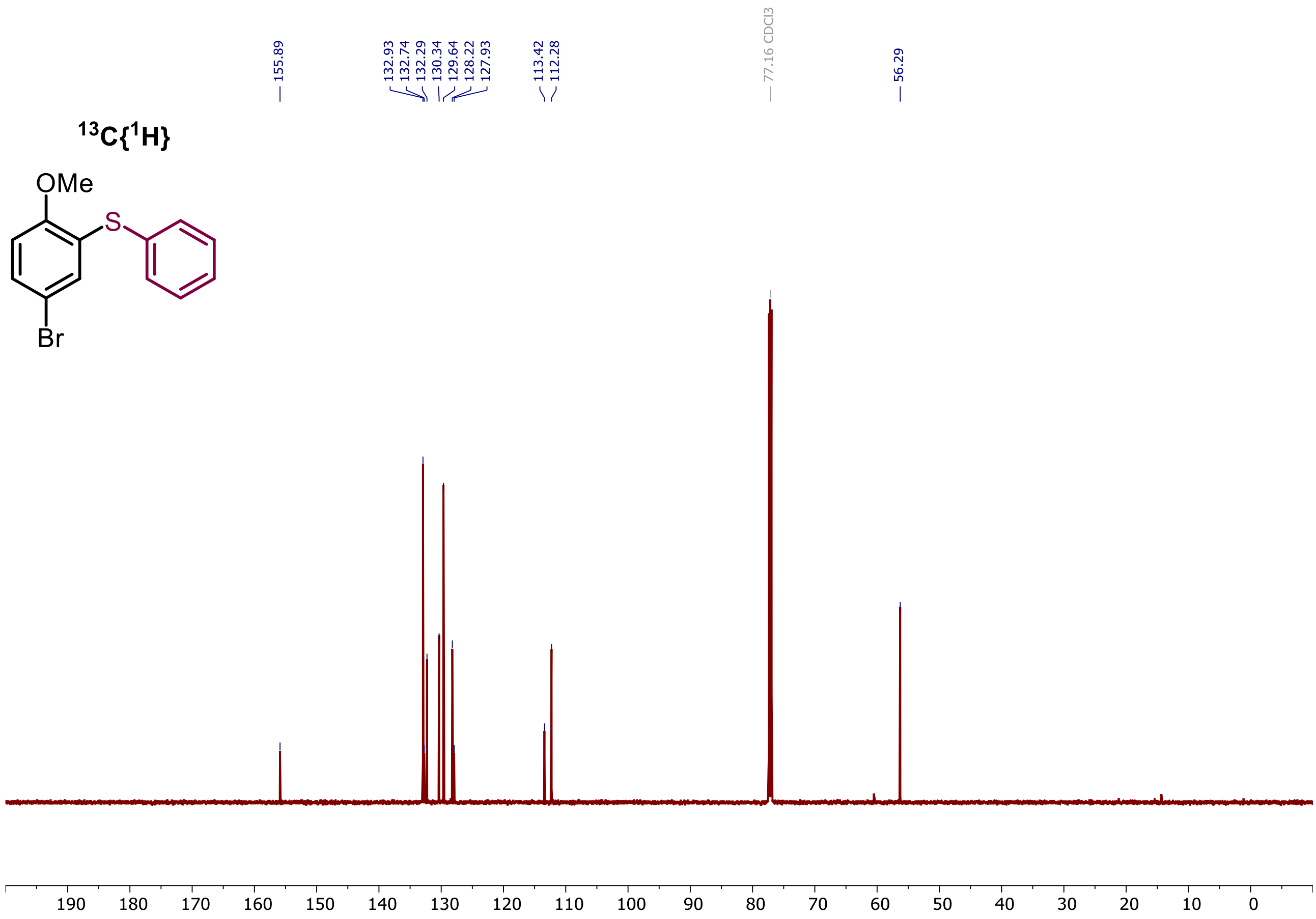

(3-((4-Fluorophenyl)thio)phenyl)trimethylsilane (9)

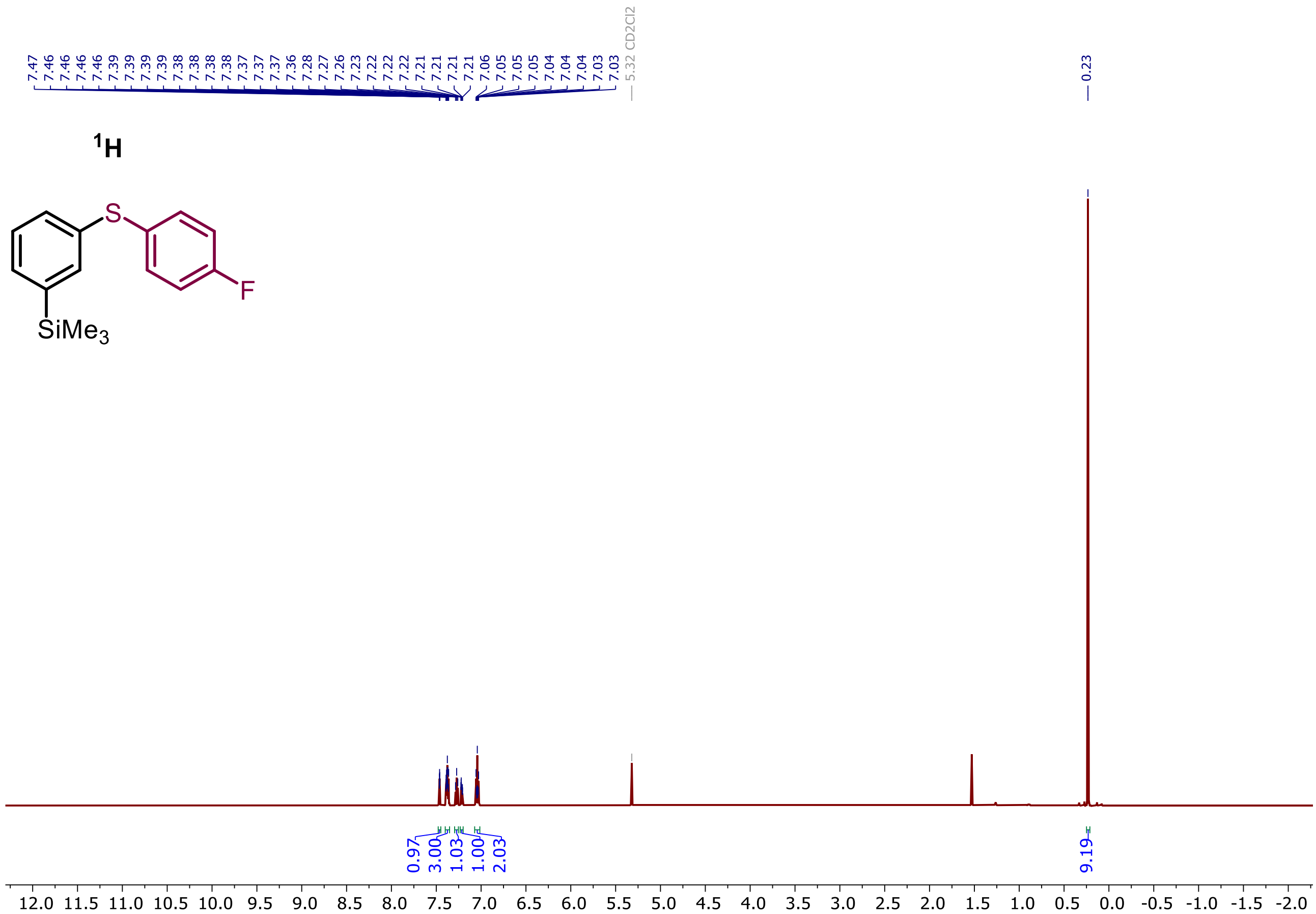

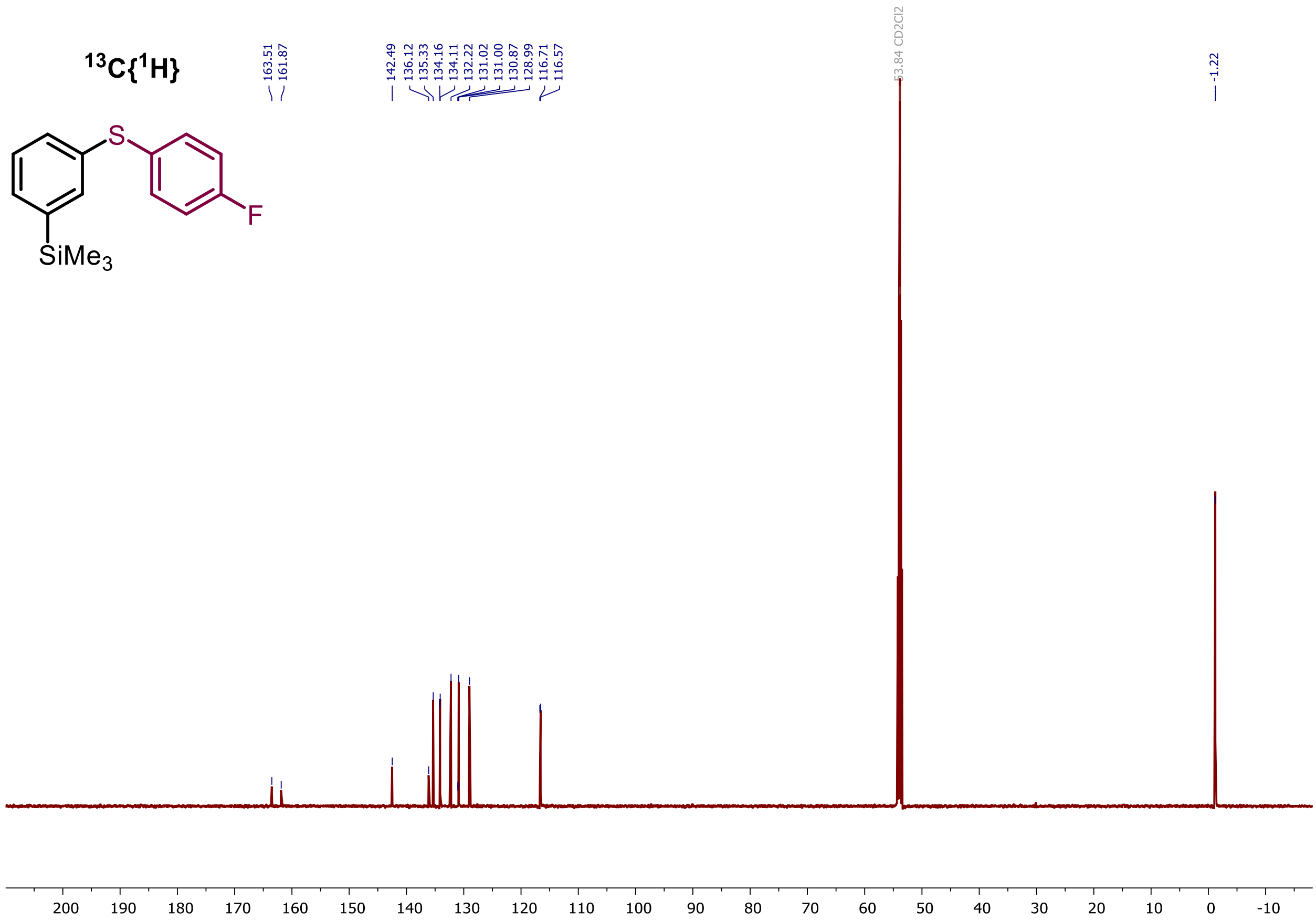
13C{1H}
163.51
161.87
142.49
136.12
135.33
134.16
134.11
132.22
131.02
131.00
130.87
128.99
116.71
116.57
53.84 CD2Cl2
-1.22
S
F
SiMe3
200 190 180 170 160 150 140 130 120 110 100 90 80 70 60 50 40 30 20 10 0 -10

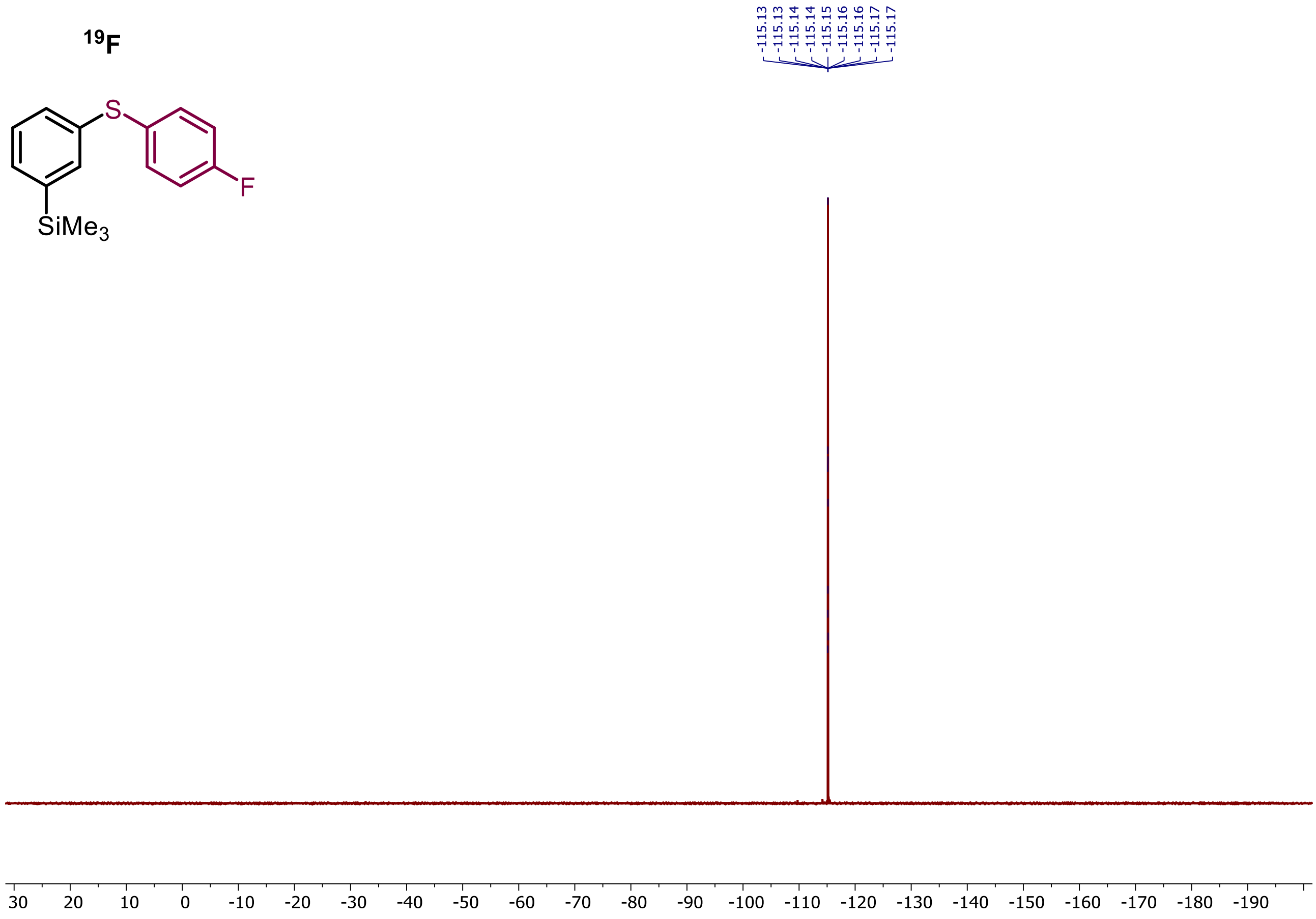
19F
-115.13
-115.13
-115.14
-115.14
-115.15
-115.15
-115.16
-115.16
-115.17
-115.17
S
F
SiMe3
30 20 10 0 -10 -20 -30 -40 -50 -60 -70 -80 -90 -100 -110 -120 -130 -140 -150 -160 -170 -180 -190

(3-Bromo-5-((4-fluorophenyl)thio)phenyl)trimethylsilane (10)

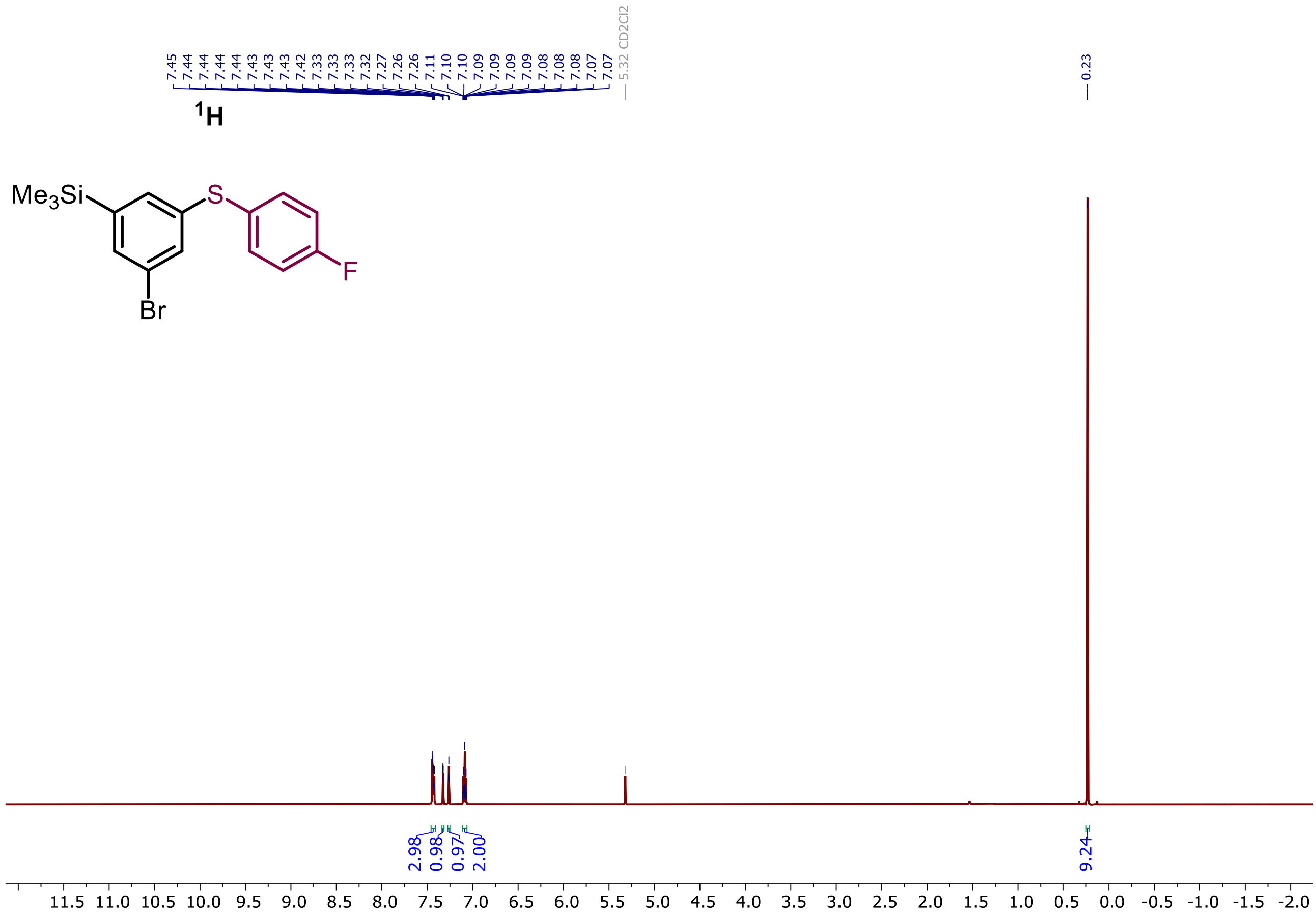

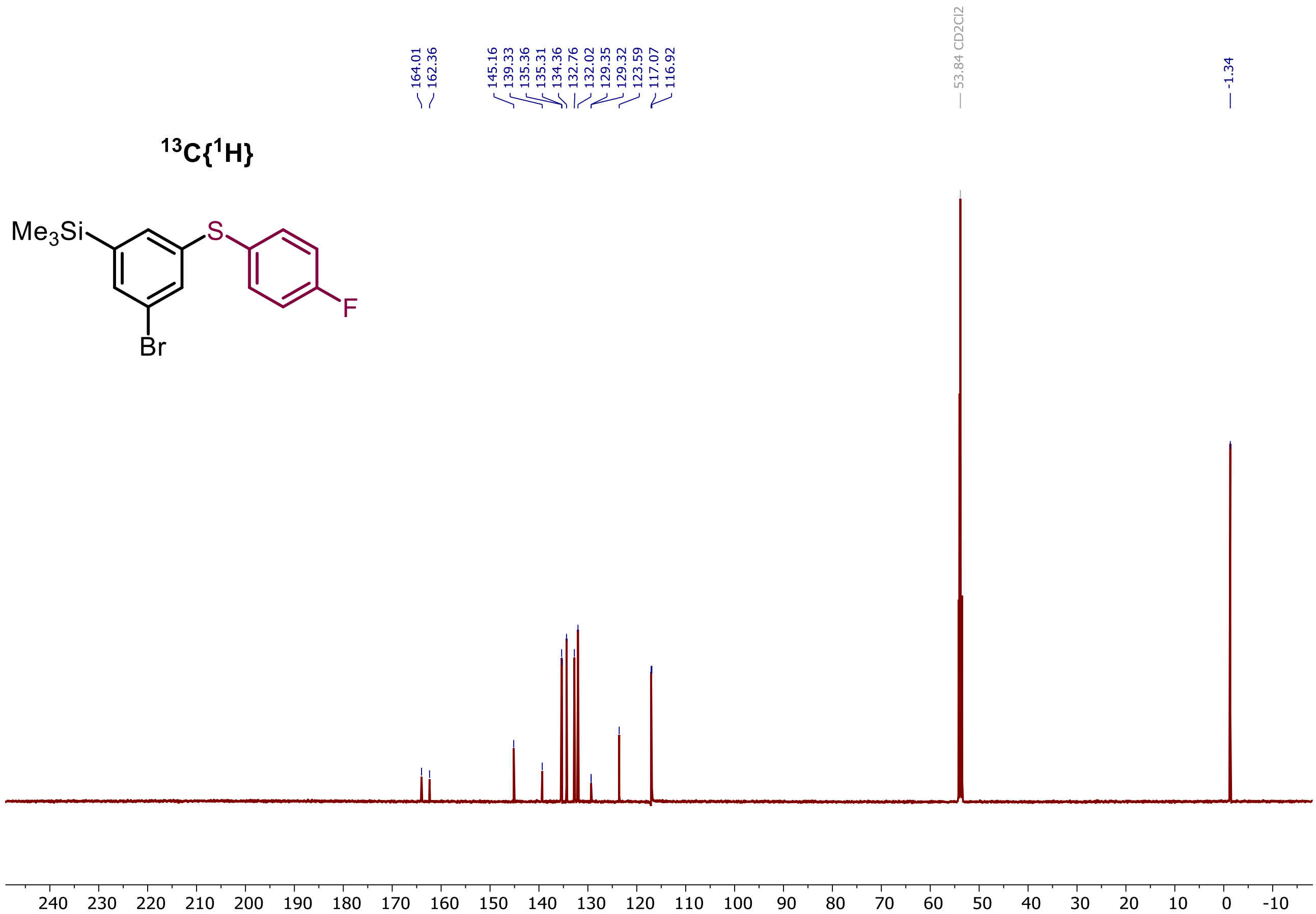

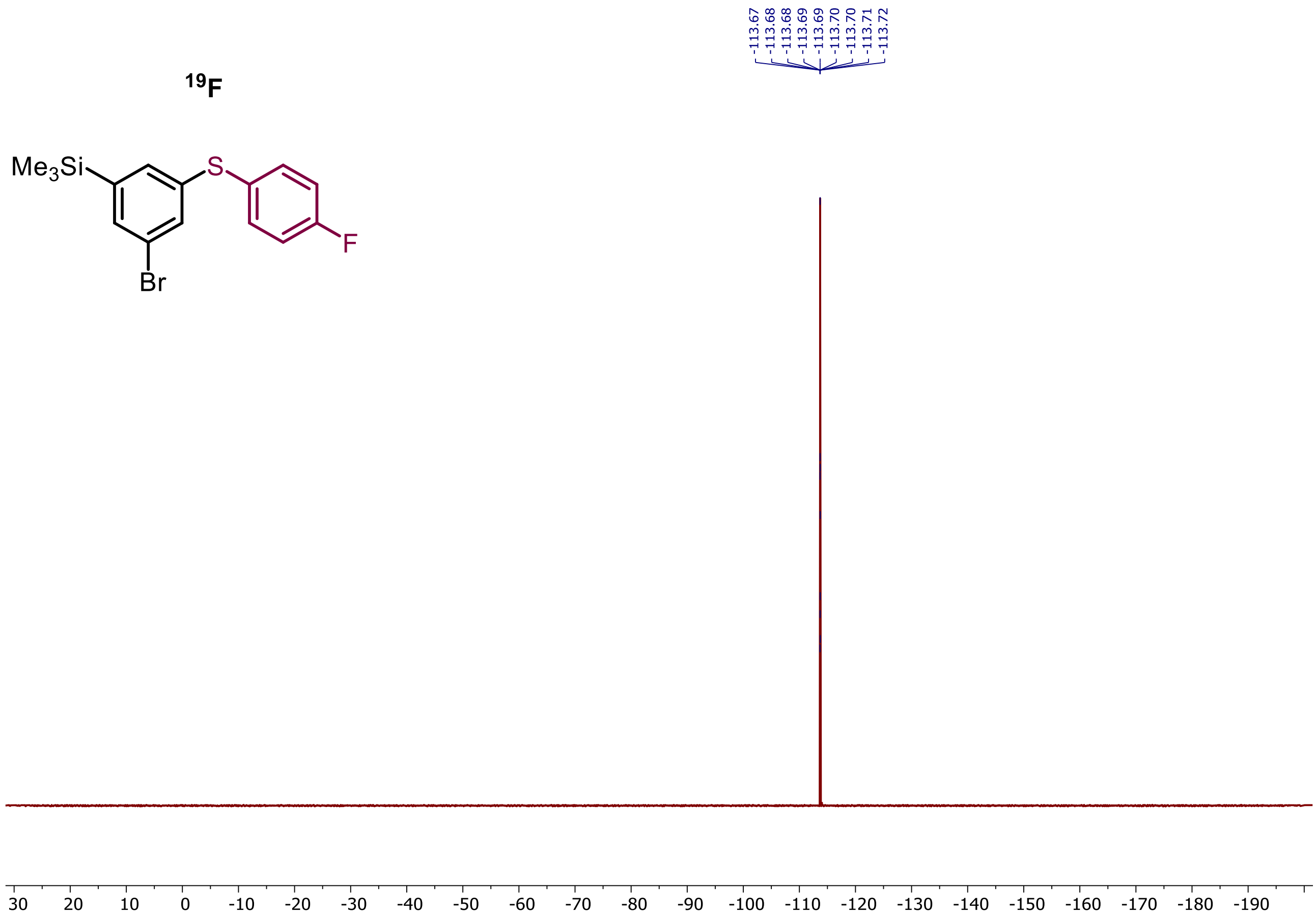

(4-Fluorophenyl)(3-(trifluoromethyl)phenyl)sulfane (11)

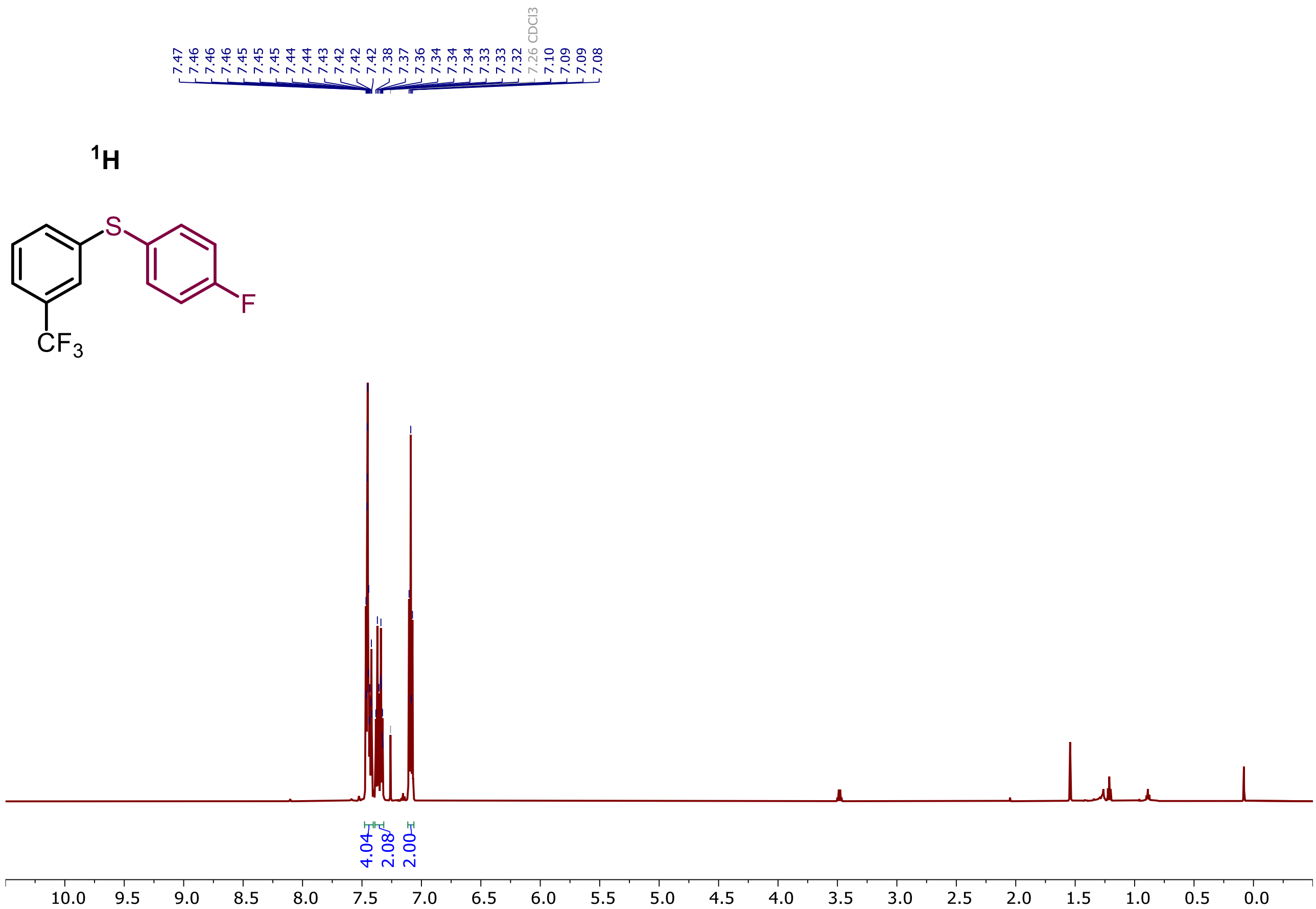

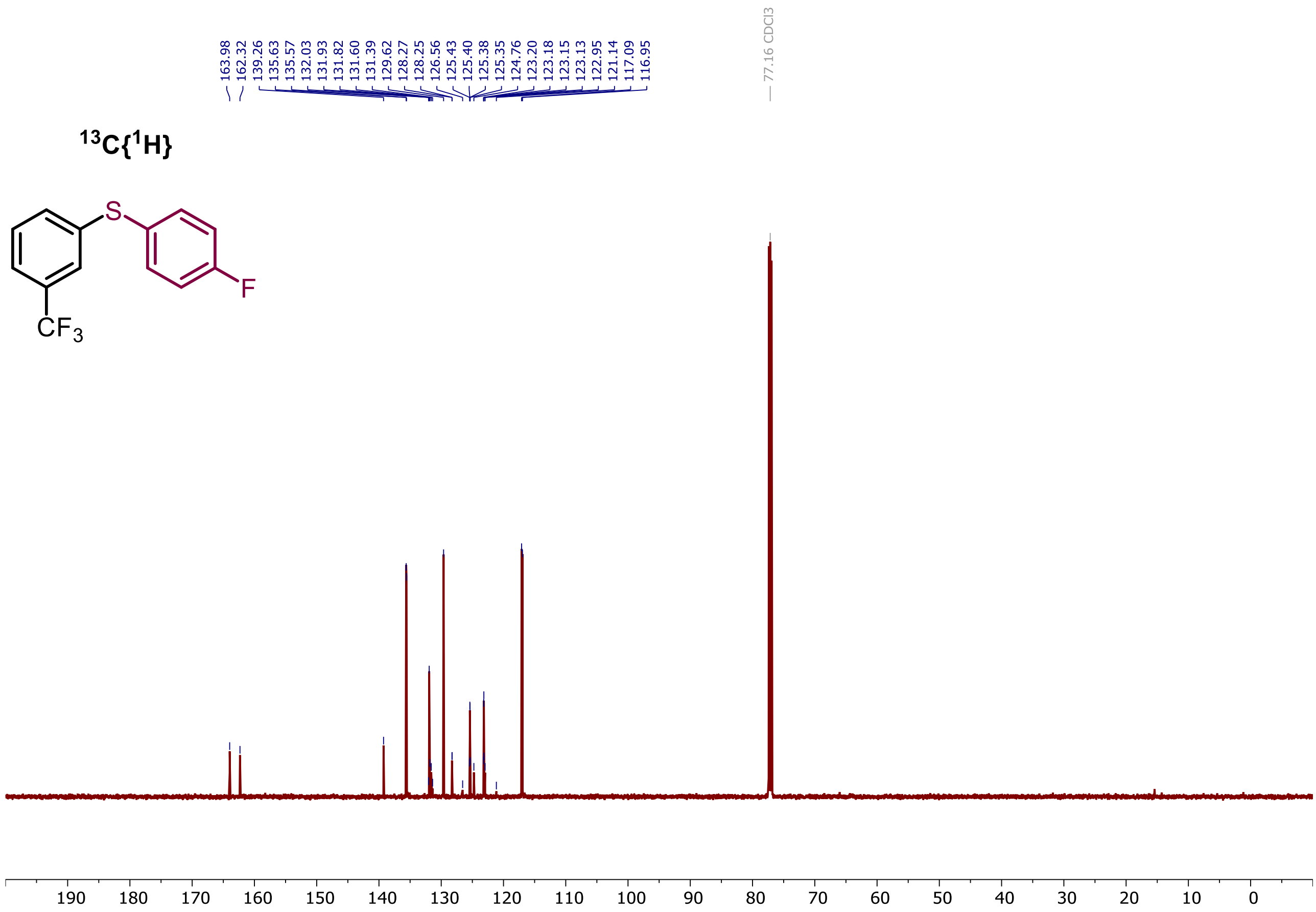
13C{1H}
S
F
CF3
163.98
162.32
139.26
135.63
135.57
132.03
131.93
131.82
131.60
131.39
129.62
128.27
128.25
126.56
125.43
125.40
125.38
125.35
124.76
123.20
123.18
123.15
123.13
122.95
121.14
117.09
116.95
77.16 CDCl3
190
180
170
160
150
140
130
120
110
100
90
80
70
60
50
40
30
20
10
0

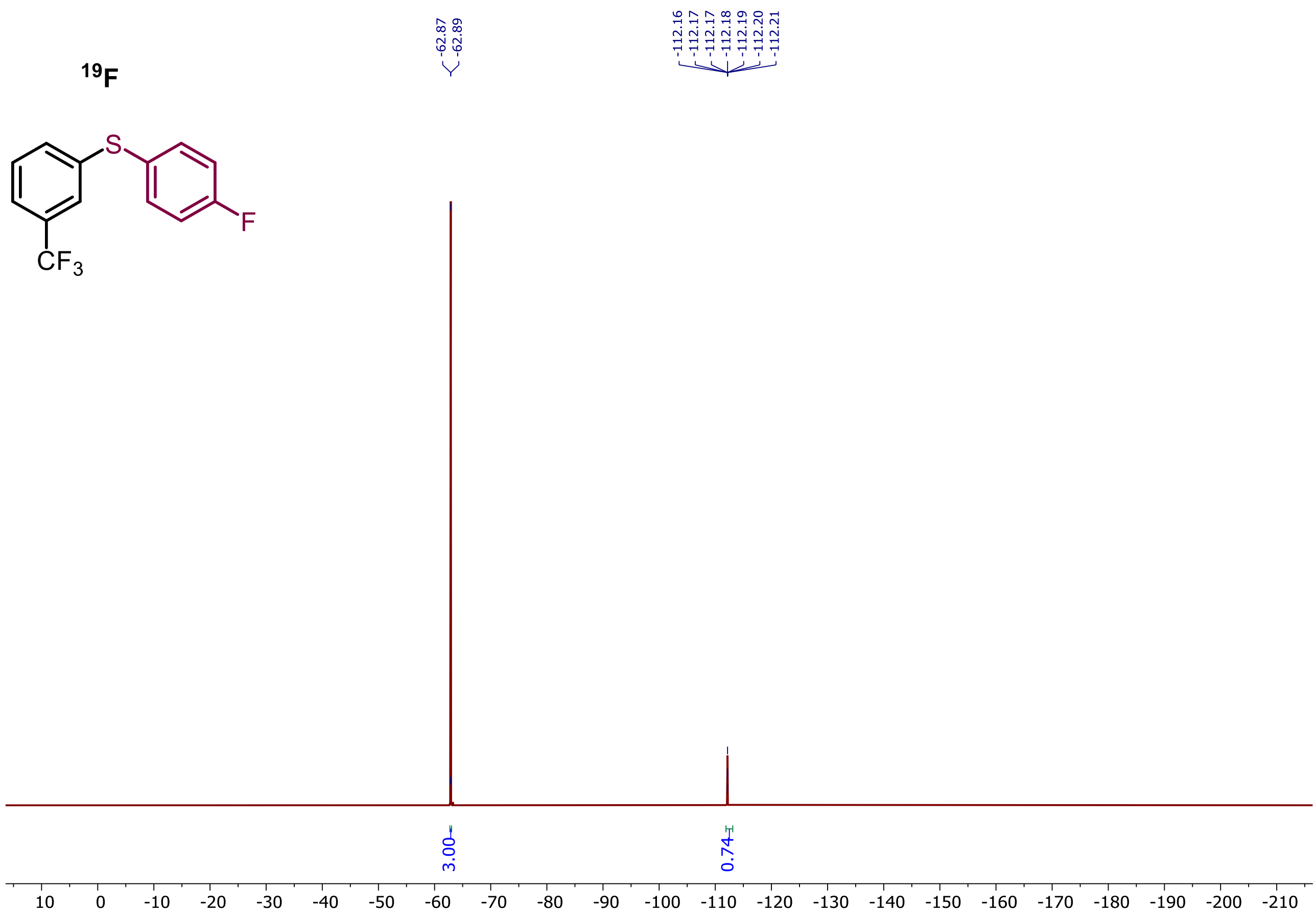
19F
S
F
CF3
-62.87
-62.89
-112.16
-112.17
-112.17
-112.18
-112.19
-112.20
-112.21
3.00
0.74
10
0
-10
-20
-30
-40
-50
-60
-70
-80
-90
-100
-110
-120
-130
-140
-150
-160
-170
-180
-190
-200
-210

5-((4-Fluorophenyl)thio)-2-methoxybenzonitrile (12)

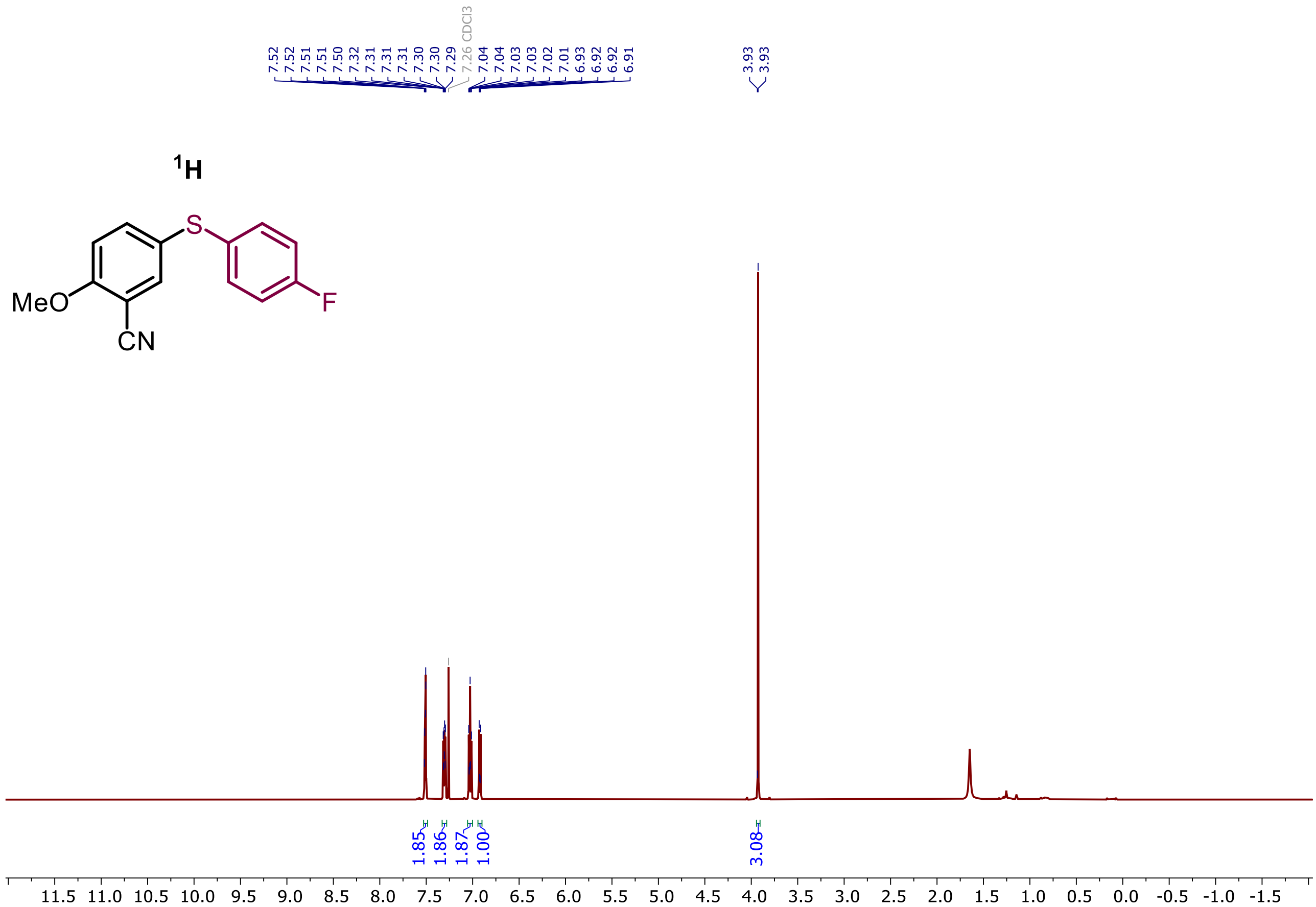

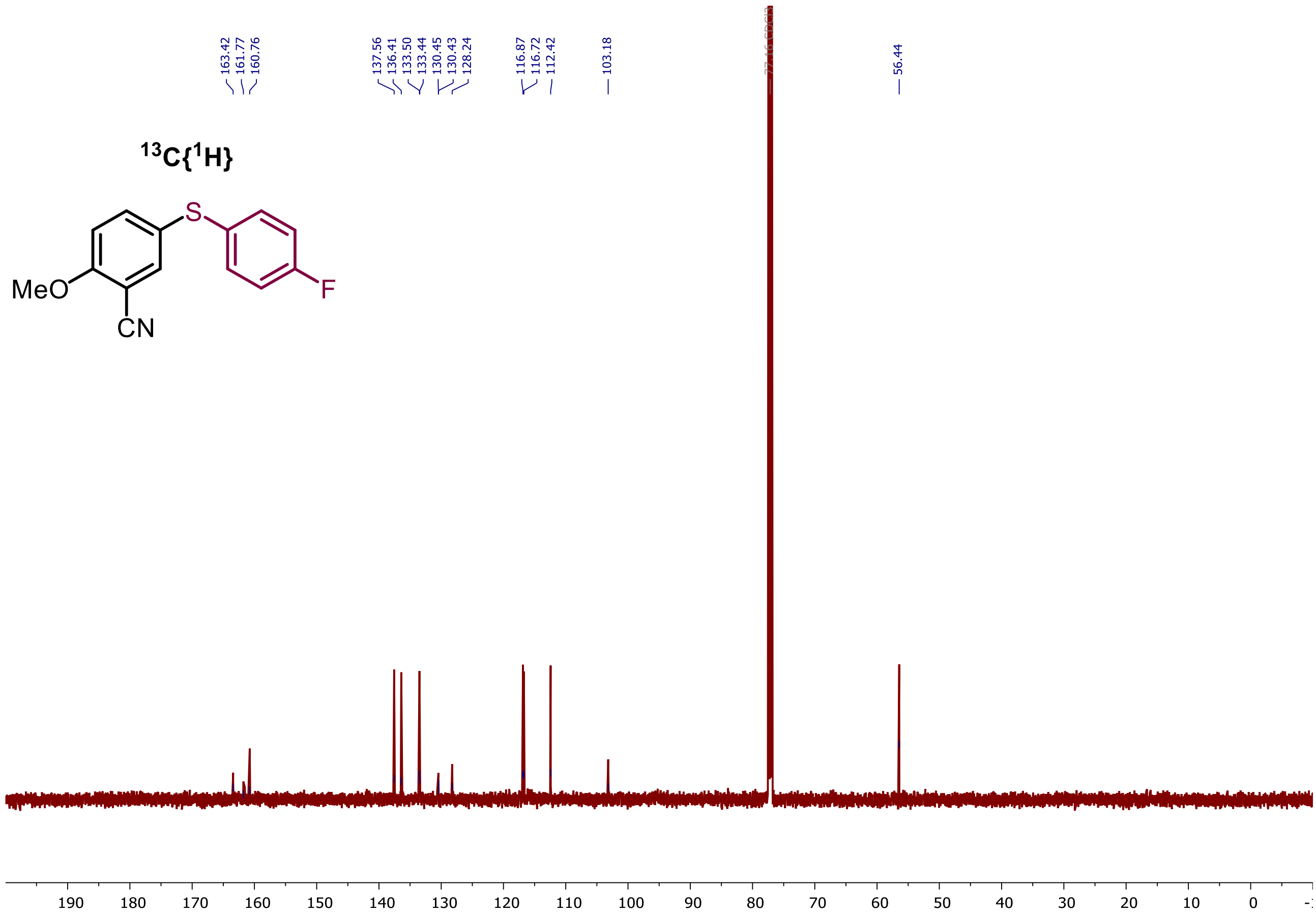

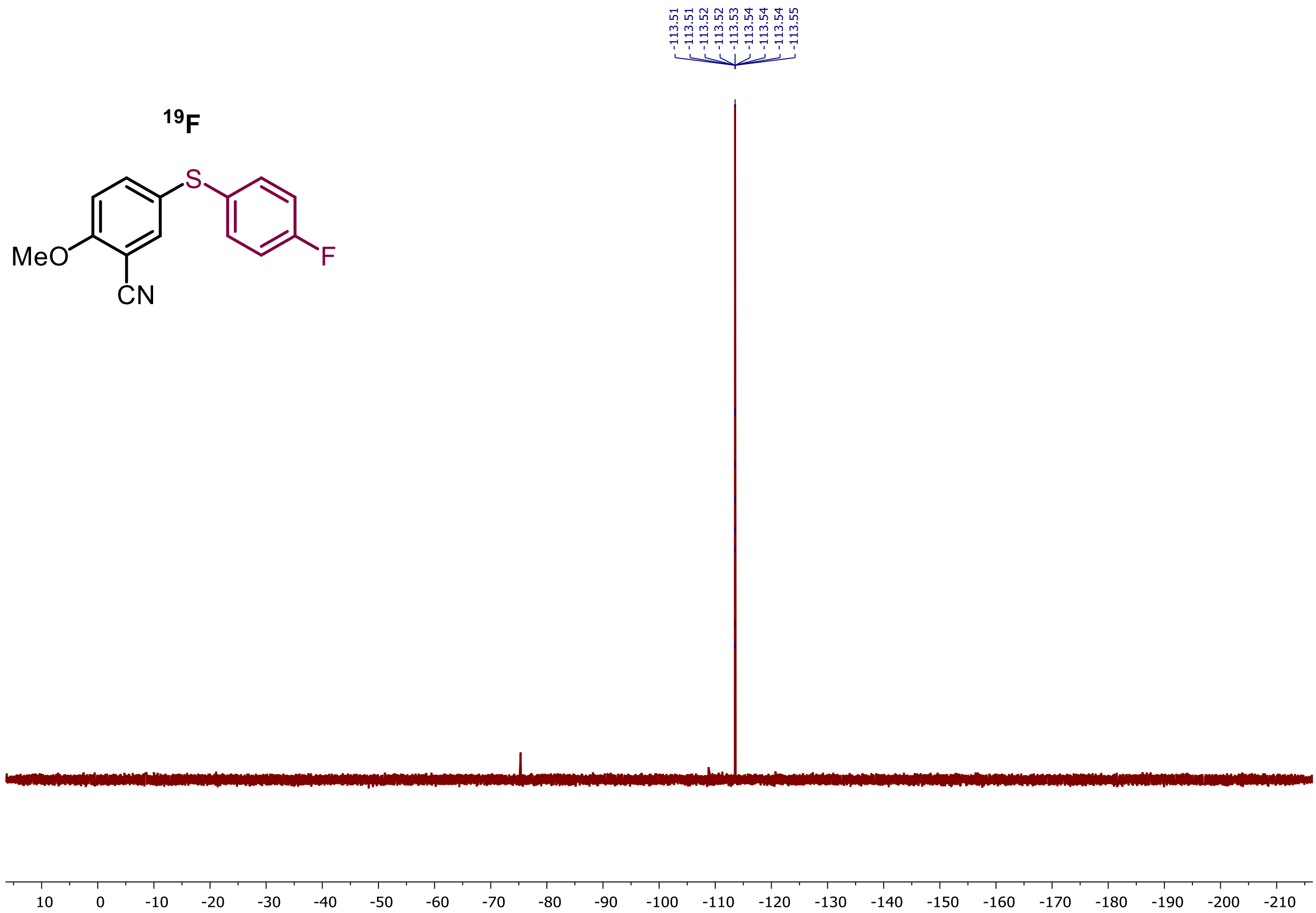

5-((4-Bromophenyl)thio)-2-methoxybenzonitrile (13)

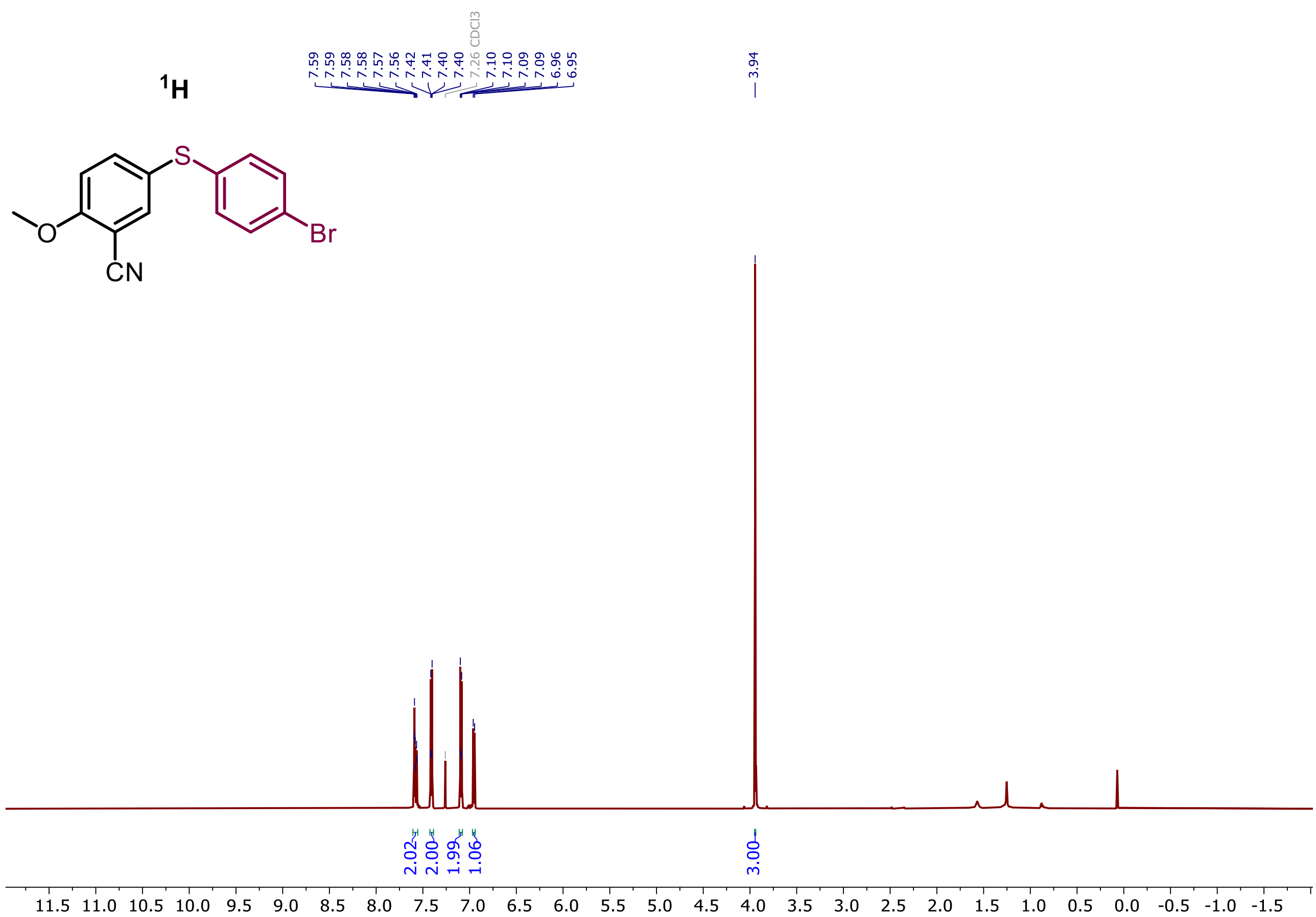

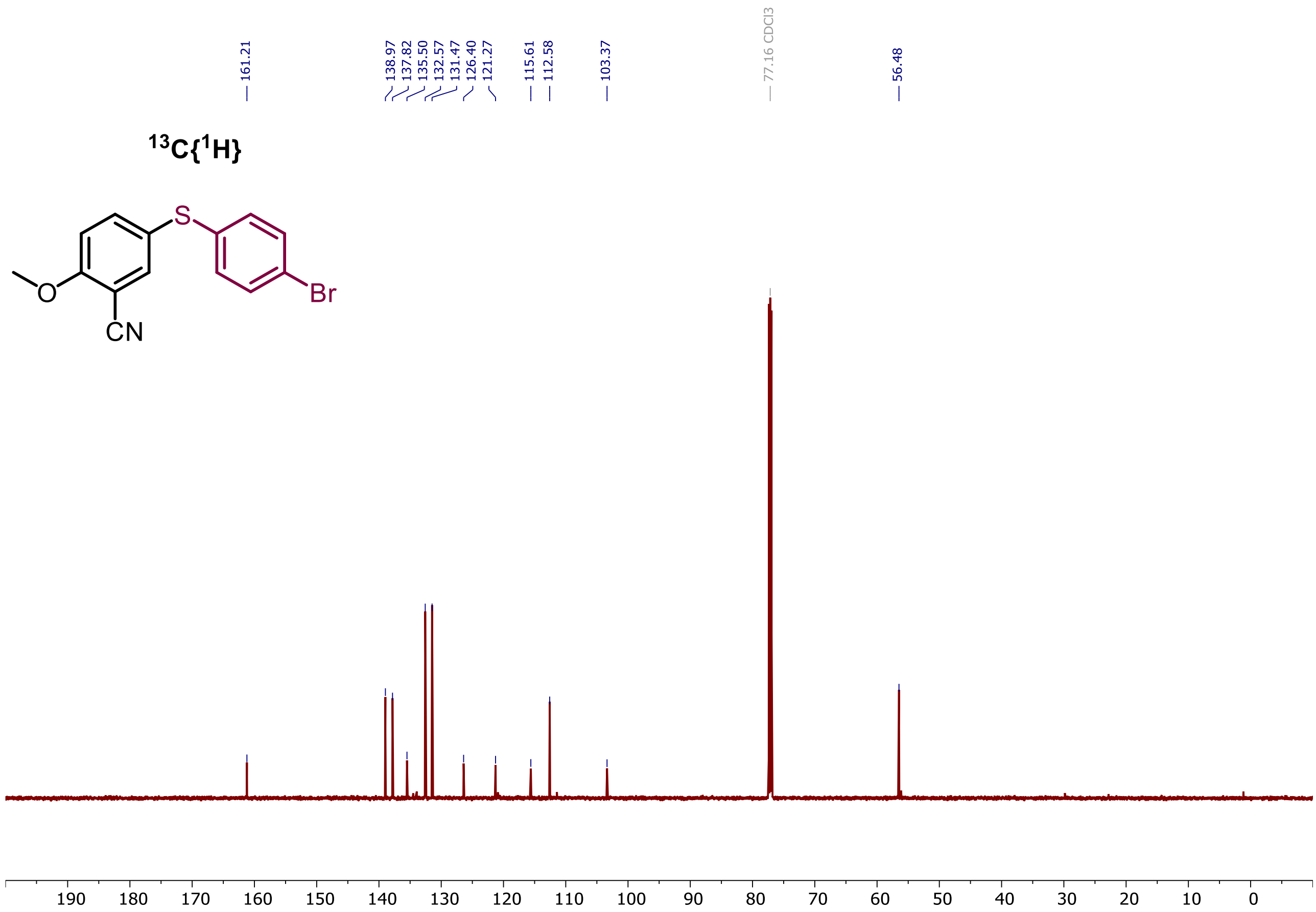

(4-Bromo-3-chloro-2-methylphenyl)(4-fluorophenyl)sulfane (14)

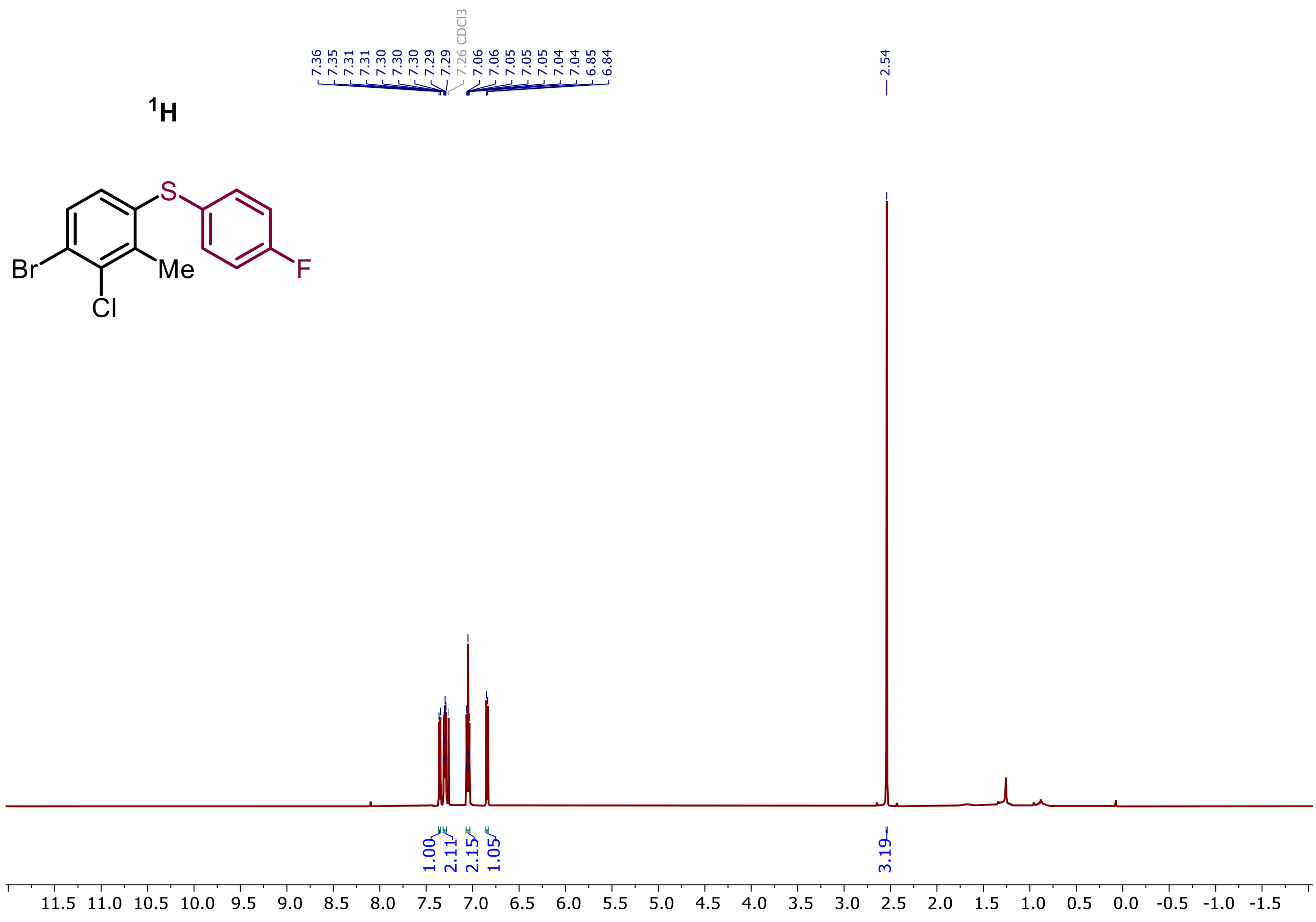

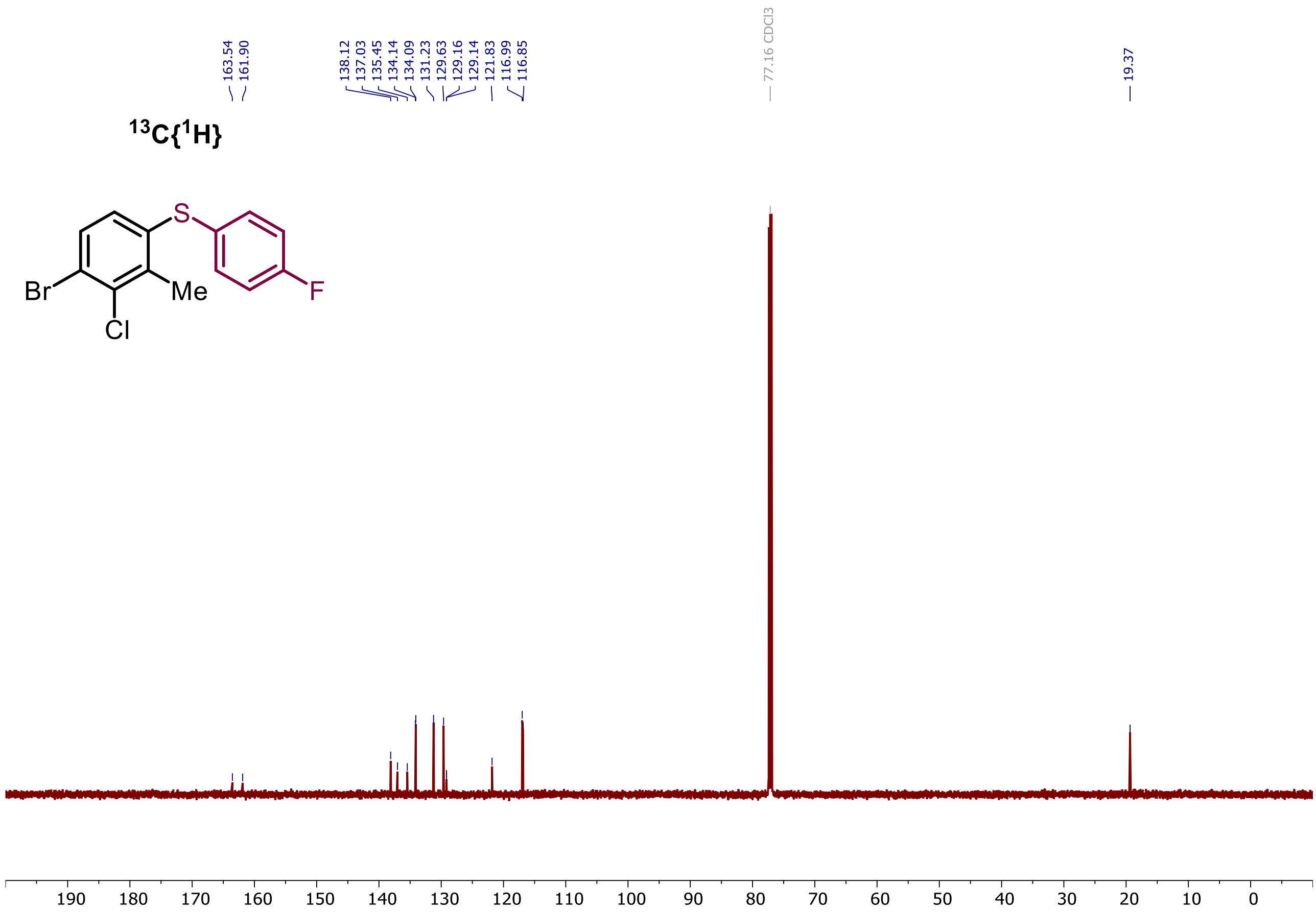

163.54
161.90
138.12
137.03
135.45
134.14
134.09
131.23
129.63
129.16
129.14
121.83
116.99
116.85
77.16 CDCl3
19.37
$^{13}C\{^1H\}$
S
Br
Me
F
Cl
190 180 170 160 150 140 130 120 110 100 90 80 70 60 50 40 30 20 10 0

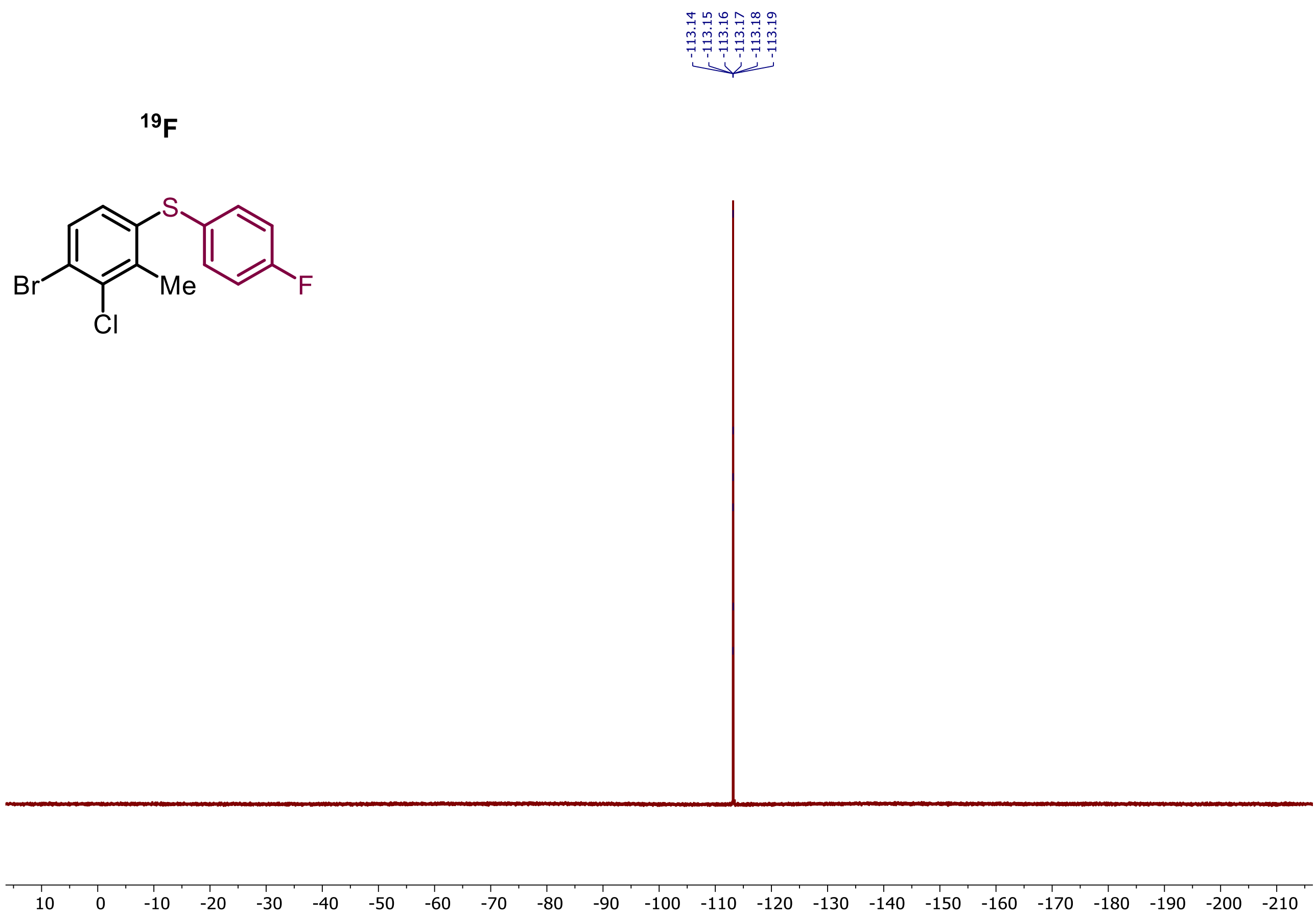

-113.14
-113.15
-113.16
-113.17
-113.18
-113.19
^{19}F
S
Br
Me
F
Cl
10 0 -10 -20 -30 -40 -50 -60 -70 -80 -90 -100 -110 -120 -130 -140 -150 -160 -170 -180 -190 -200 -210

(4-Methoxyphenyl)(2,3,5,6-tetrafluorophenyl)sulfane (15)

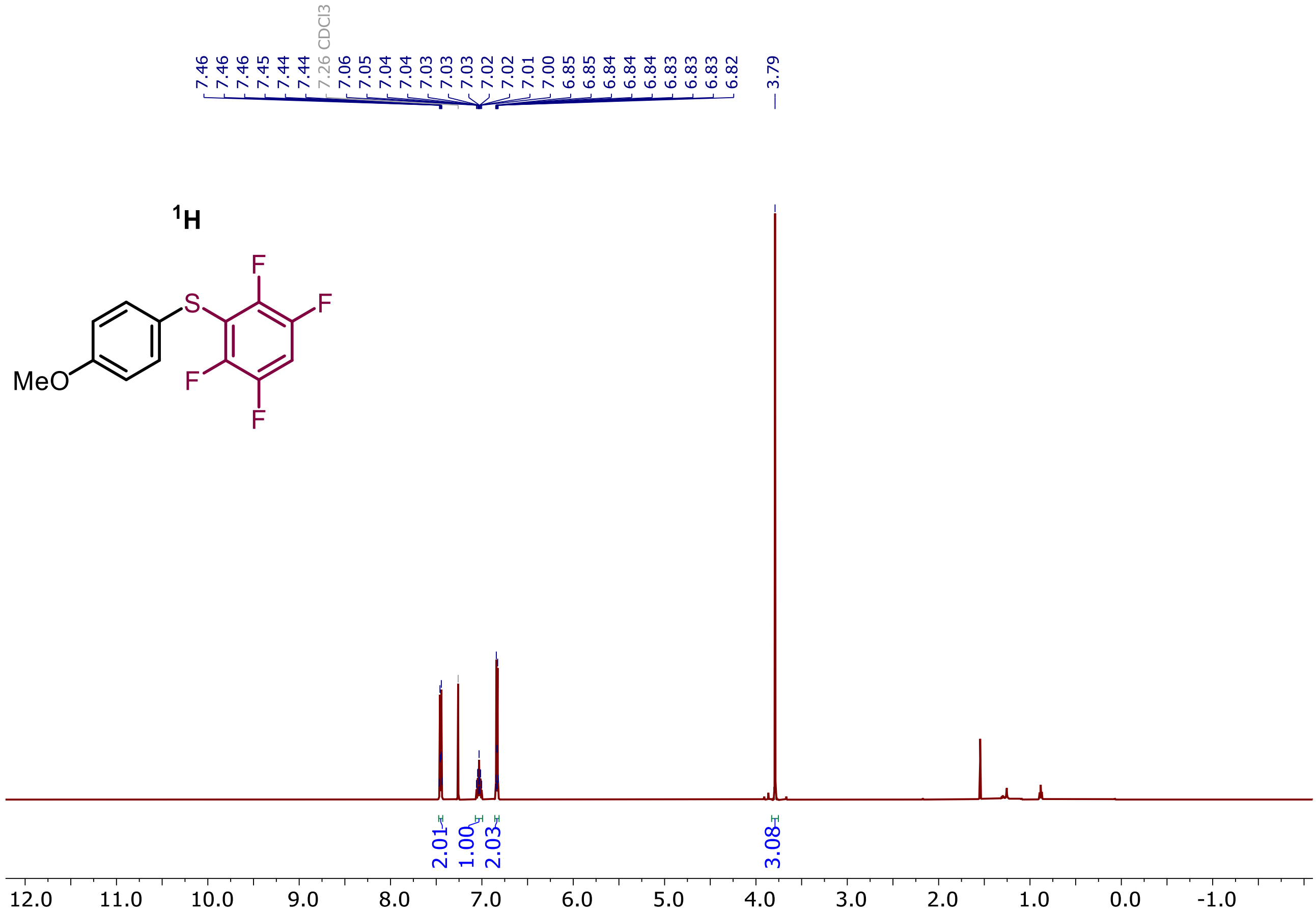

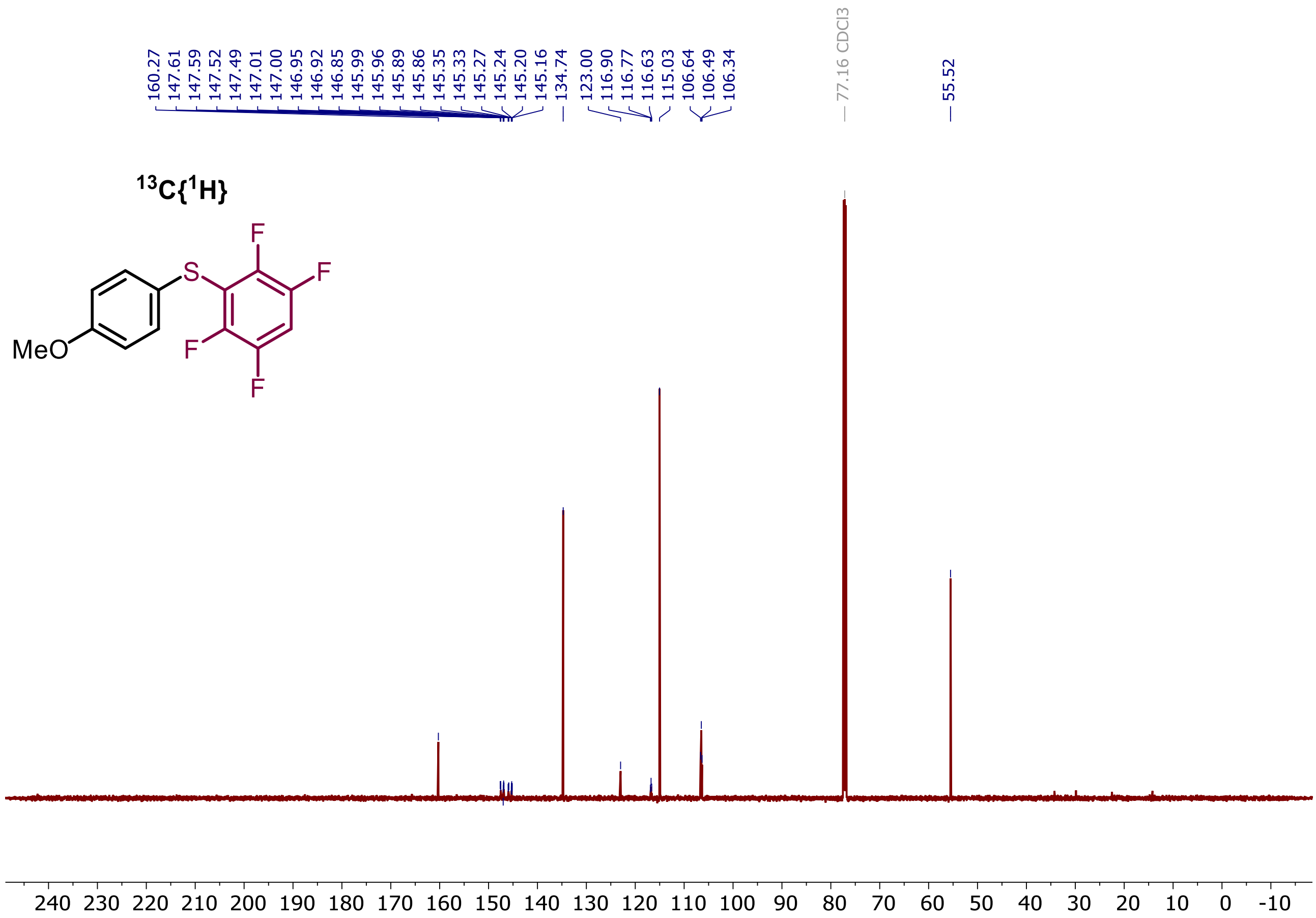

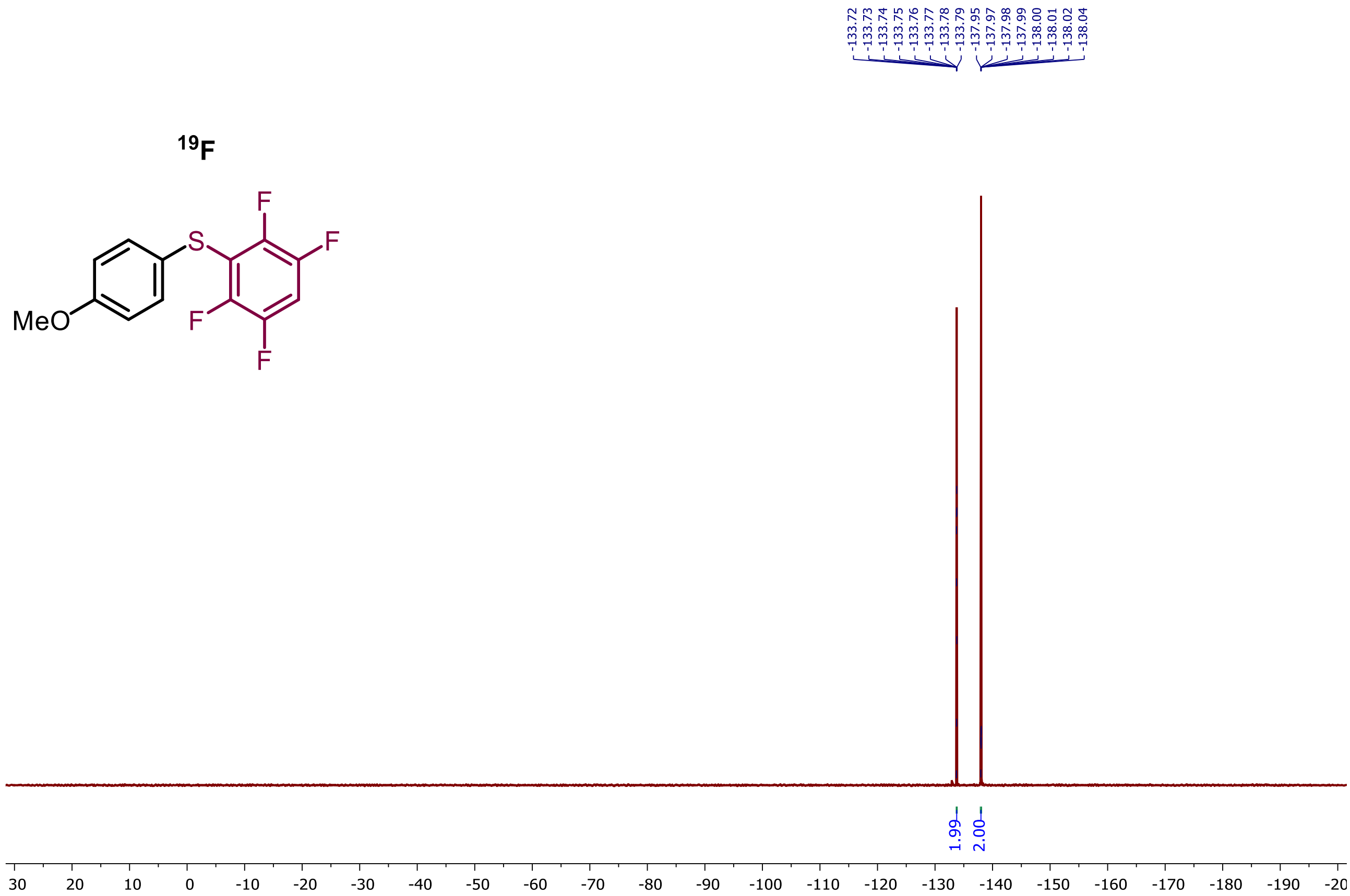

Methyl(4-((2,4,6-triisopropylphenyl)thio)phenyl)sulfane (16)

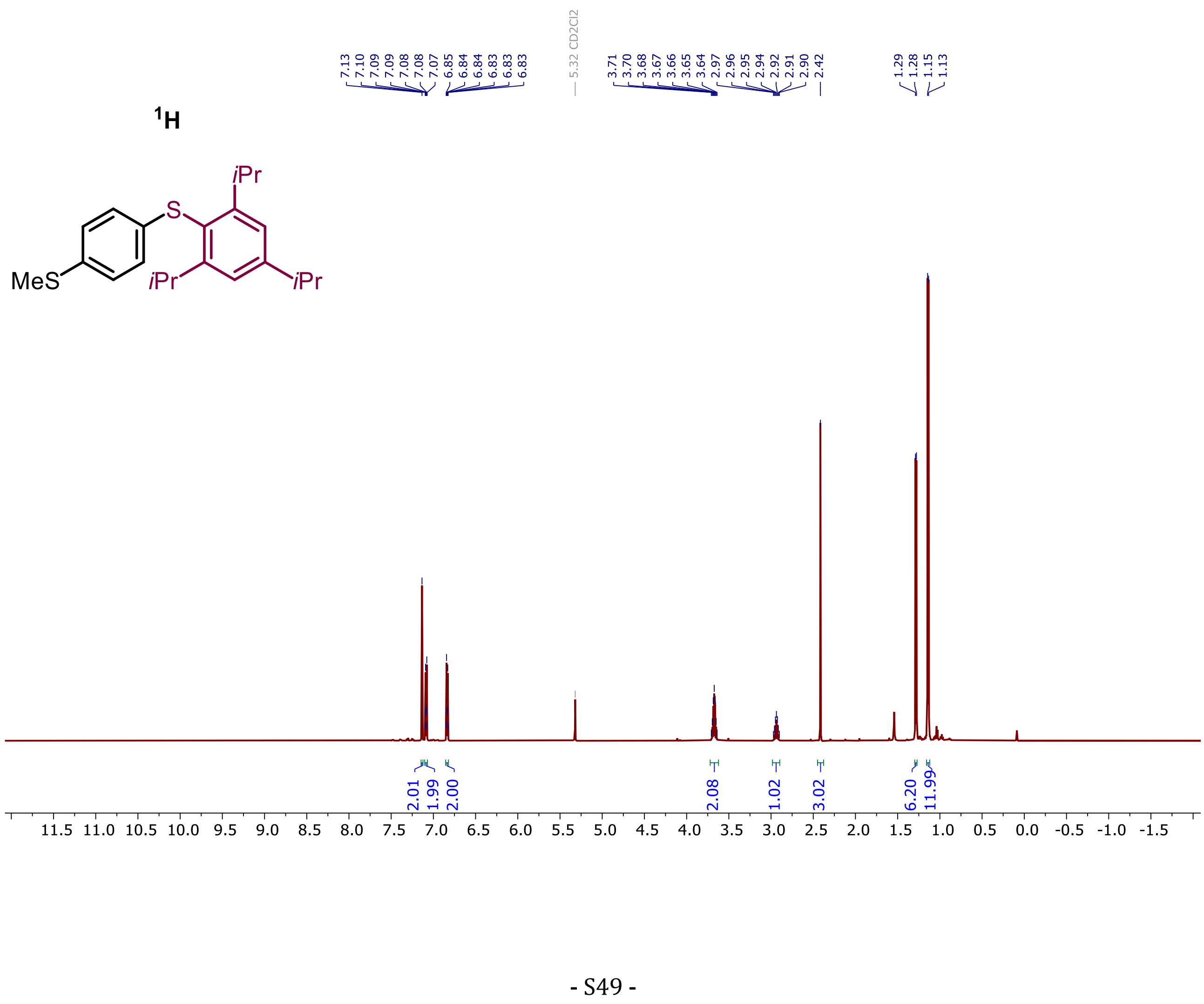

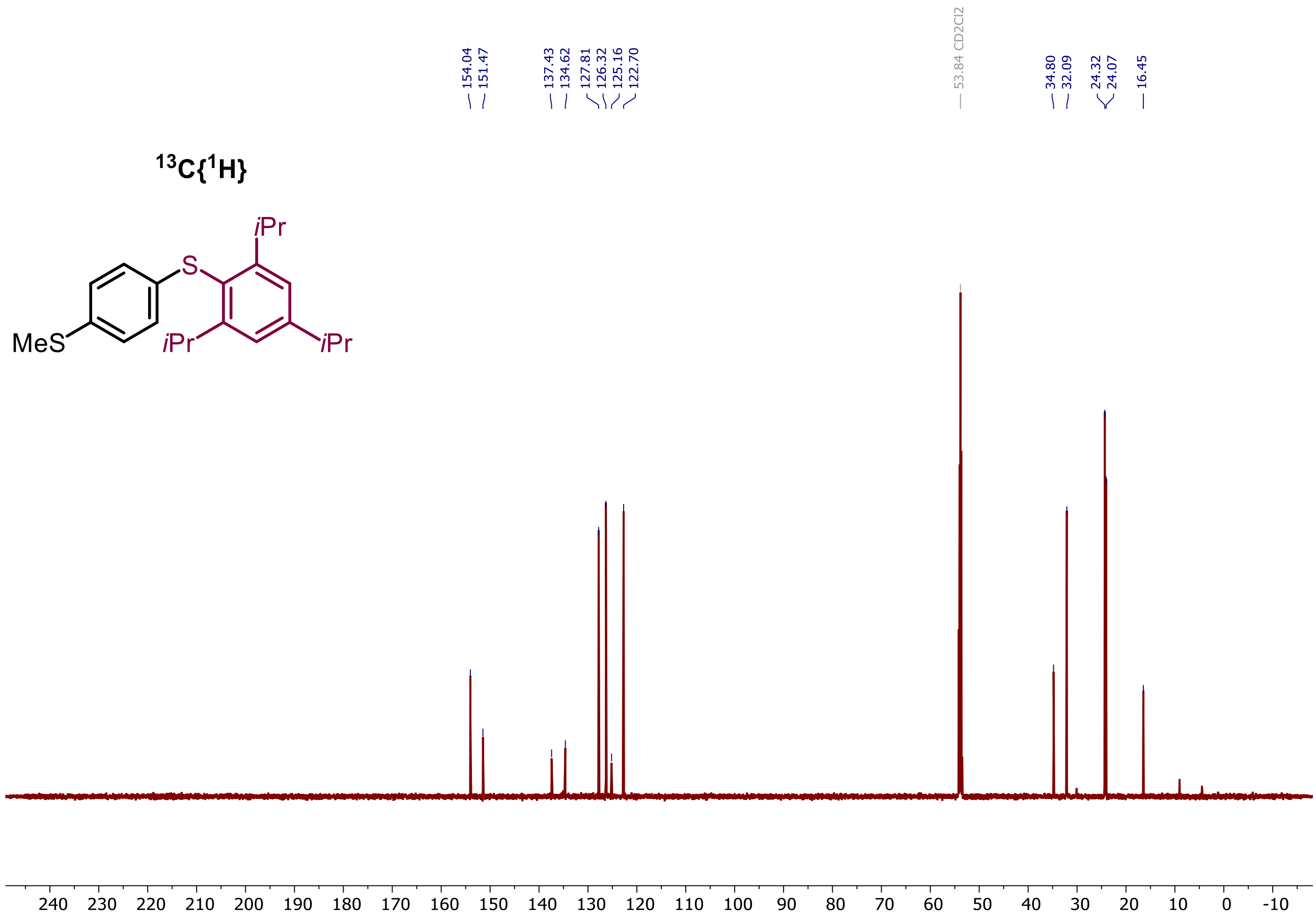

(4-Fluorophenyl)(2,4,6-triisopropylphenyl)sulfane (17)

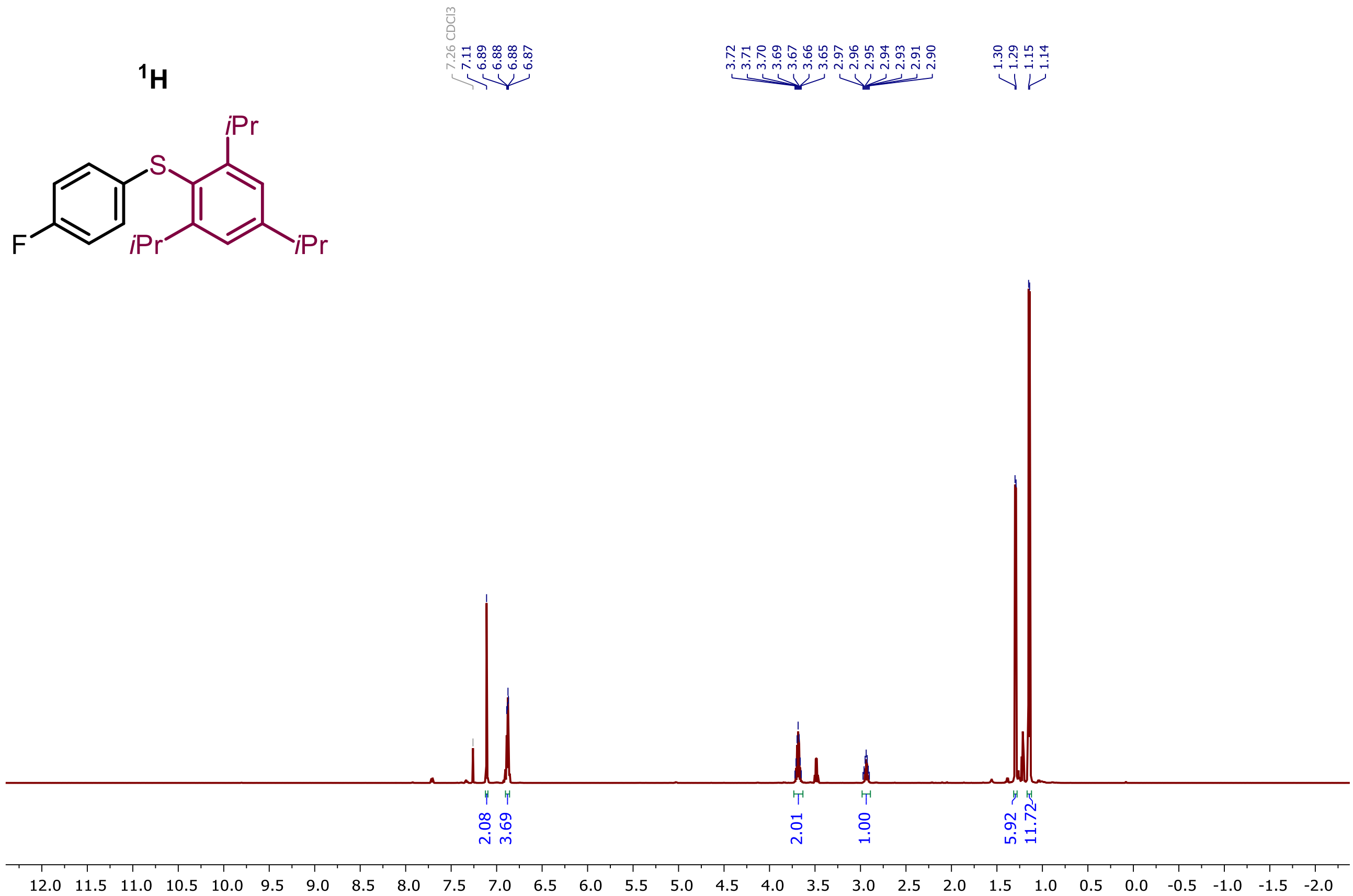

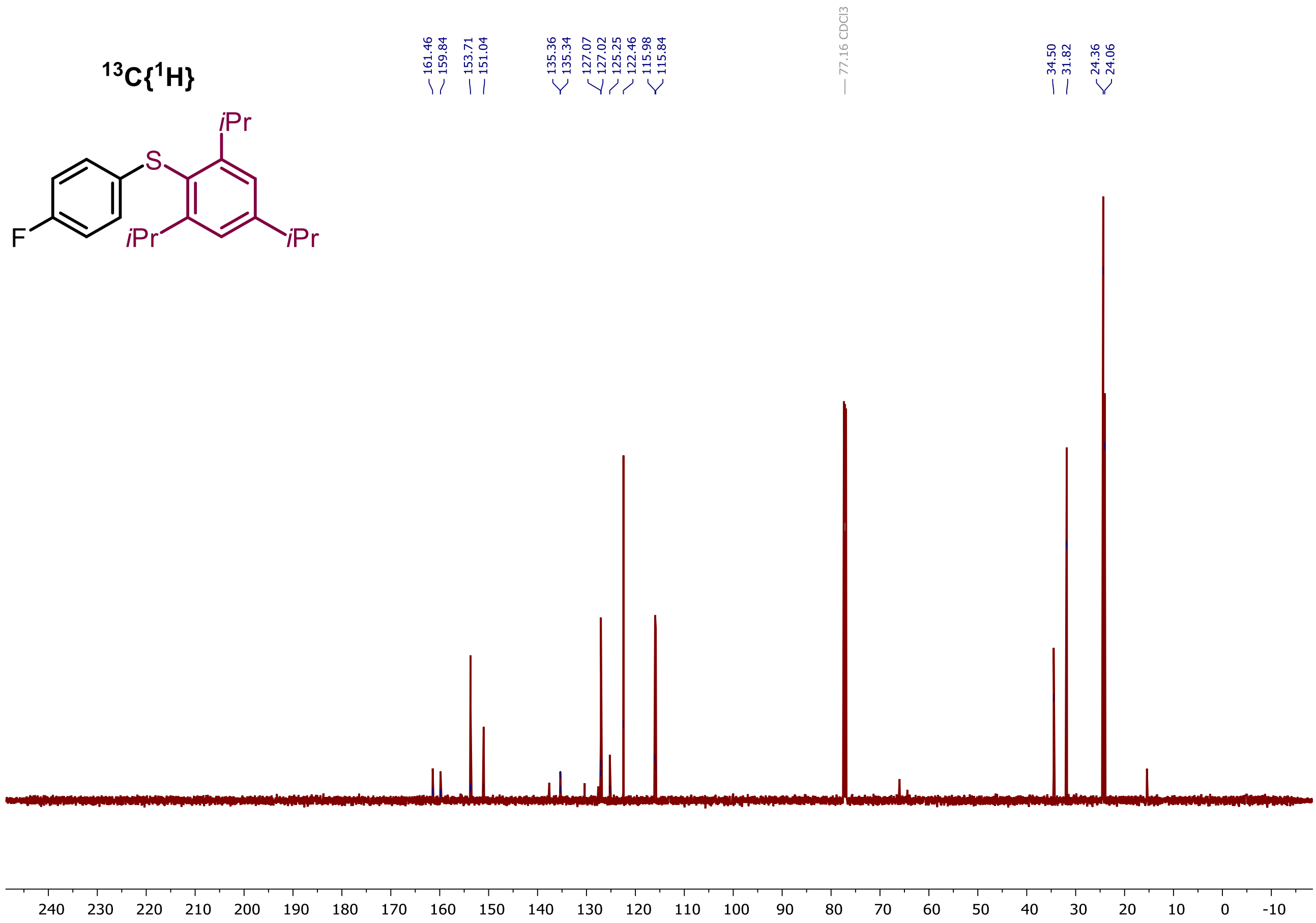

13C{1H}
iPr
S
F
iPr
iPr
161.46
159.84
153.71
151.04
135.36
135.34
127.07
127.02
125.25
122.46
115.98
115.84
77.16 CDCl3
34.50
31.82
24.36
24.06
240 230 220 210 200 190 180 170 160 150 140 130 120 110 100 90 80 70 60 50 40 30 20 10 0 -10

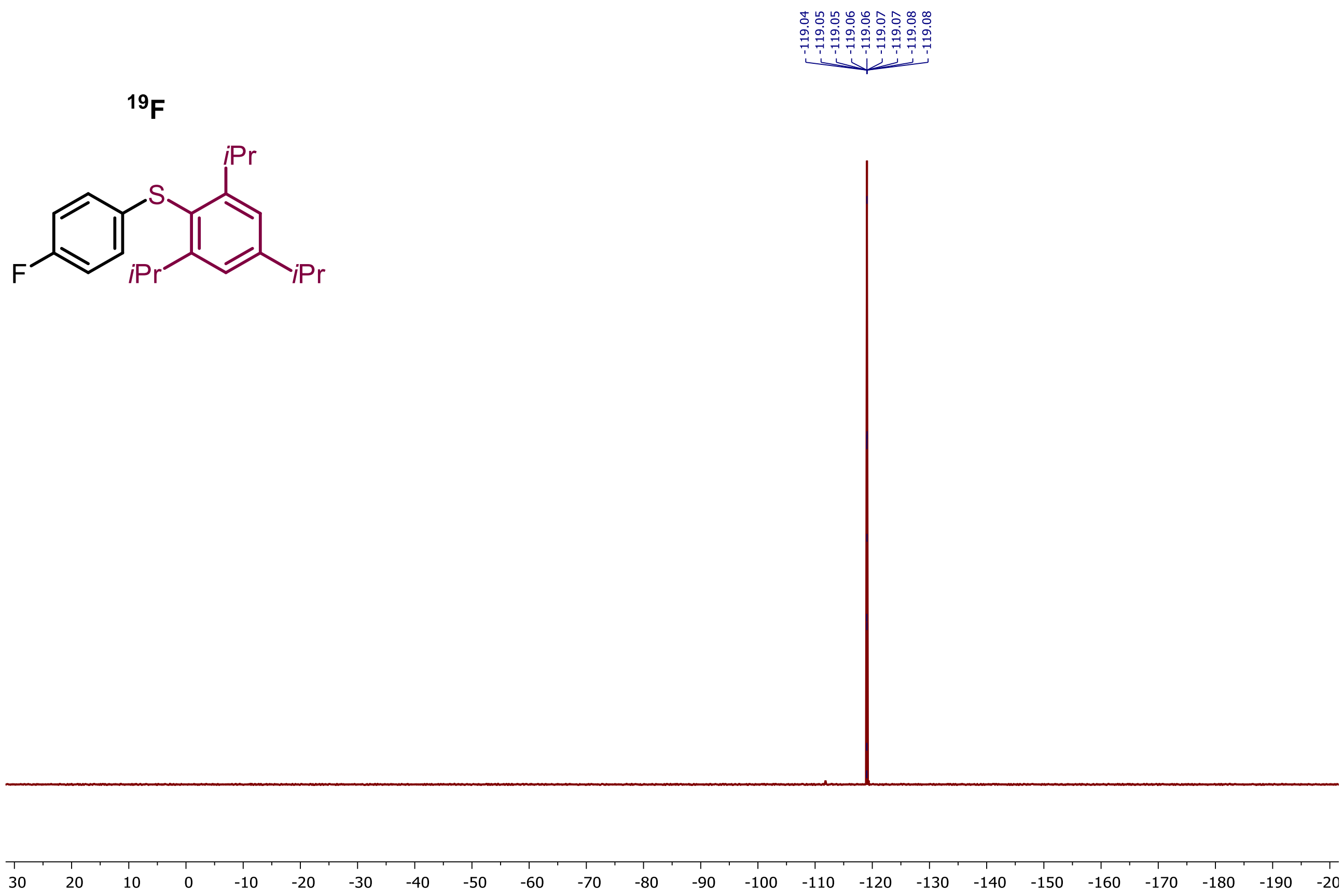

19F
iPr
S
F
iPr
iPr
-119.04
-119.05
-119.05
-119.06
-119.06
-119.07
-119.07
-119.08
-119.08
30 20 10 0 -10 -20 -30 -40 -50 -60 -70 -80 -90 -100 -110 -120 -130 -140 -150 -160 -170 -180 -190 -200

2-Methoxy-5-(o-tolylthio)benzonitrile (18)

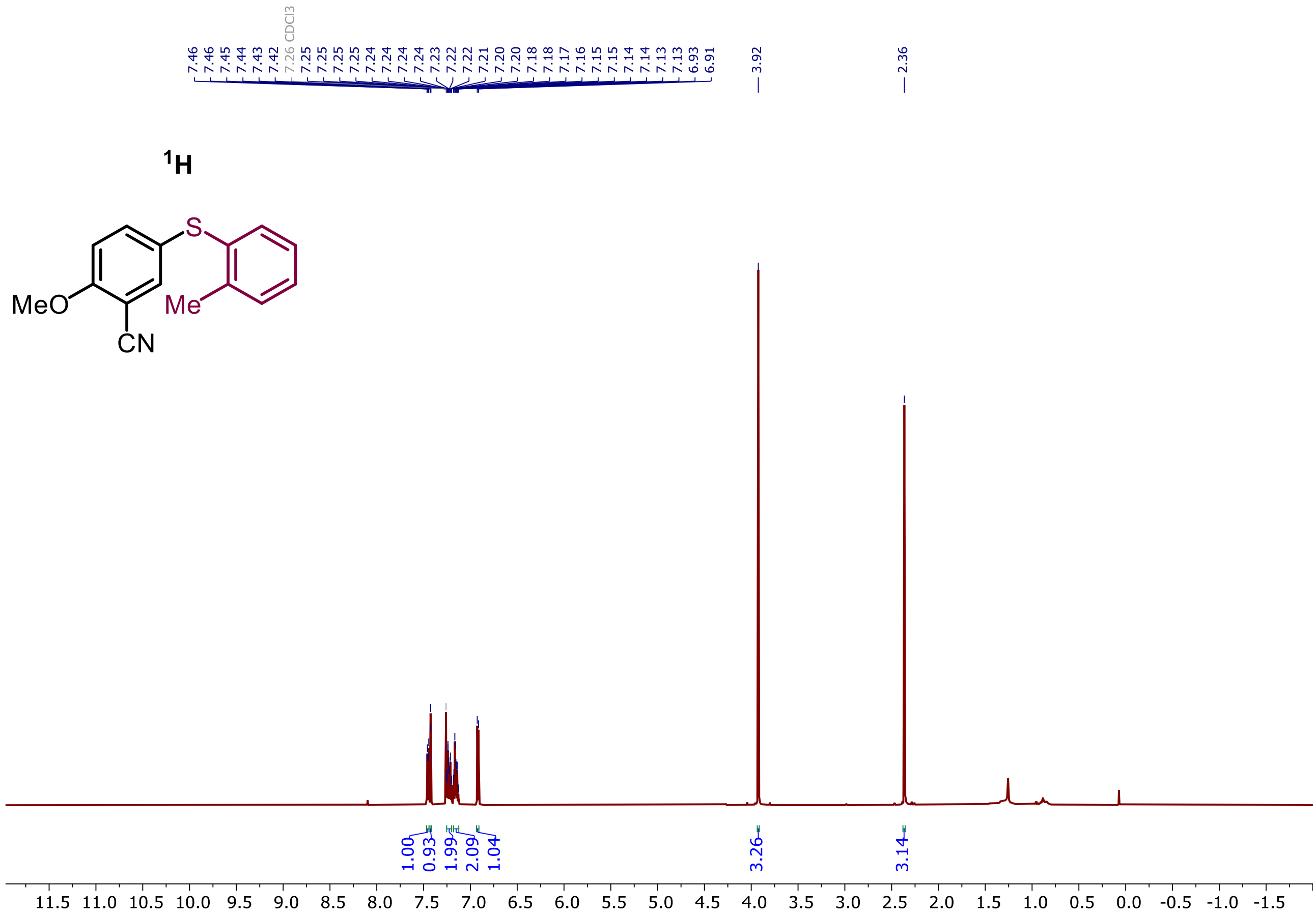

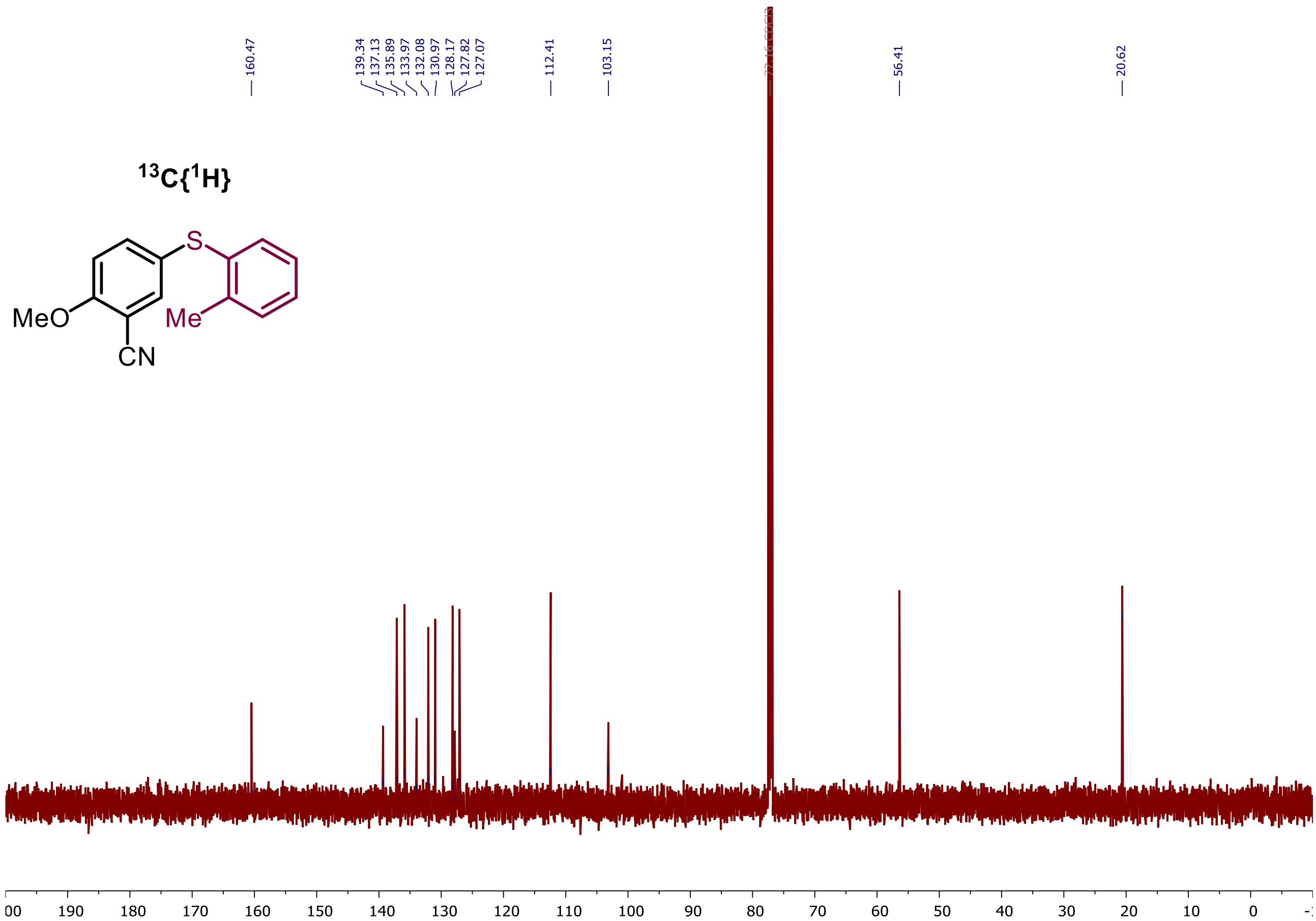

(4-Fluorophenyl)(mesityl)sulfane (19)

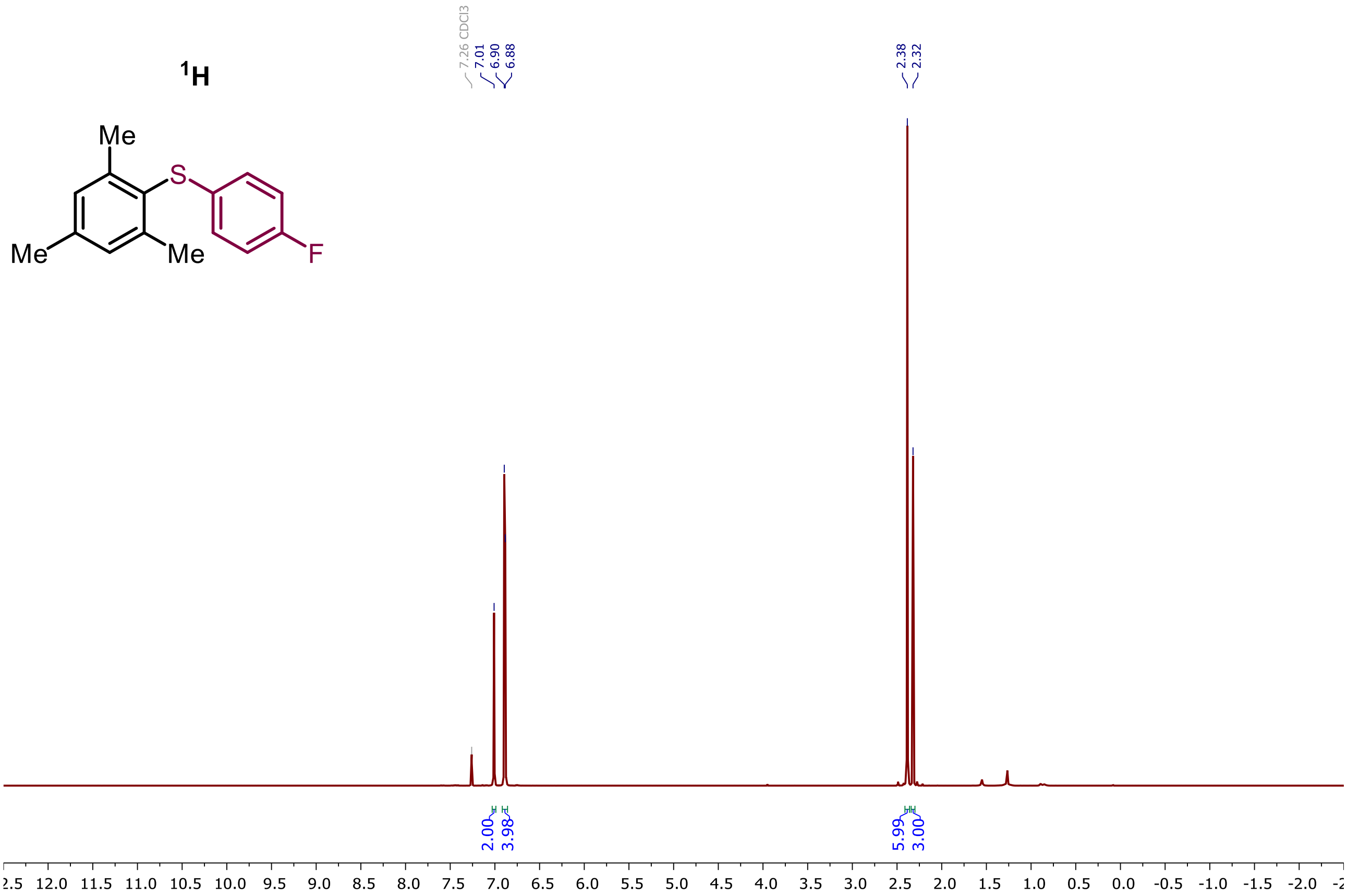

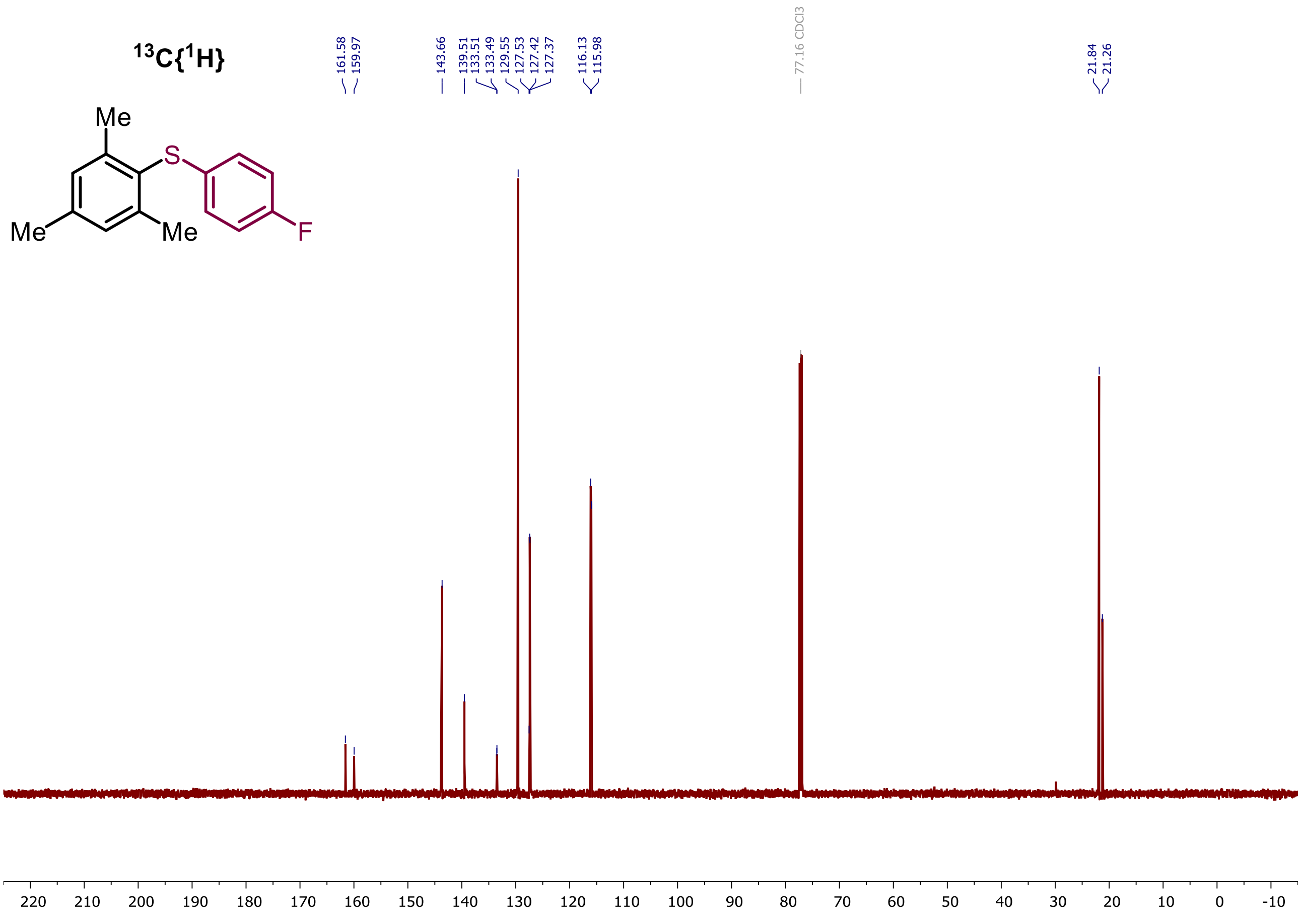

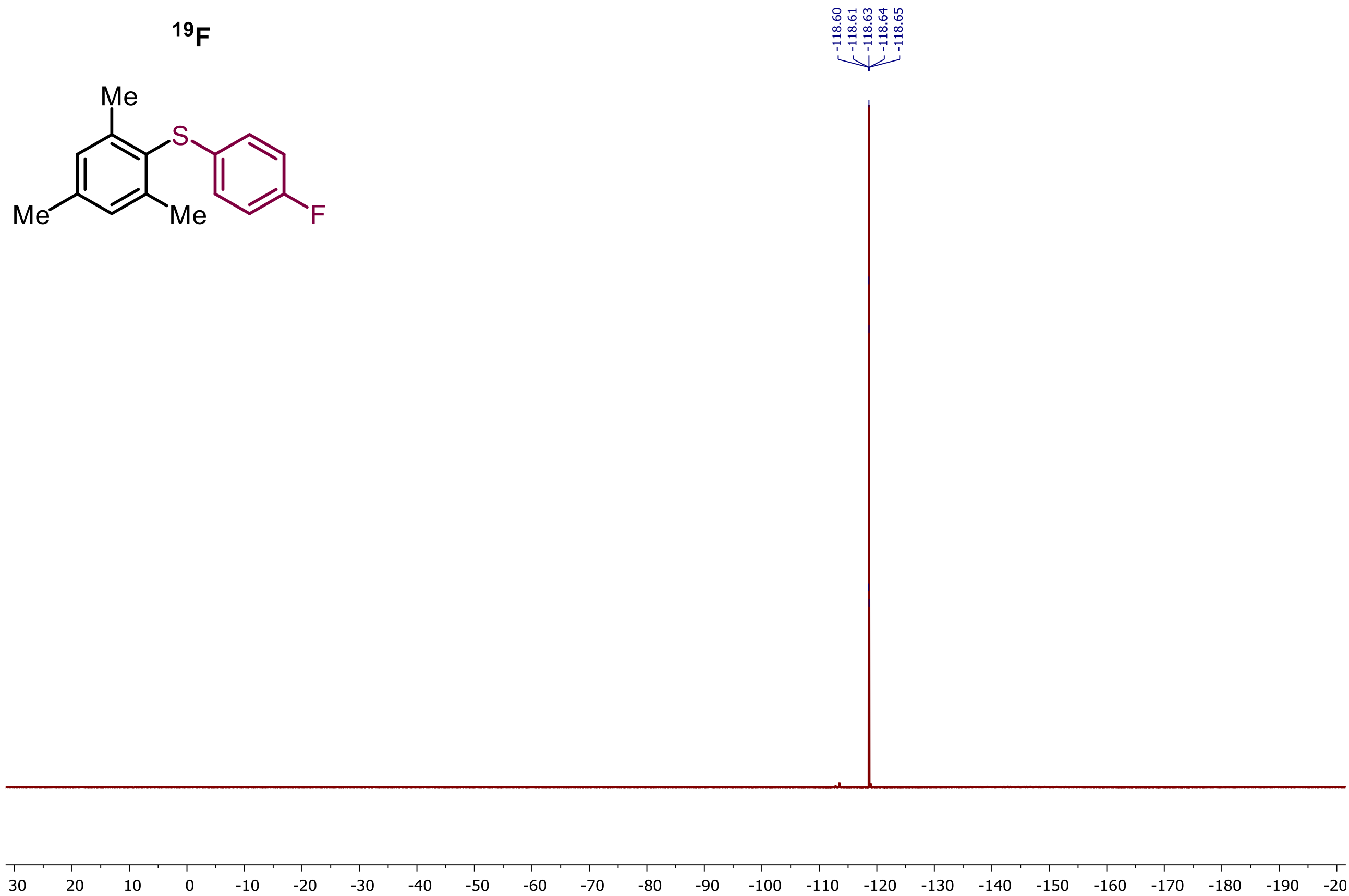

2-((4-Fluorophenyl)thio)furane (20)

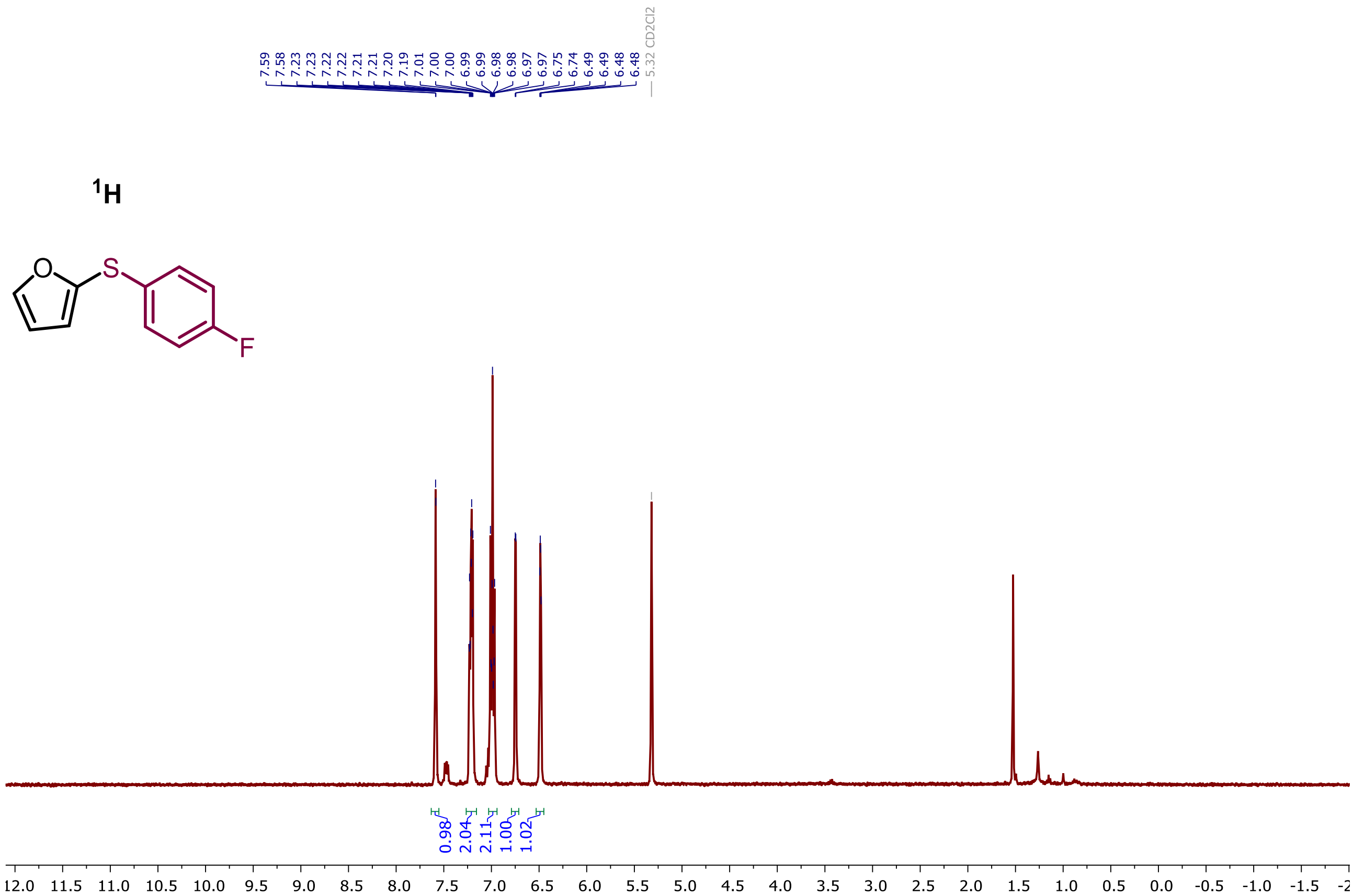

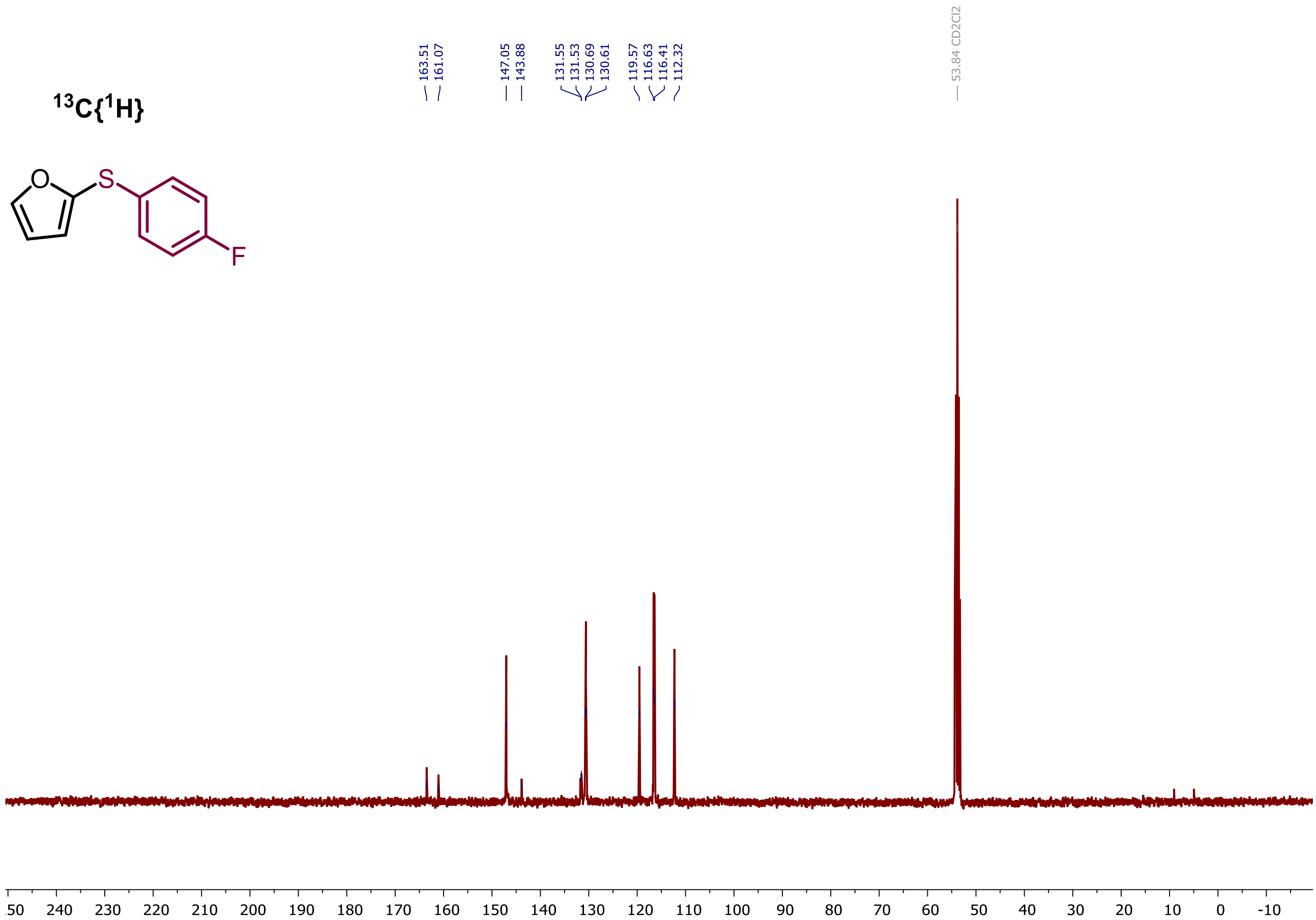
13C{1H}
163.51
161.07
147.05
143.88
131.55
131.53
130.69
130.61
119.57
116.63
116.41
112.32
53.84 CD2Cl2
S
O
F

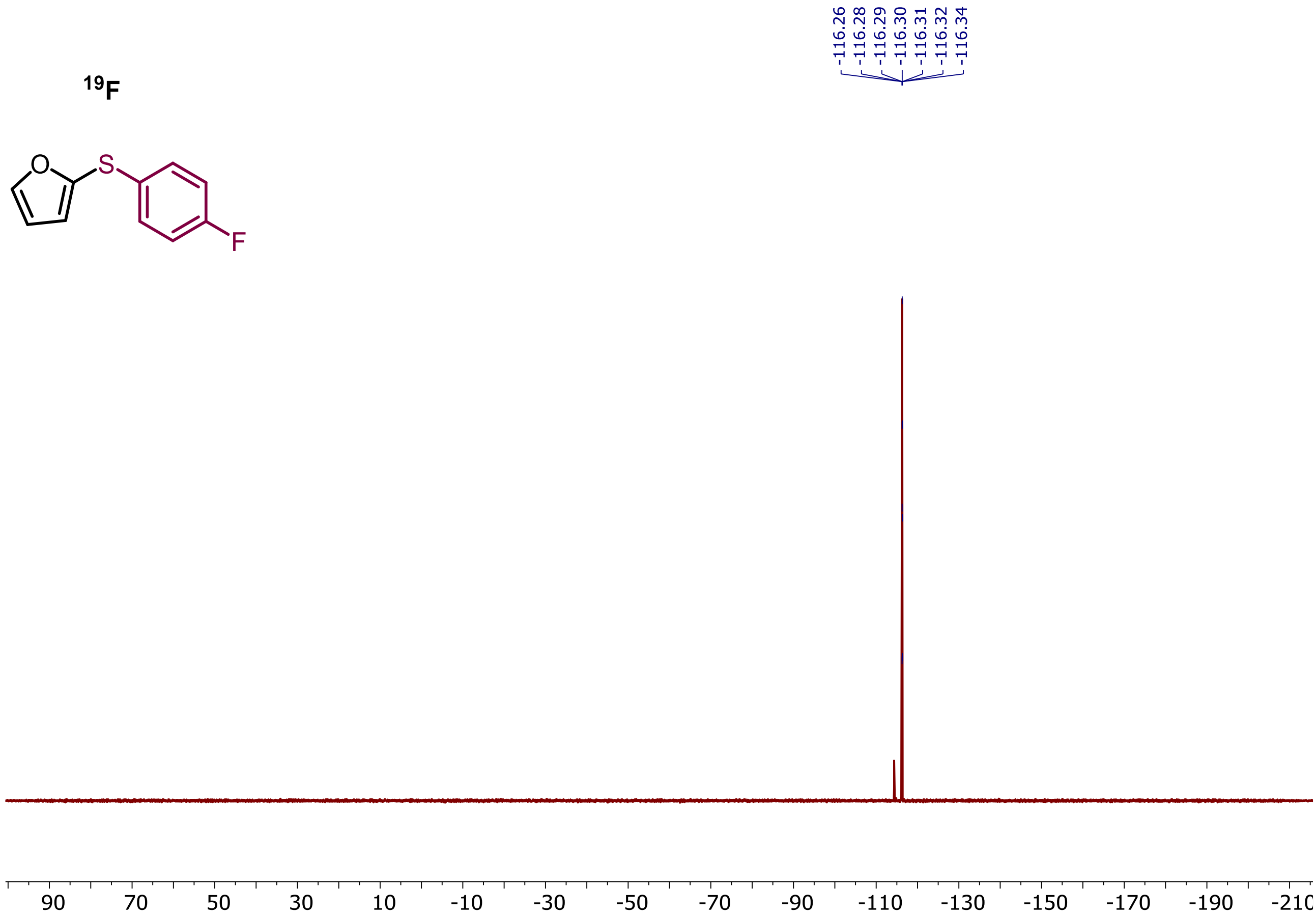
19F
-116.26
-116.28
-116.29
-116.30
-116.31
-116.32
-116.34
S
O
F

(4-Fluorophenyl)(phenyl)selane (21)

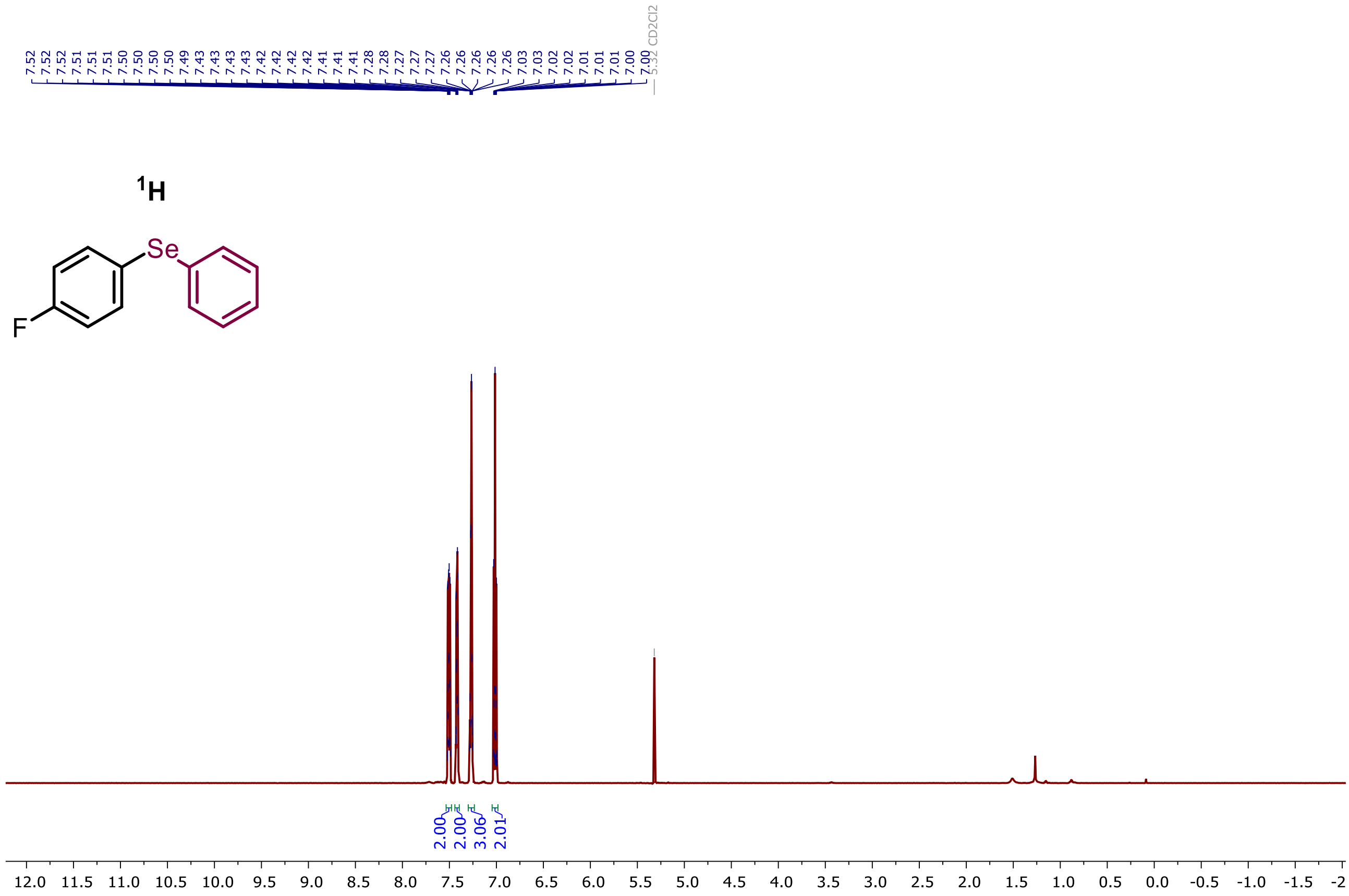

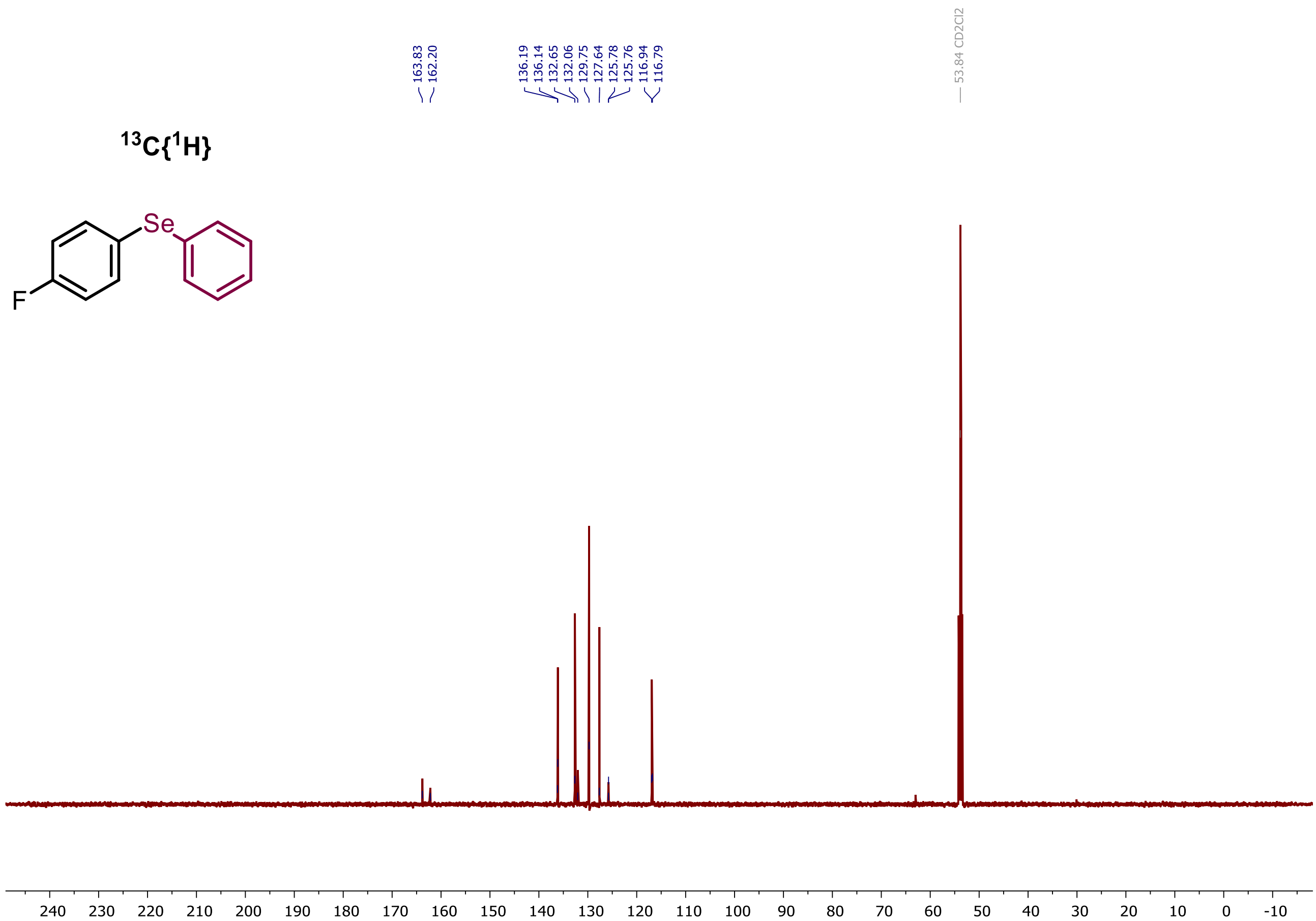

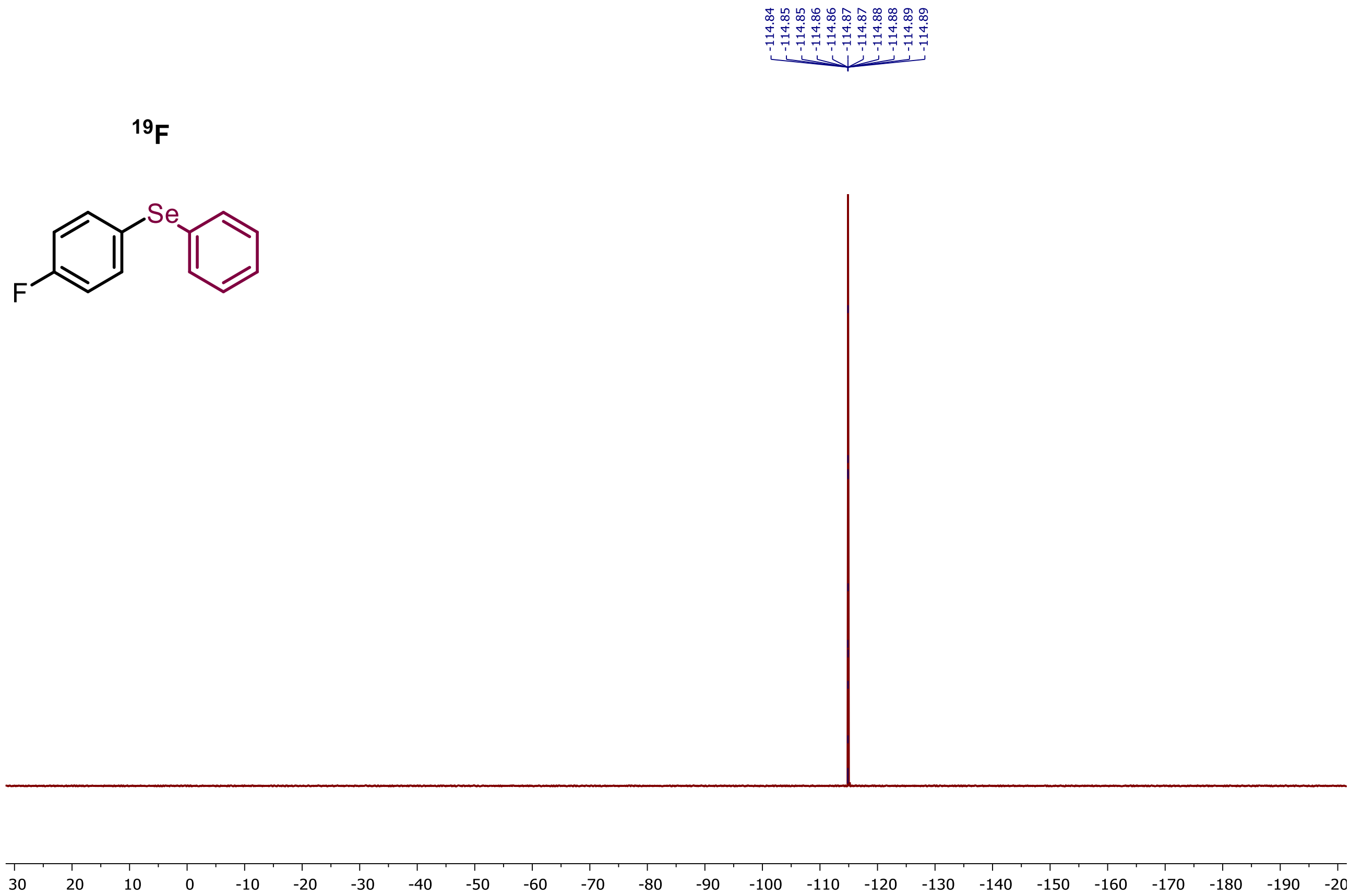

Hexyl(4-methoxyphenyl)sulfane (22)

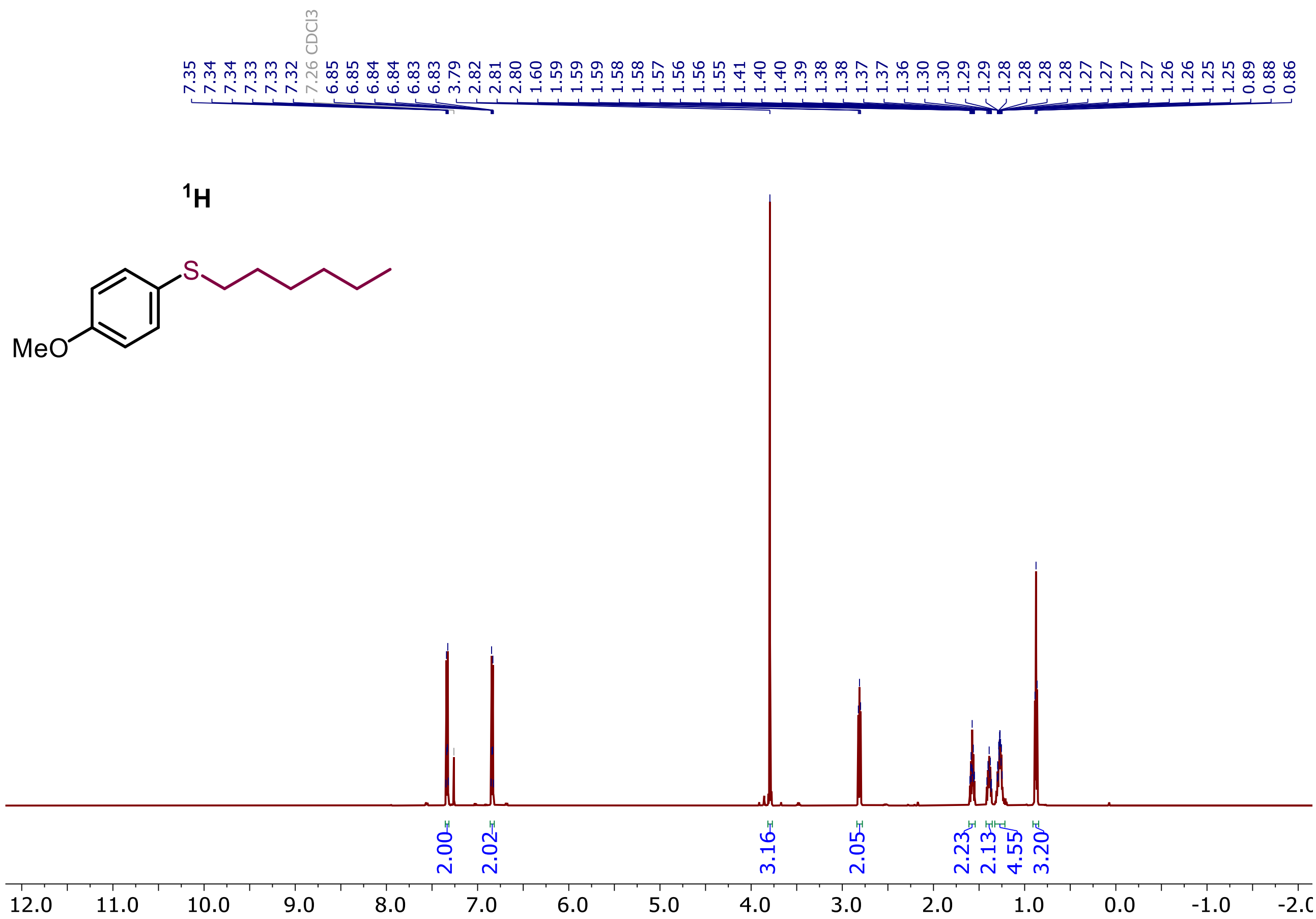

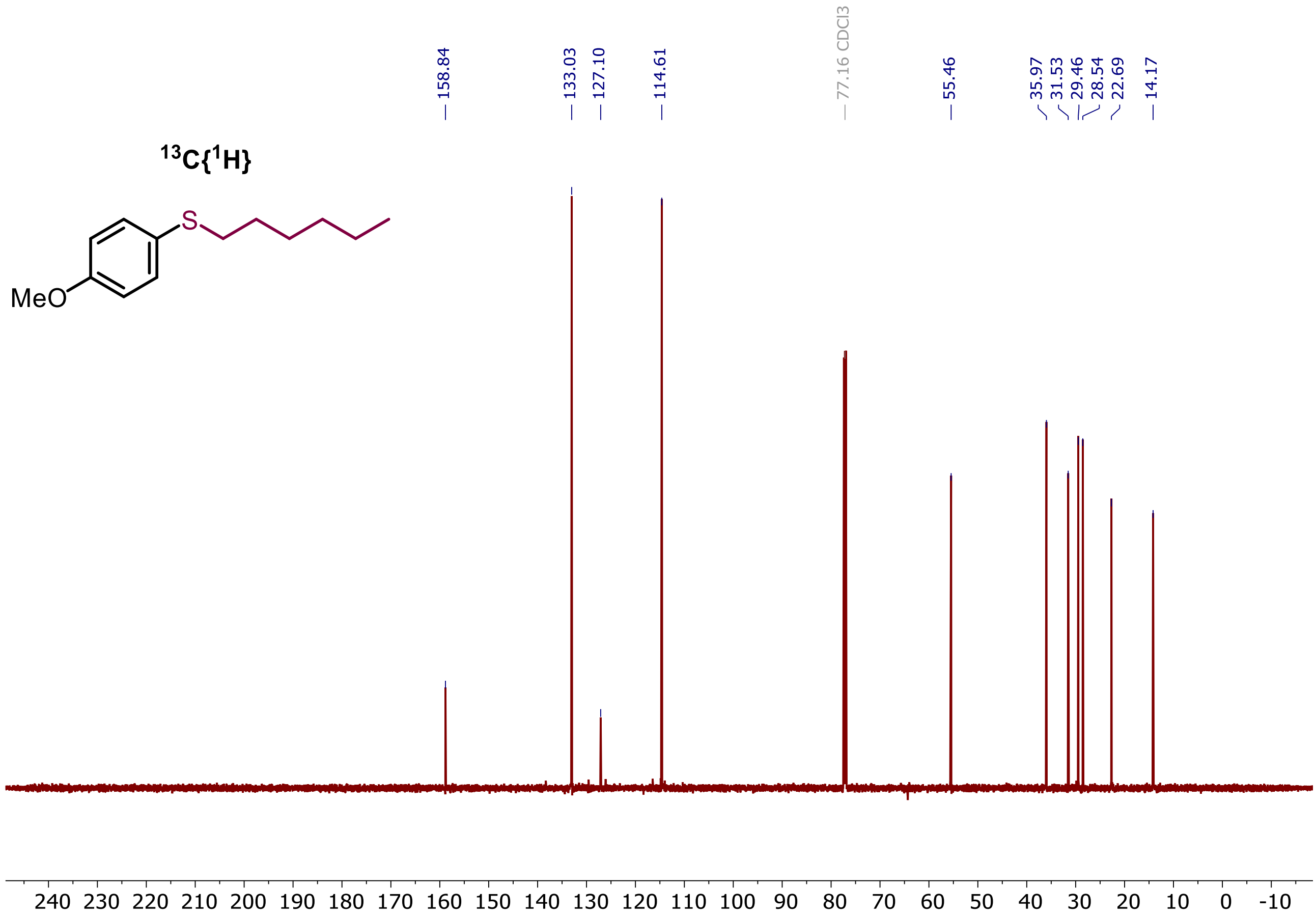

(4-methoxyphenyl)(phenethyl)sulfane (23)

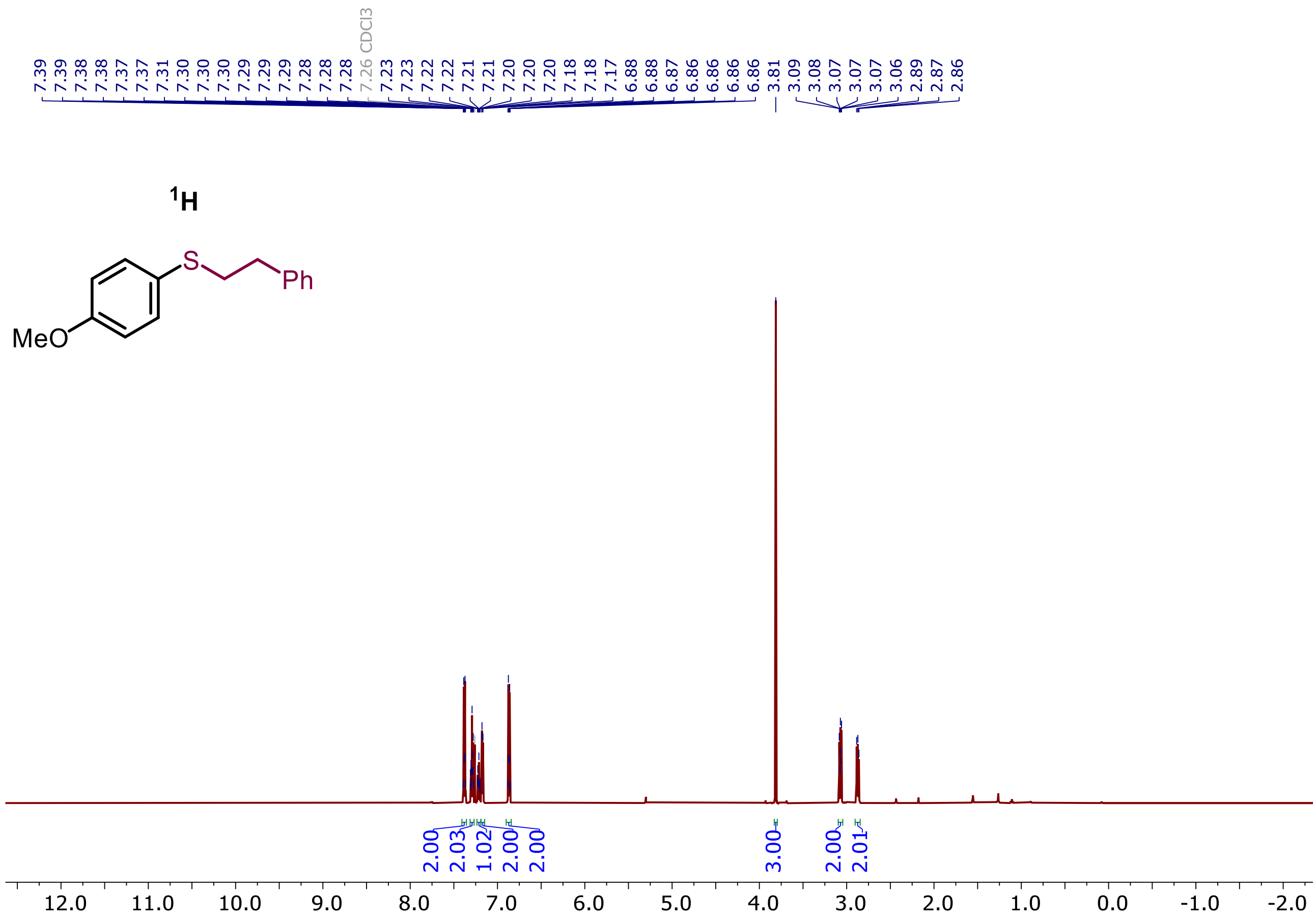

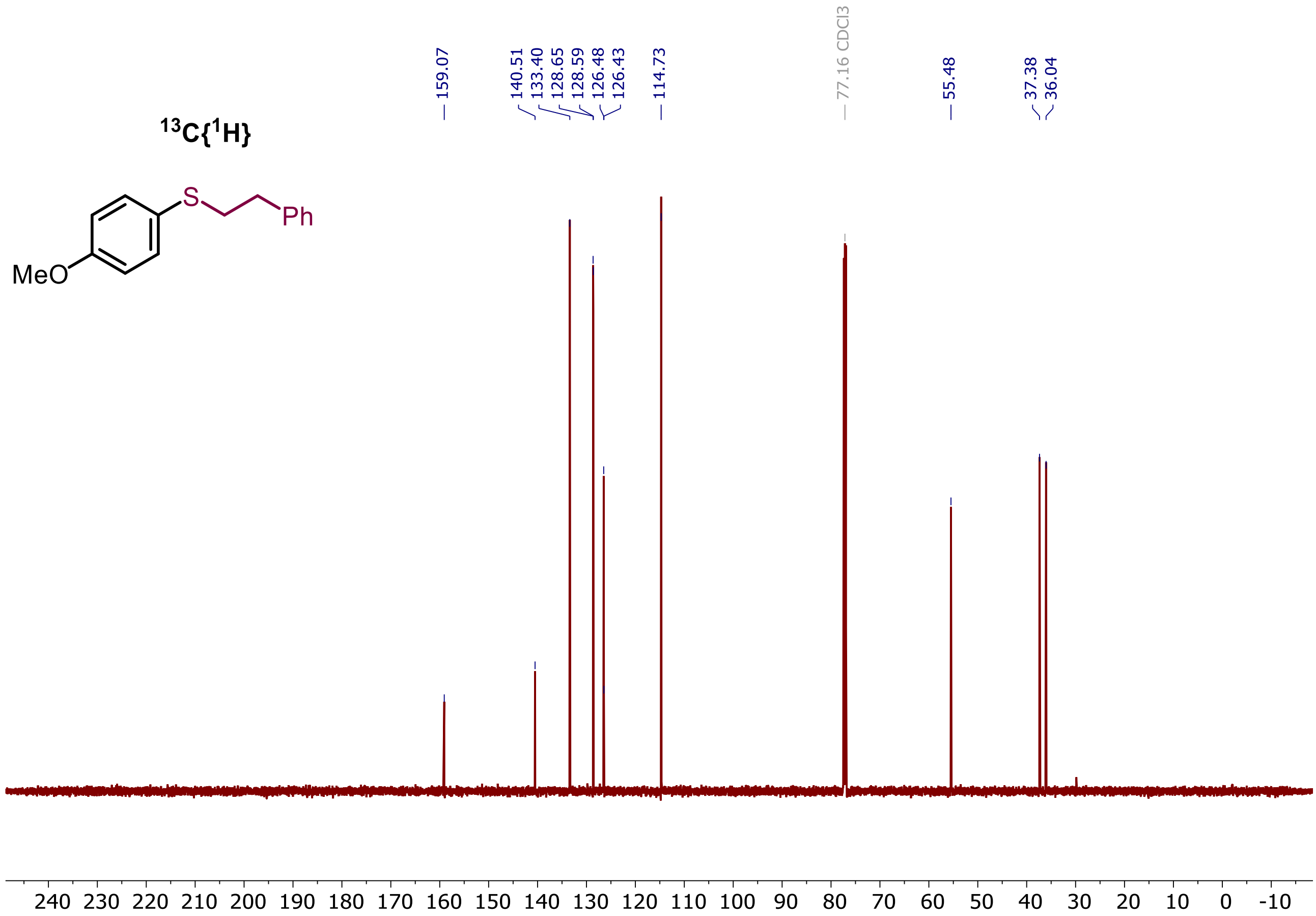

Methyl(4-(phenethylthio)phenyl)sulfane (24)

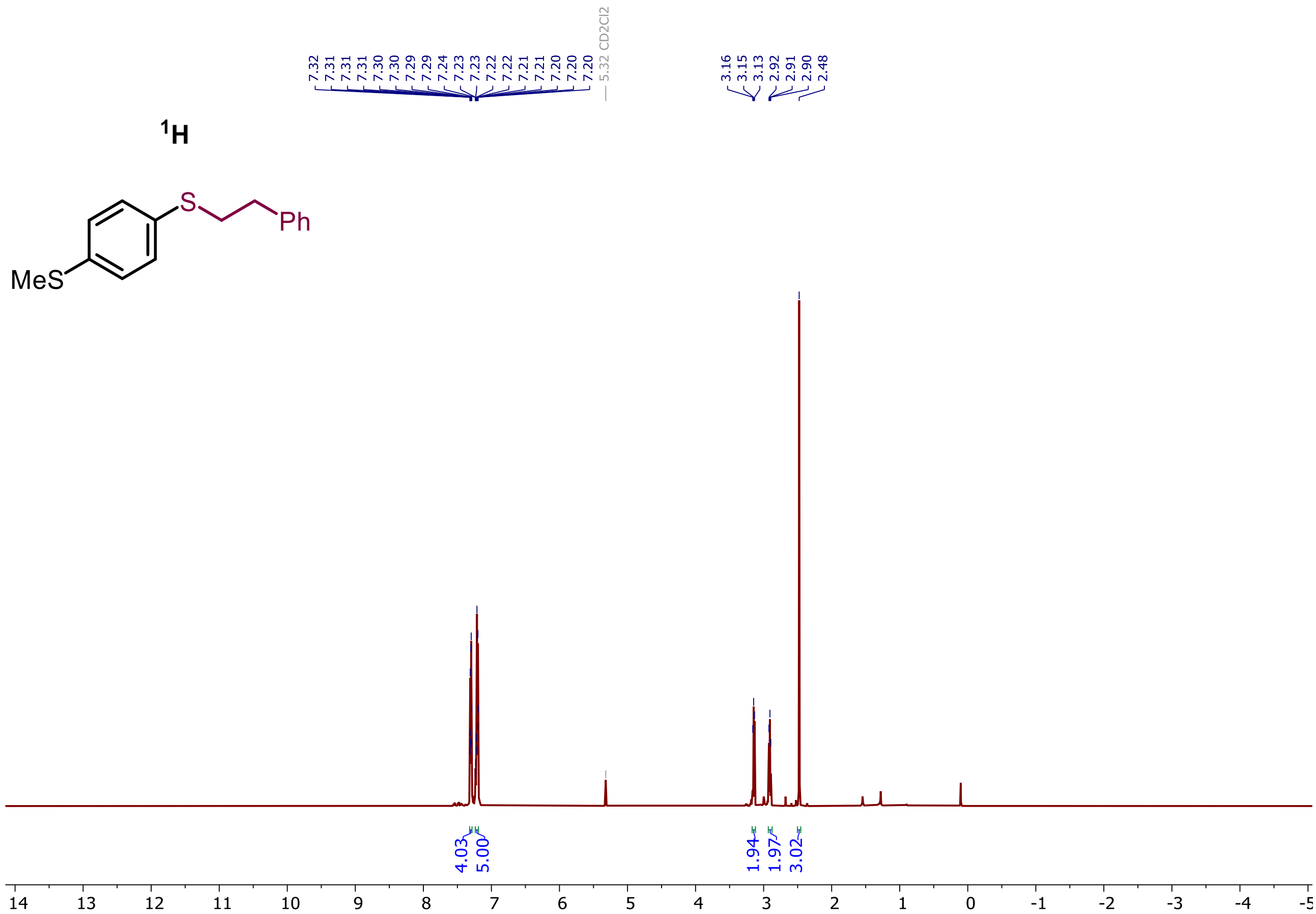

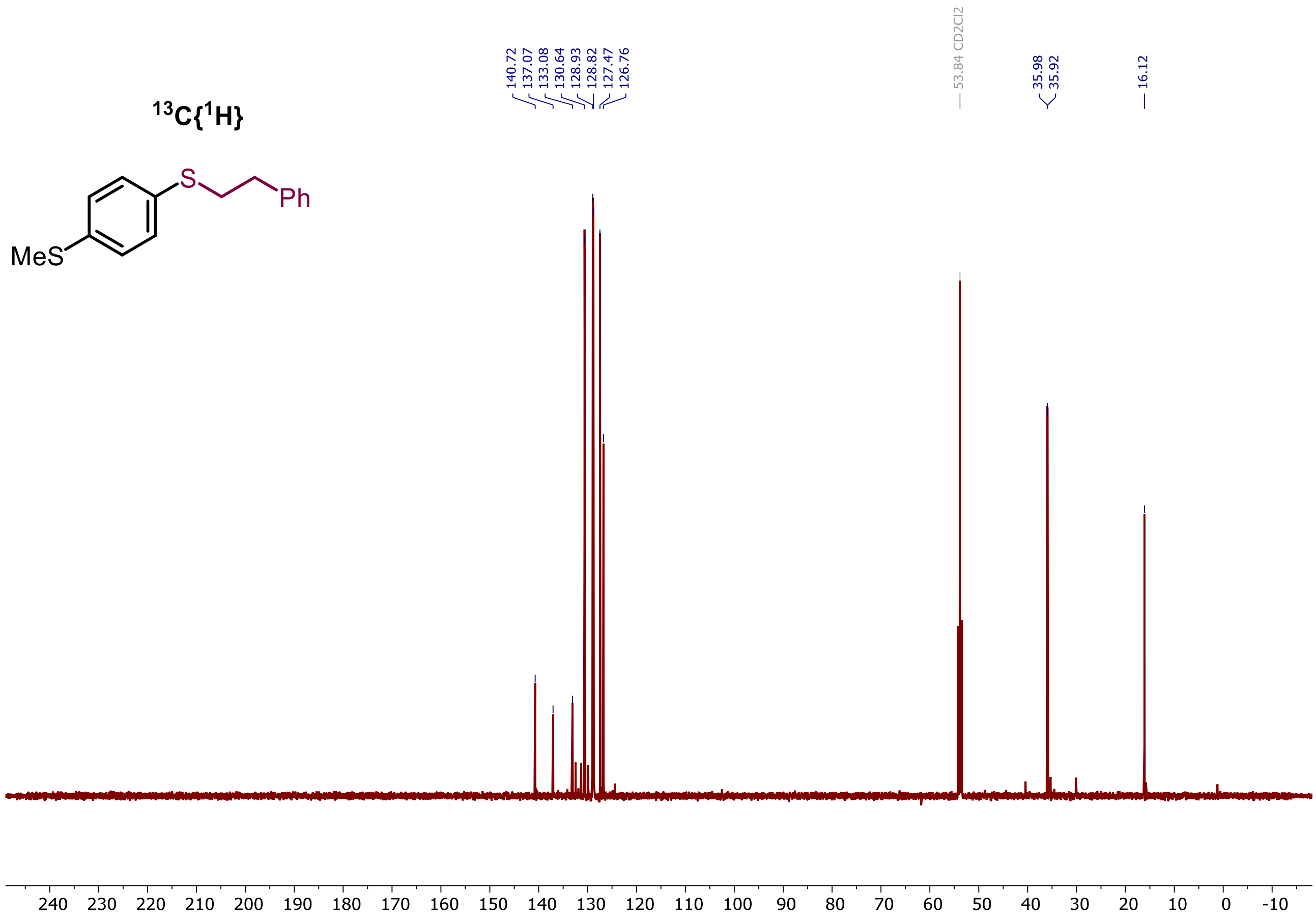

(4-(*tert*-Butyl)phenyl)(phenethyl)sulfane (25)

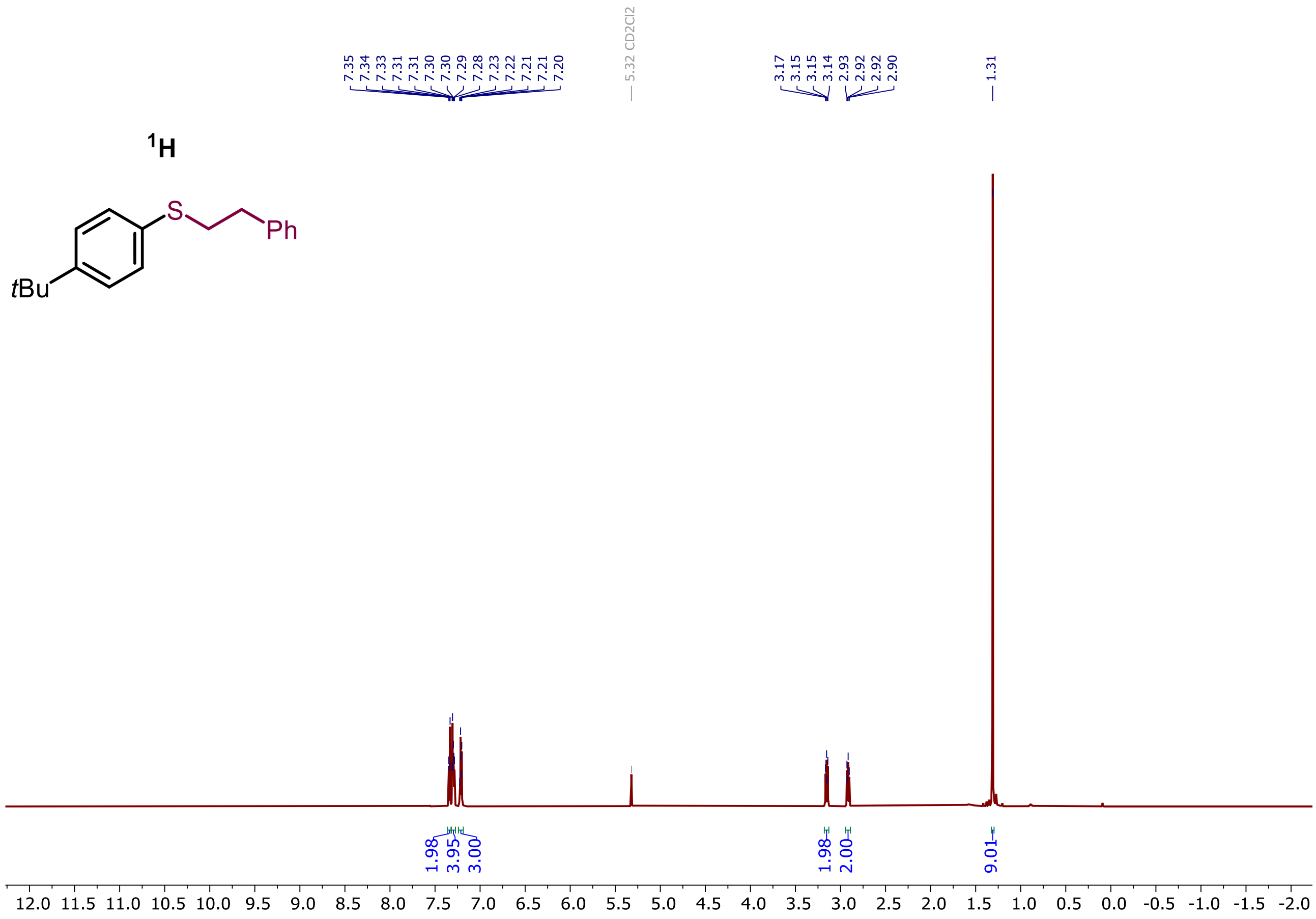

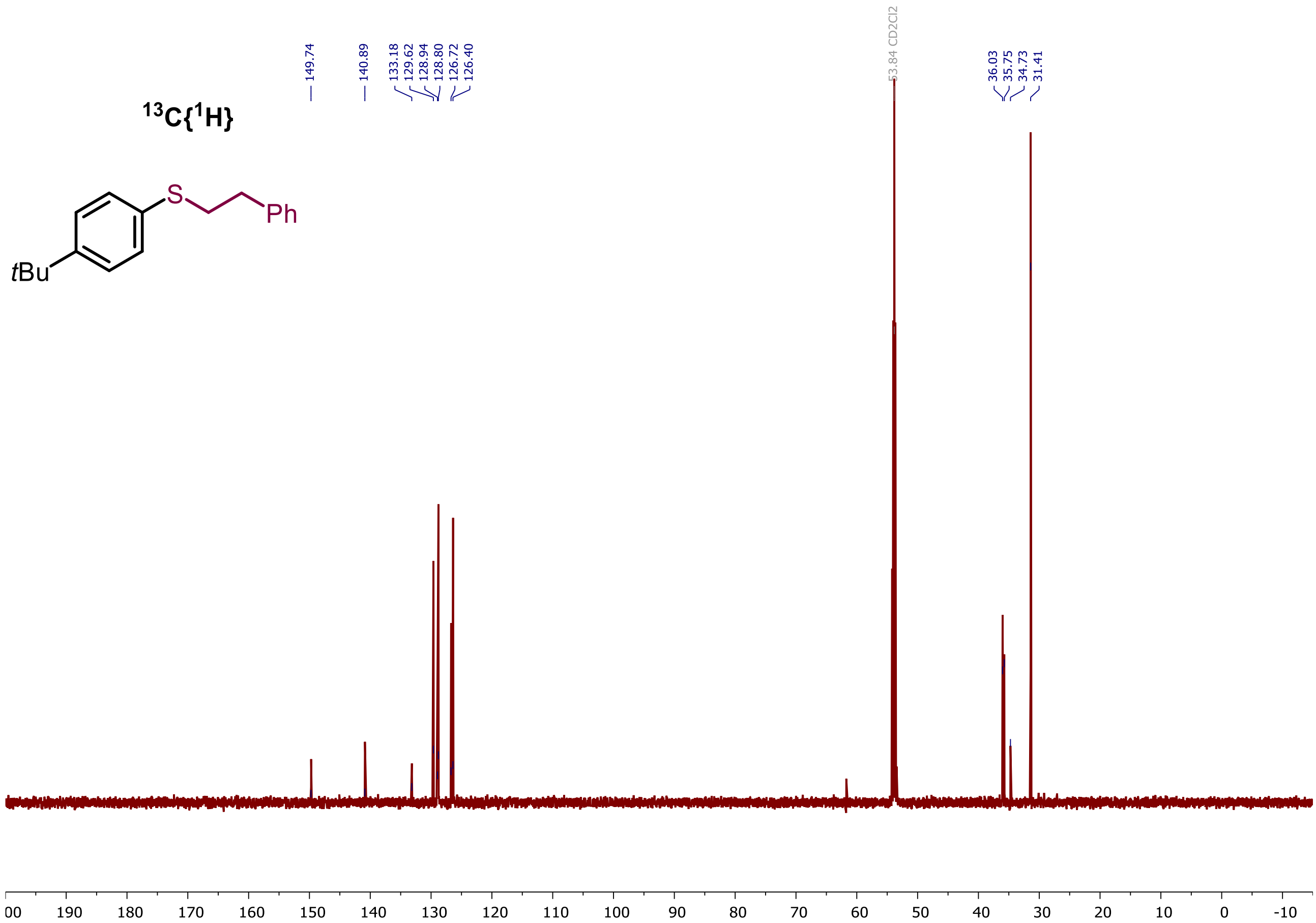

Mesityl(phenethyl)sulfane (26)

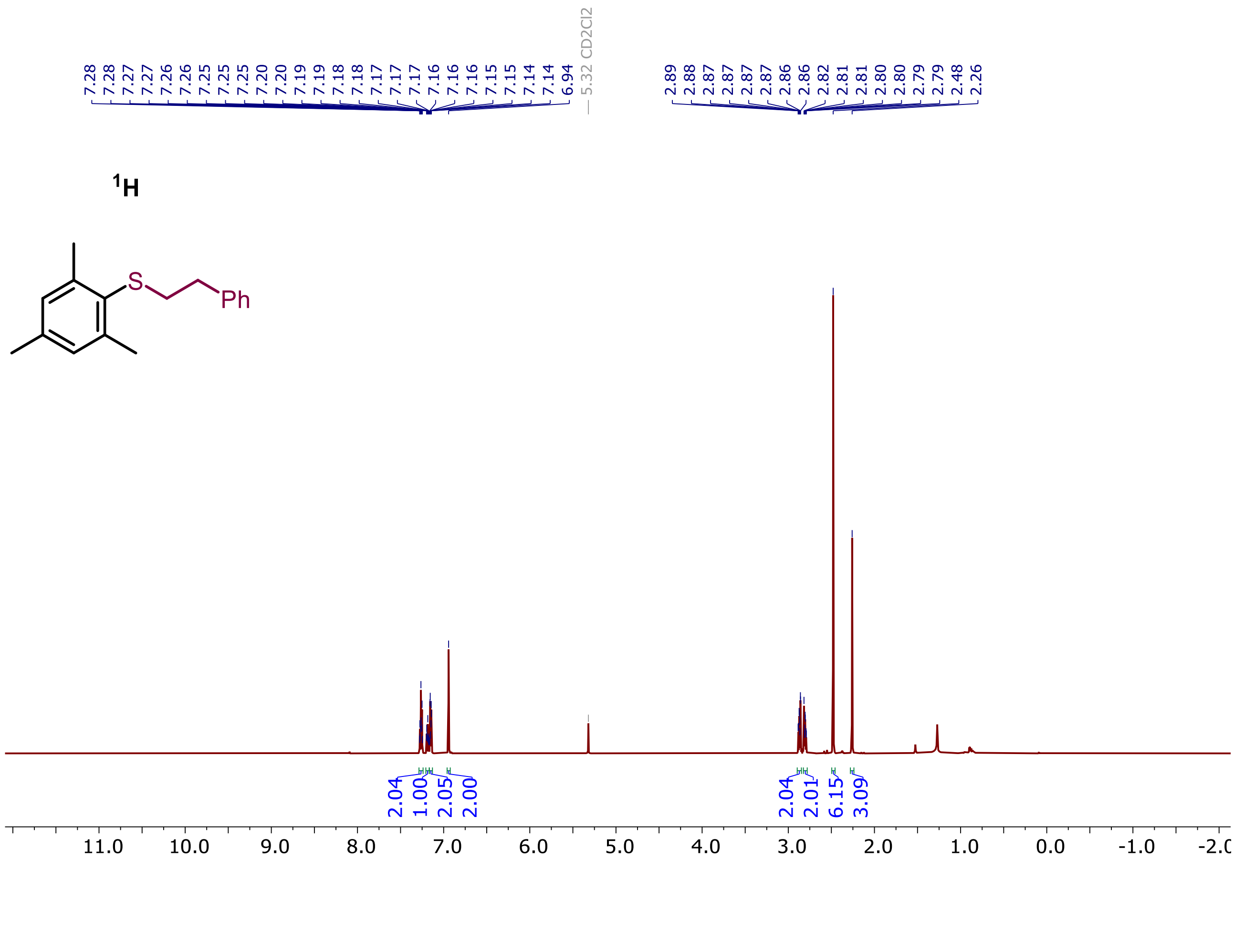

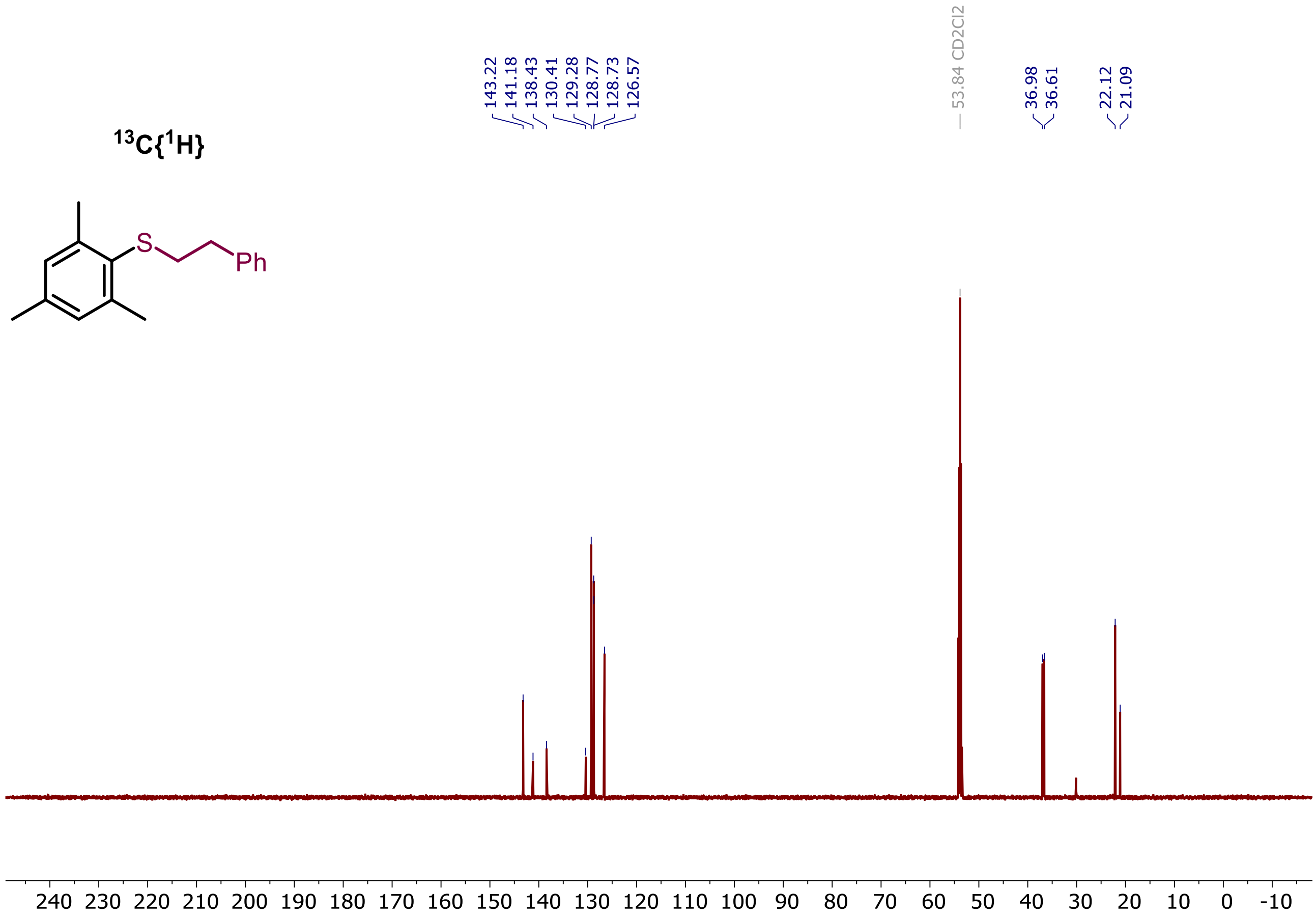

Methyl ((*S*)-3-((4-fluorophenyl)thio)-2-methylpropanoyl)-L-prolinate (27)

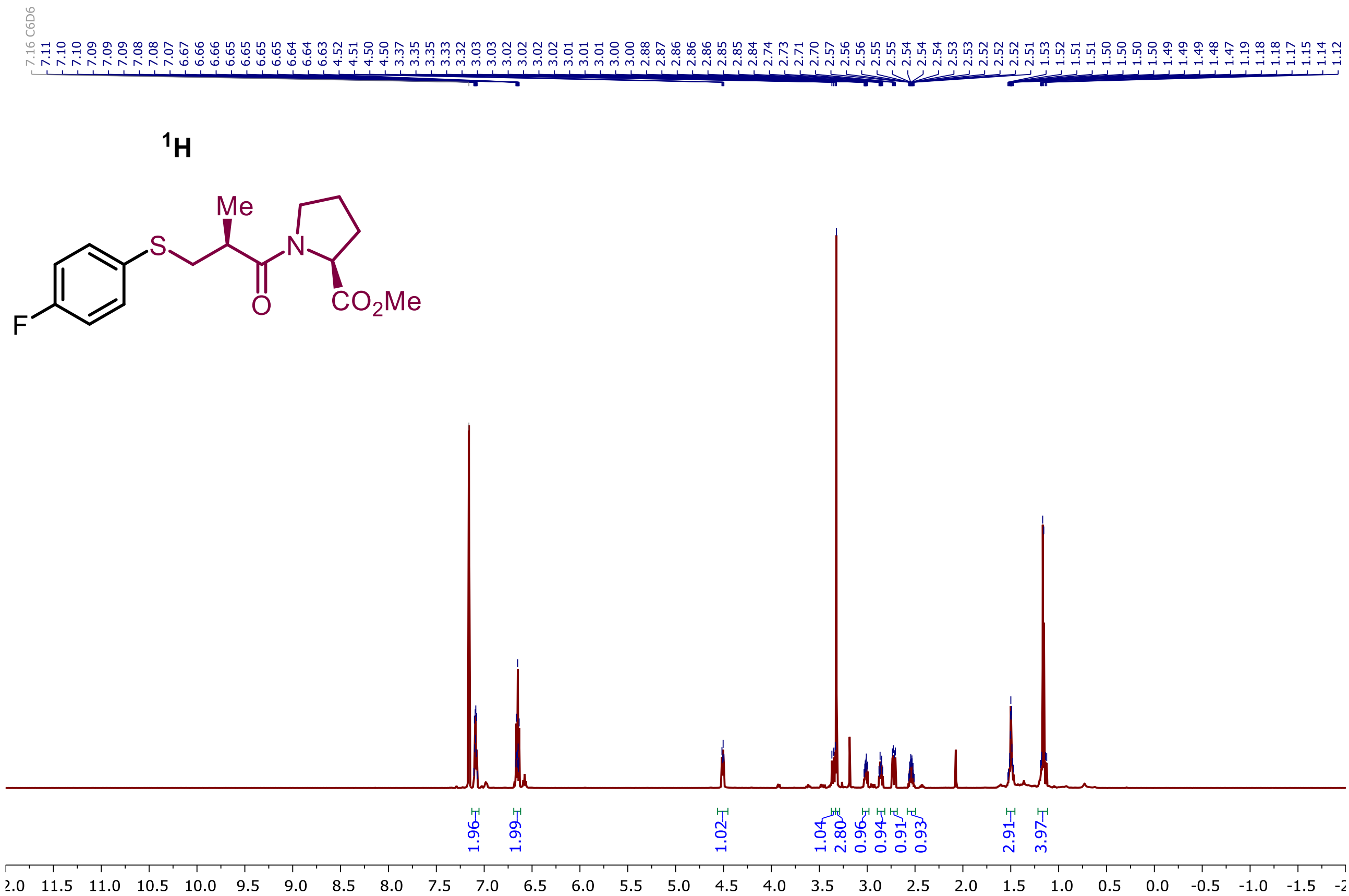

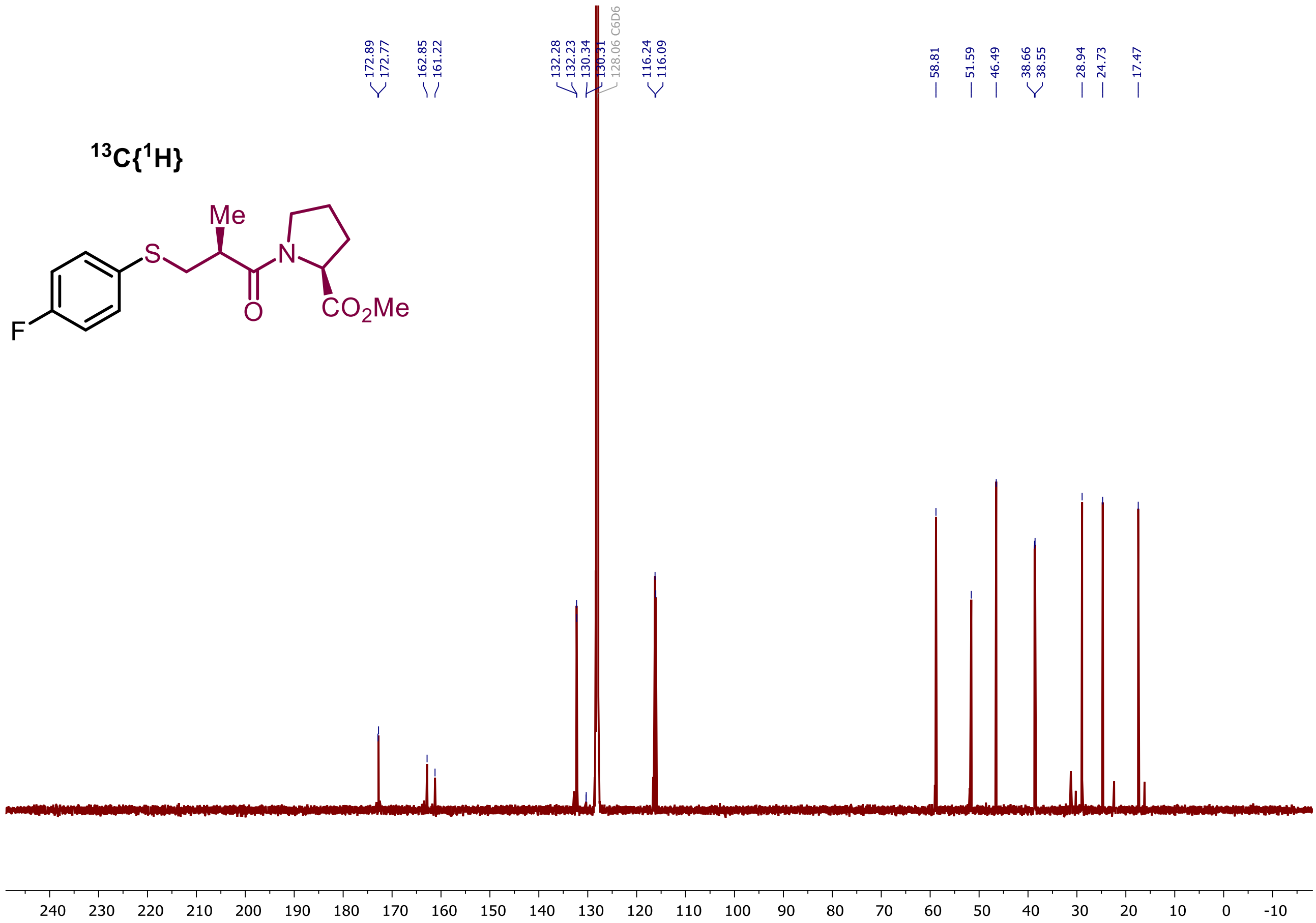
13C{1H}
172.89
172.77
162.85
161.22
132.28
132.23
130.34
130.31
128.06 C6D6
116.24
116.09
58.81
51.59
46.49
38.66
38.55
28.94
24.73
17.47
Me
S
N
F
O
CO2Me
240 230 220 210 200 190 180 170 160 150 140 130 120 110 100 90 80 70 60 50 40 30 20 10 0 -10

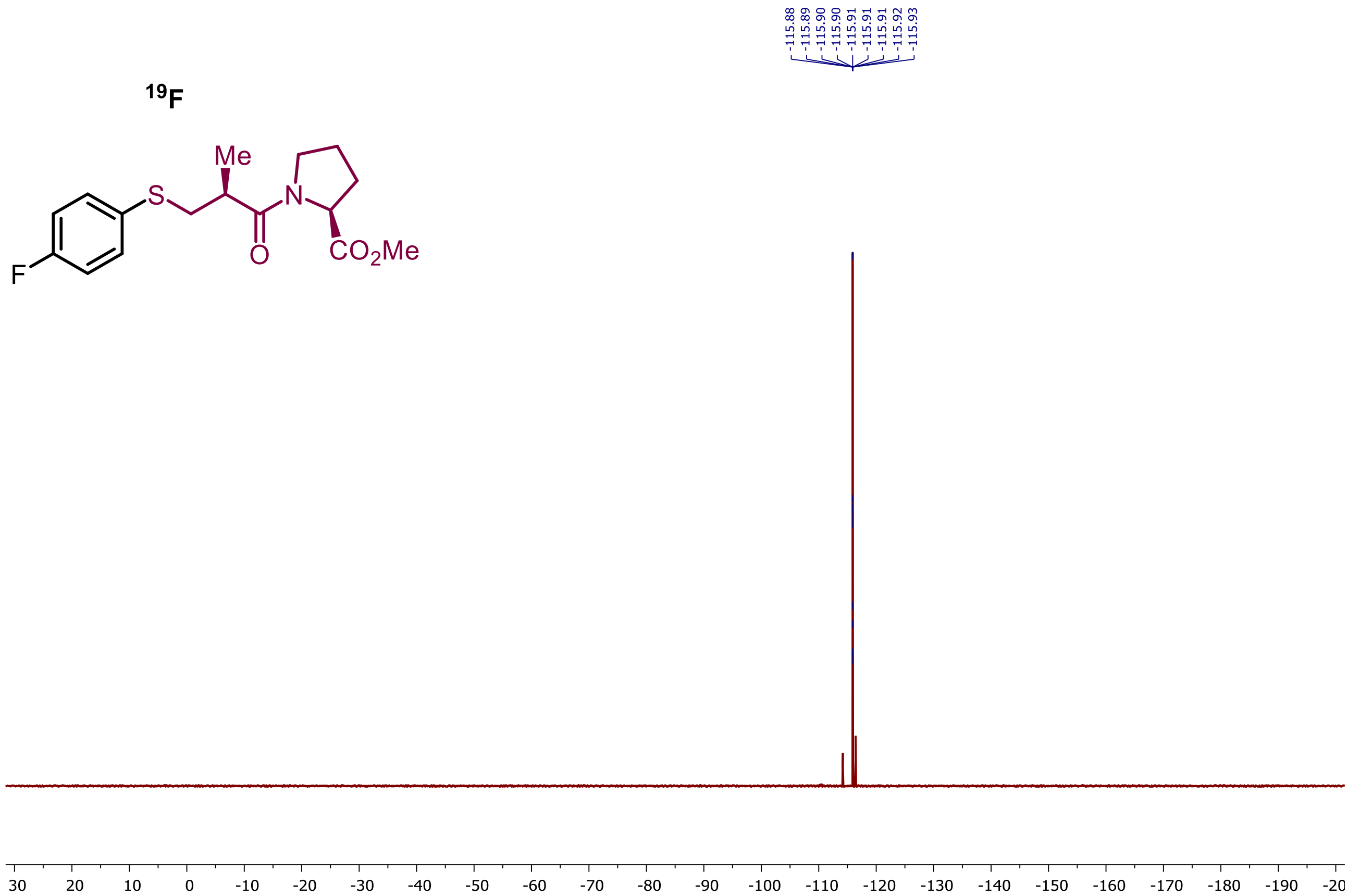
19F
-115.88
-115.89
-115.90
-115.90
-115.91
-115.91
-115.91
-115.92
-115.93
Me
S
N
F
O
CO2Me
30 20 10 0 -10 -20 -30 -40 -50 -60 -70 -80 -90 -100 -110 -120 -130 -140 -150 -160 -170 -180 -190 -20

Triethyl(4-(*p*-tolylthio)phenyl)germane (29)

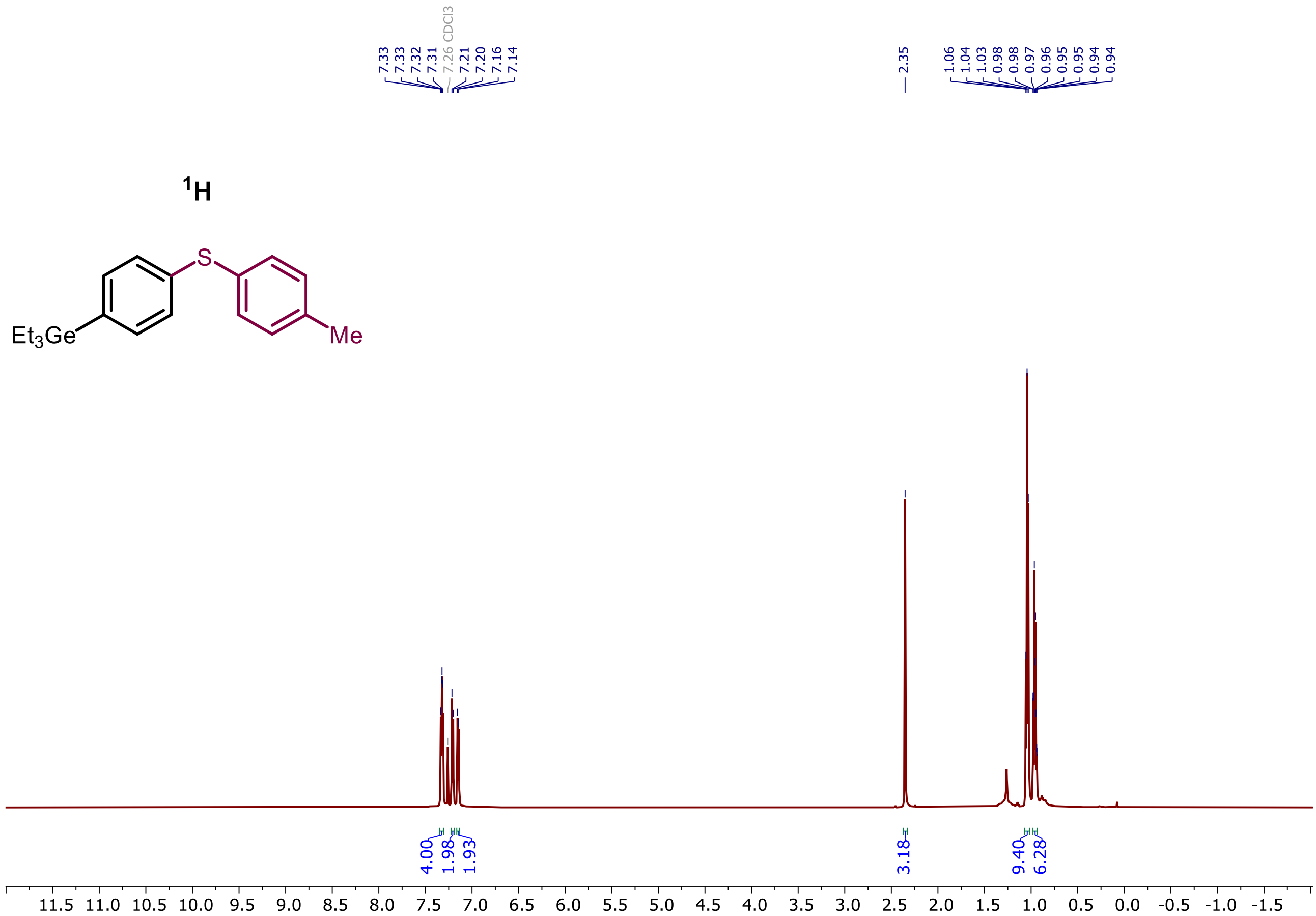

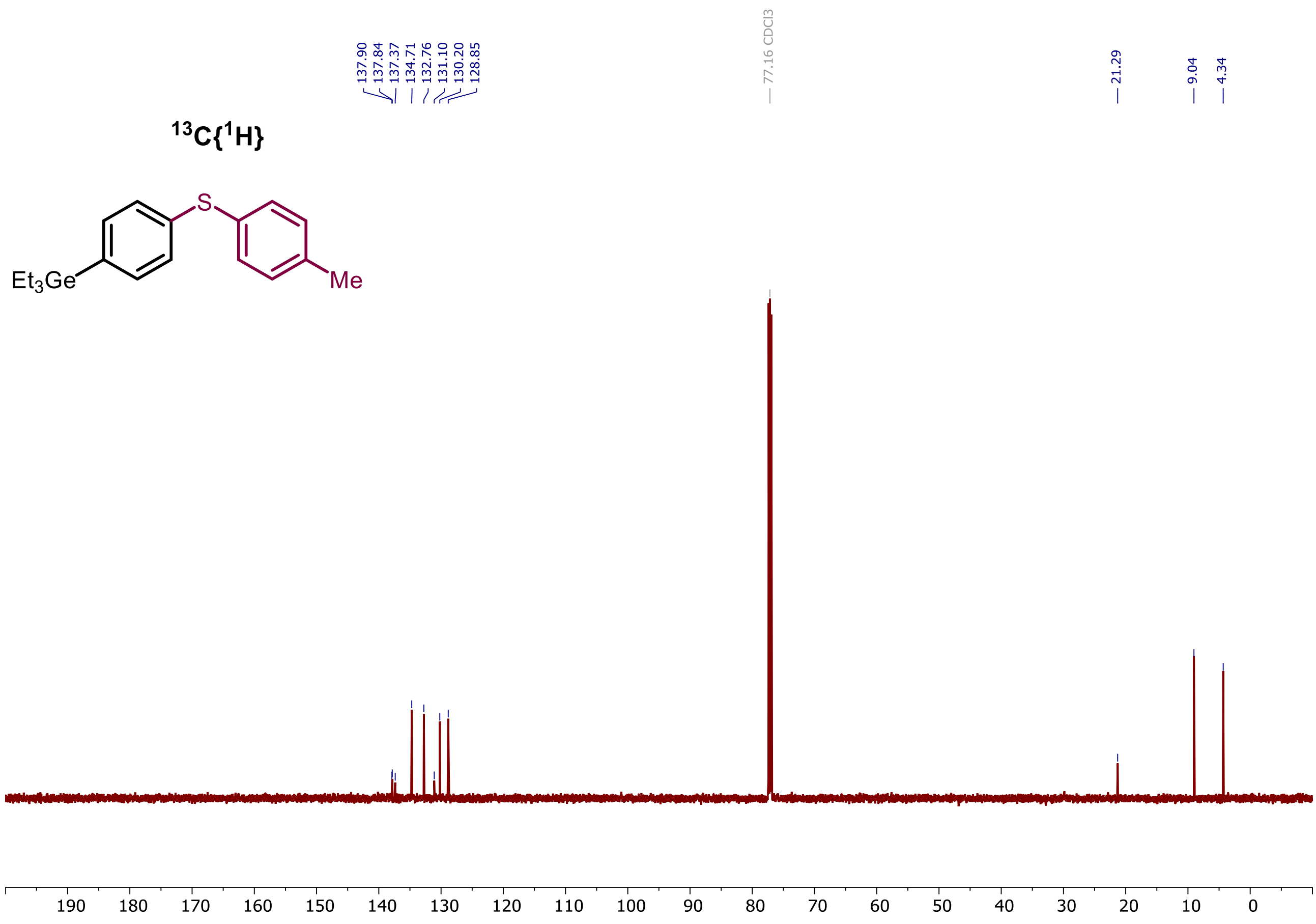

Triethyl(4-((4-fluorophenyl)thio)phenyl)germane (30)

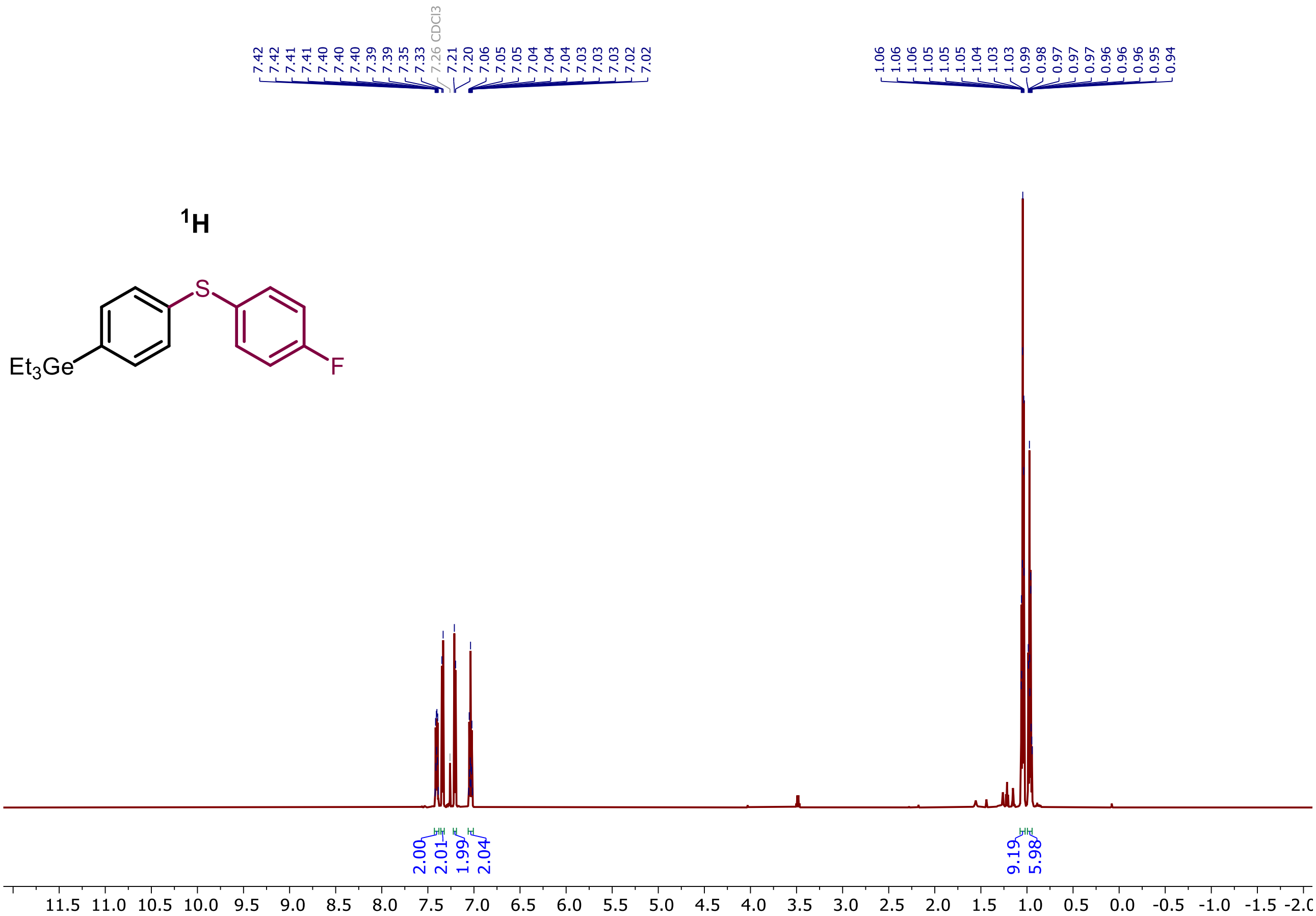

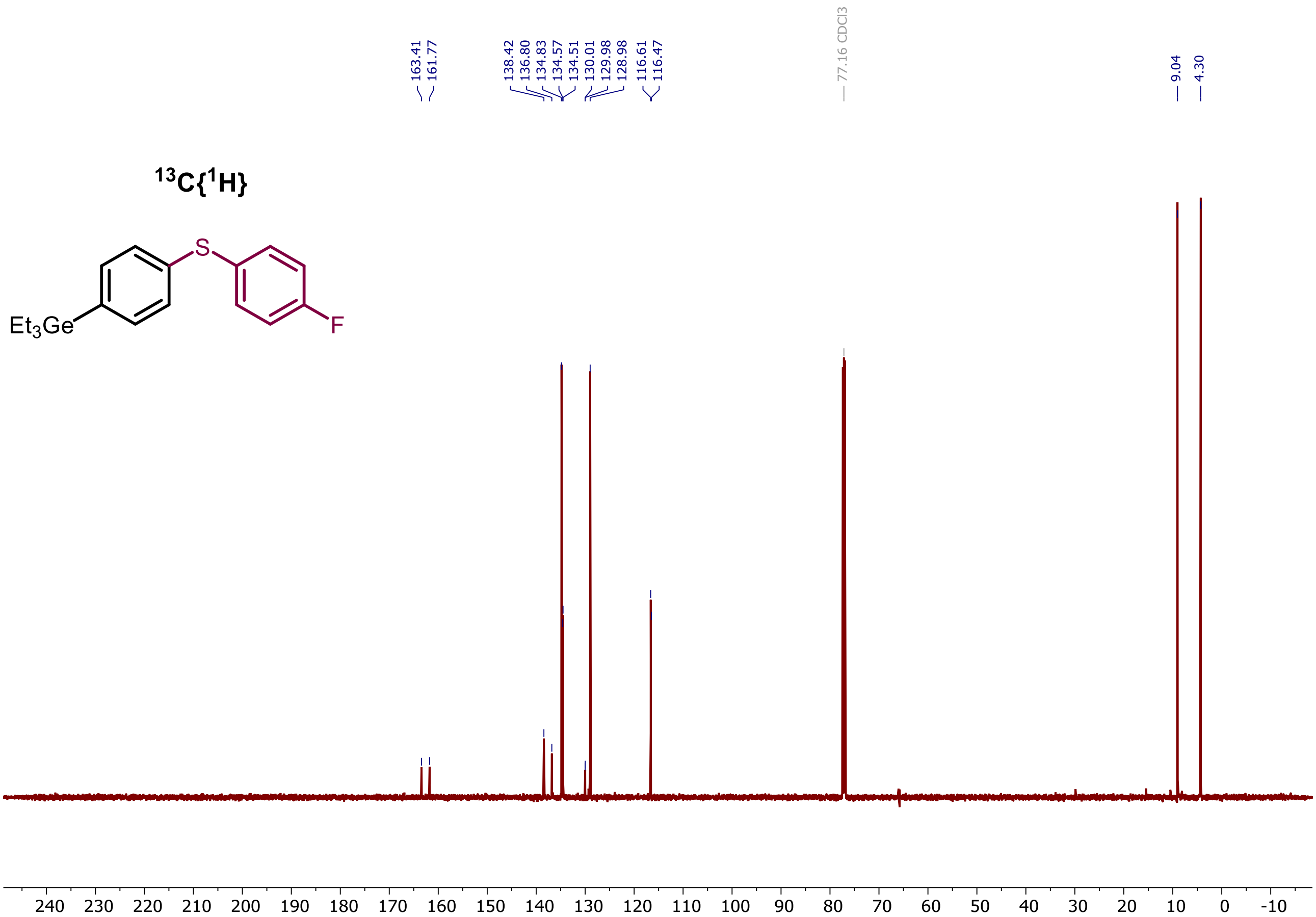

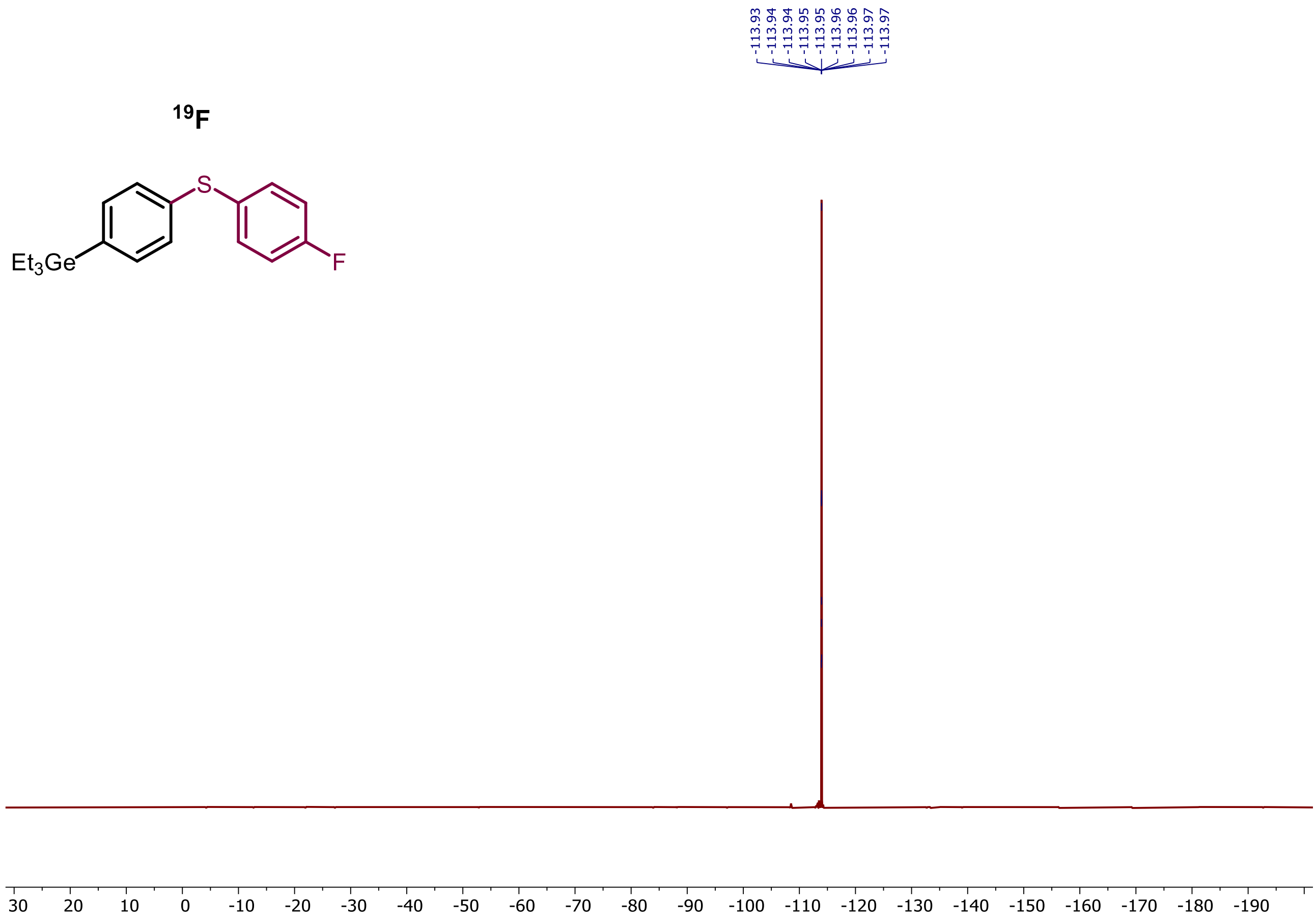

(3-((4-Fluorophenyl)thio)-5-hexylphenyl)trimethylsilane (32)

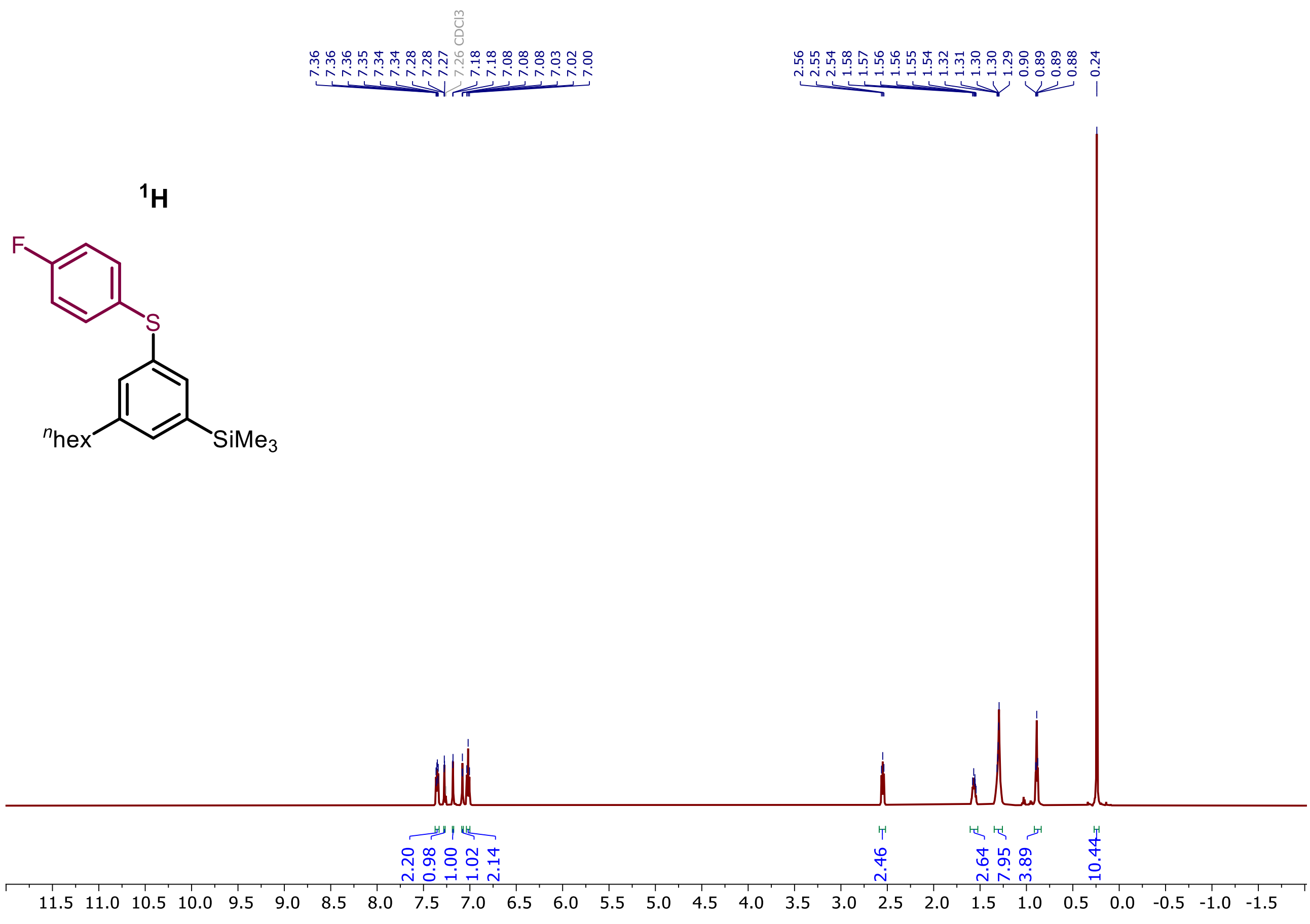

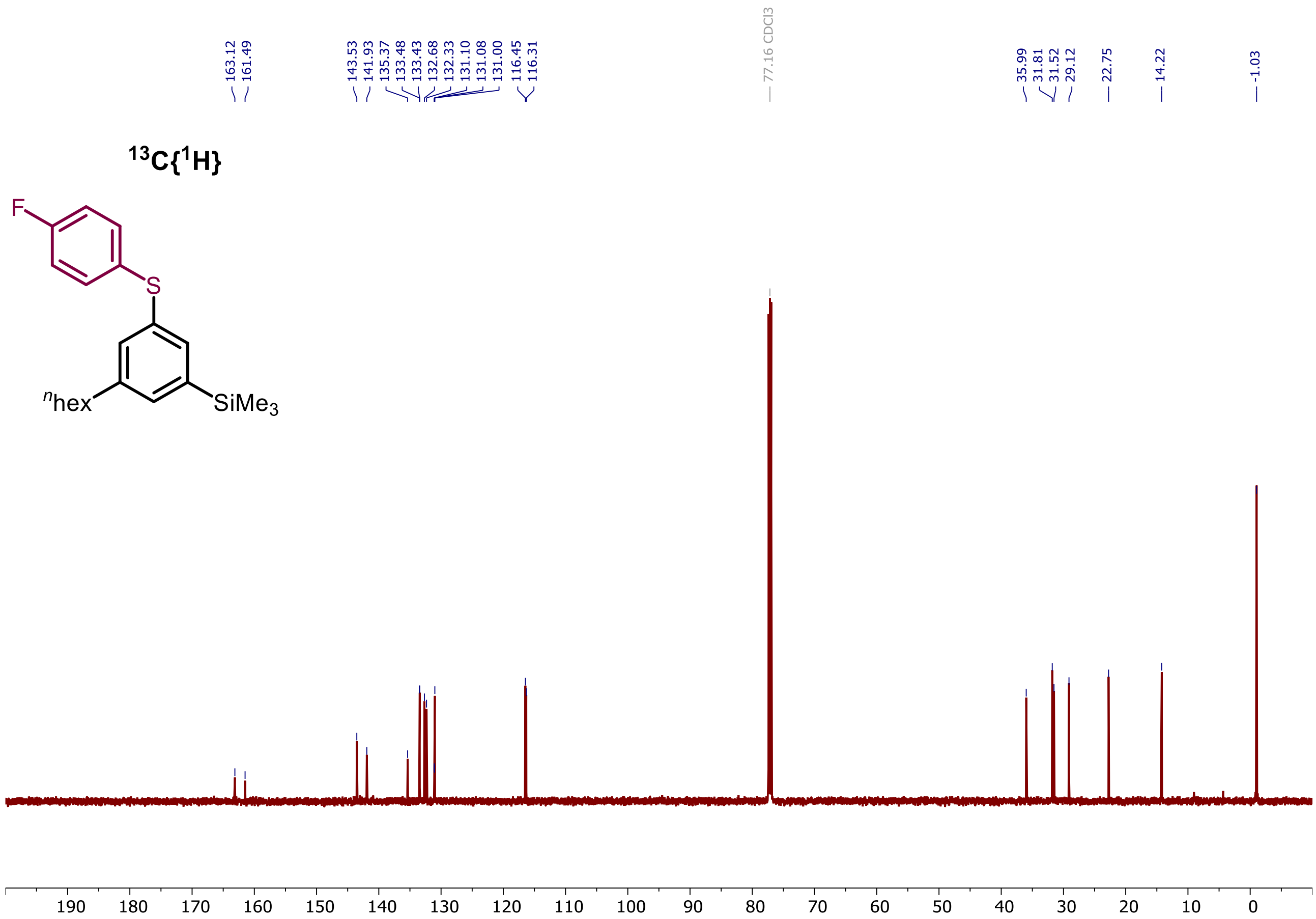

163.12
161.49
143.53
141.93
135.37
133.48
133.43
132.68
132.33
131.10
131.08
131.00
116.45
116.31
77.16 CDCl3
35.99
31.81
31.52
29.12
22.75
14.22
-1.03
$^{13}C\{^1H\}$
F
S
^{n}hex
$SiMe_3$
190 180 170 160 150 140 130 120 110 100 90 80 70 60 50 40 30 20 10 0

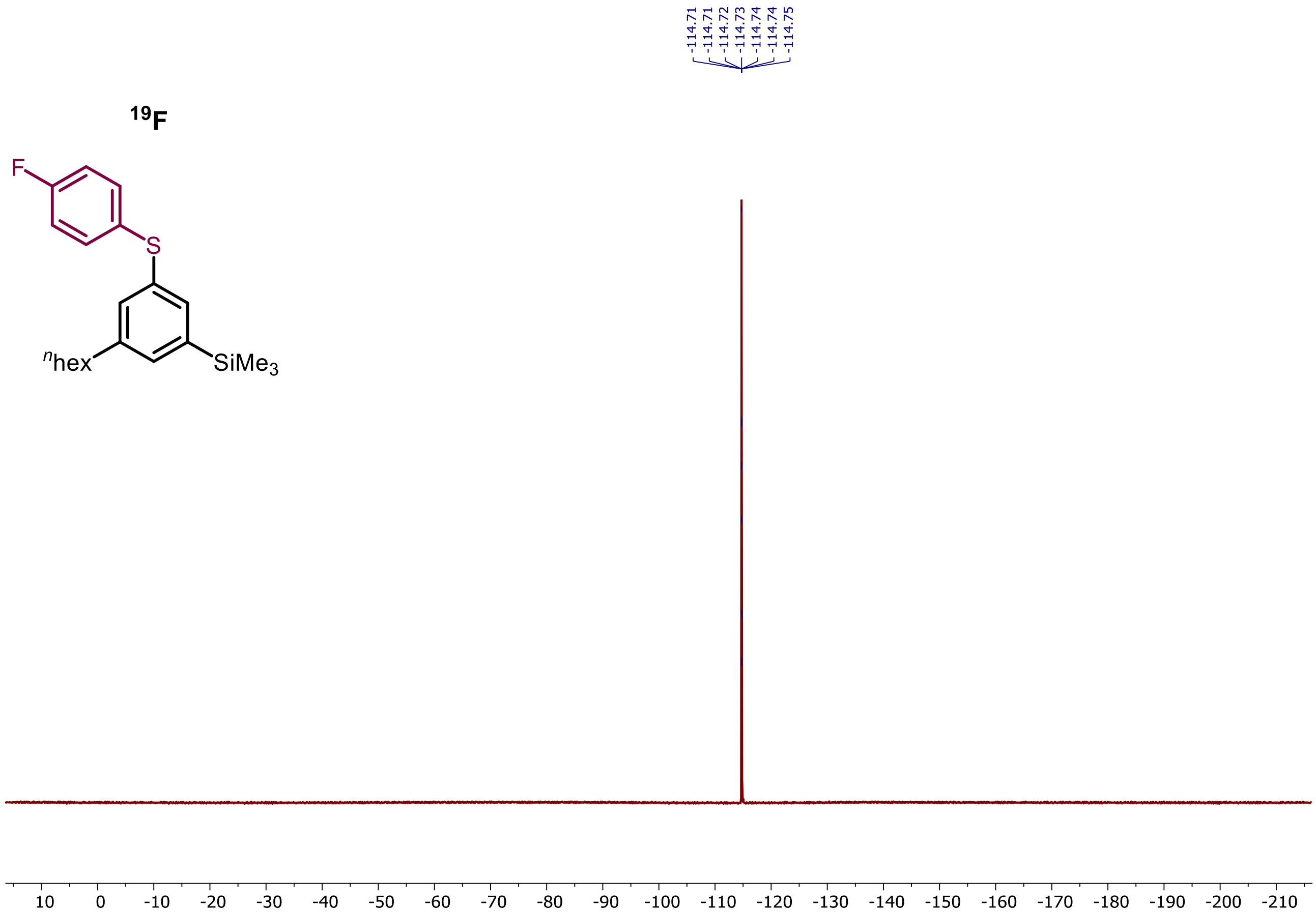

-114.71
-114.71
-114.72
-114.73
-114.74
-114.74
-114.75
^{19}F
F
S
^{n}hex
$SiMe_3$
10 0 -10 -20 -30 -40 -50 -60 -70 -80 -90 -100 -110 -120 -130 -140 -150 -160 -170 -180 -190 -200 -210

(4-Fluorophenyl)(3-hexyl-5-iodophenyl)sulfane (33)

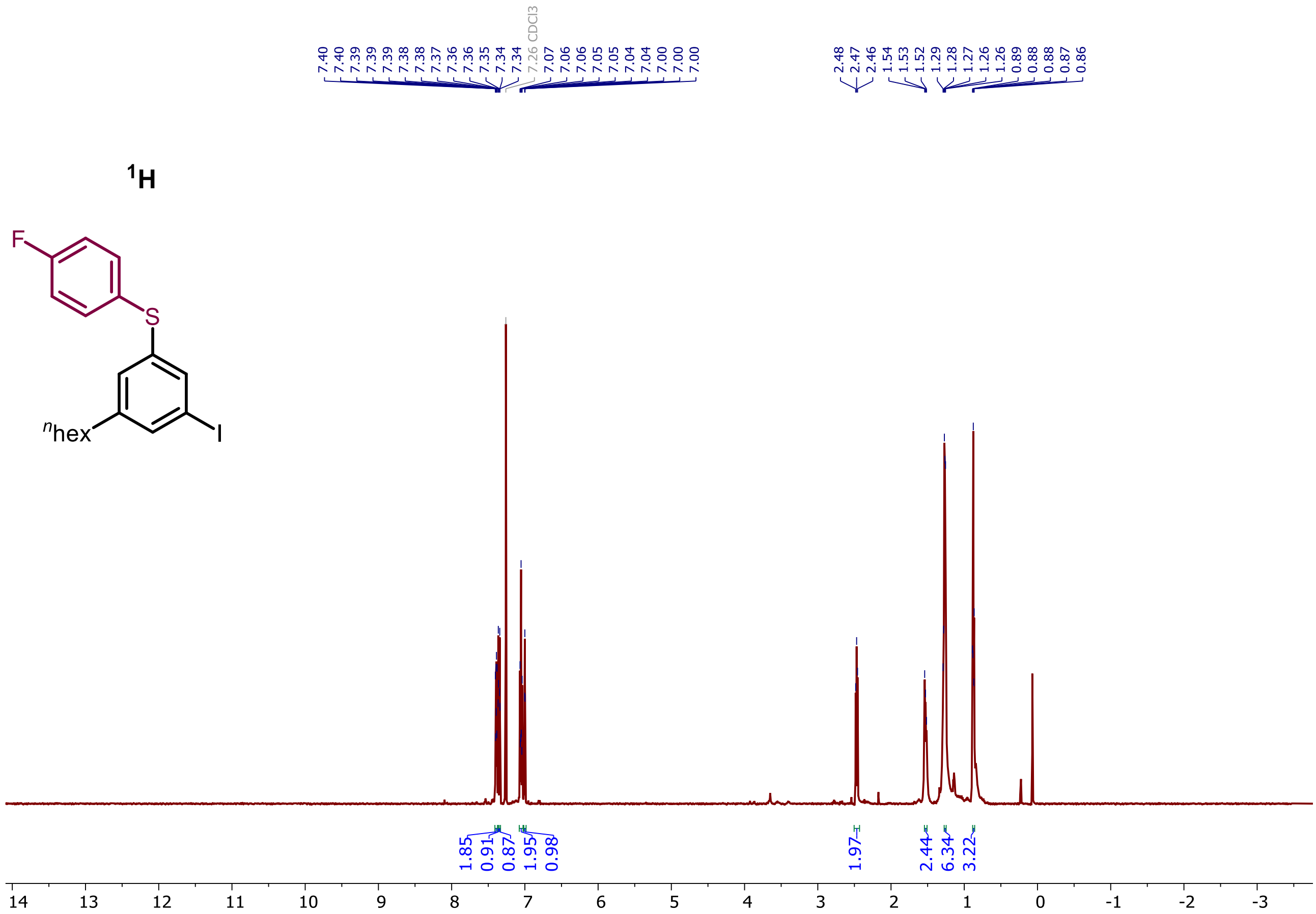

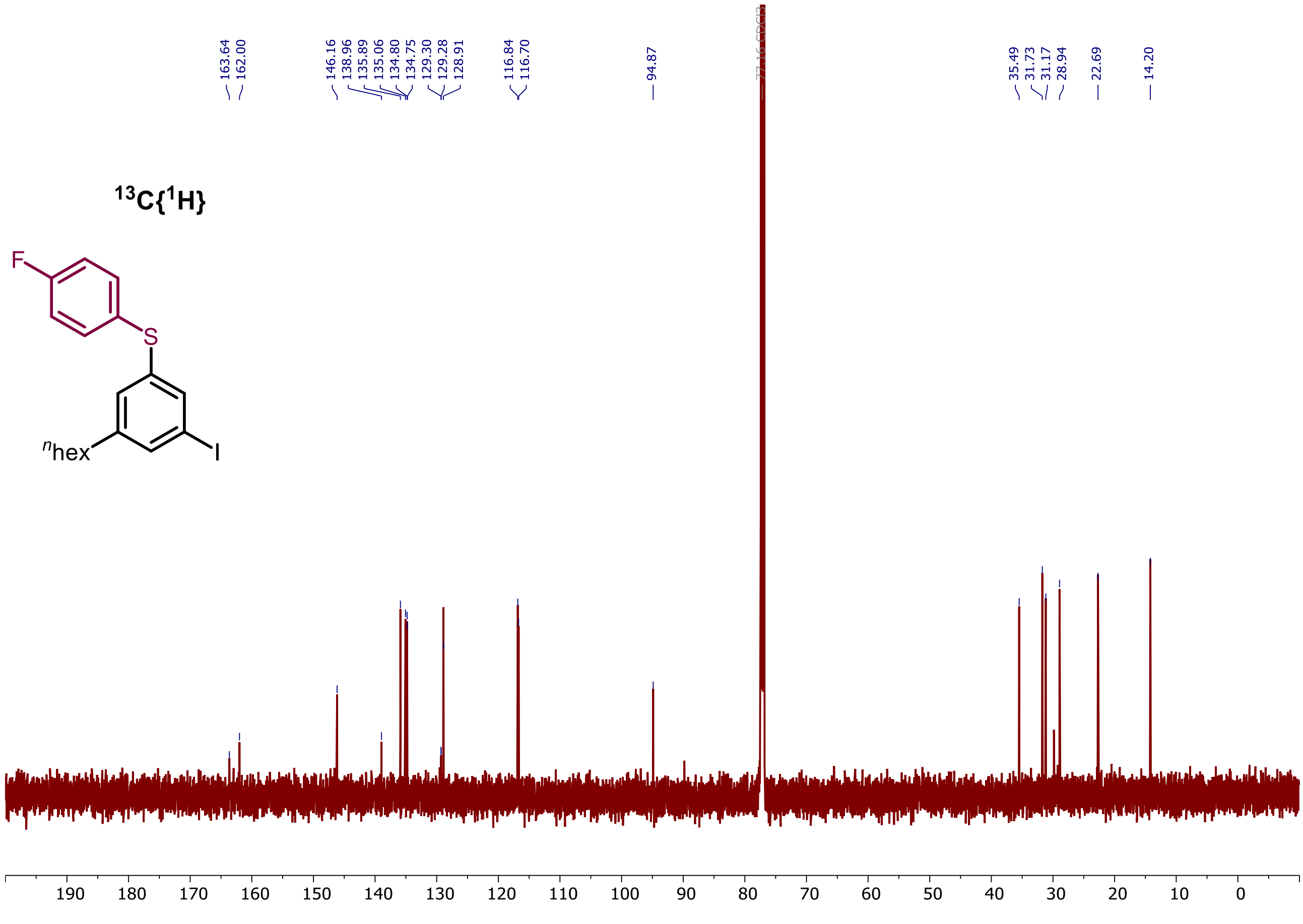

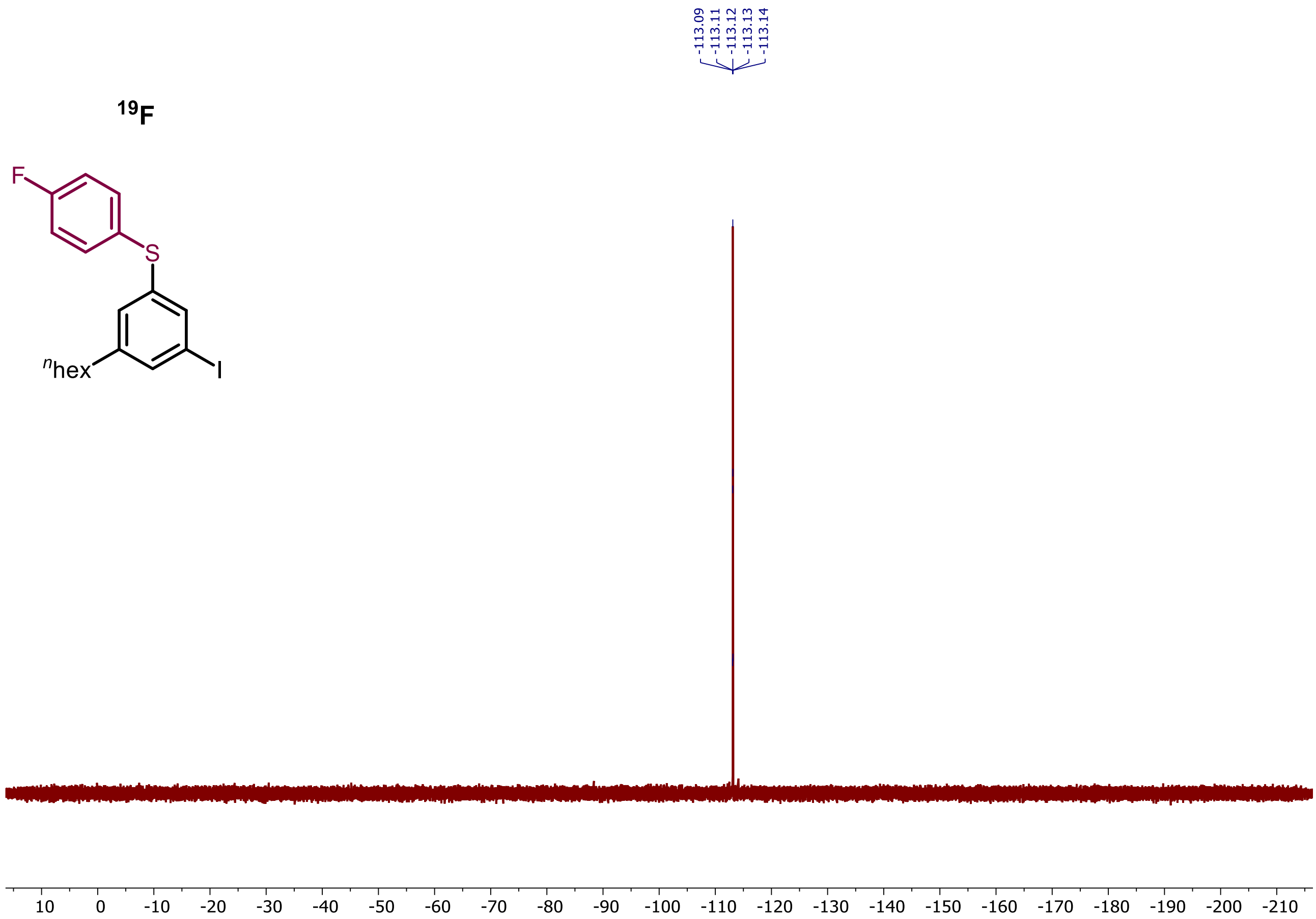

(2,2-diphenylvinyl)(4-fluorophenyl)sulfane

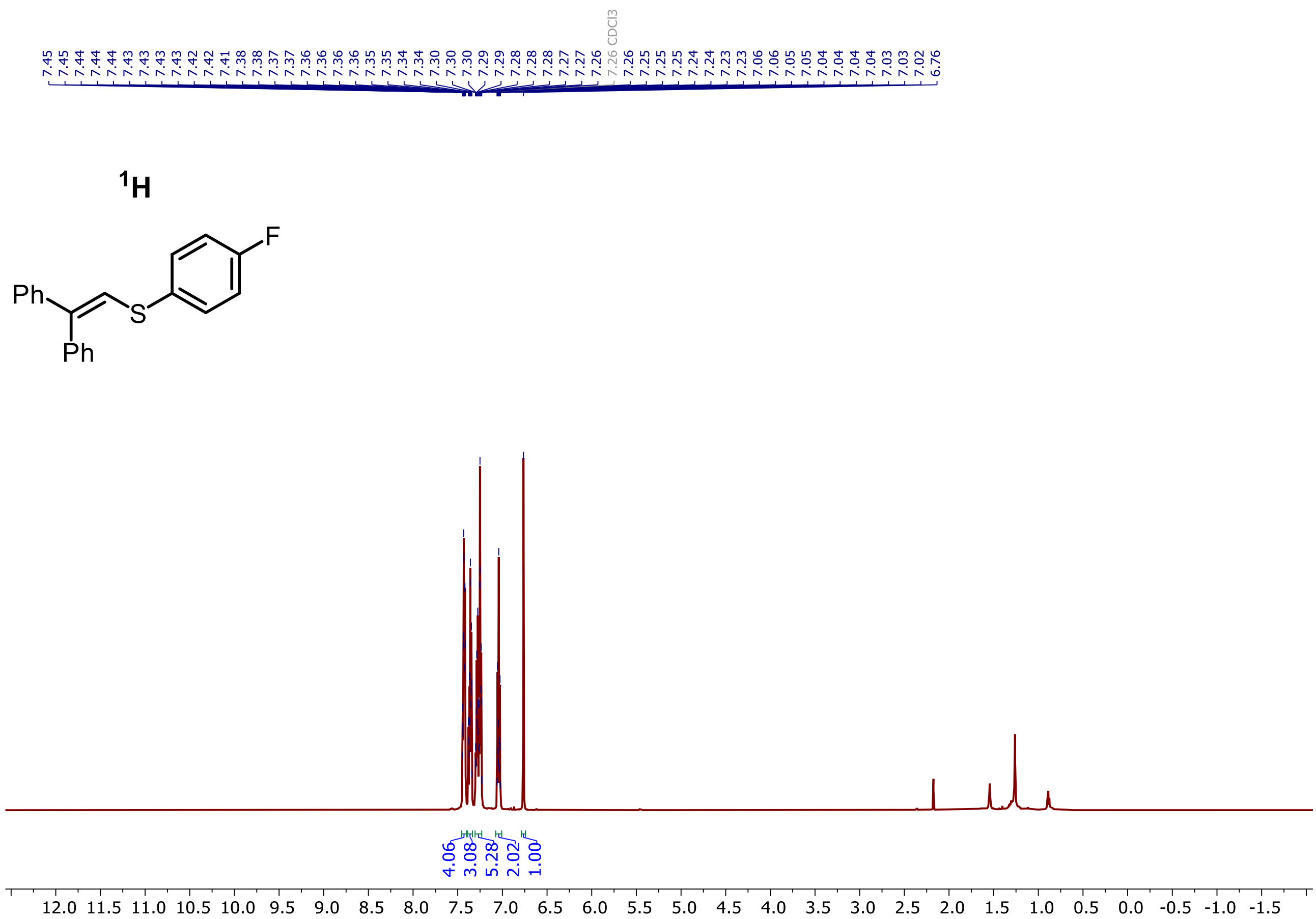

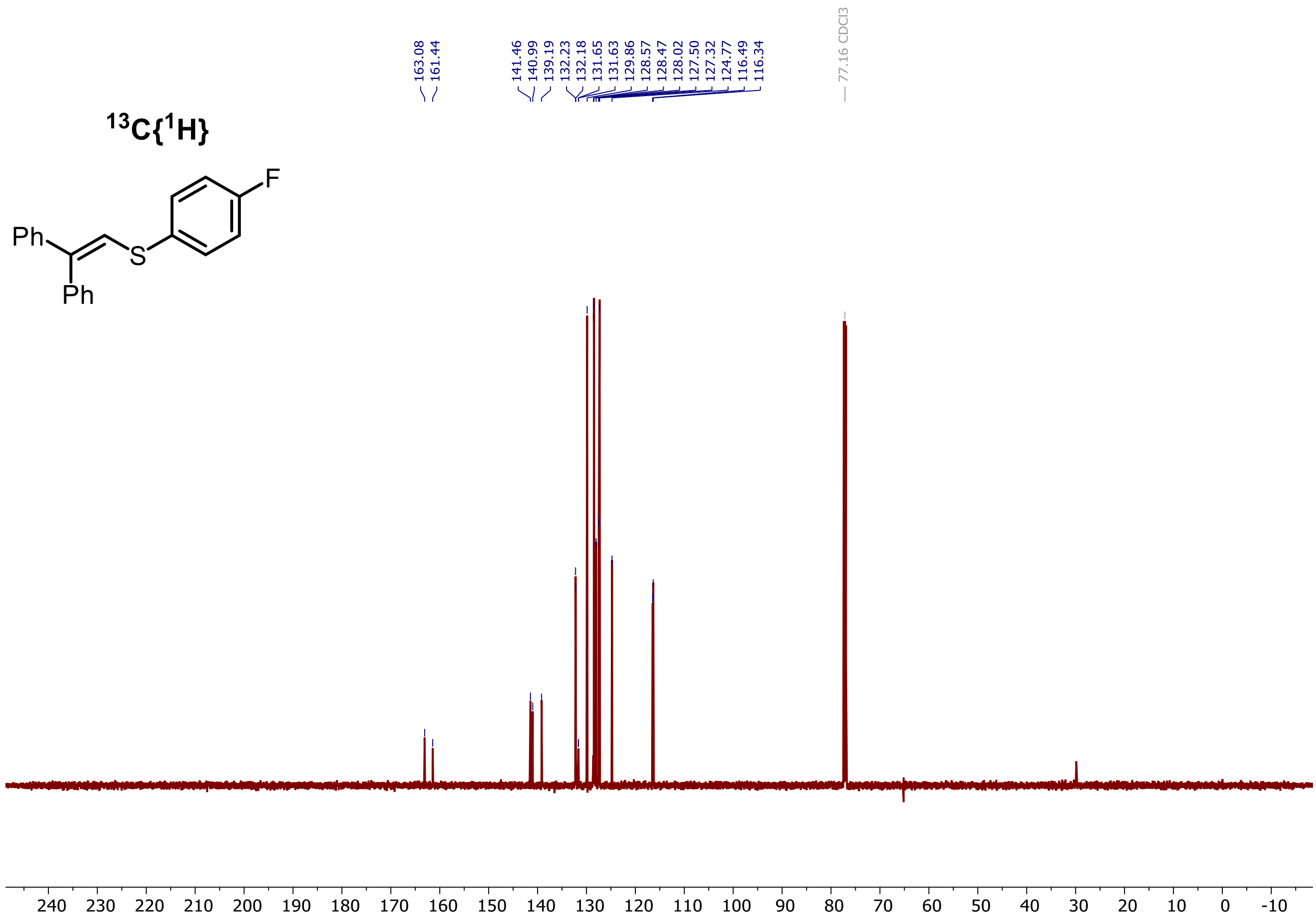

13C{1H}
Ph
Ph
S
F
163.08
161.44
141.46
140.99
139.19
132.23
132.18
131.65
131.63
129.86
128.57
128.47
128.02
127.50
127.32
124.77
116.49
116.34
77.16 CDCl3
240 230 220 210 200 190 180 170 160 150 140 130 120 110 100 90 80 70 60 50 40 30 20 10 0 -10

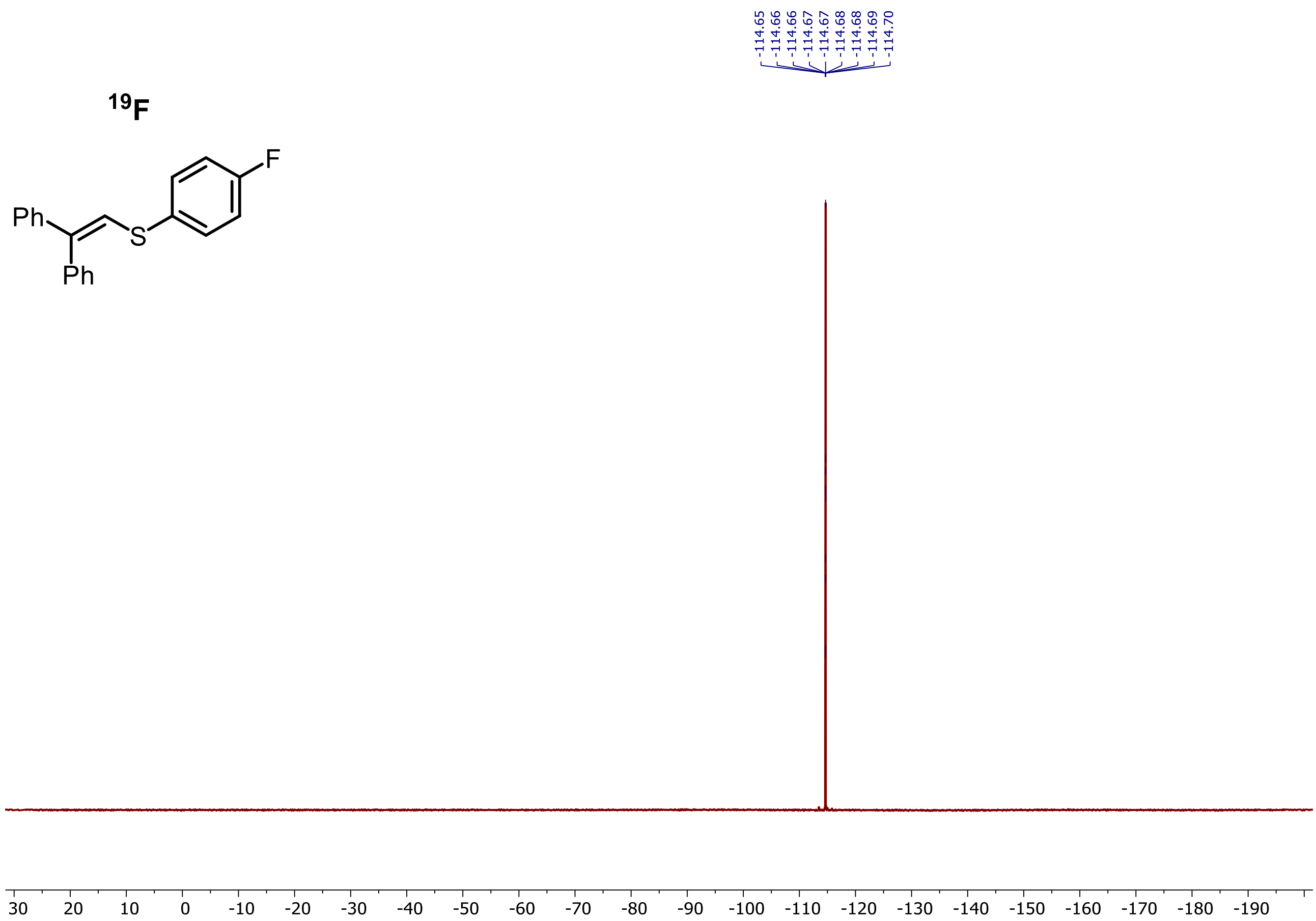

19F
Ph
Ph
S
F
-114.65
-114.66
-114.66
-114.67
-114.67
-114.68
-114.68
-114.69
-114.70
30 20 10 0 -10 -20 -30 -40 -50 -60 -70 -80 -90 -100 -110 -120 -130 -140 -150 -160 -170 -180 -190

(4-(((4-fluorophenyl)thio)methyl)tetrahydrofuran-3-yl)methyl 2,2,2-trifluoroacetate

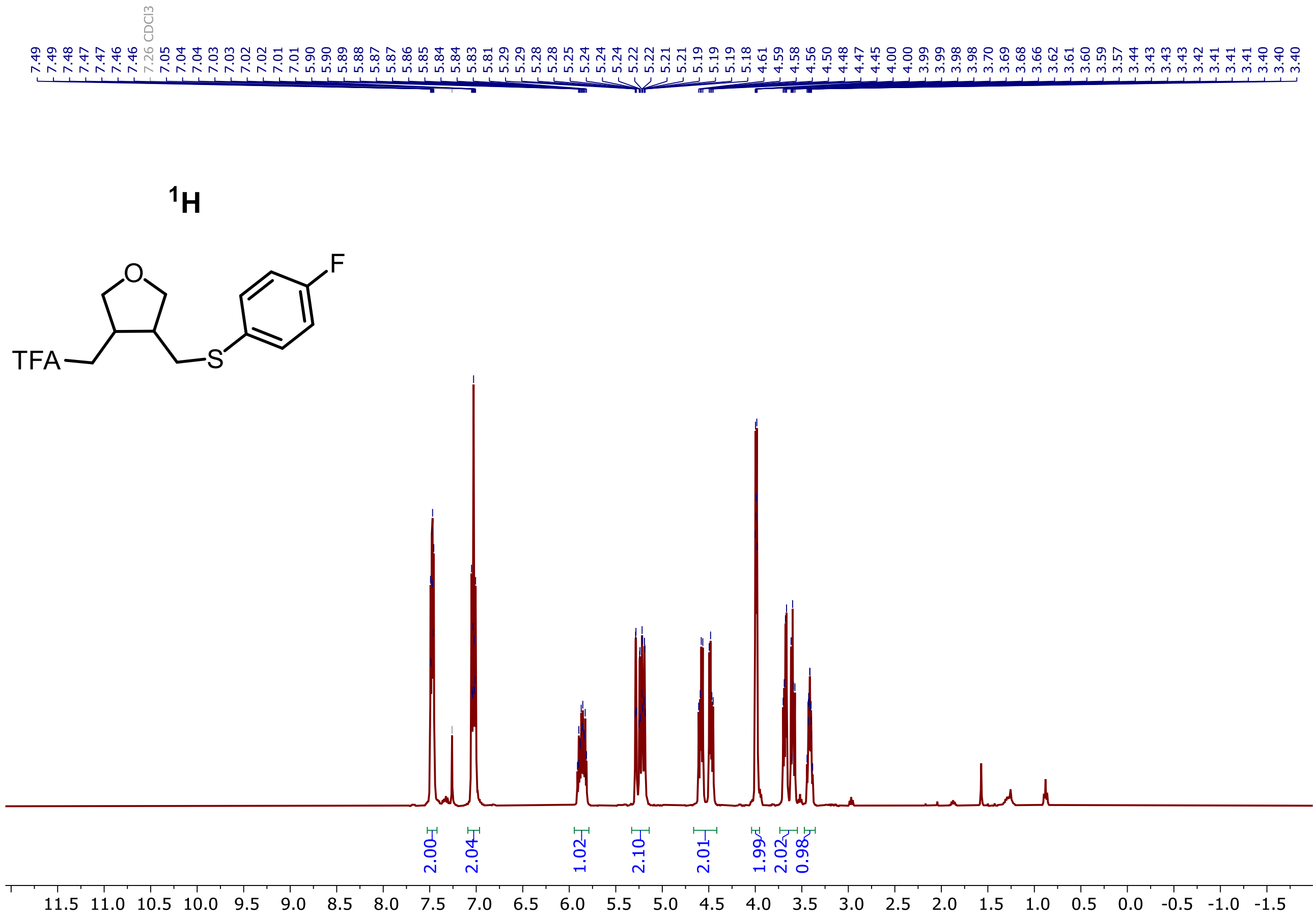

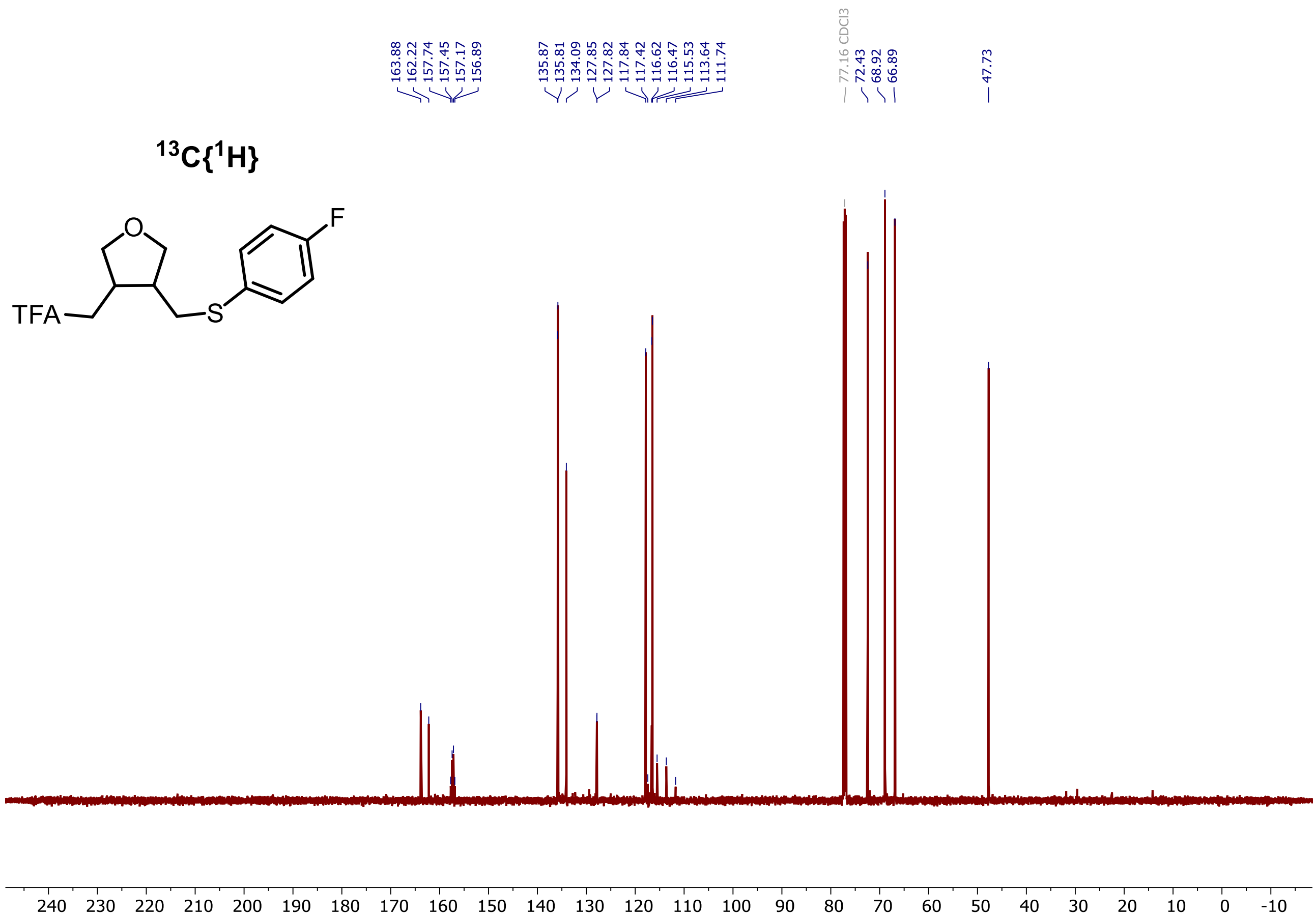

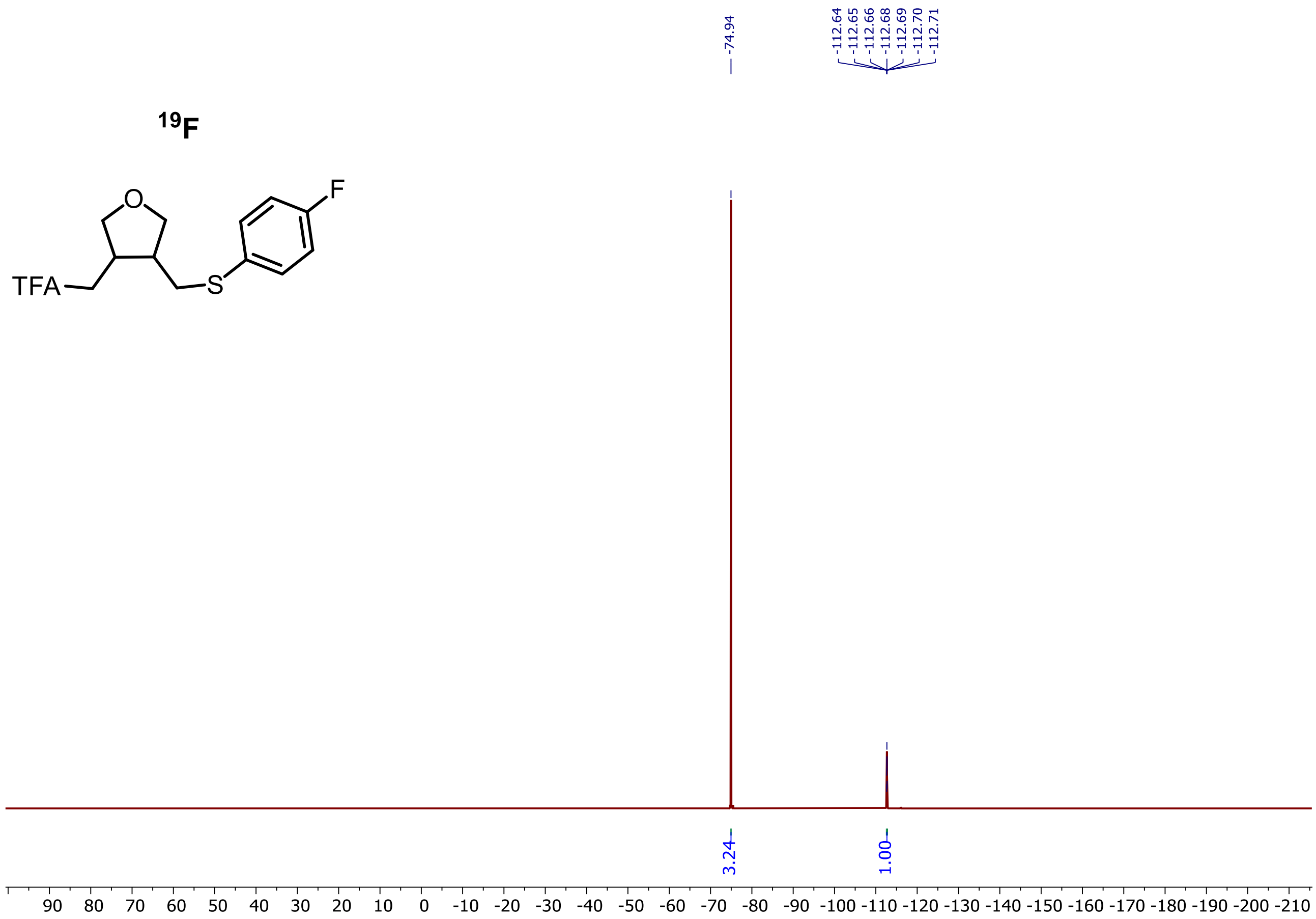
19F
TFA
O
S
F
-74.94
-112.64
-112.65
-112.66
-112.68
-112.69
-112.70
-112.71
3.24
1.00
90 80 70 60 50 40 30 20 10 0 -10 -20 -30 -40 -50 -60 -70 -80 -90 -100 -110 -120 -130 -140 -150 -160 -170 -180 -190 -200 -210

8. References

1. C. Fricke, K. Deckers, F. Schoenebeck, "Orthogonal Stability and Reactivity of Aryl Germanes Enables Rapid and Selective (Multi)Halogenations," *Angewandte Chemie International Edition* 59 (2020): 18717-18722, https://doi.org/10.1002/anie.202008372.

2. A. Dahiya, F. Schoenebeck, "Base-Mediated Direct C–H Germylation of Heteroarenes and Arenes," *Organic Letters* 23 (2021): 6010-6013, https://doi.org/10.1021/acs.orglett.1c02079.

3. G. J. Sherborne, A. G. Gevondian, I. Funes-Ardoiz, A. Dahiya, C. Fricke, F. Schoenebeck, "Modular and Selective Arylation of Aryl Germanes (C–$GeEt_3$) over C–Bpin, C–SiR_3 and Halogens Enabled by Light-Activated Gold Catalysis," *Angewandte Chemie International Edition* 59 (2020): 15543-15548, https://doi.org/10.1002/anie.202005066.

4. C. Fricke, G. J. Sherborne, I. Funes-Ardoiz, E. Senol, S. Guven, F. Schoenebeck, "Orthogonal Nanoparticle Catalysis with Organogermanes," *Angewandte Chemie International Edition* 58 (2019): 17788-17795, https://doi.org/10.1002/anie.201910060.

5. A. Dahiya, A. G. Gevondian, F. Schoenebeck, "Orthogonal C–O Bond Construction with Organogermanes," *Journal of the American Chemical Society* 145 (2023): 7729-7735, https://doi.org/10.1021/jacs.3c01081.

6. A. Dahiya, C. Fricke, F. Schoenebeck, "Gold-Catalyzed Chemoselective Couplings of Polyfluoroarenes with Aryl Germanes and Downstream Diversification," *Journal of the American Chemical Society* 142 (2020): 7754-7759, https://doi.org/10.1021/jacs.0c02860.

7. S. Langle, F. David-Quillot, A. Balland, M. Abarbri, A. Duchêne, "General access to para-substituted styrenes," *Journal of Organometallic Chemistry* 671 (2003): 113-119, https://doi.org/10.1016/S0022-328X(03)00053-6.

8. H. Xie, B. Breit, "Nickel-Catalyzed Regioselective Hydrothiolation of Allenes Enabled by Visible-Light Photoredox Catalysis," *Organic Letters* 26 (2024): 4438-4442, https://doi.org/10.1021/acs.orglett.4c01027.

9. B. Li, J. Wang, G. Tu, G. Ju, K. Zhou, Y. Zhao, "Hemin-Promoted Direct C–H Thiolation in the Synthesis of Diaryl Sulfides," 26 (2023): e202201458, https://doi.org/10.1002/ejoc.202201458.

10. S. P. Bakare, M. Patil, "Thiolate-assisted copper(i) catalyzed C–S cross coupling of thiols with aryl iodides: scope, kinetics and mechanism," *New Journal of Chemistry* 46 (2022): 6283-6295, https://doi.org/10.1039/D2NJ00043A.

11. T. Hostier, V. Ferey, G. Ricci, D. Gomez Pardo, J. Cossy, "Synthesis of Aryl Sulfides: Metal-Free C–H Sulfenylation of Electron-Rich Arenes," *Organic Letters* 17 (2015): 3898-3901, https://doi.org/10.1021/acs.orglett.5b01889.

12. T. Scattolin, E. Senol, G. Yin, Q. Guo, F. Schoenebeck, "Site-Selective C–S Bond Formation at C–Br over C–OTf and C–Cl Enabled by an Air-Stable, Easily Recoverable, and Recyclable Palladium(I) Catalyst," *Angewandte Chemie International Edition* 57 (2018): 12425-12429, https://doi.org/10.1002/anie.201806036.

13. C. G. Bates, R. K. Gujadhur, D. Venkataraman, "A General Method for the Formation of Aryl−Sulfur Bonds Using Copper(I) Catalysts," *Organic Letters* 4 (2002): 2803-2806, https://doi.org/10.1021/ol0264105.

14. J. Zhang, C. M. Medley, J. A. Krause, H. Guan, "Mechanistic Insights into C−S Cross-Coupling Reactions Catalyzed by Nickel Bis(phosphinite) Pincer Complexes," *Organometallics* 29 (2010): 6393-6401, https://doi.org/10.1021/om100816d.

15. B. Liu, C.-H. Lim, G. M. Miyake, "Visible-Light-Promoted C–S Cross-Coupling via Intermolecular Charge Transfer," *Journal of the American Chemical Society* 139 (2017): 13616-13619, https://doi.org/10.1021/jacs.7b07390.

16. I. Kalvet, T. Sperger, T. Scattolin, G. Magnin, F. Schoenebeck, "Palladium(I) Dimer Enabled Extremely Rapid and Chemoselective Alkylation of Aryl Bromides over Triflates and Chlorides in Air," *Angewandte Chemie International Edition* 56 (2017): 7078-7082, https://doi.org/10.1002/anie.201701691.

17. M. J. Frisch, G. W. Trucks, H. B. Schlegel*, et al., Gaussian 16, Revision A.03*, Gaussian, Inc., Wallingford, CT. (2016).
